# Synthesis of imidazo[4,5-*e*][1,3]thiazino[2,3-*c*][1,2,4]triazines via a base-induced rearrangement of functionalized imidazo[4,5-*e*]thiazolo[2,3-*c*][1,2,4]triazines

**DOI:** 10.3762/bjoc.19.80

**Published:** 2023-07-28

**Authors:** Dmitry B Vinogradov, Alexei N Izmest’ev, Angelina N Kravchenko, Yuri A Strelenko, Galina A Gazieva

**Affiliations:** 1 N. D. Zelinsky Institute of Organic Chemistry, Russian Academy of Sciences, 47 Leninsky Prosp., Moscow 119991, Russian Federation

**Keywords:** N,S-heterocycles, ring expansion, skeletal rearrangement, 1,3-thiazines, thiazolidine-4-ones

## Abstract

A series of imidazo[4,5-*e*][1,3]thiazino[2,3-*c*][1,2,4]triazines was synthesized via a cascade sequence of hydrolysis and skeletal rearrangement of imidazo[4,5-*e*]thiazolo[2,3-*c*][1,2,4]triazin-7(8*H*)-ylidene)acetic acid esters in methanol upon treatment with excess KOH. Imidazo[4,5-*e*]thiazolo[3,2-*b*][1,2,4]triazin-6(7*H*)-ylidene)acetic acid esters are also suitable substrates for the reaction. In this case hydrolysis and thiazole ring expansion were accompanied with the change of the thiazolotriazine junction type from thiazolo[3,2-*b*][1,2,4]triazine to thiazino[2,3-*c*][1,2,4]triazine.

## Introduction

**Figure 1:**
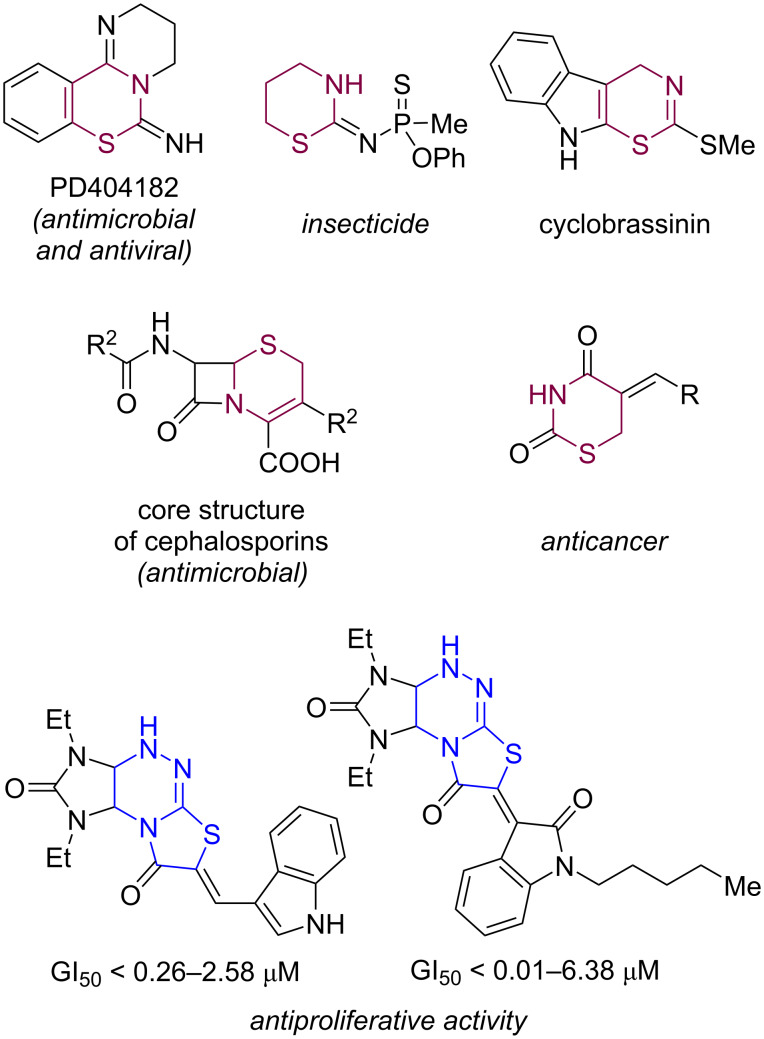
Examples of natural and synthetic bioactive 1,3-thiazine and imidazothiazolotriazine derivatives with high antiproliferative activity.

Nitrogen- and sulfur-containing heterocyclic compounds are widely represented in nature and used for the synthesis of biologically active substances. Among the 1,3-thiazine derivatives, promising compounds as antimicrobial and antiviral drugs (PD404182) [1–4], sedative [5] and antitumor agents [6–8], as well as fungicides [9–10] and insecticides [11] have been found (Figure 1). The 1,3-thiazine heterocyclic system is comprised in some natural phytoalexins (cyclobrassinin, sinalbins A and B, rutalexin, and others) [12] and 7-aminocephalosporanic acid (7-ACA), which is a key fragment of broad-spectrum cephalosporin antibiotics [13–14].

Condensed 1,2,4-triazines attract attention of researchers due to their diverse biological activities [15] and also their application as starting materials for the constructing of new heterocyclic systems [16–17]. Recent studies of the antitumor activity of imidazo[4,5-*e*]thiazolo[2,3-*c*]-1,2,4-triazines revealed a number of compounds with a high antiproliferative effect towards a large number of human cancer cell lines (Figure 1) [18–19]. Therefore, the synthesis of new imidazothiazolotriazines and closely related hybrid compounds including fragments of 1,3-thiazine and imidazo-1,2,4-triazine is still highly relevant.

**Scheme 1:**
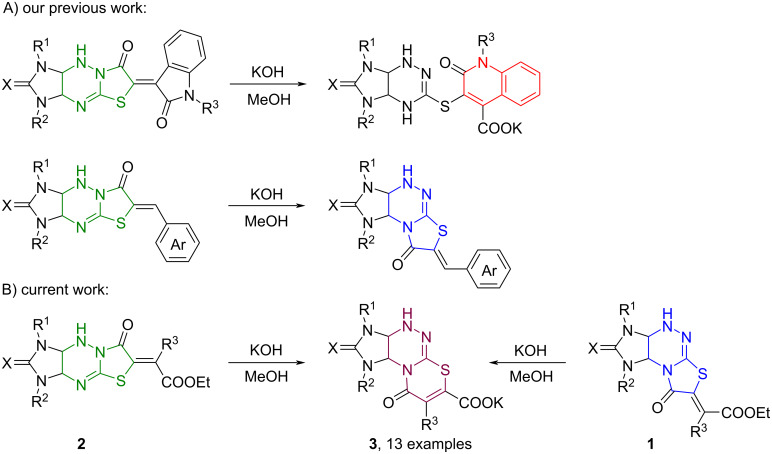
Base-induced transformations and rearrangements of functionalized imidazo[4,5-*e*]thiazolo[3,2-*b*]-1,2,4-triazine derivatives into new heterocyclic systems.

Earlier we have demonstrated that imidazo[4,5-*e*]thiazolo[3,2-*b*]-1,2,4-triazines and their derivatives functionalized at position 6 are capable of undergoing skeletal rearrangements and transformations of the heterocyclic system proceeding by an ANRORC mechanism under the action of KOH in methanol. Thus, 6-oxindolylideneimidazo[4,5-*e*]thiazolo[3,2-*b*]-1,2,4-triazines are transformed into substituted 2-oxoquinoline-4-carboxylates in the presence of excess KOH [20] while their 6-arylmethylidene derivatives undergo rearrangement into the corresponding isomeric derivatives of imidazo[4,5-*e*]thiazolo[2,3-*c*]-1,2,4-triazine [18,21] (Scheme 1A).

In the present study, we report a new base-induced recyclization of functionalized imidazothiazolotriazines **1** and **2** resulting in derivatives of the new heterocyclic system, namely imidazo[4,5-*e*][1,3]thiazino[2,3-*c*][1,2,4]triazines **3** (Scheme 1B).

## Results and Discussion

**Scheme 2:**
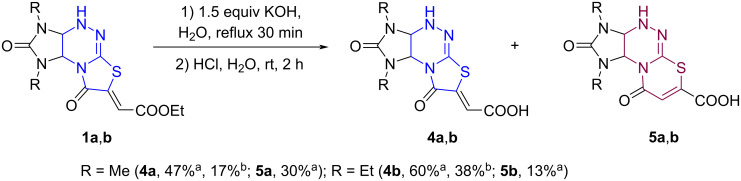
Alkaline hydrolysis of esters **1a**,**b**. ^a^Determined by ^1^H NMR spectroscopy; ^b^isolated yields.

In a continuation of our studies [22] aimed at the synthesis of functionalized imidazothiazolotriazine derivatives, we attempted to hydrolyze the ester group of imidazothiazolotriazines **1** and to prepare the corresponding carboxylic acids **4** using an aqueous KOH solution. Heating esters **1a**,**b** in an aqueous solution of KOH and subsequent addition of hydrochloric acid led to the corresponding acids **4a**,**b** as the main products. Acids **4a**,**b** were isolated from the mixtures in 17 and 38% yield, respectively. However, we also detected by ^1^H NMR spectroscopy the formation of other minor products, presumably derivatives of a new heterocyclic system, imidazo[4,5-*e*][1,3]thiazino[2,3-*c*][1,2,4]triazines **5a**,**b** (Scheme 2).

**Scheme 3:**
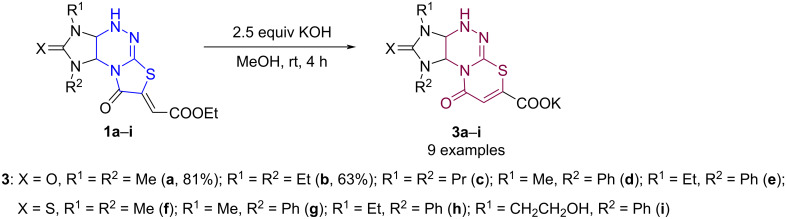
Synthesis of potassium imidazo[4,5-*e*][1,3]thiazino[2,3-*c*][1,2,4]triazine-7-carboxylates.

To prepare the new compounds **5**, the solvent, amount of KOH, reaction time, and temperature were varied. Increasing the amount of KOH and reaction time led to an increase in the yield of the potassium salts **3a**,**b** even at room temperature. It was found that stirring esters **1a–i** in methanol in the presence of 2.5 equivalents of an aqueous solution of KOH provided selective formation of imidazo[4,5-*e*][1,3]thiazino[2,3-*c*][1,2,4]triazines **3a**–**i** as a result of ester group hydrolysis and thiazolidine ring expansion to the corresponding thiazine (Scheme 3). The potassium salts **3a**,**b** were isolated in 81 and 63% yield, respectively. Compounds **3c**–**i** were used in further transformations without isolation.

**Scheme 4:**
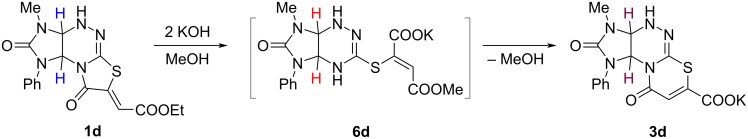
Plausible rearrangement mechanism of imidazo[4,5-*e*]thiazolo[2,3-*c*][1,2,4]triazine **1d** into imidazo[4,5-*e*][1,3]thiazino[2,3-*c*][1,2,4]triazine **3d**.

**Figure 2:**
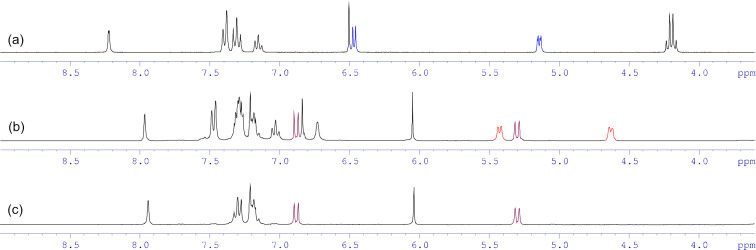
^1^H NMR spectra of the starting compound **1d** (a) and the reaction mixture after 1.5 (b) and 4 (c) hours in DMSO-*d*_6_ (the colored signals correspond to the protons shown in red in Scheme 4).

^1^H NMR reaction monitoring showed that compound **1d** under conditions of excess of KOH in methanol undergoes alkaline hydrolysis along with transesterification of the ester group to give the ring-opened form **6d** (Scheme 4), the maximum concentration of which was observed approximately 30 minutes after the start of the reaction. After an hour, the signals of the starting imidazothiazolotriazine **1d** disappeared, and after 2–4 hours of reaction, only signals of the target product **3d** were observed in the ^1^H NMR spectrum (Figure 2).

**Scheme 5:**
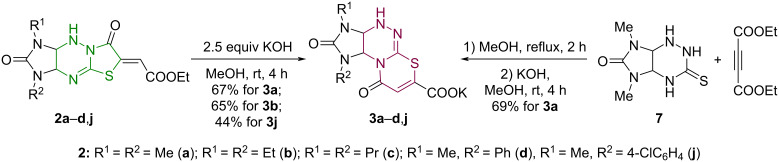
Synthetic approaches to imidazo[4,5-*e*][1,3]thiazino[2,3-*c*][1,2,4]triazines **3a**–**d**,**j**.

Compounds **3a**–**d**,**j** were prepared from imidazo[4,5-*e*]thiazolo[3,2-*b*]-1,2,4-triazines **2a**-**d**,**j** of linear structure under similar conditions (Scheme 5). The isolated yield of the potassium salt **3b** (65%) corresponded to the yield of the product obtained from the isomeric structure **1b** of the angular type (63%), while the yield of compound **3a** (67%) in the analogous reaction was inferior to that of the transformation of structure **1a** (81%). Salt **3j** was isolated in 44% yield. The absence of signals of the starting or intermediate compounds in the ^1^H NMR spectrum of the reaction mixture (for **3c**,**d**) also indicates the complete conversion of esters **2c**,**d** to the potassium salts **3c**,**d** after 4 hours of reaction.

A one-pot method for the synthesis of 1,3-dimethylimidazo[4,5-*e*][1,3]thiazino[2,3-*c*][1,2,4]triazine **3a** was exemplified by successive reactions of imidazo[4,5-*e*][1,2,4]triazine **7** with diethyl acetylenedicarboxylate and excess KOH.

**Scheme 6:**
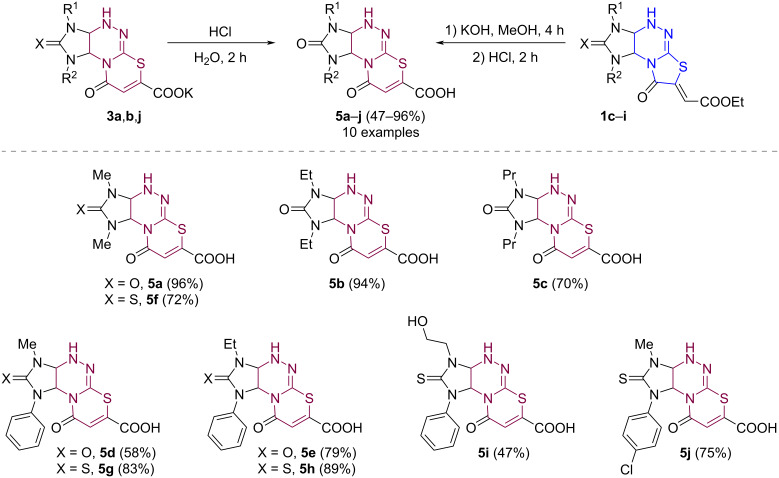
Synthesis of imidazo[4,5-*e*][1,3]thiazino[2,3-*c*][1,2,4]triazine-7-carboxylic acids **5a**–**j**.

Acidification of aqueous solutions of potassium salts **3a**,**b**,**j** or the reaction masses containing potassium salts **3c**–**i** in methanol (obtained from **1c**–**i**) with hydrochloric acid led to the formation of the corresponding 1,3-dialkyl-2,9-dioxoimidazo[4,5-*e*][1,3]thiazino[2,3-*c*][1,2,4]triazine-7-carboxylic acids **5a**–**j** in 47–96% yields (Scheme 6).

**Scheme 7:**
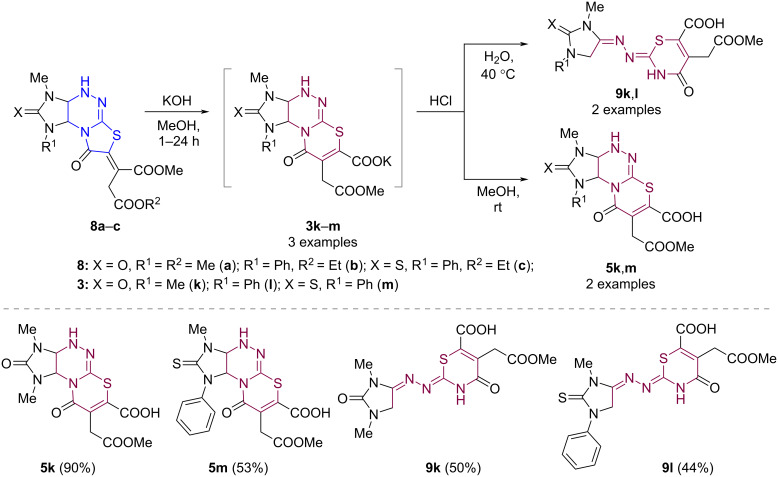
Synthesis of imidazo[4,5-*e*][1,3]thiazino[2,3-*c*][1,2,4]triazine-7-carboxylic acids **5k**,**m**.

The developed method is also applicable to substrates **8a**–**c** [23] substituted in the methylidene fragment. Thus, compounds **8a**–**c** were converted into the corresponding potassium salts **3k**–**m** under the same conditions (MeOH, room temperature, 1–24 h) (Scheme 7). However, acidification of aqueous solutions of the salts **3k**–**m** with excess hydrochloric acid and further evaporation of the solvent at 40 °C led to decomposition products, two of which were isolated and characterized by NMR spectroscopy including 2D experiments and HRMS data. The target acids **5k**,**m** were obtained using equivalent amounts of HCl at room temperature. however, acid **5l** underwent partial transformations even under these conditions and was not isolated as individual substance.

**Scheme 8:**
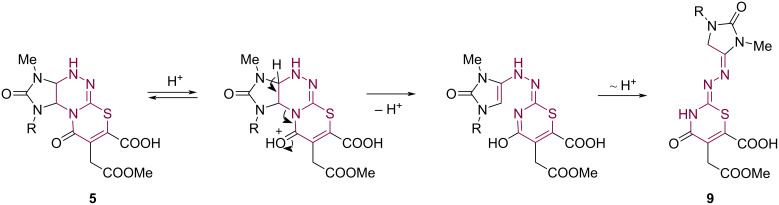
Plausible path for the formation of products **9**.

We assumed the following mechanism for the formation of products **9** (Scheme 8). Redistribution of the electron density in the acid molecule **5** after protonation of the carbonyl group in the thiazine ring leads to the cleavage of the triazine C–N bond. Further proton transfer gives product **9**.

The structures of the synthesized compounds **3a**,**b**,**j** and **5a**–**k**,**m** were confirmed by IR, ^1^H and ^13^C NMR spectroscopy, and high-resolution mass spectrometry. the potassium salts **3c**–**i**,**k**,**m** were characterized by their ^1^H and ^13^C NMR spectra.

**Figure 3:**
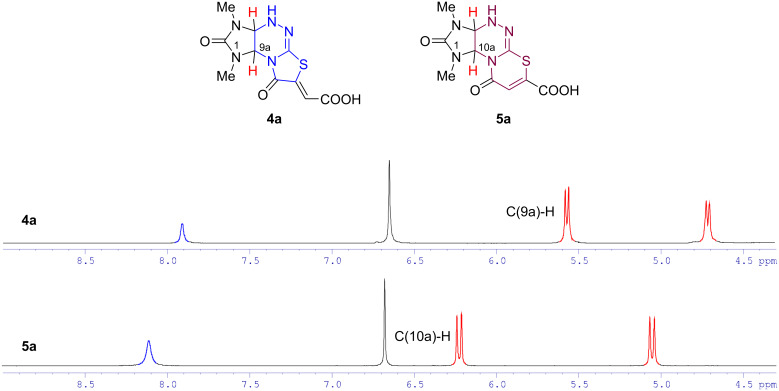
^1^H NMR spectra of compounds **4a** and **5a** in DMSO-*d*_6_ in the region of 4.3–9.0 ppm.

In the ^1^H NMR spectra, the doublets of the bridging hydrogen atom C(9a)H in compound **4** and C(10a)H in compound **5** are characteristic signals which allow to attribute the synthesized compounds to one of the two heterocyclic systems, i.e., imidazo[4,5-*e*]thiazolo[2,3-*c*][1,2,4]triazine and imidazo[4,5-*e*][1,3]thiazino[2,3-*c*][1,2,4]triazine. Thus, the signals of the corresponding protons for isomeric acids **4a** and **5a** appeared at δ 5.59 and 6.23 ppm, respectively, that is obviously due to a deshielding effect of the carbonyl group of the products **4a** and **5a** as well as its closer location in structures **5** (Figure 3).

**Figure 4:**
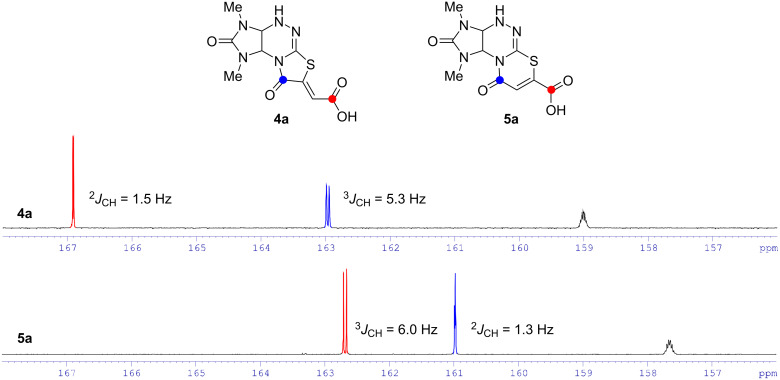
^13^C NMR GATED spectra of compounds **4a** and **5a** in DMSO-*d*_6_ in the region of 156.0–168.0 ppm.

In the downfield region of the ^13^C NMR spectra registered without proton decoupling for isomeric acids **4a** and **5a**, the carbon atom doublets of the carboxyl groups, carbonyl groups of thiazole (for **4a**) or thiazine (for **5a**) cycles, as well as multiplets of carbonyl groups of the urea fragment are observed (Figure 4). Values of spin–spin interaction constants ^3^*J*_CH_ equal to 5.3–6.0 Hz indicate the *cis*-orientation of the vinyl proton and the carbonyl (for **4a**, blue) or the carboxyl group (for **5a**, red) relative to the double bond [24–26]. The values of spin–spin interaction constants of other doublets (^2^*J*_CH_ = 1.3–1.5 Hz) indicate the position of the carboxyl (for **4a**, red) or carbonyl (for **5a**, blue) groups through two bonds relative to the olefinic proton.

**Figure 5:**
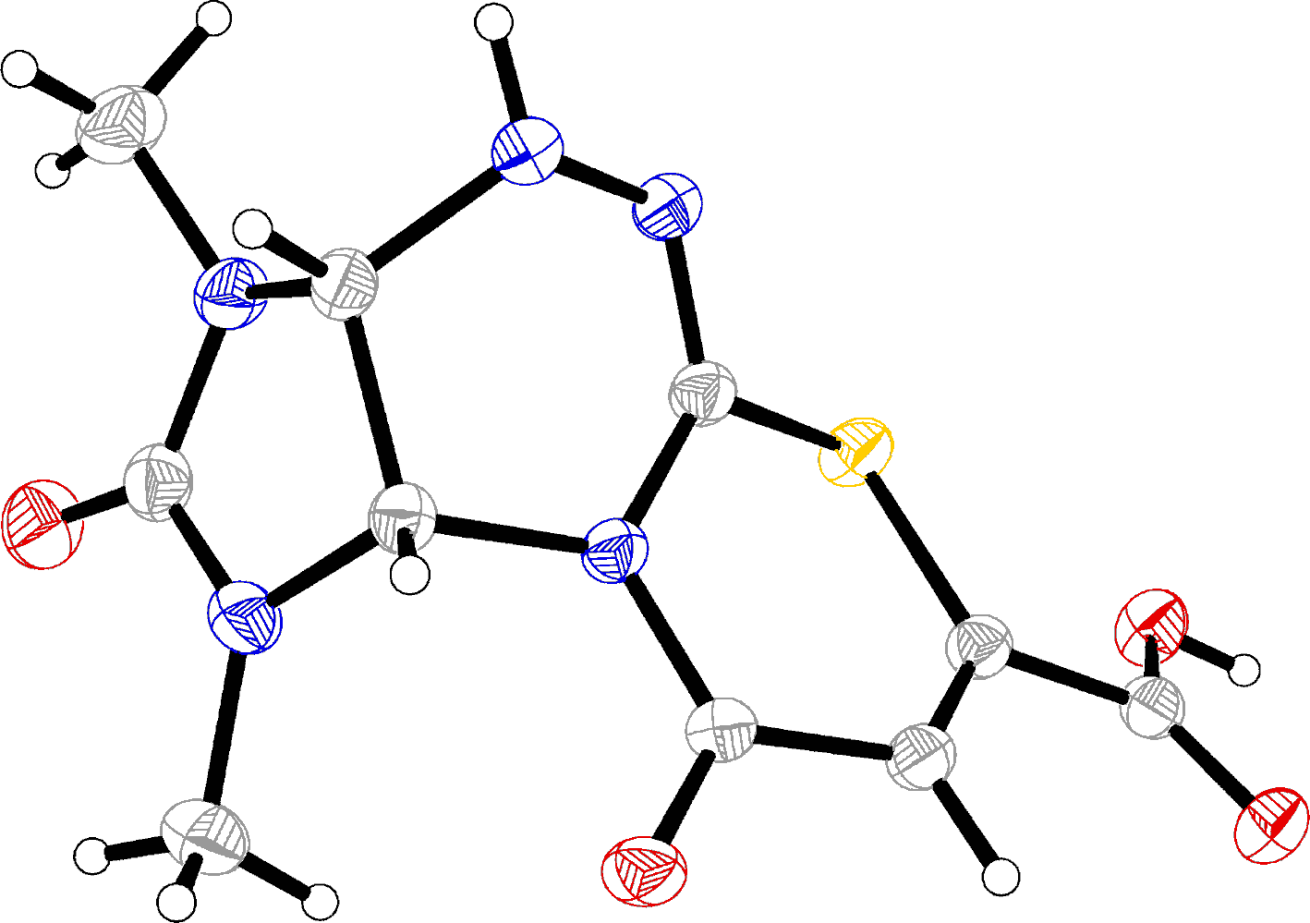
General view of **5a** in the crystal in thermal ellipsoid representation (*p* = 80%).

The structure of compound **5a** was additionally confirmed by X-ray structural analysis data (Figure 5).

## Conclusion

In summary, routes for the selective formation of various derivatives of the new heterocyclic system, imidazo[4,5-*e*][1,3]thiazino[2,3-*c*][1,2,4]triazine, were found during the cascade processes of alkaline hydrolysis of the ester group in functionalized derivatives of imidazo[4,5-*e*]thiazolo[3,2-*b*][1,2,4]triazine or imidazo[4,5-*e*]thiazolo[2,3-*c*][1,2,4]triazine and the expansion of the thiazolidine ring to a thiazine core. The methodology proved to be effective for the preparation of a series of target compounds with different substituents in the tricyclic fragment.

## Supporting Information

File 1 Experimental and analytical data.

File 2 Crystallographic information file for compound **5a**.
