# Supplementary material for: Synthesis of imidazo[4,5-e][1,3]thiazino[2,3-c][1,2,4]triazines via a base-induced rearrangement of functionalized imidazo[4,5-e]thiazolo[2,3-c][1,2,4]triazines
Source: Beilstein J Org Chem. 2023 Jul 28;19:1047–54. doi: 10.3762/bjoc.19.80 (PMC10390826; doi:10.3762/bjoc.19.80)
Supplement: File 1 — Experimental and analytical data. [file Beilstein_J_Org_Chem-19-1047-s001.pdf]

BEILSTEIN JOURNAL OF ORGANIC CHEMISTRY

Supporting Information

for

Synthesis of imidazo[4,5-*e*][1,3]thiazino[2,3-*c*][1,2,4]triazines via a base-induced rearrangement of functionalized imidazo[4,5-*e*]thiazolo[2,3-*c*][1,2,4]triazines

Dmitry B. Vinogradov, Alexei N. Izmest'ev, Angelina N. Kravchenko, Yuri A. Strelenko and Galina A. Gazieva

Beilstein J. Org. Chem. **2023,** *19,* 1047–1054. doi:10.3762/bjoc.19.80

Experimental and analytical data

License and Terms: This is a supporting information file under the terms of the Creative Commons Attribution License (https://creativecommons.org/licenses/by/4.0). Please note that the reuse, redistribution and reproduction in particular requires that the author(s) and source are credited and that individual graphics may be subject to special legal provisions.
The license is subject to the *Beilstein Journal of Organic Chemistry* terms and conditions: (https://www.beilstein-journals.org/bjoc/terms)

Table of contents

1. Experimental ... S2
2. Synthesis of compounds **1c**,**f**,**i** ... S2
3. Synthesis of compounds **2c**,**i**,**j** ... S3
4. General procedure for the synthesis of potassium imidazo[4,5-*e*][1,3]thiazino[2,3-*c*][1,2,4]triazine-7-carboxylates **3a–m** ... S4
5. General procedure for the synthesis of (imidazo[4,5-*e*]thiazolo[2,3-*c*][1,2,4]triazin-6-ylidene)acetic acid **4a**,**b** ... S7
6. General procedure for the synthesis of imidazo[4,5-*e*][1,3]thiazino[2,3-*c*][1,2,4]triazine-7-carboxylic acid **5a–k**,**m** ... S8
7. Procedure for the synthesis imidazo[4,5-*e*][1,2,4]triazines **7i**,**j** ... S11
8. Procedure for the synthesis of compounds **9k**,**l** ... S12
9. Procedure for the synthesis of 1-(4-chlorophenyl)-3-methylthiourea **10** ... S13
10. Procedure for the synthesis of 1-(4-chlorophenyl)-4,5-dihydroxy-3-methylimidazolidine-2-thione **11j** ... S13
11. Crystallographic data ... S13
12. References ... S14
13. ^{1}H NMR monitoring of the rearrangement of **1d** into **3d** ... S15
14. Spectra ^{1}H and ^{13}C NMR of **1c**,**f**,**i** and **2c**,**i**,**j** ... S16
15. Spectra ^{1}H and ^{13}C NMR of **3a–m**, ^{1}H–^{13}C HMBC NMR of **3k** ... S28
16. Spectra ^{1}H and ^{13}C NMR of **4a**,**b,** ^{1}H–^{13}C HMBC NMR of **4a** ... S55
17. Spectra ^{1}H, ^{13}C NMR of **5a–k**,**m**, ^{1}H–^{13}C HSQC of **5k** and ^{1}H–^{13}C HMBC of **5a**,**k** ... S60
18. Spectra ^{1}H and ^{13}C NMR of **7i**,**j** ... S87
19. Spectra ^{1}H, ^{13}C, ^{1}H–^{13}C HMBC NMR of **9k**,**l** ... S91
20. Spectra ^{1}H and ^{13}C NMR of **10** ... S97
21. Spectra ^{13}C NMR GATED of **4a** and **5a** ... S99

Experimental

Melting points were determined in open glass capillaries on a Gallenkamp (Sanyo) melting point apparatus. The ^1H and ^{13}C NMR spectra were recorded on Bruker AM300 (300.13 MHz), Bruker DRX500 (500.13 MHz) and Bruker AV600 (600.13 MHz) spectrometers using DMSO-d_6 as solvent. Chemical shifts (δ) are given in ppm from TMS as an internal standard. IR spectra were recorded on a Bruker ALPHA instrument in KBr pellets. High resolution mass spectra (HRMS) were measured on a Bruker micrOTOF II instrument using electrospray ionization (ESI). The measurements were done in a positive ion mode (interface capillary voltage−4500 V) or in a negative ion mode (3200 V); mass range from *m*/*z* 50 to *m*/*z* 3000 Da; external or internal calibration was done with electrospray calibrant solution (Fluka). A syringe injection was used for solutions in acetonitrile or methanol (flow rate 3 mL min^{-1}). Nitrogen was applied as a dry gas; the interface temperature was set at 180 °C. All the reagents were purchased from Acros Organics and used without further purification.

Compounds **1a**,**b**,**d**,**e**,**g**,**h** and **2a**,**b**,**d**,**e**,**g**,**h**,[1] **7c**,[2] **8a–c**[3] and **11i**[4] were prepared according to known procedures.

Synthesis of compounds 1c,f,i

For **1c**: Diethyl acetylenedicarboxylate (2.1 mmol) was added to a refluxing suspension of 5,7-dipropyl-3-thioxohexahydro-1*H*-imidazo[4,5-*e*][1,2,4]triazin-6(2*H*)-one (**6c**, 2 mmol) in 4 mL of EtOH and the mixture was refluxed for 2 h. Then, triethylamine (0.275 mL, 2 mmol) was added to the suspension, and the mixture was refluxed for additional 4 h. After cooling, the precipitate of compound **1c** was filtered off, washed with MeOH, and dried.

For **1f**: Diethyl acetylenedicarboxylate (2.1 mmol) was added to a refluxing suspension of 5,7-dimethyltetrahydro-1*H*-imidazo[4,5-*e*][1,2,4]triazine-3,6(2*H*,4*H*)-dithione (**6f**, 2 mmol) in 4 mL of MeOH and the mixture was refluxed for 4 h. After cooling, the precipitate of compound **1f** was filtered off, washed with MeOH, and dried.

For **1i**: EtONa as 21% in ethanol (0.187 mL, 0.5 mmol) was added to a refluxing suspension of ester **2i** (1 mmol) in EtOH (10 ml) and the mixture was refluxed for 1 h. After cooling, the precipitate of compound **1i** was filtered off and washed with MeOH.

Ethyl (*Z*)-2-(2,8-dioxo-1,3-dipropyl-1,2,3,3a,4,9a-hexahydroimidazo[4,5-*e*]thiazolo[2,3-*c*][1,2,4]triazin-7(8*H*)-ylidene)acetate (1c). Yield 312 mg (41%); yellow solid; mp: 138–140 °C. IR (KBr): ν 3272 (NH), 3080 (=CH), 2969, 2936, 2875 (Alk), 1690, 1649 (C=O) cm^{-1}. ^1H NMR (600 MHz, DMSO-d_6): δ 0.75–0.84 (m, 6H, $2CH_3$), 1.25 (t, *J* = 7.1 Hz, 3H, CH_3), 1.35–1.50 (m, 3H, CH_2), 1.53–1.67 (m, 1H, CH_2), 2.90–3.03 (m, 1H, NCH_2), 3.09–3.13 (m, 1H, NCH_2), 3.16–3.23 (m, 1H, NCH_2), 4.23 (q, *J* = 7.1 Hz, 2H, OCH_2), 4.87 (dd, *J* = 5.8, 2.2 Hz, 1H, 3a-H), 5.71 (d, *J* = 5.9 Hz, 1H, 9a-H), 6.72 (s, 1H, =CH), 8.04 (d, *J* = 2.2 Hz, 1H, 4-NH). ^{13}C NMR (150 MHz, DMSO-d_6): δ 11.05, 11.10, 14.05 ($3CH_3$), 20.48, 20.74 ($2CH_2$), 41.82, 44.68 ($2NCH_2$), 61.39, 62.21, 64.14 (OCH_2, C-3a, C-9a), 113.59 (=CH), 134.95 (5a-C=N), 141.79 (C-7), 158.28, 162.74 (2-C=O, 8-C=O), 165.71 (COOEt). HRMS (ESI): *m*/*z* [*M* + H]$^+$ calcd for $C_{16}H_{23}N_5O_4S$: 382.1544; found: 382.1545.

(*Z*)-Ethyl 2-(1,3-dimethyl-8-oxo-2-thioxo-1,2,3,3a,4,9a-hexahydroimidazo[4,5-*e*]thiazolo[2,3-*c*][1,2,4]triazin-7(8*H*)-ylidene)acetate (1f). Yield 450 mg (66%); orange solid; mp: 236–238 °C. IR (KBr): ν 3272 (NH), 3080 (=CH), 2969, 2936, 2875 (Alk), 1690, 1649 (C=O) cm^{-1}. ^1H NMR (300 MHz, DMSO-d_6): δ 1.26 (t, *J* = 7.1 Hz, 3H, CH_3), 2.95 (s, 3H, NCH_3), 3.17 (s, 3H, NCH_3), 4.25 (q, *J* = 7.1 Hz, 2H, OCH_2), 5.13 (d, *J* = 6.1 Hz, 1H, 3a-H), 5.88 (d, *J* = 6.2 Hz, 1H, 9a-H), 6.74 (s, 1H, =CH), 8.25 (d, *J* = 1.7 Hz, 1H, NH). ^{13}C NMR (75 MHz, DMSO-d_6): δ 14.06 (CH_3), 31.46, 35.46 ($2NCH_3$), 61.46, 66.90, 67.52 (OCH_2, C-3a, C-9a), 113.93 (=CH), 136.06 (5a-C=N), 141.47 (C-7), 162.51, 165.68 (2-C=O, 8-C=O), 183.79 (COOEt). HRMS (ESI): *m/z* $[M + H]^+$ calcd for $C_{12}H_{16}N_5O_3S_2$: 342.0689; found: 342.0698.

(*Z*)-Ethyl 2-(3-(2-hydroxyethyl)-8-oxo-1-phenyl-2-thioxo-1,2,3,3a,4,9a-hexahydroimidazo[4,5-*e*]thiazolo[2,3-*c*][1,2,4]triazin-7(8*H*)-ylidene)acetate (1i). Yield 165 mg (38%); bright yellow solid; mp: 226–227 °C. IR (KBr): ν 3498, 3291 (NH), 3087, 3061 (=CH), 2956, 2928, 2875 (Alk), 1725, 1669, 1649 (C=O) cm^{-1}. ^1H NMR (300 MHz, DMSO-d_6): δ 1.24 (t, *J* = 7.1 Hz, 3H, CH_3), 3.55-3.69 (m, 3H, CH_2), 3.84-3.89 (m, 3H, CH_2), 4.22 (q, *J* = 6.9 Hz, 2H, OCH_2), 5.46 (d, *J* = 6.2 Hz, 1H, 3a-H), 6.35 (d, *J* = 6.3 Hz, 1H, 9a-H), 6.50 (s, 1H, =CH), 7.23-7.39 (m, 5H, Ph), 8.20 (d, *J* = 0.8 Hz, 1H, NH). ^{13}C NMR (75 MHz, DMSO-d_6): δ 14.03 (CH_3), 46.41 (NCH_2), 58,33, 61.48, 66.00, 67.80 ($2OCH_2$, C-3a, C-9a), 113.99 (=CH), 127.61, 128.53, 128.84 (Ph-2-6), 135.49, 139.09, 141.24 (5a-C=N, C-7m Ph-1), 161.20 (8-C=O), 165.61 (COOEt), 182.42 (2-C=S). HRMS (ESI): *m/z* $[M + H]^+$ calcd for $C_{18}H_{19}N_5O_4S_2$: 434.0951; found: 434.0954.

Synthesis of compounds 2c,i,j

For **2c**: Diethyl acetylenedicarboxylate (2.1 mmol) was added to a suspension of 5,7-dipropyl-3-thioxohexahydro-1*H*-imidazo[4,5-*e*][1,2,4]triazin-6(2*H*)-one (**7c**, 2 mmol) in glacial AcOH (4 mL), and the mixture was stirred at room temperature for 4 h. The reaction mass was poured into 40 mL of distilled water, the precipitate of compound **2c** that formed was filtered off, washed with water, and dried.

For **2i,j**: Diethyl acetylenedicarboxylate (3 mmol for **2i** or 2.2 mmol for **2j**) was added to a refluxing suspension of imidazo[4,5-*e*][1,2,4]triazines **7i,j** (2 mmol) in MeOH (4 mL) and the mixture was refluxed for 6 h. After cooling, the precipitate of compounds **2i,j** were filtered off, washed with MeOH, and dried.

Ethyl (*Z*)-2-(2,7-dioxo-1,3-dipropyl-1,2,3,3a,9,9a-hexahydroimidazo[4,5-*e*]thiazolo[3,2-*b*][1,2,4]triazin-6(7*H*)-ylidene)acetate (2c). Yield 541 mg (71%); pale yellow solid; mp: 132–134 °C. IR (KBr): ν 3231 (NH), 3075 (=CH), 2966, 2934, 2875 (Alk), 1733, 1692, 1651 (C=O, C=N, C=C) cm^{-1}. ^1H NMR (600 MHz, DMSO-d_6): δ 0.71 (t, *J* = 7.4 Hz, 3H, CH_3), 0.87 (t, *J* = 7.4 Hz, 3H, CH_3), 1.25 (t, *J* = 7.1 Hz, 3H, CH_3), 1.34–1.45 (m, 2H, CH_2), 1.46–1.63 (m, 2H, CH_2), 2.94–2.98 (m, 1H, NCH_2), 3.00–3.11 (m, 2H, NCH_2), 3.18–3.28 (m, 1H, NCH_2), 4.24 (q, *J* = 7.1 Hz, 2H, OCH_2), 4.88 (dd, *J* = 6.0, 2.3 Hz, 1H, 9a-H), 4.91 (d, *J* = 6.0 Hz, 1H, 3a-H), 6.82 (s, 1H, =CH), 6.89 (d, *J* = 2.4 Hz, 1H, 9-NH). ^{13}C NMR (150 MHz, DMSO-d_6): δ 10.93, 11.30, 13.99 ($3CH_3$), 20.71, 20.98 ($2CH_2$), 42.26, 42.50 ($2NCH_2$), 61.61, 63.64, 65.01 (OCH_2, C-3a, C-9a), 116.50 (=CH), 139.01 (C-6), 148.75 (4a-C=N),

158.28, 159.18 (2-C=O, 7-C=O), 165.30 (COOEt). HRMS (ESI): *m/z* [*M* + H]$^+$ calcd for $C_{16}H_{23}N_5O_4S$: 382.1544; found: 382.1541.

(*Z*)-Ethyl 2-(1-(2-hydroxyethyl)-7-oxo-3-phenyl-2-thioxo-1,2,3,3a,9,9a-hexahydroimidazo[4,5-*e*]thiazolo[3,2-*b*][1,2,4]triazin-6(7*H*)-ylidene)acetate (2i). Yield 485 mg (56%); pale yellow solid; mp: 131–133 °C. ^{1}H NMR (300 MHz, DMSO-d_6): δ 1.27 (t, *J* = 7.1 Hz, 3H, CH_3), 3.50-3.56 (m, 1H, CH_2), 3.61-3.67 (m, 3H, NCH_2), 4.26 (q, *J* = 7.0 Hz, 2H, OCH_2), 5.51-5.57 (m, 2H, 3a-H, 9a-H), 6.88 (s, 1H, =CH), 7.21 (s, 1H, NH), 7.30 (t, *J* = 7.3 Hz, 1H, Ph-4), 7.43 (t, *J* = 7.7 Hz, 2H, Ph-3,5), 7.57 (d, *J* = 7.7 Hz, 2H, Ph-3,5). ^{13}C NMR (75 MHz, DMSO-d_6): δ 14.04 (CH_3), 45.53 (NCH_2), 57.94, 61.76, 68.00, 68.31 (2OCH_2, C-3a, C-9a), 116.85 (=CH), 126.67, 127.48, 128.42 (Ph-2-6), 138.72, 138.79 (Ph-1, C-6), 150.55 (4a-C=N), 159.30 (7-C=O), 165.40 (COOEt), 180.61 (2-C=S). HRMS (ESI): *m/z* [*M* + H]$^+$ calcd for $C_{18}H_{19}N_5O_4S_2$: 434.0951; found: 434.0938.

(*Z*)-Ethyl 2-(3-(4-chlorophenyl)-1-methyl-7-oxo-2-thioxo-1,2,3,3a,9,9a-hexahydroimidazo[4,5-*e*]thiazolo[3,2-*b*][1,2,4]triazin-6(7*H*)-ylidene)acetate (2j). Yield 428 mg (49%); pale yellow solid; mp: 233–235 °C. IR (KBr): ν 3387, 3225 (NH), 3087 (=CH), 2978, 2935, 2913 (Alk), 1741, 1695, 1651, 1616 (C=O, C=N, C=C) cm^{-1}. ^{1}H NMR (300 MHz, DMSO-d_6): δ 1.27 (t, *J* = 7.1 Hz, 3H, CH_3), 3.02 (s, 3H, CH_3), 4.26 (q, *J* = 7.1 Hz, 2H, OCH_2), 5.34 (d, *J* = 6.6 Hz, 1H, 9a-H), 5.58 (d, *J* = 6.6 Hz, 1H, 3a-H), 6.87 (s, 1H, =CH), 7.25 (s, 1H, NH), 7.49 (d, *J* = 8.6 Hz, 2H, Ar), 7.63 (d, *J* = 8.7 Hz, 2H, Ar). ^{13}C NMR (75 MHz, DMSO-d_6): δ 14.01 (CH_3), 30.83 (NCH_3), 61.73 (OCH_2), 68.03, 68.21 (C-3a, C-9a), 116.86 (=CH), 128.38, 128.82, 130.80 (Ph-2-6), 137.72, 138.76 (Ph-1, C-6), 150.69 (4a-C=N), 159.24 (7-C=O), 165.35 (COOEt), 180.78 (2-C=S). HRMS (ESI): *m/z* [*M* + H]$^+$ calcd for $C_{17}H_{16}N_5O_3S_2$: 438.0456; found: 438.0446.

General procedure for the synthesis of potassium imidazo[4,5-*e*][1,3]thiazino[2,3-*c*][1,2,4]triazine-7-carboxylates 3a–m

Method A (for **3a–i**): To a stirring suspension of imidazo[4,5-*e*]thiazolo[2,3-*c*][1,2,4]triazines **1a–i** (1 mmol) in MeOH (3.5 mL for **3a**,**b** or 7 mL for **3c–i**), 0.250 mL KOH (2.5 mmol, 40% aq. solution) was added and the mixture was stirred at room temperature for 4 h. The precipitate of compounds **3a**,**b**,**j** was filtered off, washed with MeOH, and dried. For compounds **3c–i** the reaction mixtures were evaporated at reduced pressure. Compounds **3c–i** were used without isolation.

Method B (for **3a–d**,**j**): To a stirring suspension of imidazo[4,5-*e*]thiazolo[3,2-*b*][1,2,4]triazines **2a–d**,**j** (1 mmol) in MeOH (3.5 mL for **3a**,**b** or 7 mL for **3c**,**d**,**j**), 0.250 mL KOH (2.5 mmol, 40% aq. solution) was added and the mixture was stirred at room temperature for 4 h. The precipitate of compounds **3a**,**b**,**j** was filtered off, washed with MeOH, and dried. For compounds **3c**,**d** the reaction mixtures were evaporated at reduced pressure. Compounds **3c**,**d** were used without isolation.

Method C (for **3a**): Diethyl acetylenedicarboxylate (1.05 mmol) was added to a refluxing suspension of 3-thioxoimidazo[4,5-*e*][1,2,4]triazine (**7a**, 1 mmol) in 10 mL of MeOH and the mixture was refluxed for 2 h. The reaction mixture was cooled, 0.250 mL KOH (2.5 mmol, 40% aq. solution) was added and stirred at room temperature for 4 h.

The reaction mixture was evaporated at reduced pressure. The residue in the flask was triturated with MeCN (3 mL), the precipitate of compound **3a** was filtered off, washed with MeCN, and dried.

Method D (for **3k–m**): To a stirring suspension of imidazo[4,5-*e*]thiazolo[2,3-*c*][1,2,4]triazines **8a–c** (1 mmol) in MeOH (10 mL), KOH (40% aq. solution, 0.250 mL (2.5 mmol) for **3k** or 0.350 mL (3.5 mmol) for **3l,m**) was added and the mixture was stirred at room temperature (1 h for **3k** or 24 h for **3l,m**). The reaction mixtures were evaporated at reduced pressure. Compounds **3k–m** were used without isolation.

Potassium 1,3-dimethyl-2,9-dioxo-1,2,3,3a,4,10a-hexahydro-9*H*-imidazo[4,5-*e*][1,3]thiazino[2,3-*c*][1,2,4]triazine-7-carboxylate (3a). Method A: yield 241 mg (81%); method B: yield 199 mg (67%); method C: yield 205 mg (69%); pale yellow solid; mp: 273–275 °C. IR (KBr): ν 3284, 3258 (NH), 3061 (=CH), 2986, 2906, 2869 (Alk), 1722, 1688, 1632 (C=O, C=N, C=C) cm^{-1}. ^{1}H NMR (300 MHz, DMSO-d_6): δ 2.58 (s, 6H, $2CH_3$), 5.02 (d, *J* = 8.4 Hz, 1H, 3a-H), 6.20 (d, *J* = 8.4 Hz, 1H, 10a-H), 6.34 (s, 1H, =CH), 7.79 (s, 1H, 4-NH). ^{13}C NMR (75 MHz, DMSO-d_6): δ 27.35, 28.37 ($2CH_3$), 66.64, 69.99 (C-3a, C-10a), 113.30 (=CH), 139.66 (C-7), 154.94, 157.67, 161.53, 162.80 (5a-C=N, 2-C=O, 9-C=O, COOK). HRMS (ESI): *m/z* [*M* + H]$^+$ calcd for $C_{10}H_{11}N_5O_4S$: 298.0605; found: 298.0607.

Potassium 1,3-diethyl-2,9-dioxo-1,2,3,3a,4,10a-hexahydro-9*H*-imidazo[4,5-*e*][1,3]thiazino[2,3-*c*][1,2,4]triazine-7-carboxylate (3b). Method A: yield 205 mg (63%); method B: yield 211 mg (65%); yellow solid; mp: 260–262 °C. IR (KBr): ν 3281 (NH), 3073, 3051 (=CH), 2988, 2952, 2940, 2875 (Alk), 1710, 1664, 1629, 1609 (C=O, C=N, C=C) cm^{-1}. ^{1}H NMR (300 MHz, DMSO-d_6): δ 0.95 (t, *J* = 7.1 Hz, 6H, $2CH_3$), 2.81–2.93 (m, 1H, NCH_2), 2.96–3.07 (m, 1H, NCH_2), 3.11–3.31 (m, 2H, NCH_2), 5.18 (d, *J* = 8.7 Hz, 1H, 3a-H), 6.28 (d, *J* = 8.7 Hz, 1H, 10a-H), 6.33 (s, 1H, =CH), 7.76 (s, 1H, 4-NH). ^{13}C NMR (75 MHz, DMSO-d_6): δ 12.63, 13.93 ($2CH_3$), 34.60, 36.00 ($2NCH_2$), 65.56, 68.47 (C-3a, C-10a), 113.74 (=CH), 140.40 (C-7), 155.89, 157.02, 161.79, 163.37 (5a-C=N, 2-C=O, 9-C=O, COOK). HRMS (ESI): *m/z* [*M* + H]$^+$ calcd for $C_{12}H_{15}N_5O_4S$: 326.0918; found: 326.0912.

Potassium 2,9-dioxo-1,3-dipropyl-1,2,3,3a,4,10a-hexahydro-9*H*-imidazo[4,5-*e*][1,3]thiazino[2,3-*c*][1,2,4]triazine-7-carboxylate (3c). ^{1}H NMR (300 MHz, DMSO-d_6): δ 0.71–0.86 (m, 6H, $2CH_3$), 1.29–1.53 (m, 4H, CH_2), 2.70–2.82 (m, 1H, NCH_2), 2.92–3.11 (m, 2H, NCH_2), 3.23–3.30 (m, 1H, NCH_2), 5.20 (d, *J* = 8.7 Hz, 1H, 3a-H), 6.28 (d, *J* = 8.7 Hz, 1H, 10a-H), 6.35 (s, 1H, =CH), 7.78 (s, 1H, 4-NH). ^{13}C NMR (75 MHz, DMSO-d_6): δ 10.96 ($2CH_3$), 19.97, 20.80 ($2CH_2$), 41.15, 42.26 ($2NCH_2$), 65.11, 68.50 (C-3a, C-10a), 113.11 (=CH), 139.91 (C-7), 155.51, 157.11, 161.12, 162.87 (5a-C=N, 2-C=O, 9-C=O, COOK).

Potassium 3-methyl-2,9-dioxo-1-phenyl-1,2,3,3a,4,10a-hexahydro-9*H*-imidazo[4,5-*e*][1,3]thiazino[2,3-*c*][1,2,4]triazine-7-carboxylate (3d). ^{1}H NMR (300 MHz, DMSO-d_6): δ 2.71 (s, 3H, NCH_3), 5.30 (d, *J* = 8.8 Hz, 1H, 3a-H), 6.04 (s, 1H, =CH), 6.88 (d, *J* = 8.8 Hz, 1H, 10a-H), 7.12–7.27 (m, 3H, Ph-2,4,6), 7.30 (t, *J* = 7.6 Hz, 2H, Ph-3,5), 7.95 (s, 1H, 4-NH). ^{13}C NMR (150 MHz, DMSO-d_6): δ 27.24 (NCH_3), 65.84, 70.63 (C-3a, C-10a), 113.20 (=CH), 126.00 (Ph-4), 126.48, 128.38 (Ph-2,6, Ph-3,5), 136.40, 140.55 (Ph-1, C-7), 154.92, 155.78, 161.22, 161.72 (5a-C=N, 2-C=O, 9-C=O, COOK).

Potassium 3-ethyl-2,9-dioxo-1-phenyl-1,2,3,3a,4,10a-hexahydro-9*H*-imidazo[4,5-*e*][1,3]thiazino[2,3-*c*][1,2,4]triazine-7-carboxylate (3e). ^{1}H NMR (300 MHz, DMSO-d_6): δ 1.05 (t, *J* = 7.1 Hz, 3H, CH_3), 3.04–3.16 (m, 1H, NCH_2), 3.19–3.23 (m, 1H, NCH_2), 5.45 (d, *J* = 8.8 Hz, 1H, 3a-H), 6.05 (s, 1H, =CH), 6.88 (d, *J* = 8.8 Hz, 1H, 10a-H), 7.12–7.22 (m, 3H, Ph-2,6, Ph-4), 7.30 (t, *J* = 7.6 Hz, 2H, Ph-3,5), 7.91 (s, 1H, 4-NH). ^{13}C NMR (75 MHz, DMSO-d_6): δ 12.16 (CH_3), 34.16 (CH_2), 65.81, 68.21 (C-3a, C-10a), 113.11 (=CH), 125.80 (Ph-4), 126.33, 128.23 (Ph-2,6, Ph-3,5), 136.38, 140.51 (Ph-1, C-7), 154.90, 155.17, 161.15, 161.67 (5a-C=N, 2-C=O, 9-C=O, COOK).

Potassium 1,3-dimethyl-9-oxo-2-thioxo-1,2,3,3a,4,10a-hexahydro-9*H*-imidazo[4,5-*e*][1,3]thiazino[2,3-*c*][1,2,4]triazine-7-carboxylate (3f). ^{1}H NMR (300 MHz, DMSO-d_6): δ 2.90 (s, 6H, $2NCH_3$), 5.39 (d, *J* = 9.0 Hz, 1H, 3a-H), 6.39 (s, 1H, =CH), 6.49 (d, *J* = 9.0 Hz, 1H, 10a-H), 8.07 (s, 1H, 4-NH). ^{13}C NMR (75 MHz, DMSO-d_6): δ 31.32 (NCH_3), 32.72 (NCH_3), 68.84 (C-10a), 73.76 (C-3a), 113.70 (C=H), 141.11 (C-7), 155.68 (5a-C=N), 161.79, 162.80 (9-C=O, COOK), 182.01 (2-C=S).

Potassium 3-methyl-9-oxo-1-phenyl-2-thioxo-1,2,3,3a,4,10a-hexahydro-9*H*-imidazo[4,5-*e*][1,3]thiazino[2,3-*c*][1,2,4]triazine-7-carboxylate (3g). ^{1}H NMR (300 MHz, DMSO-d_6): δ 3.04 (s, 3H, NCH_3), 5.65 (d, *J* = 9.5 Hz, 1H, 3a-H), 6.04 (s, 1H, =CH), 6.93 (d, *J* = 9.4 Hz, 1H, 10a-H), 7.16 (d, *J* = 7.0 Hz, 2H, Ph-2,6), 7.21–7.41 (m, 3H, Ph-3,5, Ph-4), 8.17 (s, 1H, 4-NH). ^{13}C NMR (75 MHz, DMSO-d_6): δ 30.83 (CH_3), 68.60, 74.38 (C-3a, C-10a), 112.96 (=CH), 127.54 (Ph-4), 128.50, 128.99 (Ph-2,6, Ph-3,5), 137.64, 141.34 (Ph-1, C-7), 154.85, 161.09 (5a-C=N, 9-C=O, COOK), 180.61 (2-C=S).

Potassium 3-ethyl-9-oxo-1-phenyl-2-thioxo-1,2,3,3a,4,10a-hexahydro-9*H*-imidazo[4,5-*e*][1,3]thiazino[2,3-*c*][1,2,4]triazine-7-carboxylate (3h). ^{1}H NMR (300 MHz, DMSO-d_6): δ 1.11 (t, *J* = 7.2 Hz, 3H, CH_3), 3.40–3.52 (m, 3H, NCH_2), 3.71–3.79 (m, 1H, NCH_2), 5.74 (dd, *J* = 9.5, 0.8 Hz, 1H, 3a-H), 6.00 (s, 1H, =CH), 6.89 (d, *J* = 9.5 Hz, 1H, 10a-H), 7.11–7.18 (m, 2H, Ph-2,6), 7.24–7.38 (m, 3H, Ph-3-5), 8.16 (s, 1H, 4-NH). ^{13}C NMR (75 MHz, DMSO-d_6): δ 11.32 (CH_3), 37.67 (CH_2), 68.59, 72.36 (C-3a, C-10a), 112.89 (=CH), 127.47 (Ph-4), 128.43, 129.05 (Ph-2,6, Ph-3,5), 137.55, 141.46 (Ph-1, C-7), 155.00, 161.07 (5a-C=N, 9-C=O, COOK), 179.90 (2-C=S).

Potassium 3-(2-hydroxyethyl)-9-oxo-1-phenyl-2-thioxo-2,3,3a,4,9,10a-hexahydro-1*H*-imidazo[4,5-*e*][1,3]thiazino[2,3-*c*][1,2,4]triazine-7-carboxylate (3i). ^{1}H NMR (300 MHz, DMSO-d_6): δ 3.53–3.67 (m, 4H, $2CH_2$), 5.80 (d, *J* = 9.5 Hz, 1H, 3a-H), 6.02 (s, 1H, =CH), 6.94 (d, *J* = 9.6 Hz, 1H, 10a-H), 7.15 (d, *J* = 7.0 Hz, 2H, Ph-2,6), 7.26–7.35 (m, 3H, Ph-3-5), 8.13 (s, 1H, 4-NH). ^{13}C NMR (75 MHz, DMSO-d_6): δ 46.05 (NCH_2), 58.69, 68.92, 74.48 (OCH_2, C-3a, C-10a), 113.66 (=CH), 128.15 (Ph-4), 129.04, 129.54 (Ph-2,3,5,6), 137.91, 141.57 (Ph-1, C-7), 154.96, 161.56, 161.76 (5a-C=N, 9-C=O, COOK), 180.80 (2-C=S).

Potassium 1-(4-chlorophenyl)-3-methyl-9-oxo-2-thioxo-2,3,3a,4,9,10a-hexahydro-1*H*-imidazo[4,5-*e*][1,3]thiazino[2,3-*c*][1,2,4]triazine-7-carboxylate (3j). Yield 197 mg (44%); pale yellow solid; mp: 242–244 °C. IR (KBr): ν 3445, 3287 (NH), 3088 (=CH), 2928 (Alk), 1661, 1610 (C=O, C=N, C=C) cm^{-1}. ^{1}H NMR (300 MHz, DMSO-d_6): δ 3.02 (s, 3H, NCH_3), 5.63 (d, *J* = 9.4 Hz, 1H, 9a-H), 6.06 (s, 1H, =CH), 6.90 (d, *J* = 9.3 Hz, 1H, 3a-H), 7.18 (d, *J* = 8.6 Hz, 2H, Ar), 7.41 (d, *J* = 8.6 Hz, 2H, Ar), 8.18 (s, 1H, NH). ^{13}C NMR (75

MHz, DMSO-d_6): δ 31.37 (NCH_3), 69.07, 74.68 (C-3a, C-9a), 113.63 (=CH), 129.12, 131.32, 132.55 (Ar-2-4), 137.16, 141.62 (Ar-1, C-7), 155.12, 161.73 (5a-C=N, 9-C=O, COOK), 180.94 (2-C=S). HRMS (ESI): *m/z* [*M* + H]$^+$ calcd for $C_{15}H_{12}ClN_5O_3S_2$: 410.0143; found: 410.0151.

Potassium 8-(2-methoxy-2-oxoethyl)-1,3-dimethyl-2,9-dioxo-1,2,3,3a,4,10a-hexa-hydro-9*H*-imidazo[4,5-*e*][1,3]thiazino[2,3-*c*][1,2,4]triazine-7-carboxylate (3k). ^{1}H NMR (300 MHz, DMSO-d_6): δ 2.58 (s, 6H, $2NCH_3$), 3.54 (s, 3H, OCH_3), 4.08 (s, 2H, =C-CH_2), 5.02 (d, *J* = 8.4 Hz, 1H, 3a-H), 6.16 (d, *J* = 8.4 Hz, 1H, 10a-H), 7.78 (s, 1H, 4-NH). ^{13}C NMR (75 MHz, DMSO-d_6): δ 27.87 (NCH_3), 28.88 (NCH_3), 32.60 (=C-CH_2), 51.72 (OCH_3), 68.03 (C-10a), 70.33 (C-3a), 117.90 (C-8), 139.70 (C-7), 150.94 (5a-C=N), 158.20, 164.08, 172.03 (2-C=O, 9-C=O, COOK, COOMe).

Potassium 8-(2-methoxy-2-oxoethyl)-3-methyl-2,9-dioxo-1-phenyl-1,2,3,3a,4,10a-hexahydro-9*H*-imidazo[4,5-*e*][1,3]thiazino[2,3-*c*][1,2,4]triazine-7-carboxylate (3l). ^{1}H NMR (300 MHz, DMSO-d_6): δ 2.69 (s, 3H, NCH_3), 3.42 (s, 3H, OCH_3), 3.48 (d, *J* = 16.6 Hz, 1H, =C-CH_2), 4.22 (d, *J* = 16.5 Hz, 1H, =C-CH_2), 5.29 (d, *J* = 8.7 Hz, 1H, 3a-H), 6.81 (d, *J* = 8.7 Hz, 1H, 10a-H), 7.10-7.23 (m, 3H, Ph-3-5), 7.27 (d, *J* = 7.8 Hz, 2H, Ph-2,6), 7.92 (s, 1H, 4-NH). ^{13}C NMR (75 MHz, DMSO-d_6): δ 27.69 (NCH_3), 32.37 (=C-CH_2), 51.65 (OCH_3), 67.26, 70.85 (C-3a, C-10a), 117.96 (C-8), 126.29 (Ph-4), 126.81, 128.74 (Ph-2,6, Ph-3,5), 137.03, 140.42 (C-7, Ph-1), 150.68 (5a-C=N), 156.34, 163.13, 171.67 (2-C=O, 9-C=O, COOK, COOMe).

Potassium 8-(2-methoxy-2-oxoethyl)-3-methyl-9-oxo-1-phenyl-2-thioxo-1,2,3,3a,4,10a-hexahydro-9*H*-imidazo[4,5-*e*][1,3]thiazino[2,3-*c*][1,2,4]triazine-7-carboxylate (3m). ^{1}H NMR (300 MHz, DMSO-d_6): δ 3.02 (s, 3H, NCH_3), 3.32 (d, *J* = 16.5 Hz, 1H, =C-CH_2), 3.42 (s, 3H, OCH_3), 4.26 (d, *J* = 16.5 Hz, 1H, =C-CH_2), 5.63 (d, *J* = 9.4 Hz, 1H, 3a-H), 6.83 (d, *J* = 9.4 Hz, 1H, 10a-H), 7.14 (d, *J* = 6.7 Hz, 2H, Ph-2,6), 7.23-7.32 (m, 3H, Ph-3-5), 8.15 (s, 1H, 4-NH). ^{13}C NMR (75 MHz, DMSO-d_6): δ 31.34 (NCH_3), 32.24 (=C-CH_2), 51.68 (OCH_3), 70.10, 74.59 (C-3a, C-10a), 118.06 (C-8), 127.99 (Ph-4), 129.01, 129.52 (Ph-2,6, Ph-3,5), 138.27, 141.35 (C-7, Ph-1), 150.50 (5a-C=N), 162.58, 163.03, 171.67 (9-C=O, COOK, COOMe), 181.20 (2-C=S).

General procedure for the synthesis of (imidazo[4,5-*e*]thiazolo[2,3-*c*][1,2,4]triazin-6-ylidene)acetic acids 4a,b

To a stirring suspension of imidazo[4,5-*e*]thiazolo[2,3-*c*][1,2,4]triazines **1a**,**b** (1 mmol) in 10 mL of H_2O, 0.150 mL KOH (1.5 mmol, 40% aq. solution) was added. The mixture was stirred at reflux for 30 min. After that, 0.150 mL of conc. HCl was added. The first precipitates of acids **4a**,**b** were filtered off, washed with water, and dried. After standing, precipitates of compounds **5a**,**b** were filtered off, washed with water, and dried.

(*Z*)-2-(1,3-Dimethyl-2,8-dioxo-1,2,3,3a,4,9a-hexahydroimidazo[4,5-*e*]thia-zolo[2,3-*c*][1,2,4]triazin-7(8*H*)-ylidene)acetic acid (4a). Yield 50 mg (17%); yellow solid; mp: 237–238 °C. IR (KBr): ν 3339 (NH), 3044 (=CH), 2940 (Alk), 2672, 2558 (COOH), 1707, 1678, 1649 (C=O, C=N, C=C) cm^{-1}. ^{1}H NMR (600 MHz, DMSO-d_6): δ 2.62 (s, 3H, NCH_3), 2.85 (s, 3H, NCH_3), 4.78 (d, *J* = 6.1 Hz, 1H, 3a-H), 5.62 (d, *J* = 6.0 Hz, 1H, 9a-H), 6.68 (s, 1H, =CH), 7.96 (s, 1H, 4-NH). ^{13}C NMR (150 MHz, DMSO-d_6): δ 27.88 (3-NCH_3), 31.29 (1-NCH_3), 63.93 (C-3a), 65.98 (C-9a), 114.65 (=CH), 135.77 (5a-C=N), 141.35 (C-7),

158.98 (2-C=O), 162.96 (8-C=O), 166.85 (COOH). HRMS (ESI): *m/z* [*M* + H]$^+$ calcd for $C_{10}H_{11}N_5O_4S$: 298.0605; found: 298.0613.

(*Z*)-2-(1,3-Diethyl-2,8-dioxo-1,2,3,3a,4,9a-hexahydroimidazo[4,5-*e*]thiazolo[2,3-*c*][1,2,4]triazin-7(8*H*)-ylidene)acetic acid (4b). Yield 123 mg (38%); yellow solid; mp: 275–276 °C. IR (KBr): ν 3288 (NH), 3105 (COOH), 3069 (=CH), 2997, 2947 (Alk), 2612 (COOH), 1716, 1690, 1647 (C=O, C=N, C=C) cm^{-1}. ^{1}H NMR (300 MHz, DMSO-*d*$_6$): δ 1.01 (t, *J* = 7.1 Hz, 3H, CH_3), 1.07 (t, *J* = 7.0 Hz, 3H, CH_3), 3.03–3.31 (m, 3H, NCH_2), 3.38–3.48 (m, 1H, NCH_2), 4.85 (d, *J* = 5.9 Hz, 1H, 3a-H), 5.69 (d, *J* = 5.9 Hz, 1H, 9a-H), 6.70 (s, 1H, =CH), 7.95 (s, 1H, 4-NH). ^{13}C NMR (75 MHz, DMSO-*d*$_6$): δ 12.72, 12.96 (2CH_3), 34.90, 37.79 (2CH_2), 61.53 (C-3a), 63.66 (C-9a), 114.80 (=CH), 135.48 (5a-C=N), 141.25 (C-7), 157.92 (2-C=O), 162.98 (8-C=O), 166.89 (COOH). HRMS (ESI): *m/z* [*M* + H]$^+$ calcd for $C_{12}H_{15}N_5O_4S$: 326.0918; found: 326.0910.

General procedure for the synthesis of imidazo[4,5-*e*][1,3]thiazino[2,3-*c*][1,2,4]triazine-7-carboxylic acids 5a–k,m

For **5a**,**b**,**j**: To a solution of potassium salts **3a**,**b**,**j** (1 mmol) in water (10 mL), conc. HCl was added (to pH 2–3). After standing at room temperature for 2 h, the precipitate of compounds **5a**,**b**,**j** was filtered off, washed with water, and dried.

For **5c–i**: To a stirring suspension of imidazo[4,5-*e*]thiazolo[2,3-*c*][1,2,4]triazines **1c–i** (1 mmol) in MeOH (7 mL), 0.250 mL of KOH (2.5 mmol, 40% aq. solution) was added and the mixture was stirred at room temperature for 4 h. Then, 0.360 mL of conc. HCl was added and the reaction mixture was evaporated under reduced pressure to 1/3 of the volume. After standing at room temperature for 2 h, the precipitate of compounds **5c–i** was filtered off, washed with water, and dried.

For **5k**,**m**: To a stirring suspension of imidazo[4,5-*e*]thiazolo[2,3-*c*][1,2,4]triazines **8a**,**c** (1 mmol) in MeOH (10 mL), KOH (40% aq. solution) was added (0.250 mL, 2.5 mmol for **3k** or 0.350 mL, 3.5 mmol for **3m**). The reaction mixtures were evaporated at reduced pressure. To the residual solid, H_2O (3 mL) was added and the orange solution was acidified by 2N HCl to pH 4. The water was co-evaporated under reduced pressure with 3-fold excess (9 mL) MeOH at 25 °C. The residual solid was dissolved in 5 mL MeOH, the inorganic precipitate was decanted and the methanolic solution was evaporated. The solid residue of **5k**,**m** was dried.

1,3-Dimethyl-2,9-dioxo-1,2,3,3a,4,10a-hexahydro-9*H*-imidazo[4,5-*e*][1,3]thiazino[2,3-*c*][1,2,4]triazine-7-carboxylic acid (5a). Yield 285 mg (96%); orange solid; mp: 112–114 °C. IR (KBr): ν 3300 (NH), 3046 (=CH), 3000, 2973, 2938, 2906 (Alk), 2758, 2570 (COOH) 1733, 1714, 1628, 1568 (C=O, C=N, C=C) cm^{-1}. ^{1}H NMR (300 MHz, DMSO-*d*$_6$): δ 2.58 (s, 3H, CH_3), 2.62 (s, 3H, CH_3), 5.06 (d, *J* = 8.3 Hz, 1H, 3a-H), 6.23 (d, *J* = 8.2 Hz, 1H, 10a-H), 6.68 (s, 1H, =CH), 8.11 (s, 1H, 4-NH). ^{13}C NMR (75 MHz, DMSO-*d*$_6$): δ 27.52, 28.57 (2CH_3), 67.64, 69.82 (C-3a, C-10a), 119.64 (=CH), 135.73, 142.81 (C-7, 5a-C=N), 157.63, 160.98, 162.75 (2-C=O, 9-C=O, COOH). HRMS (ESI): *m/z* [*M* + H]$^+$ calcd for $C_{10}H_{11}N_5O_4S$: 298.0605; found: 298.0609.

1,3-Ethyl-2,9-dioxo-1,2,3,3a,4,10a-hexahydro-9*H*-imidazo[4,5-*e*][1,3]thiazino[2,3-*c*][1,2,4]triazine-7-carboxylic acid (5b). Yield 305 mg (94%); yellow solid; mp: 203–204 °C. IR (KBr): ν 3346 (NH), 3077 (=CH), 2975, 2930, 2871 (Alk), 2703, 2549 (COOH) 1706, 1665, 1617, 1590 (C=O, C=N, C=C) cm^{-1}. ^1H NMR (300 MHz, DMSO-d_6): δ 0.89–1.04 (m, 6H, $2CH_3$), 2.82–2.91 (m, 1H, NCH_2), 2.93–3.08 (m, 1H, NCH_2), 3.12–3.22 (m, 1H, NCH_2), 3.25–3.37 (m, 1H, NCH_2), 5.22 (d, *J* = 8.5 Hz, 1H, 3a-H), 6.31 (d, *J* = 8.5 Hz, 1H, 10a-H), 6.68 (s, 1H, =CH), 8.10 (s, 1H, 4-NH). ^{13}C NMR (125 MHz, DMSO-d_6): δ 12.18, 13.48 ($2CH_3$), 34.33, 35.64 ($2CH_2$), 66.06, 67.81 (C-3a, C-10a), 119.65 (=CH), 135.79, 143.14 (C-7, 5a-C=N), 156.50, 161.07, 162.76 (2-C=O, 9-C=O, COOH). HRMS (ESI): *m/z* [*M* + H]$^+$ calcd for $C_{12}H_{15}N_5O_4S$: 348.0737; found: 348.0737.

2,9-Dioxo-1,3-dipropyl-1,2,3,3a,4,10a-hexahydro-9*H*-imidazo[4,5-*e*][1,3]thiazino[2,3-*c*][1,2,4]triazine-7-carboxylic acid (5c). Yield 248 mg (70%); yellow solid; mp: 198–199 °C. IR (KBr): ν 3291 (NH), 3079 (=CH), 2967, 2935, 2877 (Alk), 2585 (COOH) 1722, 1679, 1616 (C=O, C=N, C=C) cm^{-1}. ^1H NMR (300 MHz, DMSO-d_6): δ 0.71–0.88 (m, 6H, $2CH_3$), 1.30–1.59 (m, 4H, $2CH_2$), 2.73–2.87 (m, 1H, NCH_2), 2.96–3.12 (m, 2H, NCH_2), 3.19–3.31 (m, 1H, NCH_2), 5.24 (d, *J* = 8.5 Hz, 1H, 3a-H), 6.31 (d, *J* = 8.5 Hz, 1H, 10a-H), 6.69 (s, 1H, =CH), 8.13 (s, 1H, 4-NH). ^{13}C NMR (75 MHz, DMSO-d_6): δ 10.97 ($2CH_3$), 19.98, 20.79 ($2CH_3$), 41.30, 42.29 ($2CH_2$), 66.09, 68.26 (C-3a, C-10a), 119.56 (=CH), 135.72, 143.16 (C-7, 5a-C=N), 157.04, 161.03, 162.70 (2-C=O, 9-C=O, COOH). HRMS (ESI): *m/z* [*M* + H]$^+$ calcd for $C_{14}H_{19}N_5O_4S$: 354.1231; found: 354.1233.

3-Methyl-2,9-dioxo-1-phenyl-1,2,3,3a,4,10a-hexahydro-9*H*-imidazo[4,5-*e*][1,3]thiazino[2,3-*c*][1,2,4]triazine-7-carboxylic acid (5d). Yield 208 mg (58%); yellow solid; mp: 224–225 °C. IR (KBr): ν 3263 (NH), 3072 (=CH, Ph), 2997, 2917 (Alk), 2724, 2550 (COOH), 1728, 1702, 1679, 1618 (C=O, C=N, C=C) cm^{-1}. ^1H NMR (300 MHz, DMSO-d_6) 2.71 (s, 3H, NCH_3), 5.34 (d, *J* = 8.6 Hz, 1H, 3a-H), 6.40 (s, 1H, =CH), 6.91 (d, *J* = 8.6 Hz, 1H, 10a-H), 7.14–7.26 (m, 3H, Ph-2,4,6), 7.29–7.36 (m, 2H, Ph-3,5), 8.27 (s, 1H, 4-NH). ^{13}C NMR (75 MHz, DMSO-d_6) 27.27 (CH_3), 66.71, 70.27 (C-3a, C-10a), 119.34 (=CH), 126.16 (Ph-4), 126.40 (Ph-2,6), 128.13 (Ph-1), 128.44 (Ph-3,5), 136.17, 142.72 (C-7, 5a-C=N), 155.61, 159.78, 162.61 (2-C=O, 9-C=O, COOH). HRMS (ESI): *m/z* [*M* + H]$^+$ calcd for $C_{15}H_{13}N_5O_4S$: 360.0761; found: 360.0755.

3-Ethyl-2,9-dioxo-1-phenyl-1,2,3,3a,4,10a-hexahydro-9*H*-imidazo[4,5-*e*][1,3]thiazino[2,3-*c*][1,2,4]triazine-7-carboxylic acid (5e). Yield 295 mg (79%); yellow solid; mp: 225–226 °C. IR (KBr): ν 3299 (NH), 3052 (=CH, Ph), 2979, 2934, 2874 (Alk), 2752, 2568 (COOH), 1698, 1656, 1620, 1593 (C=O, C=N, C=C) cm^{-1}. ^1H NMR (300 MHz, DMSO-d_6): δ 1.05 (t, *J* = 7.1 Hz, 3H, CH_3), 3.10–3.21 (m, 1H, NCH_2), 3.28–3.38 (m, 1H, NCH_2), 5.49 (d, *J* = 8.6 Hz, 1H, 3a-H), 6.41 (s, 1H, =CH), 6.92 (d, *J* = 8.6 Hz, 1H, 10a-H), 7.16–7.28 (m, 3H, Ph-2,4,6), 7.33 (t, *J* = 7.6 Hz, 2H, Ph-3,5), 8.27 (s, 1H, 4-NH). ^{13}C NMR (75 MHz, DMSO-d_6): δ 12.19 (CH_3), 34.36 (CH_2), 66.87, 67.96 (C-3a, C-10a), 119.45 (=CH), 126.15 (Ph-4), 126.43 (Ph-2,6), 128.47 (Ph-3,5), 136.20, 136.27 142.72 (Ph-1, C-7, 5a-C=N), 155.10, 159.83, 162.70 (2-C=O, 9-C=O, COOH). HRMS (ESI): *m/z* [*M* + H]$^+$ calcd for $C_{16}H_{15}N_5O_4S$: 374.0918; found: 374.0917.

1,3-Dimethyl-9-oxo-2-thioxo-1,2,3,3a,4,10a-hexahydro-9*H*-imidazo[4,5-*e*][1,3]thiazino[2,3-*c*][1,2,4]triazine-7-carboxylic acid (5f). Yield 222 mg (72%); yellow solid, mp: 168–169 °C. IR (KBr): ν 3326 (NH), 3066 (=CH), 2926 (Alk), 2572 (COOH) 1718, 1670, 1616 (C=O, C=N, C=C), 1300 (2-C=S) cm^{-1}. ^{1}H NMR (300 MHz, DMSO-d_6): δ 2.91 (s, 3H, NCH_3), 2.93 (s, 3H, NCH_3), 5.42 (d, *J* = 8.9 Hz, 1H, 3a-H), 6.51 (d, *J* = 8.9 Hz, 1H, 10a-H), 6.67 (s, 1H, =CH), 8.32 (s, 1H, 4-NH). ^{13}C NMR (75 MHz, DMSO-d_6): δ 31.39 (NCH_3), 32.86 (NCH_3), 69.56, 73.51 (C-3a, C-10a), 119.22 (=CH), 137.63 (C-7), 145.01 (5a-C=N), 161. 21, 162.83 (9-C=O, COOH), 182.22 (2-C=S). HRMS (ESI): *m/z* [*M* + H]$^+$ calcd for $C_{10}H_{11}N_5O_3S_2$: 314.0376; found: 314.0390.

3-Methyl-9-oxo-1-phenyl-2-thioxo-1,2,3,3a,4,10a-hexahydro-9*H*-imidazo[4,5-*e*][1,3]thiazino[2,3-*c*][1,2,4]triazine-7-carboxylic acid (5g). Yield 311 mg (83%); yellow solid; mp: 203–204 °C. IR (KBr): ν 3296 (NH), 3067, 3008 (=CH, Ph), 2925, 2839 (Alk), 1663, 1640, 1616 (C=O, C=N, C=C), 1302 (C=S) cm^{-1}. ^{1}H NMR (300 MHz, DMSO-d_6): δ 3.05 (s, 3H, NCH_3), 5.70 (d, *J* = 9.4 Hz, 1H, 3a-H), 6.38 (s, 1H, =CH), 6.97 (d, *J* = 9.3 Hz, 1H, 10a-H), 7.21 (d, *J* = 7.2 Hz, 2H, Ph-2,6), 7.28–7.45 (m, 3H, Ph-3-5), 8.51 (s, 1H, 4-NH). ^{13}C NMR (75 MHz, DMSO-d_6): δ 30.97 (CH_3), 69.39, 74.14 (C-3a, C-10a), 119.23 (=CH), 127.81 (Ph-4), 128.77, 129.00 (Ph-2,6, Ph-3,5), 137.11, 137.45 142.71 (Ph-1, C-7, 5a-C=N), 159.25, 162.64 (9-C=O, COOH), 180.74 (2-C=S). HRMS (ESI): *m/z* [*M* + H]$^+$ calcd for $C_{15}H_{13}N_5O_3S_2$: 376.0533; found: 376.0518.

3-Ethyl-9-oxo-1-phenyl-2-thioxo-1,2,3,3a,4,10a-hexahydro-9*H*-imidazo[4,5-*e*][1,3]thiazino[2,3-*c*][1,2,4]triazine-7-carboxylic acid (5h). Yield 346 mg (89%); yellow solid; mp: 202–203 °C. IR (KBr): ν 3304 (NH), 3062 (=CH, Ph), 2975, 2932, 2873 (Alk), 2580 (COOH), 1687, 1658, 1615, 1591 (C=O, C=N, C=C), 1265 (C=S) cm^{-1}. ^{1}H NMR (300 MHz, DMSO-d_6): δ 1.12 (t, *J* = 7.0 Hz, 3H, CH_3), 3.42–3.54 (m, 1H, NCH_2), 3.70–3.81 (m, 1H, NCH_2), 5.79 (d, *J* = 9.4 Hz, 1H, 3a-H), 6.37 (s, 1H, =CH), 6.93 (d, *J* = 9.3 Hz, 1H, 10a-H), 7.17–7.23 (m, 2H, Ph-2,6), 7.24–7.40 (m, 3H, Ph-3-5), 8.48 (s, 1H, 4-NH). ^{13}C NMR (75 MHz, DMSO-d_6): δ 11.34 (CH_3), 37.85 (CH_2), 69.40, 72.14 (C-3a, C-10a), 118.97 (=CH), 127.72 (Ph-4), 128.29 (Ph-4), 128.68, 129.03 (Ph-2,6, Ph-3,5), 137.39, 143.19 (C-7, 5a-C=N), 159.28, 162.54 (9-C=O, COOH), 180.03 (2-C=S). HRMS (ESI): *m/z* [*M* + H]$^+$ calcd for $C_{16}H_{15}N_5O_3S_2$: 390.0689; found: 390.0687.

3-(2-Hydroxyethyl)-9-oxo-1-phenyl-2-thioxo-2,3,3a,4,9,10a-hexahydro-1*H*-imidazo[4,5-*e*][1,3]thiazino[2,3-*c*][1,2,4]triazine-7-carboxylic acid (5i). Yield 190 mg (47%); yellow solid; mp: 181–183 °C. IR (KBr): ν 3540, 3289 (NH, OH), 3074, 3062 (=CH, Ph), 2933, 2879 (Alk), 1704, 1682, 1642, 1612 (C=O, C=N, C=C) cm^{-1}. ^{1}H NMR (300 MHz, DMSO-d_6): δ 3.57-3.82 (m, 4H, $2CH_2$), 5.84 (d, *J* = 9.5 Hz, 1H, 3a-H), 6.36 (s, 1H, =CH), 6.98 (d, *J* = 9.4 Hz, 1H, 10a-H), 7.20 (d, *J* = 7.2 Hz, 2H, Ph-2,6), 7.26–7.38 (m, 3H, Ph-3-5), 8.43 (s, 1H, 4-NH). ^{13}C NMR (75 MHz, DMSO-d_6): δ 46.06 (NCH_2), 58.62, 69.60, 74.21 69.40, 72.14 (CH_2, C-3a, C-10a), 119.61 (=CH), 128.32 (Ph-4), 129.21, 129.49 (Ph-2,3,5,6), 137.40, 137.74, 143.24 (5a-C=N, C-7, Ph-1), 159.75, 162.93 (9-C=O, COOH), 180.91 (2-C=S). HRMS (ESI): *m/z* [*M* + H]$^+$ calcd for $C_{16}H_{15}N_5O_4S_2$: 406.0639; found: 406.0646.

1-(4-Chlorophenyl)-3-methyl-9-oxo-2-thioxo-2,3,3a,4,9,10a-hexahydro-1H-imidazo[4,5-e][1,3]thiazino[2,3-c][1,2,4]triazine-7-carboxylic acid (5j). Yield 307 mg (75%); yellow solid; mp: 212–214 °C. IR (KBr): ν 3507, 3351 (NH), 3073 (=CH, Ph), 2927 (Alk), 1719, 1682, 1615 (C=O, C=N, C=C) cm^{-1}. ^{1}H NMR (300 MHz, DMSO-d_6): δ 3.03

(s, 3H, NCH_3), 5.68 (d, *J* = 9.2 Hz, 1H, 3a-H), 6.43 (s, 1H, =CH), 6.94 (d, *J* = 9.2 Hz, 1H, 10a-H), 7.23 (d, *J* = 8.6 Hz, 2H, Ar), 7.44 (d, *J* = 8.5 Hz, 2H, Ar), 8.49 (s, 1H, 4-NH). ^{13}C NMR (75 MHz, DMSO-*d*6): δ 31.44 (CH_3), 69.76, 74.39 (C-3a, C-10a), 119.77 (=CH), 129.31, 131.30, 132.72 (Ar-2-6), 137.01, 137.51, 143.13 (Ph-1, C-7, 5a-C=N), 159.92, 162.93 (9-C=O, COOH), 181.11 (2-C=S). HRMS (ESI): *m/z* [*M* + H]$^+$ calcd for $C_{15}H_{12}Cl_5O_3S_2$: 410.0143 found: 410.0151.

8-(2-Methoxy-2-oxoethyl)-1,3-dimethyl-2,9-dioxo-1,2,3,3a,4,10a-hexahydro-9H-imidazo[4,5-*e*][1,3]thiazino[2,3-*c*][1,2,4]triazine-7-carboxylic acid (5k). Yield 332 mg (90%); yellow solid; mp: 170–171 °C. IR (KBr): ν 3328 (NH), 2954 (Alk), 2540 (COOH) 1707, 1665, 1616 (C=O, C=N, C=C) cm^{-1}. ^{1}H NMR (300 MHz, DMSO-*d*6): δ 2.59 (s, 3H, NCH_3), 2.61 (s, 3H, NCH_3), 3.59 (s, 3H, OCH_3), 3.83 (d, *J* = 16.9 Hz, 1H, CH_2), 3.95 (d, *J* = 17.1 Hz, 1H, CH_2), 5.07 (d, *J* = 8.3 Hz, 1H, 3a-H), 6.21 (d, *J* = 8.3 Hz, 1H, 10a-H), 8.10 (s, 1H, 4-NH). ^{13}C NMR (75 MHz, DMSO-*d*6): δ 27.97 (NCH_3), 29.03 (NCH_3), 33.68 (CH_2), 52.22 (OCH_3), 69.13 (C-10a), 70.19 (C-3a), 124.56 (C-8), 135.97 (5a-C=N), 140.58 (C-7), 158.11 (2-C=O), 162.61 (9-C=O), 163.80 (COOH), 170.84 (COOMe). HRMS (ESI): *m/z* [*M* + Na]$^+$ calcd for $C_{13}H_{14}N_5NaO_6S$: 392.0635; found: 392.0649.

8-(2-Methoxy-2-oxoethyl)-3-methyl-9-oxo-1-phenyl-2-thioxo-1,2,3,3a,4,10a-hexahydro-9*H*-imidazo[4,5-*e*][1,3]thiazino[2,3-*c*][1,2,4]triazine-7-carboxylic acid (5m). Yield 237 mg (53%); yellow solid; mp: 154–155 °C. IR (KBr): ν 3326 (NH), 3062, 3037 (Ph), 2951, 2931 (Alk), 2556 (COOH) 1726, 1673, 1613 (C=O, C=N, C=C), 1303 (2-C=S) cm^{-1}. ^{1}H NMR (300 MHz, DMSO-*d*6): δ 3.03 (s, 3H, NCH_3), 3.41-3.58 (m, 4H, OCH_3, =C-CH_2), 3.67 (d, *J* = 17.0 Hz, 1H, =C-CH_2), 5.69 (d, *J* = 9.4 Hz, 1H, 3a-H), 6.90 (d, *J* = 9.2 Hz, 1H, 10a-H), 7.18 (d, *J* = 7.5 Hz, 2H, Ph-2,6), 7.25-7.37 (m, 3H, Ph-3-5), 8.47 (s, 1H, 4-NH). ^{13}C NMR (75 MHz, DMSO-*d*6): δ 31.43 (NCH_3), 33.40 (=C-CH_2), 52.18 (OCH_3), 70.88, 74.39 (C-3a, C-10a), 124.60 (C-8), 128.10 (Ph-4), 129.17, 129.40 (Ph-2,6, Ph-3,5), 137.21, 138.07 (C-7, Ph-1), 161. 01, 163.79, 170.24 (5a-C=N, 9-C=O, COOH, COOMe), 181.34 (2-C=S). HRMS (ESI): *m/z* [*M* + H]$^+$ calcd for $C_{18}H_{17}N_5O_5S_2$: 448.0744; found: 448.0743.

Procedure for the synthesis imidazo[4,5-*e*][1,2,4]triazines 7i,j

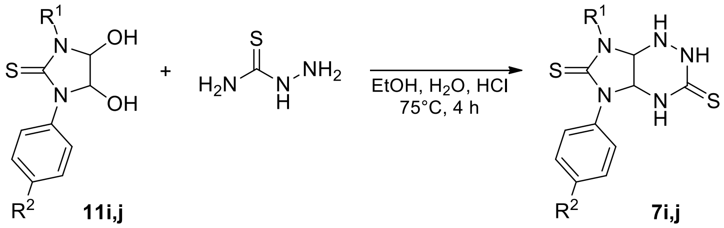

7, 11: R^1=CH_2CH_2OH, R^2=Ph (**i**); R^1=Me, R^2=4-ClC_6H_4 (**j**)

To a stirred suspension of **11i,j** (10 mmol) and thiosemicarbazide (0.91 g, 10 mmol) in a mixture of EtOH (10 mL) and H_2O (1 mL) at 75 °C, conc. HCl (0.4 mL) was added. The mixture was stirred for 4 h and cooled. The precipitate of compounds **7i,j** was filtered off, washed with MeCN, followed by MeOH, and dried.

7-(2-Hydroxyethyl)-5-phenyltetrahydro-1*H*-imidazo[4,5-*e*][1,2,4]triazine-3,6(2*H*,4*H*)-dithione (7i). Yield 1.550 g (50%); white solid; mp: 231–233 °C. ^{1}H NMR (300 MHz, DMSO-*d*6): δ 3.35-3.79 (m, 4H, CH_2), 5.37 (d, *J* = 7.3 Hz, 1H, CH), 5.46 (d, *J* = 7.3 Hz, 1H, CH), 5.97 (d, *J* = 7.3 Hz, 1H, 1-NH), 7.28-7.44 (m, 6H, Ph, OH), 8.69 (s, 1H, 4-NH), 9.65 (s, 1H, 2-NH). ^{13}C

NMR (75 MHz, DMSO-d_6): δ 45.82 (NCH_2), 58.24 (OCH_2), 66.69, 73.30 (C-4a, C-7a), 127.42 (Ph-4), 128.71, 129.18 (Ph-2,3,5,6), 138.08 (Ph-1), 181.12, 186.51 (2C=S). HRMS (ESI): *m/z* [*M* + H]$^+$ calcd for $C_{12}H_{15}N_5OS_2$: 310.0791; found: 310.0790.

5-(4-Chlorophenyl)-7-methyltetrahydro-1H-imidazo[4,5-e][1,2,4]triazine-3,6(2H,4H)-dithione (7j). Yield 1.774 g (57%); light beige solid; mp: 234–236 °C. IR (KBr): ν 3202 (NH), 2944 (Alk), 1498 (C=S) cm^{-1}. ^{1}H NMR (300 MHz, DMSO-d_6): δ 3.06 (s, 3H, CH_3), 5.33-5.42 (m, 2H, 4a-H, 7a-H), 7.32 (d, *J* = 8.5 Hz, 2H, Ar), 7.50 (d, *J* = 8.5 Hz, 2H, Ar), 8.77 (s, 1H, 4-NH), 9.69 (s, 1H, 2-NH). ^{13}C NMR (75 MHz, DMSO-d_6): δ 31.22 (NCH_3), 66.61, 73.76 (C-4a, C-7a), 128.71, 130.95, 131.84 (Ar-2-6), 137.05 (Ar-1), 180.98, 186.18 (2C=S). HRMS (ESI): *m/z* [*M* + H]$^+$ calcd for $C_{11}H_{12}ClN_5S_2$: 314.0295; found: 314.0294.

Procedure for the synthesis of compounds 9k,l

To a stirring suspension of imidazo[4,5-*e*]thiazolo[2,3-*c*][1,2,4]triazines **8a**,**b** (1 mmol) in MeOH (10 mL), 40% aq. solution KOH (0.250 mL (2.5 mmol) for **9k** or 0.350 mL (3.5 mmol) for **9l**) was added and the mixture was stirred at room temperature (1 h for **9k** or 24 h for **9l**). Then, the reaction mixtures were acidified by the addition of 0.200 mL of conc. HCl. The solvent was evaporated at reduced pressure at 40 °C. To the residual solid, H_2O (10 mL) was added. The resulting mixture was stirred and the precipitate formed was filtered off, washed with 10 mL of water and dried.

(*Z*)-2-(((*E*)-1,3-dimethyl-2-oxoimidazolidin-4-ylidene)hydrazineylidene)-5-(2-methoxy-2-oxoethyl)-4-oxo-3,4-dihydro-2*H*-1,3-thiazine-6-carboxylic acid (9k). Yield 185 mg (50%); yellow solid; mp: 199–200 °C. IR (KBr): ν 3139 (NH), 2988, 2950 (Alk), 2850 (C-S), 2568 (COOH) 1731, 1643 (C=N, C=O, NH-C=O), 1586 (C=C-S) cm^{-1}. ^{1}H NMR (300 MHz, DMSO-d_6): δ 2.83 (s, 3H, NCH_3), 2.95 (s, 3H, NCH_3), 3.60 (s, 3H, OCH_3), 3.82 (s, 2H, =C-CH_2), 4.21 (s, 2H, NCH_2), 11.73 (br.s, 1H, NH). ^{13}C NMR (75 MHz, DMSO-d_6): δ 26.74, 30.06 (2NCH_3), 33.35 (CH_2), 48.48 (CH_2), 52.23 (OCH_3), 126.93 (=C), 138.53 (=C-S), 148.78, 157.24, 157.76, 162.79, 164.46, 170.69 (NH-C=N, C-C=N, CONH, COOH, COOMe). HRMS (ESI): *m/z* [*M* + Na]$^+$ calcd for $C_{13}H_{15}N_5O_6S$: 392.0635; found: 392.0628.

(*Z*)-5-(2-methoxy-2-oxoethyl)-2-(((*E*)-3-methyl-2-oxo-1-phenylimidazolidin-4-ylidene) hydrazineylidene)-4-oxo-3,4-dihydro-2*H*-1,3-thiazine-6-carboxylic acid (9l). Yield 190 mg (44%); yellow solid; mp: 188–189 °C. IR (KBr): ν 3124 (NH), 3037 (Ph), 2950 (Alk), 2856 (C-S), 2575 (COOH) 1738, 1704, 1662 (C=N, C=O, NH-C=O), 1584 (C=C-S) cm^{-1}. ^{1}H NMR (300 MHz, DMSO-d_6): δ 3.06 (s, 3H, NCH_3), 3.60 (s, 3H, OCH_3), 3.83 (s, 2H, =C-CH_2), 4.73 (s, 2H, CH_2), 7.10 (t, *J* = 7.3 Hz, 1H, Ph-4), 7.39 (t, *J* = 7.9 Hz, 2H, Ph-3,5), 7.58 (d, *J* = 7.5 Hz, 2H, Ph-2,6), 11.93 (br. s, 1H, NH). ^{13}C NMR (75 MHz, DMSO-d_6): δ 26.77 (NCH_3), 33.37 (=C-CH_2), 47.03 (CH_2), 52.25 (OCH_3), 118.18 (Ph-3,5), 123.67, 127.07 (=C, Ph-4), 129.47 (Ph-2,6), 138.43, 138.95 (=C-S, Ph-1), 149.59, 154.73, 155.95, 162.77, 164.39, 170.68 (NH-C=N, C-C=N, NH-C=O, COOH, COOMe). HRMS (ESI): *m/z* [*M* + H]$^+$ calcd for $C_{18}H_{17}N_5O_6S$: 432.0972; found: 432.0966.

Procedure for the synthesis of 1-(4-chlorophenyl)-3-methylthiourea (10)

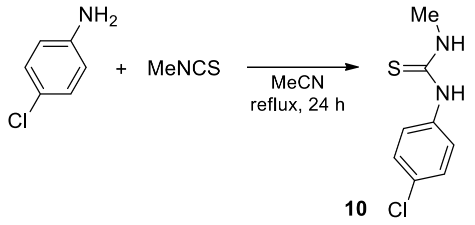

A solution of 4-chloroaniline (12.7 g, 50 mmol) and methyl isothiocyanate (7.3 g, 50 mmol) in MeCN (10 mL) was refluxed for 24 h, cooled, and evaporated under reduced pressure. The solid residue in the flask was triturated with 2 mL of cold acetonitrile, filtered, and dried.

1-(4-Chlorophenyl)-3-methylthiourea (10). Yield 8.05 g (80%); white solid; mp: 140–141 °C. ^{1}H NMR (300 MHz, DMSO-d_6): δ 2.92 (s, 3H, NCH_3), 7.36 (d, *J* = 7.9 Hz, 2H, Ar), 7.45 (d, *J* = 7.9 Hz, 2H, Ar), 7.77 (br.s, 1H, 3-NH), 9.62 (br.s, 1H, 1-NH). ^{13}C NMR (75 MHz, DMSO-d_6): δ 31.08 (NCH_3), 124.73, 128.03, 128.50 (Ar-2-6), 138.32 (Ar-1), 181.14 (C=S). HRMS (ESI): *m/z* $[M + H]^+$ calcd for $C_8H_9N_2S$: 201.0248; found: 201.0253.

Procedure for the synthesis of 1-(4-chlorophenyl)-4,5-dihydroxy-3-methylimidazolidine-2-thione (11j)

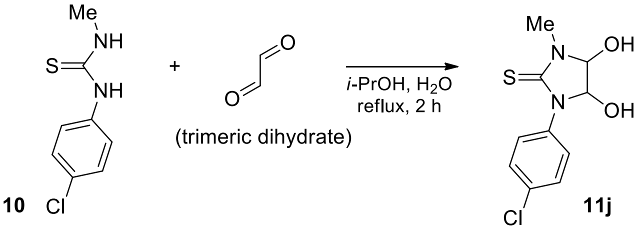

A solution of thiourea **10** (6.0 g, 30 mmol) and glyoxal trimeric dihydrate (2.1 g, 10 mmol (equivalent to 30 mmol of glyoxal) in a mixture of iPrOH (5 mL) and H_2O (0.5 mL) was refluxed for 2 h, cooled, and evaporated under reduced pressure. After standing for 1 hour, the oil crystallizes into a solid and was used without further purification.

Crystallographic data

Single crystals of **7a** were obtained from a methanol solution. Data were collected at 100K on a Bruker Quest D8 diffractometer equipped with a Photon-III area-detector (shutterless φ- and ω-scan technique), using graphite-monochromatized MoK_α radiation. The intensity data were integrated by the SAINT program[5] and were corrected for absorption and decay using SADABS.[6] The structure was solved by direct methods using SHELXT[7] and refined on F^2 using SHELXL-2018.[8] Positions of all atoms were found from the electron density difference map. Atoms were refined with individual anisotropic (non-hydrogen atoms) or isotropic (hydrogen atoms) displacement parameters. The SHELXTL program suite[5] was used for molecular graphics. Full crystallographic data have been deposited at the Cambridge Crystallographic Data Center (deposit CCDC 2250872).

References

1. A. N. Izmest'ev, D. B. Vinogradov, N. G. Kolotyrkina, A. N. Kravchenko and G. A. Gazieva, *Beilstein J. Org. Chem*., **2021**, 17, 1141.
2. A. N. Izmest'ev, L. V. Anikina, I. E. Zanin, N. G. Kolotyrkina, E. S. Izmalkova, A. N. Kravchenko, G. A. Gazieva *New J. Chem*. **2022**, *46,* 11632.
3. A. N. Izmest'ev, V. A. Motornov, D. B. Vinogradov, S. L. Ioffe, A. N. Kravchenko, G. A. Gazieva *Org. Chem. Front*., **2022**, 9, 4998.
4. V. V. Baranov, Y. V. Nelyubina, A. N. Kravchenko, N. G. Kolotyrkina, K. A. Biriukova *Tetrahedron Lett*. **2015**, 56, 6085.
5. Bruker. APEX-III. *Bruker AXS Inc.*, Madison, Wisconsin, USA, **2019**.
6. L. Krause, R. Herbst-Irmer, G. M. Sheldrick and D. Stalke, *J. Appl. Crystallogr.* **2015**, 48, 3.
7. G. M. Sheldrick, *Acta Crystallogr., Sect. A: Found. Crystallogr.,* **2015**, 71, 3.
8. G. M. Sheldrick, *Acta Crystallogr., Sect. C: Struct. Chem.,* **2015**, 71, 3.

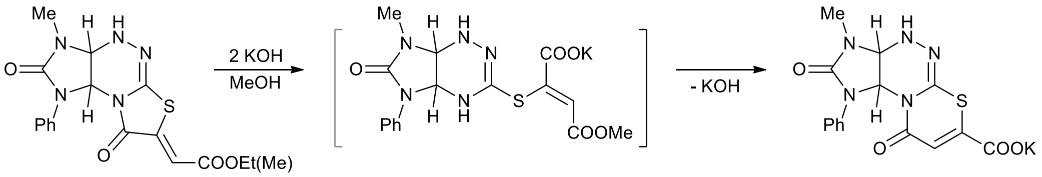
2 KOH
MeOH
- KOH
COOK
COOMe
COOEt(Me)

1d **6d** **3d**

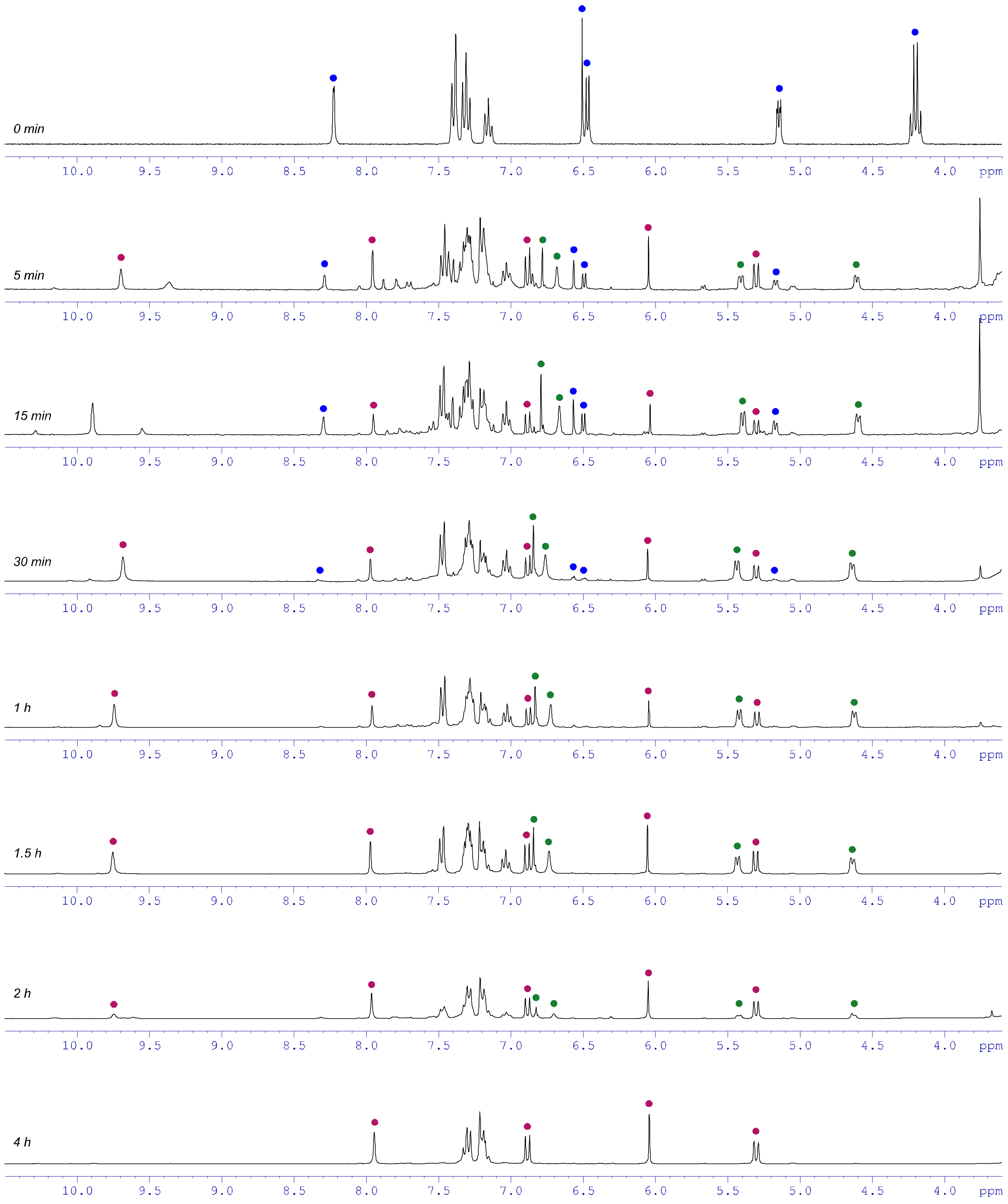
0 min
5 min
15 min
30 min
1 h
1.5 h
2 h
4 h
ppm

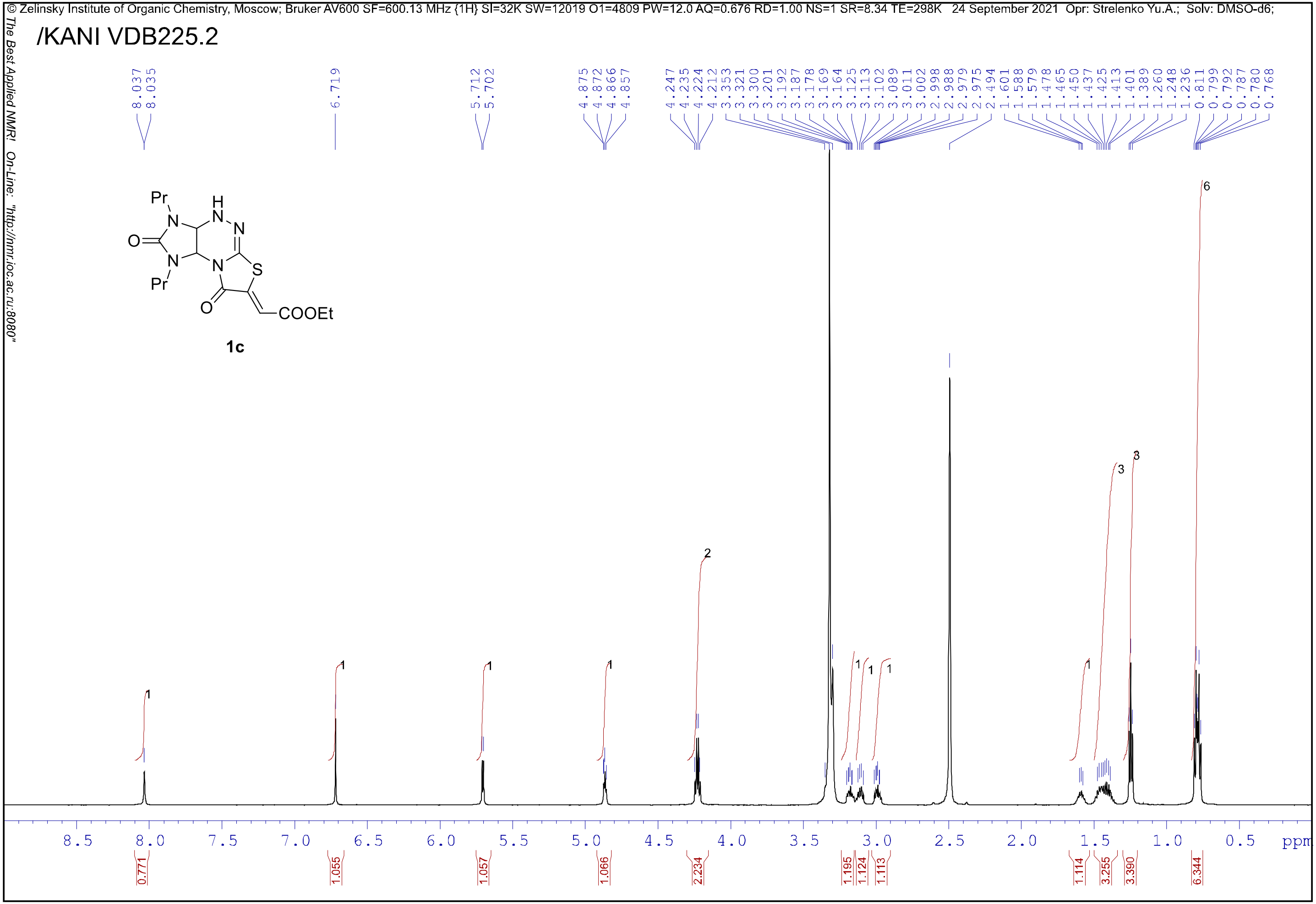
© Zelinsky Institute of Organic Chemistry, Moscow; Bruker AV600 SF=600.13 MHz {1H} SI=32K SW=12019 O1=4809 PW=12.0 AQ=0.676 RD=1.00 NS=1 SR=8.34 TE=298K 24 September 2021 Opr: Strelenko Yu.A.; Solv: DMSO-d6;
The Best Applied NMR! On-Line: "http://nmr.ioc.ac.ru:8080"
/KANI VDB225.2
8.037 8.035 6.719 5.712 5.702 4.875 4.872 4.866 4.857 4.247 4.235 4.224 4.212 3.353 3.321 3.300 3.201 3.192 3.187 3.178 3.169 3.164 3.125 3.113 3.102 3.089 3.011 3.002 2.998 2.988 2.979 2.975 2.494 1.601 1.588 1.579 1.478 1.465 1.450 1.437 1.425 1.413 1.401 1.389 1.260 1.248 1.236 0.811 0.799 0.792 0.787 0.780 0.768
Pr
H
N
N
N
O
S
COOEt
1c
0.771 1.055 1.057 1.066 2.234 1.195 1.124 1.113 1.114 3.255 3.390 6.344
8.5 8.0 7.5 7.0 6.5 6.0 5.5 5.0 4.5 4.0 3.5 3.0 2.5 2.0 1.5 1.0 0.5 ppm

© Zelinsky Institute of Organic Chemistry, Moscow; Bruker AV600 SF=150.90 MHz {13C} SI=32K SW=39058 O1=17971 PW=12.0 AQ=0.418 RD=1.00 NS=41 SR=66.53 TE=298K 27 September 2021 Opr: Strelenko Yu.A.; Solv: DMSO-d6;

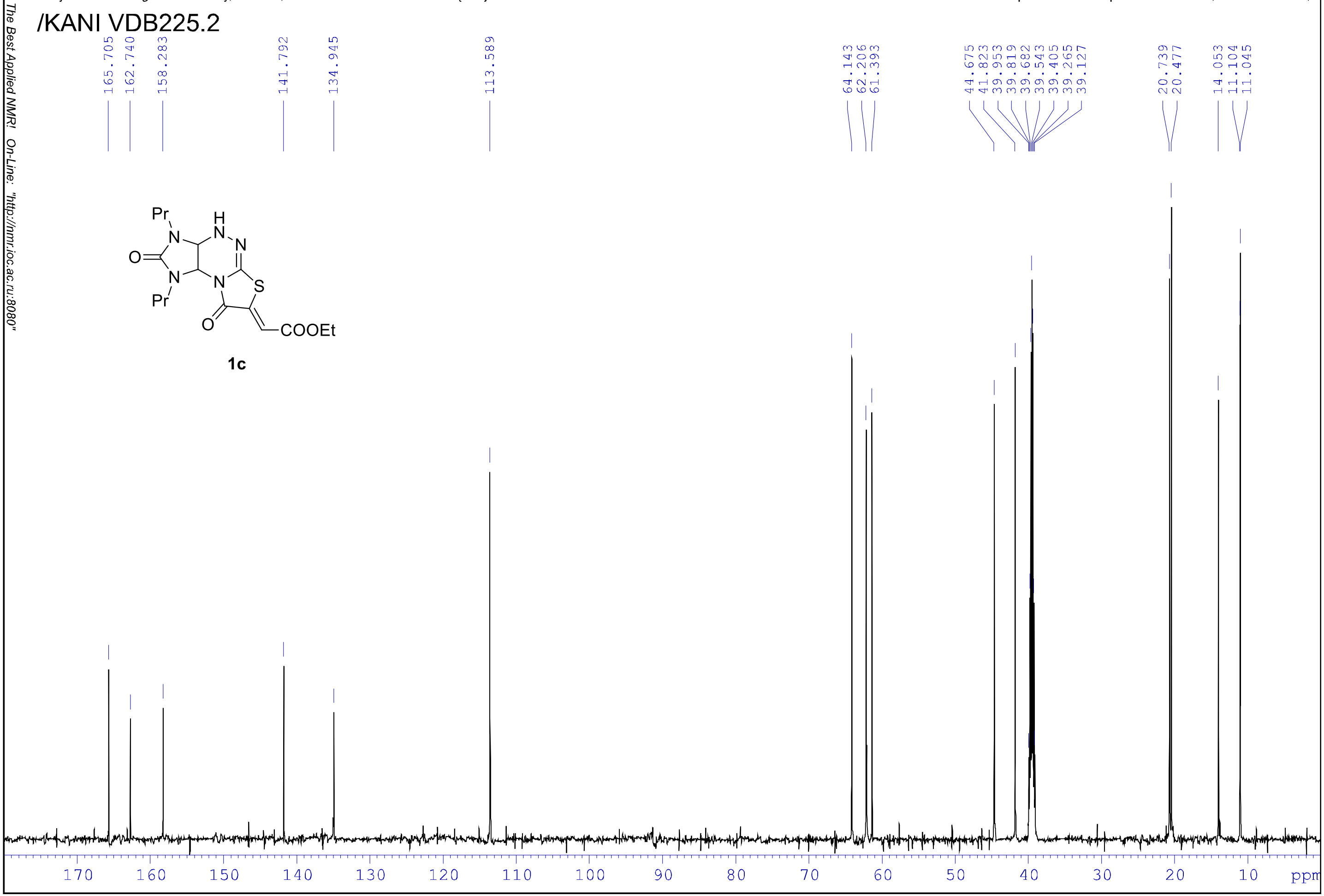

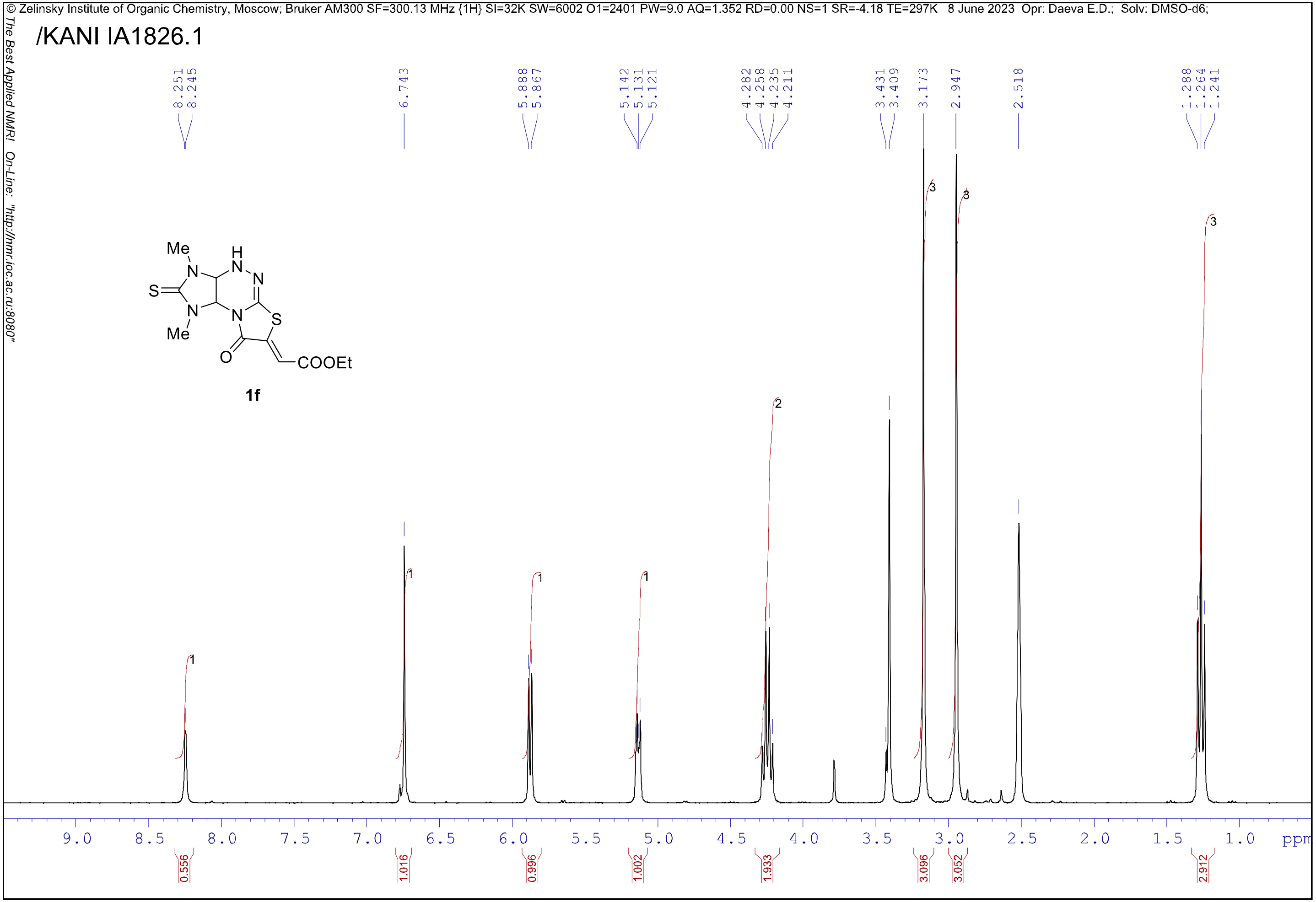
© Zelinsky Institute of Organic Chemistry, Moscow; Bruker AM300 SF=300.13 MHz {1H} SI=32K SW=6002 O1=2401 PW=9.0 AQ=1.352 RD=0.00 NS=1 SR=-4.18 TE=297K 8 June 2023 Opr: Daeva E.D.; Solv: DMSO-d6;
The Best Applied NMR! On-Line: "http://nmr.ioc.ac.ru:8080"
/KANI IA1826.1
8.251
8.245
6.743
5.888
5.867
5.142
5.131
5.121
4.282
4.258
4.235
4.211
3.431
3.409
3.173
2.947
2.518
1.288
1.264
1.241
Me
H
N
N
N
S
N
N
S
Me
O
COOEt
1f
1
1
1
1
2
3
3
3
9.0
8.5
8.0
7.5
7.0
6.5
6.0
5.5
5.0
4.5
4.0
3.5
3.0
2.5
2.0
1.5
1.0
ppm
0.556
1.016
0.996
1.002
1.933
3.096
3.052
2.912

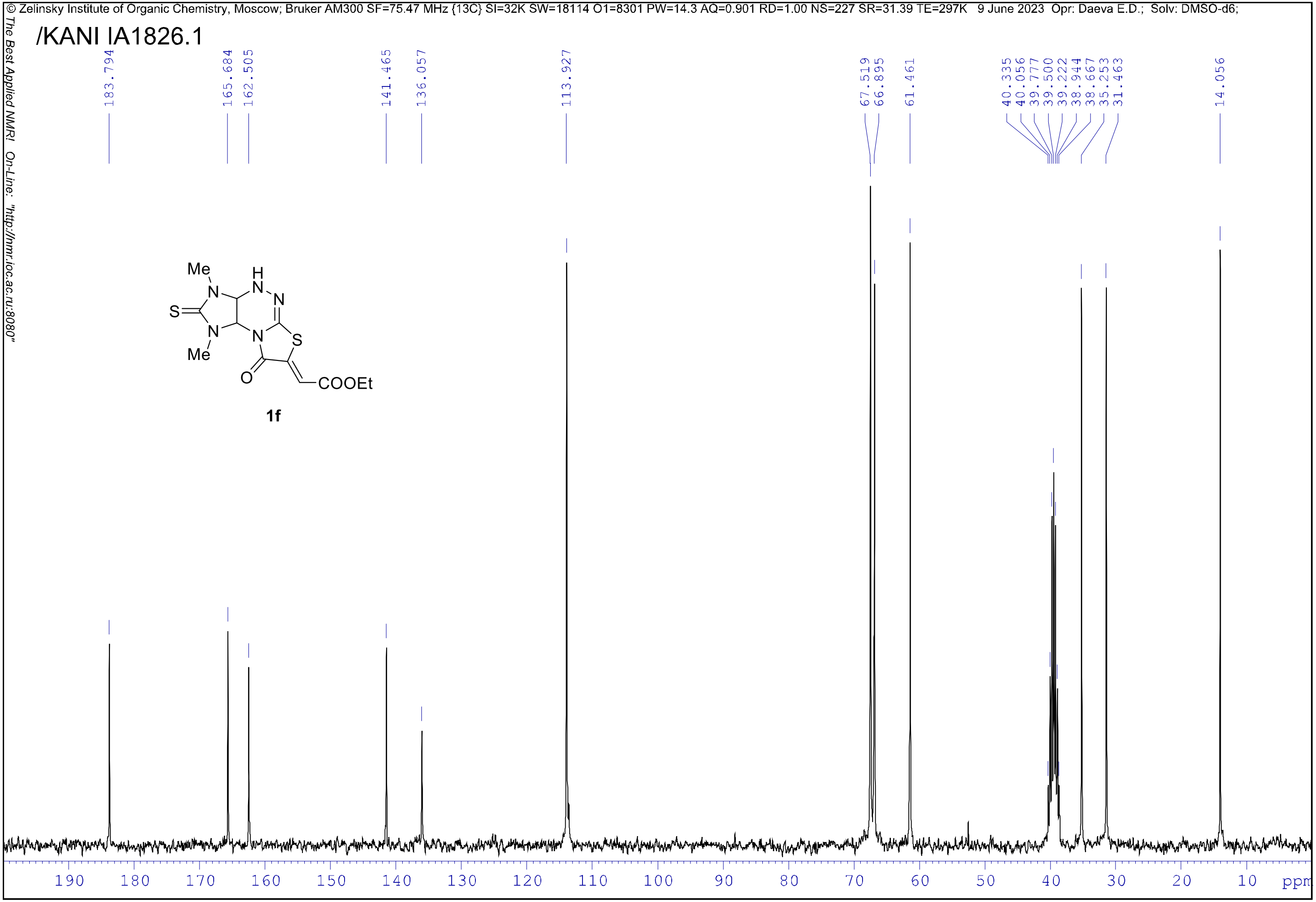
© Zelinsky Institute of Organic Chemistry, Moscow; Bruker AM300 SF=75.47 MHz {13C} SI=32K SW=18114 O1=8301 PW=14.3 AQ=0.901 RD=1.00 NS=227 SR=31.39 TE=297K 9 June 2023 Opr: Daeva E.D.; Solv: DMSO-d6;
The Best Applied NMR! On-Line: "http://nmr.ioc.ac.ru:8080"
/KANI IA1826.1
183.794
165.684
162.505
141.465
136.057
113.927
67.519
66.895
61.461
40.335
40.056
39.777
39.500
39.222
38.944
38.667
35.253
31.463
14.056
Me
H
N
N
N
S
N
N
Me
S
O
COOEt
1f
190 180 170 160 150 140 130 120 110 100 90 80 70 60 50 40 30 20 10 ppm

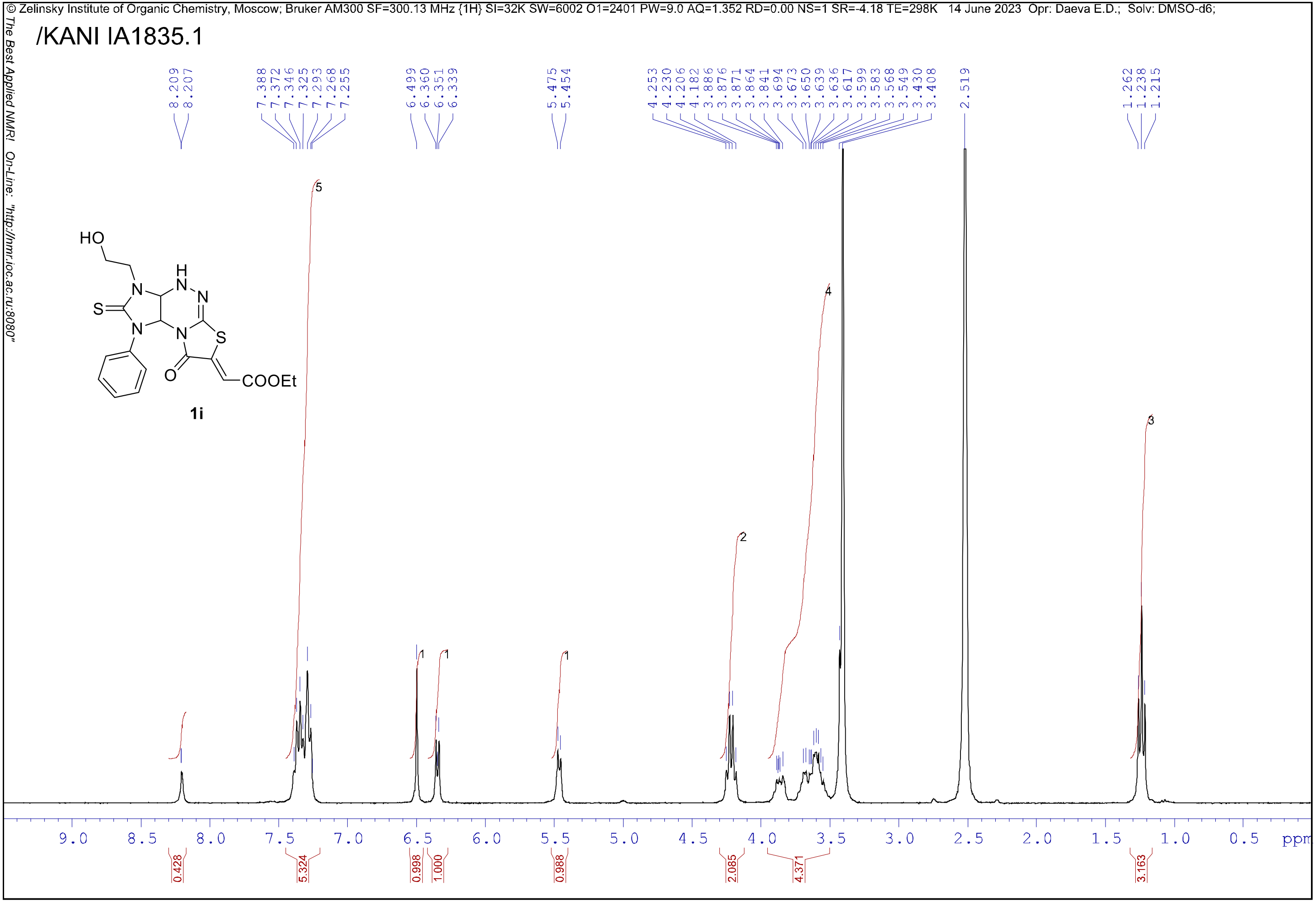
© Zelinsky Institute of Organic Chemistry, Moscow; Bruker AM300 SF=300.13 MHz {1H} SI=32K SW=6002 O1=2401 PW=9.0 AQ=1.352 RD=0.00 NS=1 SR=-4.18 TE=298K 14 June 2023 Opr: Daeva E.D.; Solv: DMSO-d6;
The Best Applied NMR! On-Line: "http://nmr.ioc.ac.ru:8080"
/KANI IA1835.1
8.209 8.207
7.388 7.372 7.346 7.325 7.293 7.268 7.255
6.499 6.360 6.351 6.339
5.475 5.454
4.253 4.230 4.206 4.182 3.886 3.876 3.871 3.864 3.841 3.694 3.673 3.650 3.639 3.636 3.617 3.599 3.583 3.568 3.549 3.430 3.408
2.519
1.262 1.238 1.215
HO
H
N
S
O
COOEt
1i
9.0 8.5 8.0 7.5 7.0 6.5 6.0 5.5 5.0 4.5 4.0 3.5 3.0 2.5 2.0 1.5 1.0 0.5 ppm
0.428 5.324 0.998 1.000 0.988 2.085 4.371 3.163

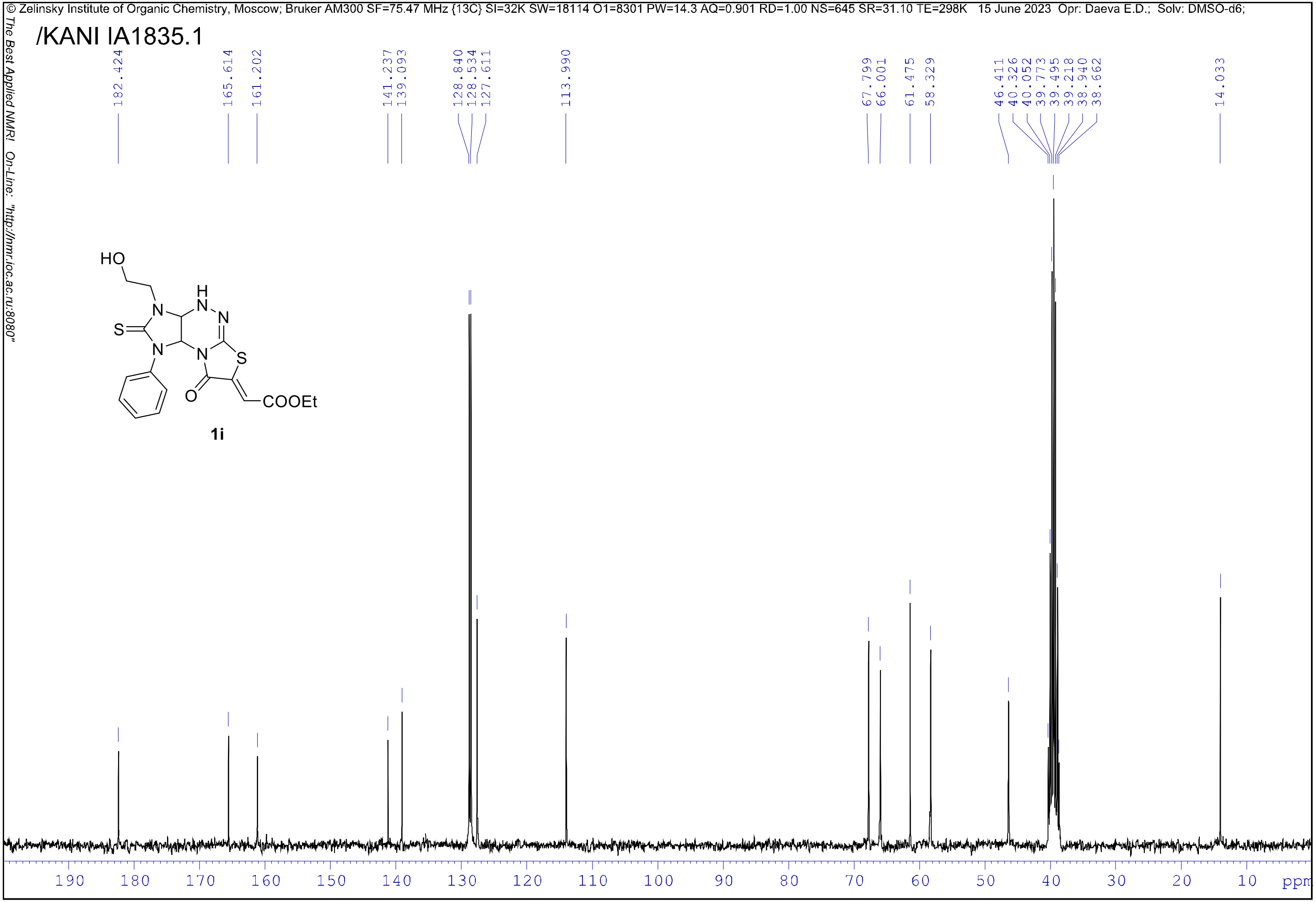
© Zelinsky Institute of Organic Chemistry, Moscow; Bruker AM300 SF=75.47 MHz {13C} SI=32K SW=18114 O1=8301 PW=14.3 AQ=0.901 RD=1.00 NS=645 SR=31.10 TE=298K 15 June 2023 Opr: Daeva E.D.; Solv: DMSO-d6;
The Best Applied NMR! On-Line: "http://nmr.ioc.ac.ru:8080"
/KANI IA1835.1
182.424
165.614
161.202
141.237
139.093
128.840
128.534
127.611
113.990
67.799
66.001
61.475
58.329
46.411
40.326
40.052
39.773
39.495
39.218
38.940
38.662
14.033
HO
H
N
S
O
COOEt
1i
190 180 170 160 150 140 130 120 110 100 90 80 70 60 50 40 30 20 10 ppm

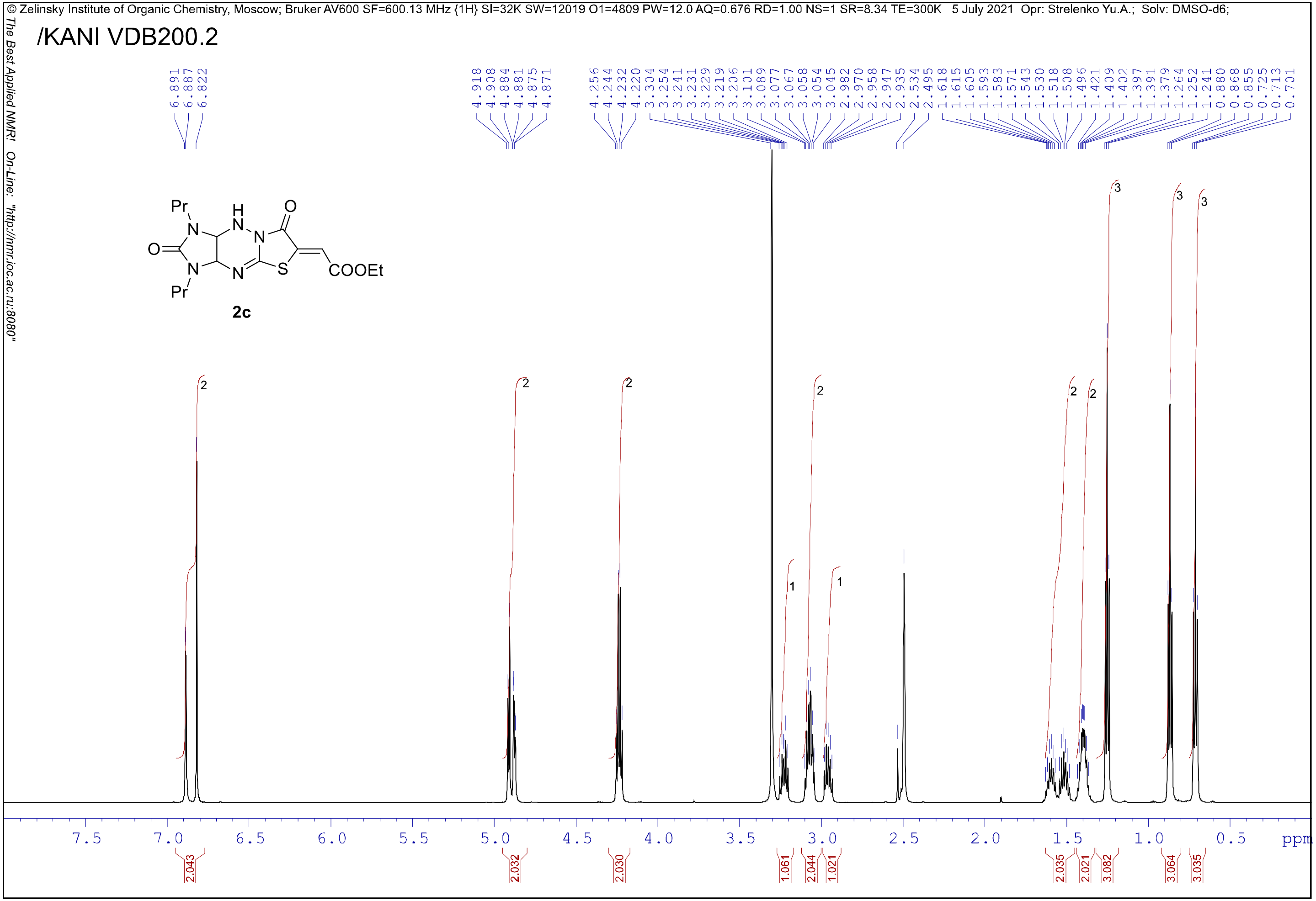
© Zelinsky Institute of Organic Chemistry, Moscow; Bruker AV600 SF=600.13 MHz {1H} SI=32K SW=12019 O1=4809 PW=12.0 AQ=0.676 RD=1.00 NS=1 SR=8.34 TE=300K 5 July 2021 Opr: Strelenko Yu.A.; Solv: DMSO-d6;
The Best Applied NMR! On-Line: "http://nmr.ioc.ac.ru:8080"
/KANI VDB200.2
6.891 6.887 6.822
4.918 4.908 4.884 4.881 4.875 4.871
4.256 4.244 4.232 4.220 3.304 3.254 3.241 3.231 3.229 3.219 3.206 3.101 3.089 3.077 3.067 3.058 3.054 3.045 2.982 2.970 2.958 2.947 2.935 2.534 2.495
1.618 1.615 1.605 1.593 1.583 1.571 1.543 1.530 1.518 1.508 1.496 1.421 1.409 1.402 1.397 1.391 1.379 1.264 1.252 1.241 0.880 0.868 0.855 0.725 0.713 0.701
2c
7.5 7.0 6.5 6.0 5.5 5.0 4.5 4.0 3.5 3.0 2.5 2.0 1.5 1.0 0.5 ppm
2.043 2.032 2.030 1.061 2.044 1.021 2.035 2.021 3.082 3.064 3.035

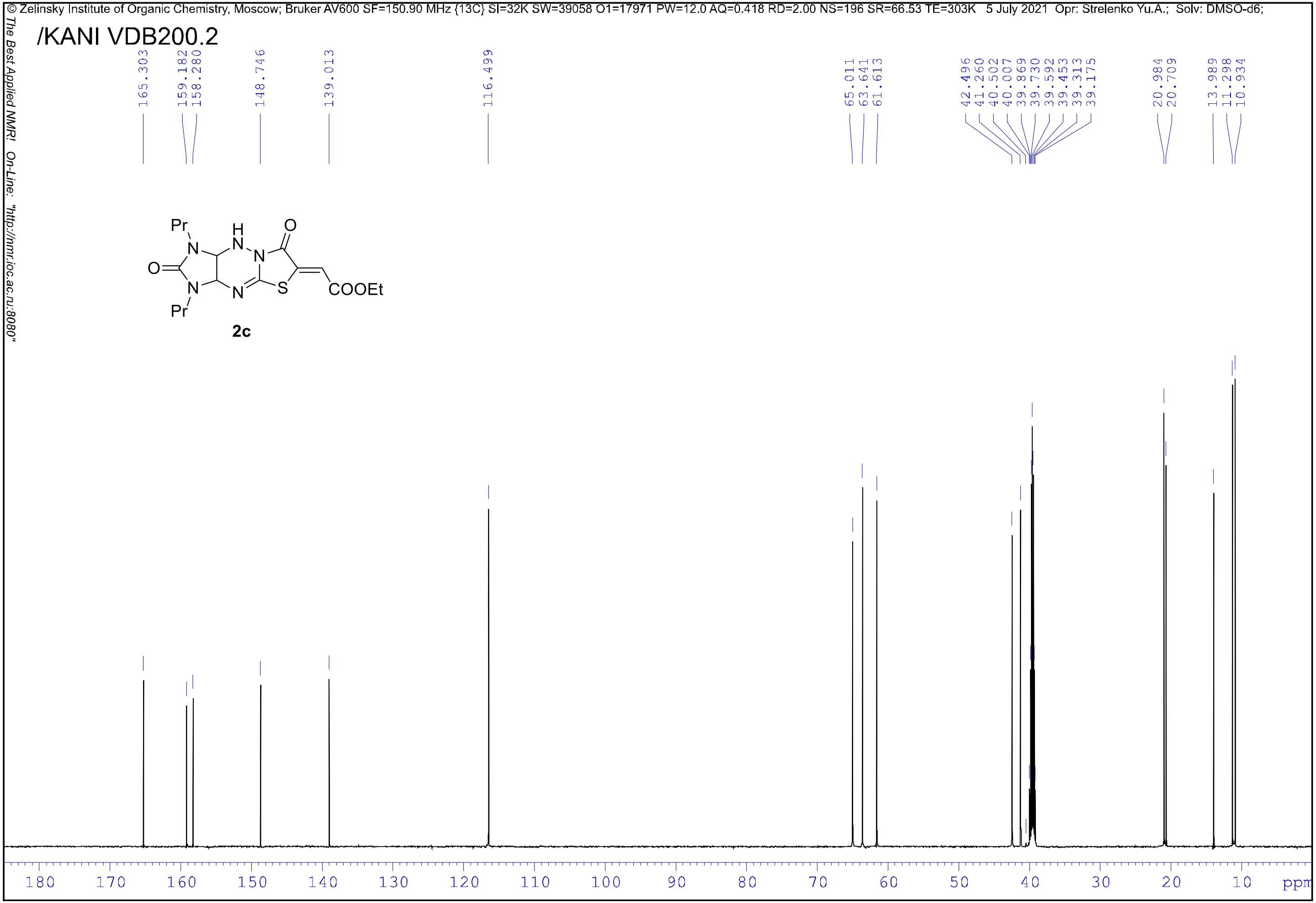
© Zelinsky Institute of Organic Chemistry, Moscow; Bruker AV600 SF=150.90 MHz {13C} SI=32K SW=39058 O1=17971 PW=12.0 AQ=0.418 RD=2.00 NS=196 SR=66.53 TE=303K 5 July 2021 Opr: Strelenko Yu.A.; Solv: DMSO-d6;
The Best Applied NMR! On-Line: "http://nmr.ioc.ac.ru:8080"
/KANI VDB200.2
165.303
159.182
158.280
148.746
139.013
116.499
65.011
63.641
61.613
42.496
41.260
40.502
40.007
39.869
39.730
39.592
39.453
39.313
39.175
20.984
20.709
13.989
11.298
10.934
Pr
H
O
N
N
N
O
N
N
S
COOEt
Pr
2c
180 170 160 150 140 130 120 110 100 90 80 70 60 50 40 30 20 10 ppm

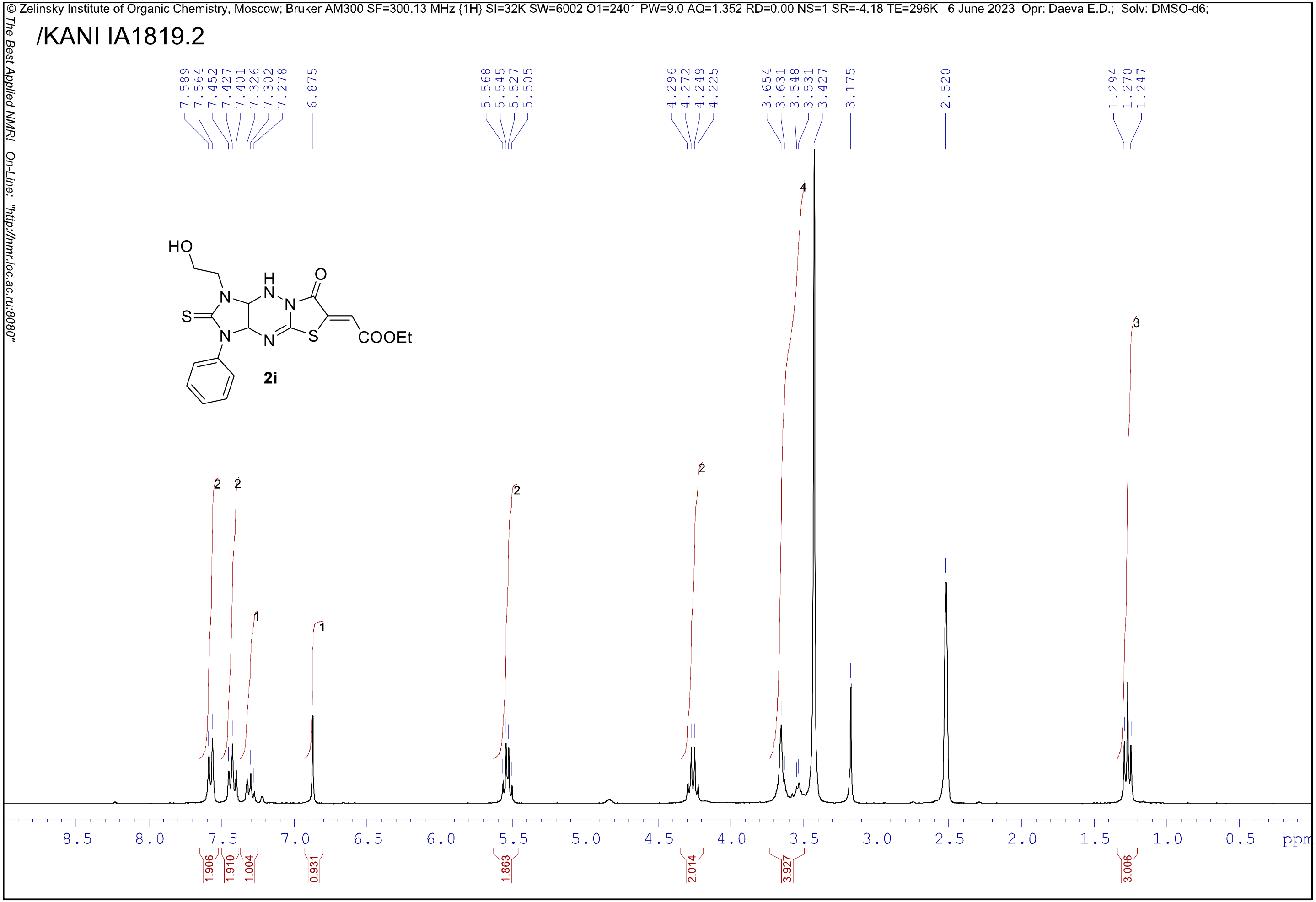
© Zelinsky Institute of Organic Chemistry, Moscow; Bruker AM300 SF=300.13 MHz {1H} SI=32K SW=6002 O1=2401 PW=9.0 AQ=1.352 RD=0.00 NS=1 SR=-4.18 TE=296K 6 June 2023 Opr: Daeva E.D.; Solv: DMSO-d6;
The Best Applied NMR! On-Line: "http://nmr.ioc.ac.ru:8080"
/KANI IA1819.2
7.589 7.564 7.452 7.427 7.401 7.326 7.302 7.278 6.875 5.568 5.545 5.527 5.505 4.296 4.272 4.249 4.225 3.654 3.631 3.548 3.531 3.427 3.175 2.520 1.294 1.270 1.247
HO
H
N
O
S
COOEt
2i
8.5 8.0 7.5 7.0 6.5 6.0 5.5 5.0 4.5 4.0 3.5 3.0 2.5 2.0 1.5 1.0 0.5 ppm
1.906 1.910 1.004 0.931 1.863 2.014 3.927 3.006

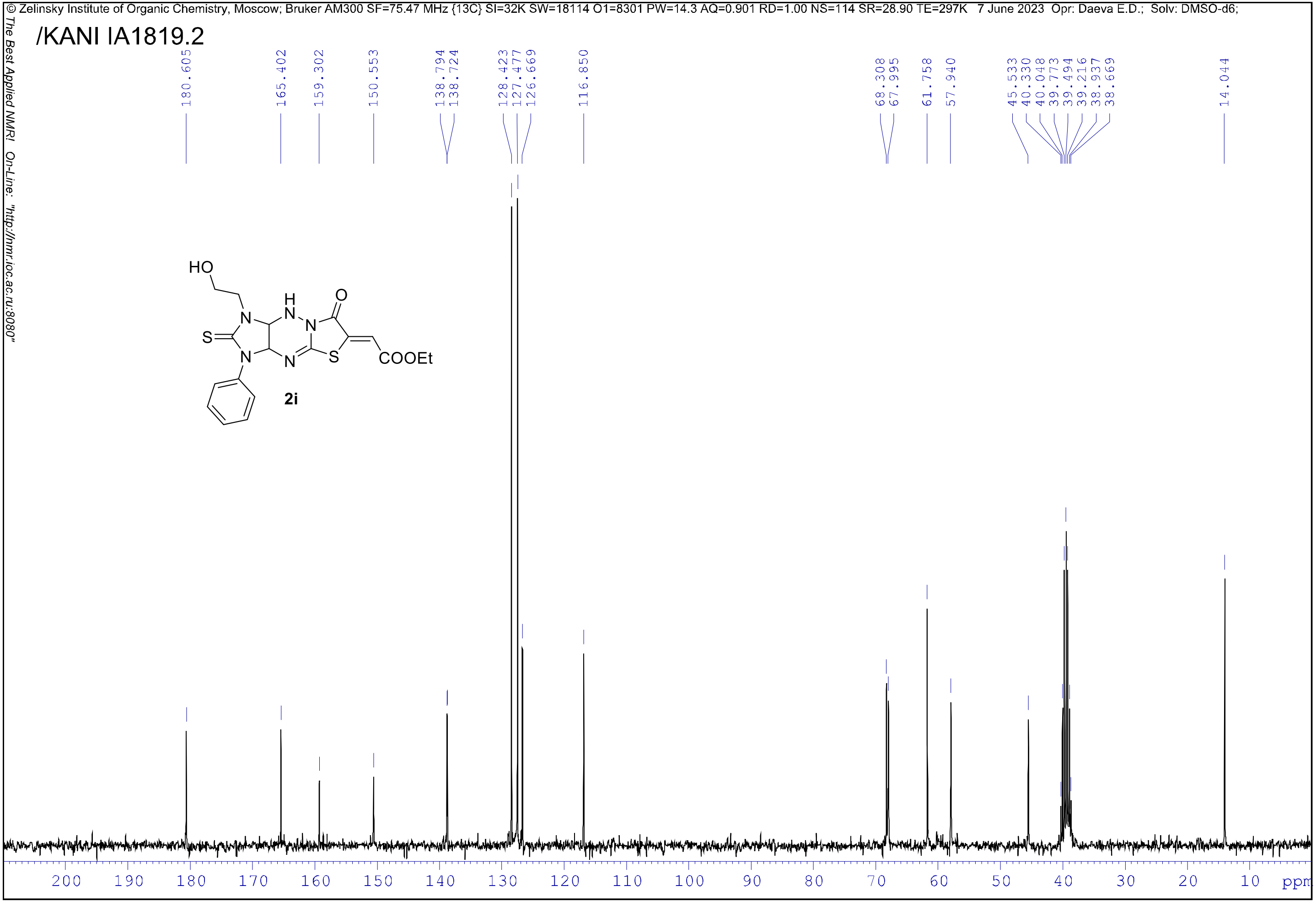
© Zelinsky Institute of Organic Chemistry, Moscow; Bruker AM300 SF=75.47 MHz {13C} SI=32K SW=18114 O1=8301 PW=14.3 AQ=0.901 RD=1.00 NS=114 SR=28.90 TE=297K 7 June 2023 Opr: Daeva E.D.; Solv: DMSO-d6;
The Best Applied NMR! On-Line: "http://nmr.ioc.ac.ru:8080"
/KANI IA1819.2
180.605
165.402
159.302
150.553
138.794
138.724
128.423
127.477
126.669
116.850
68.308
67.995
61.758
57.940
45.533
40.330
40.048
39.773
39.494
39.216
38.937
38.669
14.044
HO
H
N
N
O
S
N
N
S
COOEt
2i
200 190 180 170 160 150 140 130 120 110 100 90 80 70 60 50 40 30 20 10 ppm

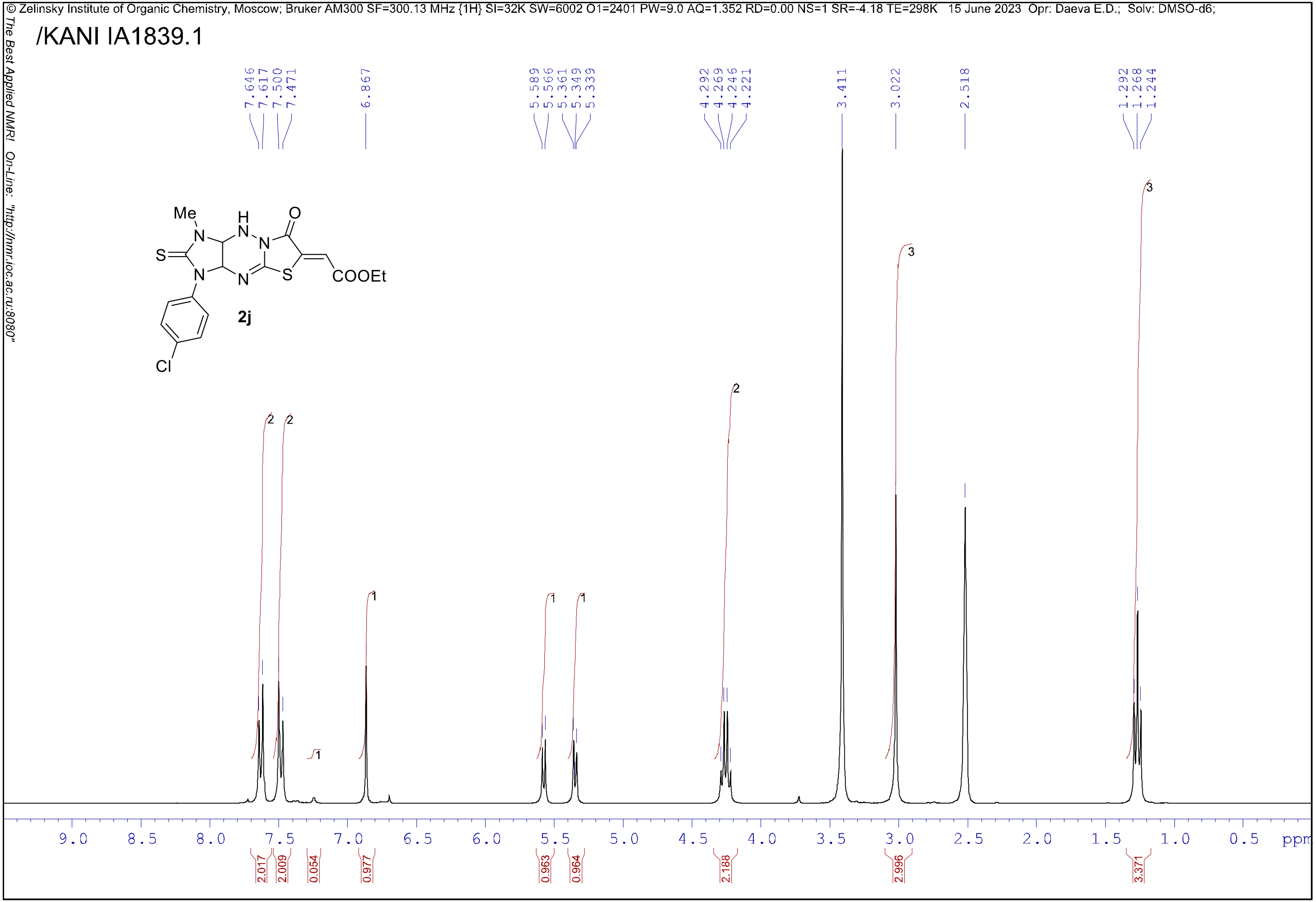
© Zelinsky Institute of Organic Chemistry, Moscow; Bruker AM300 SF=300.13 MHz {1H} SI=32K SW=6002 O1=2401 PW=9.0 AQ=1.352 RD=0.00 NS=1 SR=-4.18 TE=298K 15 June 2023 Opr: Daeva E.D.; Solv: DMSO-d6;
The Best Applied NMR! On-Line: "http://nmr.ioc.ac.ru:8080"
/KANI IA1839.1
7.646
7.617
7.500
7.471
6.867
5.589
5.566
5.361
5.349
5.339
4.292
4.269
4.246
4.221
3.411
3.022
2.518
1.292
1.268
1.244
Me
H
O
S
N
COOEt
Cl
2j
9.0
8.5
8.0
7.5
7.0
6.5
6.0
5.5
5.0
4.5
4.0
3.5
3.0
2.5
2.0
1.5
1.0
0.5
ppm
2.017
2.009
0.054
0.977
0.963
0.964
2.188
2.996
3.371

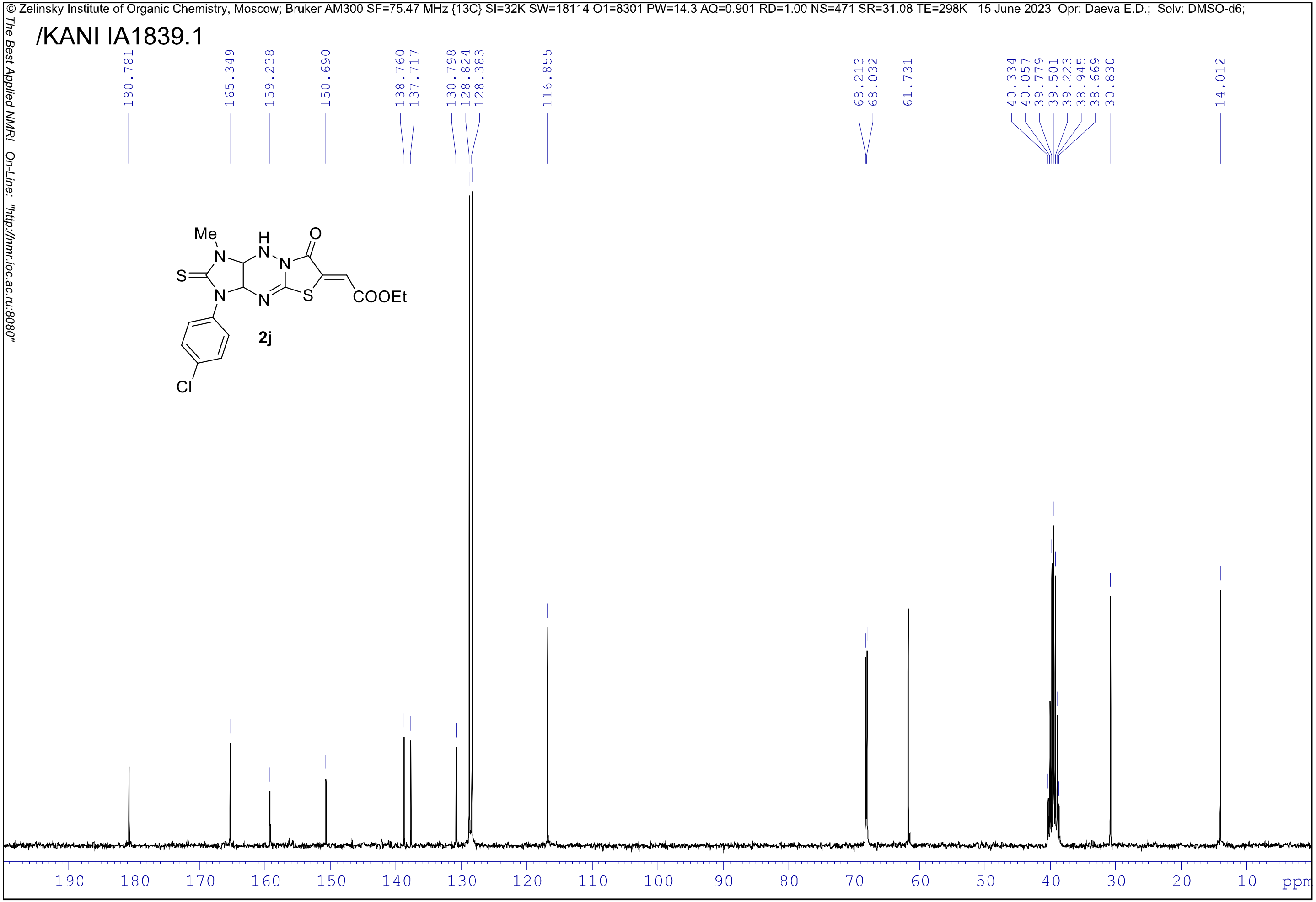
© Zelinsky Institute of Organic Chemistry, Moscow; Bruker AM300 SF=75.47 MHz {13C} SI=32K SW=18114 O1=8301 PW=14.3 AQ=0.901 RD=1.00 NS=471 SR=31.08 TE=298K 15 June 2023 Opr: Daeva E.D.; Solv: DMSO-d6;
The Best Applied NMR! On-Line: "http://nmr.ioc.ac.ru:8080"
/KANI IA1839.1
180.781
165.349
159.238
150.690
138.760
137.717
130.798
128.824
128.383
116.855
68.213
68.032
61.731
40.334
40.057
39.779
39.501
39.223
38.945
38.669
30.830
14.012
Me
H
N
O
S
COOEt
Cl
2j
190 180 170 160 150 140 130 120 110 100 90 80 70 60 50 40 30 20 10 ppm

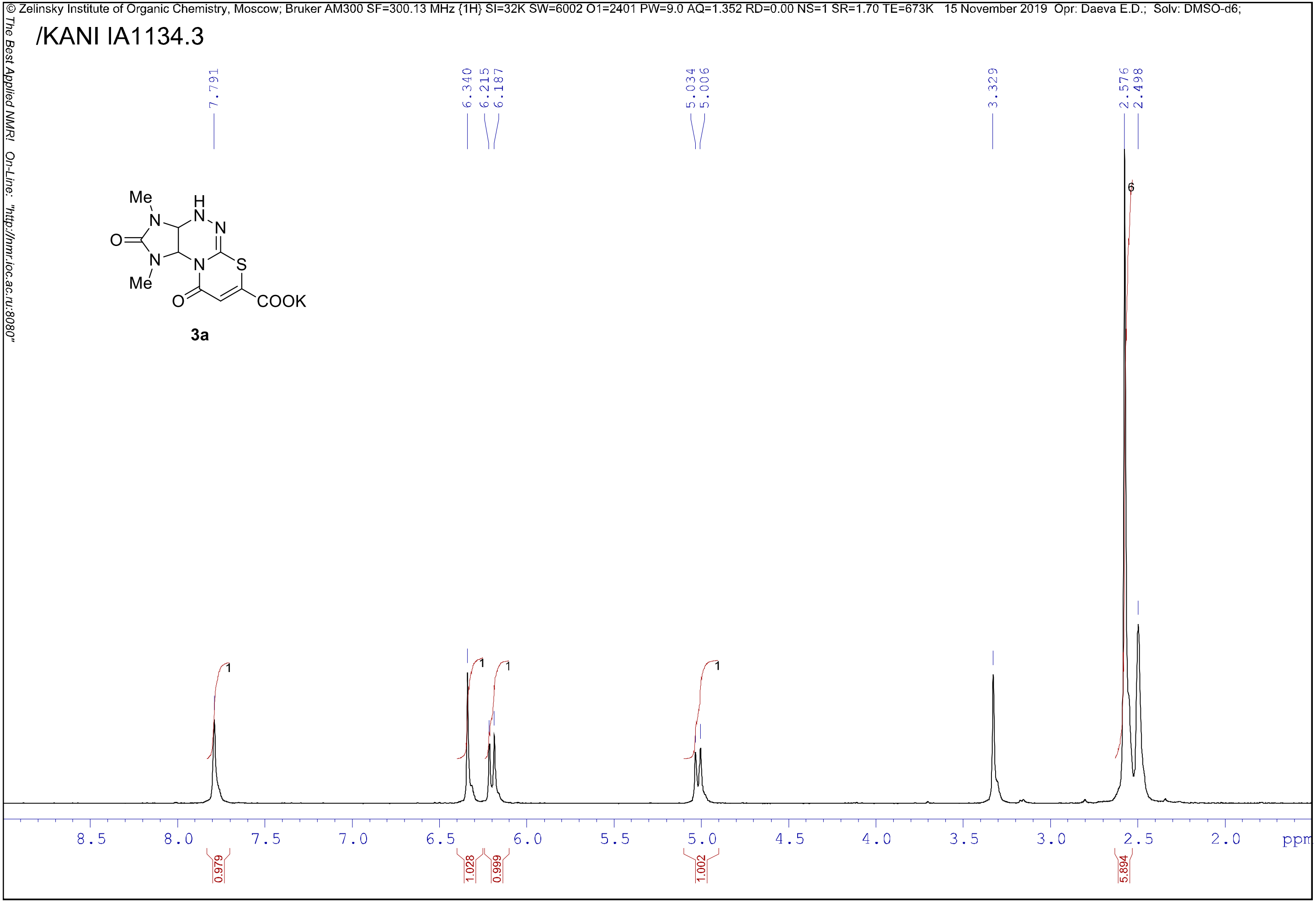
© Zelinsky Institute of Organic Chemistry, Moscow; Bruker AM300 SF=300.13 MHz {1H} SI=32K SW=6002 O1=2401 PW=9.0 AQ=1.352 RD=0.00 NS=1 SR=1.70 TE=673K 15 November 2019 Opr: Daeva E.D.; Solv: DMSO-d6;
The Best Applied NMR! On-Line: "http://nmr.ioc.ac.ru:8080"
/KANI IA1134.3
7.791
6.340
6.215
6.187
5.034
5.006
3.329
2.576
2.498
Me
H
N
N
N
O
N
N
S
Me
O
COOK
3a
0.979
1.028
0.999
1.002
5.894
ppm

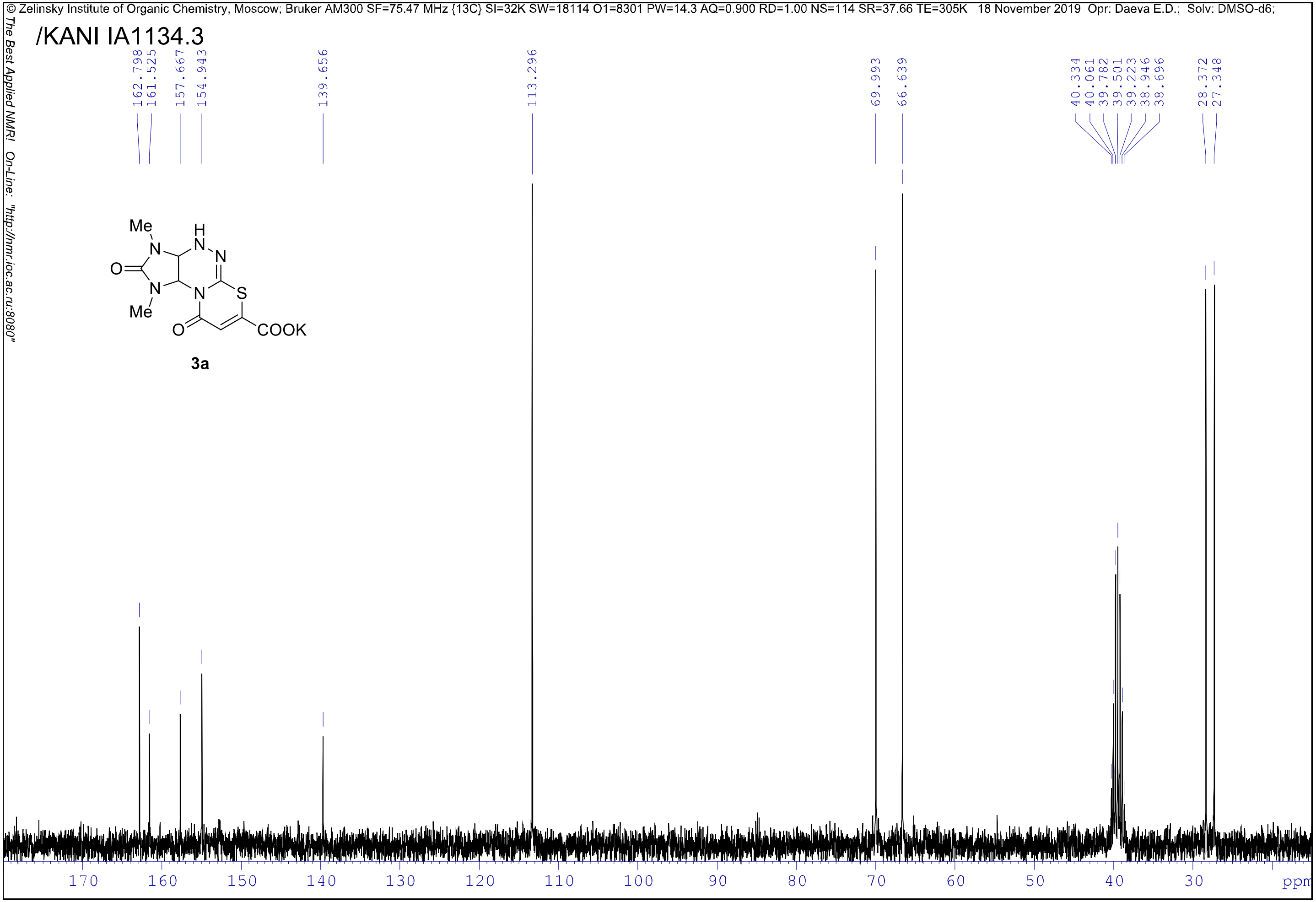
© Zelinsky Institute of Organic Chemistry, Moscow; Bruker AM300 SF=75.47 MHz {13C} SI=32K SW=18114 O1=8301 PW=14.3 AQ=0.900 RD=1.00 NS=114 SR=37.66 TE=305K 18 November 2019 Opr: Daeva E.D.; Solv: DMSO-d6;
The Best Applied NMR! On-Line: "http://nmr.ioc.ac.ru:8080"
/KANI IA1134.3
162.798
161.525
157.667
154.943
139.656
113.296
69.993
66.639
40.334
40.061
39.782
39.501
39.223
38.946
38.696
28.372
27.348
Me
H
N
N
N
O
N
N
S
Me
O
COOK
3a
170
160
150
140
130
120
110
100
90
80
70
60
50
40
30
ppm

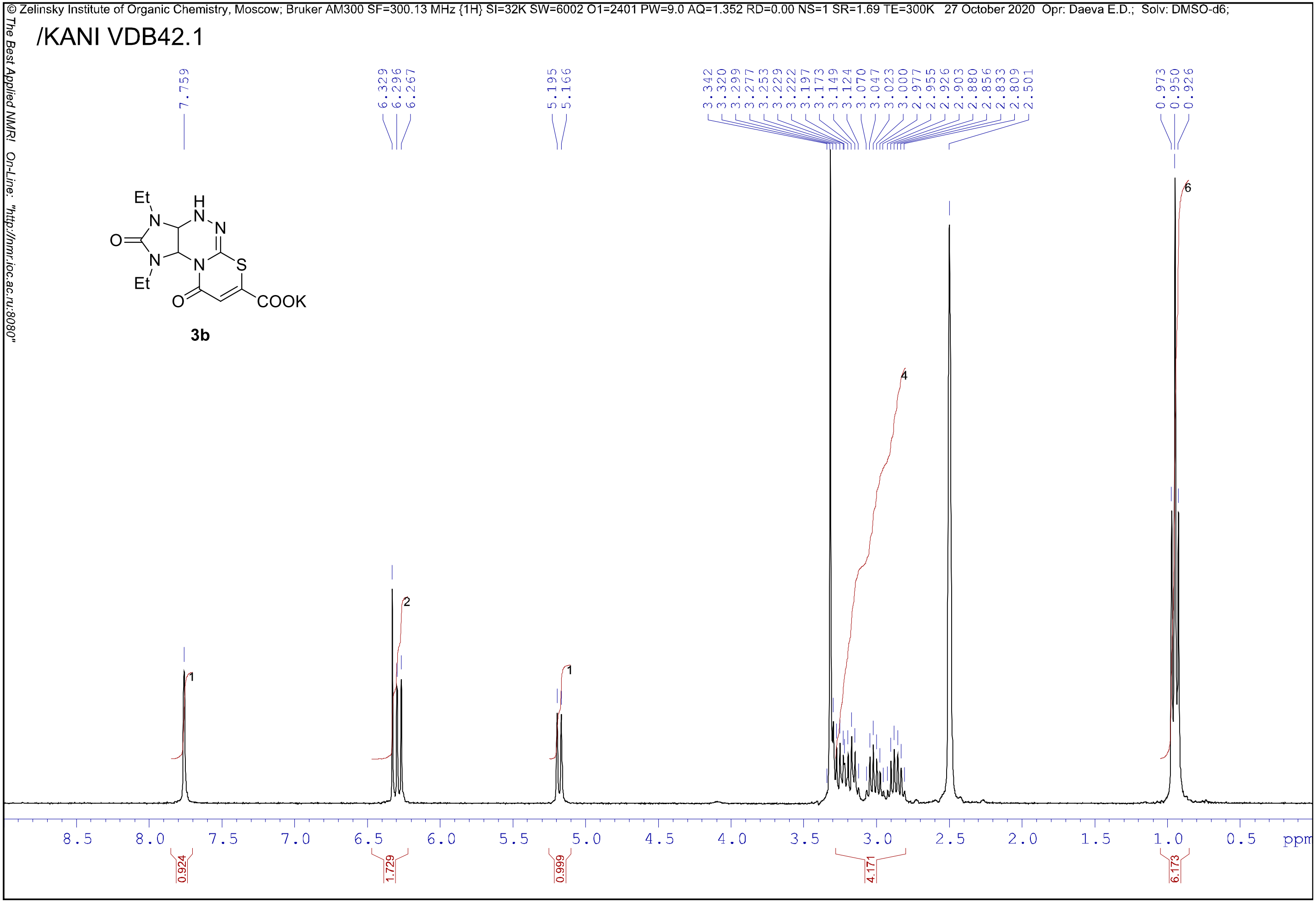
© Zelinsky Institute of Organic Chemistry, Moscow; Bruker AM300 SF=300.13 MHz {1H} SI=32K SW=6002 O1=2401 PW=9.0 AQ=1.352 RD=0.00 NS=1 SR=1.69 TE=300K 27 October 2020 Opr: Daeva E.D.; Solv: DMSO-d6;
/KANI VDB42.1
The Best Applied NMR! On-Line: "http://nmr.ioc.ac.ru:8080"
7.759
6.329
6.296
6.267
5.195
5.166
3.342
3.320
3.299
3.277
3.253
3.229
3.222
3.197
3.173
3.149
3.124
3.070
3.047
3.023
3.000
2.977
2.955
2.926
2.903
2.880
2.856
2.833
2.809
2.501
0.973
0.950
0.926
Et
H
N
N
N
O
N
N
S
Et
O
COOK
3b
1
2
1
4
6
8.5
8.0
7.5
7.0
6.5
6.0
5.5
5.0
4.5
4.0
3.5
3.0
2.5
2.0
1.5
1.0
0.5
ppm
0.924
1.729
0.999
4.171
6.173

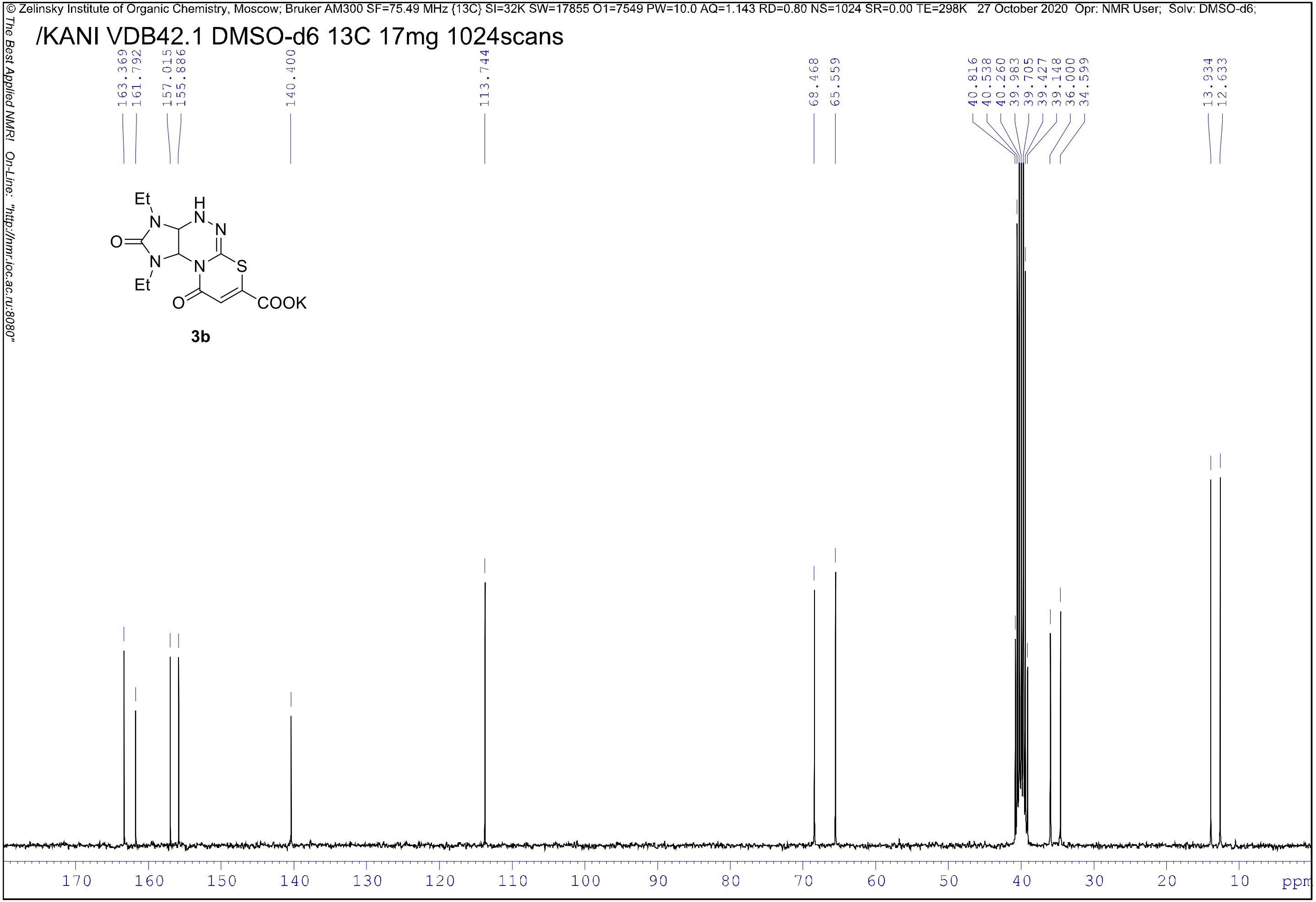
© Zelinsky Institute of Organic Chemistry, Moscow; Bruker AM300 SF=75.49 MHz {13C} SI=32K SW=17855 O1=7549 PW=10.0 AQ=1.143 RD=0.80 NS=1024 SR=0.00 TE=298K 27 October 2020 Opr: NMR User; Solv: DMSO-d6;
The Best Applied NMR! On-Line: "http://nmr.ioc.ac.ru:8080"
/KANI VDB42.1 DMSO-d6 13C 17mg 1024scans
163.369
161.792
157.015
155.886
140.400
113.744
68.468
65.559
40.816
40.538
40.260
39.983
39.705
39.427
39.148
36.000
34.599
13.934
12.633
Et
H
N
N
N
O
N
N
S
Et
O
COOK
3b
170
160
150
140
130
120
110
100
90
80
70
60
50
40
30
20
10
ppm

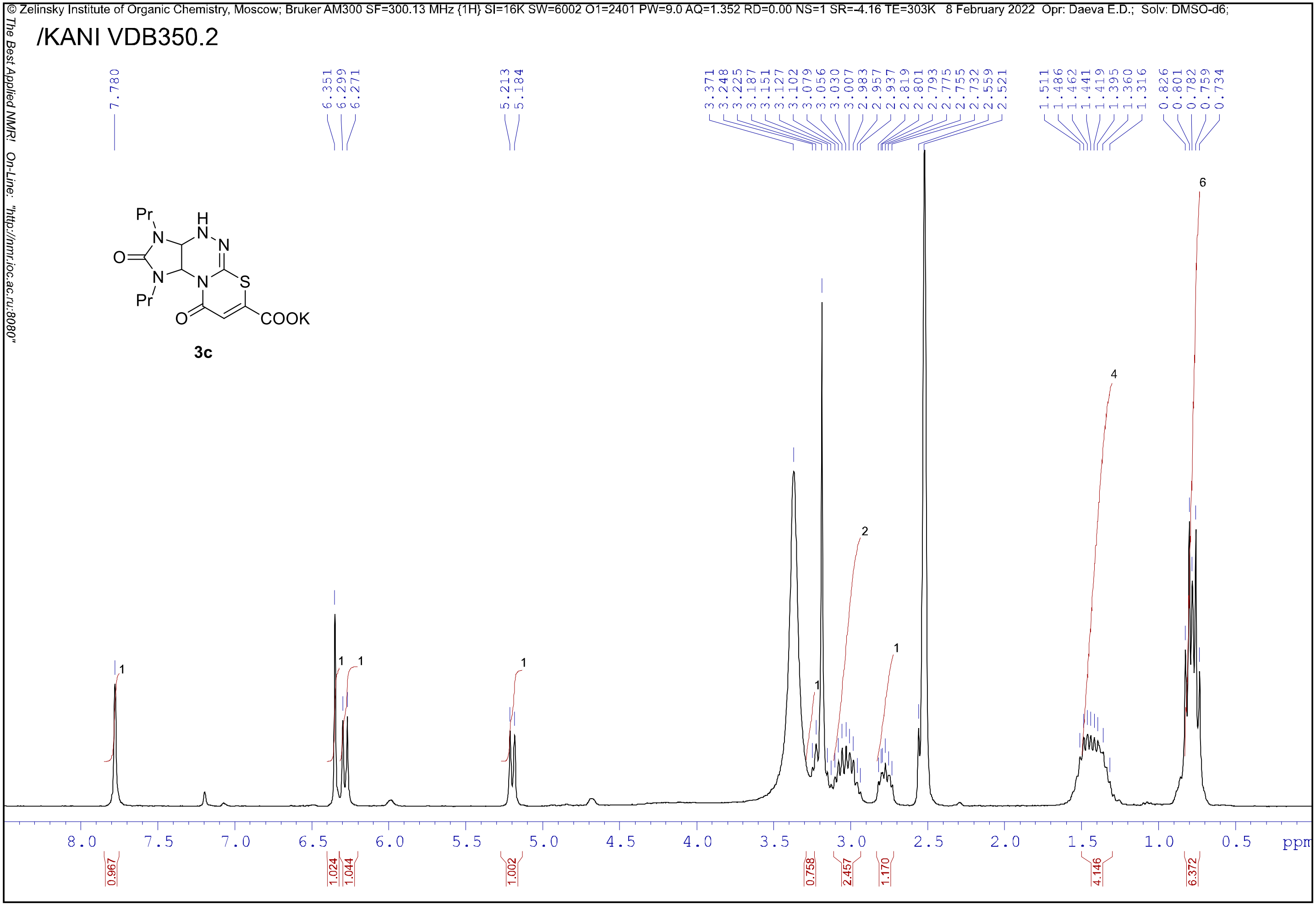
© Zelinsky Institute of Organic Chemistry, Moscow; Bruker AM300 SF=300.13 MHz {1H} SI=16K SW=6002 O1=2401 PW=9.0 AQ=1.352 RD=0.00 NS=1 SR=-4.16 TE=303K 8 February 2022 Opr: Daeva E.D.; Solv: DMSO-d6;
/KANI VDB350.2
The Best Applied NMR! On-Line: "http://nmr.ioc.ac.ru:8080"
7.780
6.351 6.299 6.271
5.213 5.184
3.371 3.248 3.225 3.187 3.151 3.127 3.102 3.079 3.056 3.030 3.007 2.983 2.957 2.937 2.819 2.801 2.793 2.775 2.755 2.732 2.559 2.521
1.511 1.486 1.462 1.441 1.419 1.395 1.360 1.316
0.826 0.801 0.782 0.759 0.734
Pr
H
N
N
N
O
N
N
S
Pr
O
COOK
3c
8.0 7.5 7.0 6.5 6.0 5.5 5.0 4.5 4.0 3.5 3.0 2.5 2.0 1.5 1.0 0.5 ppm
0.967 1.024 1.044 1.002 0.758 2.457 1.170 4.146 6.372

© Zelinsky Institute of Organic Chemistry, Moscow; Bruker AM300 SF=75.47 MHz {13C} SI=32K SW=18114 O1=8301 PW=14.3 AQ=0.901 RD=1.00 NS=1023 SR=39.27 TE=303K 9 February 2022 Opr: Daeva E.D.; Solv: DMSO-d6;

The Best Applied NMR! On-Line: "http://nmr.ioc.ac.ru:8080"

/KANI VDB350.2

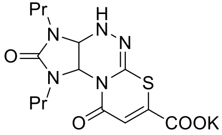

3c

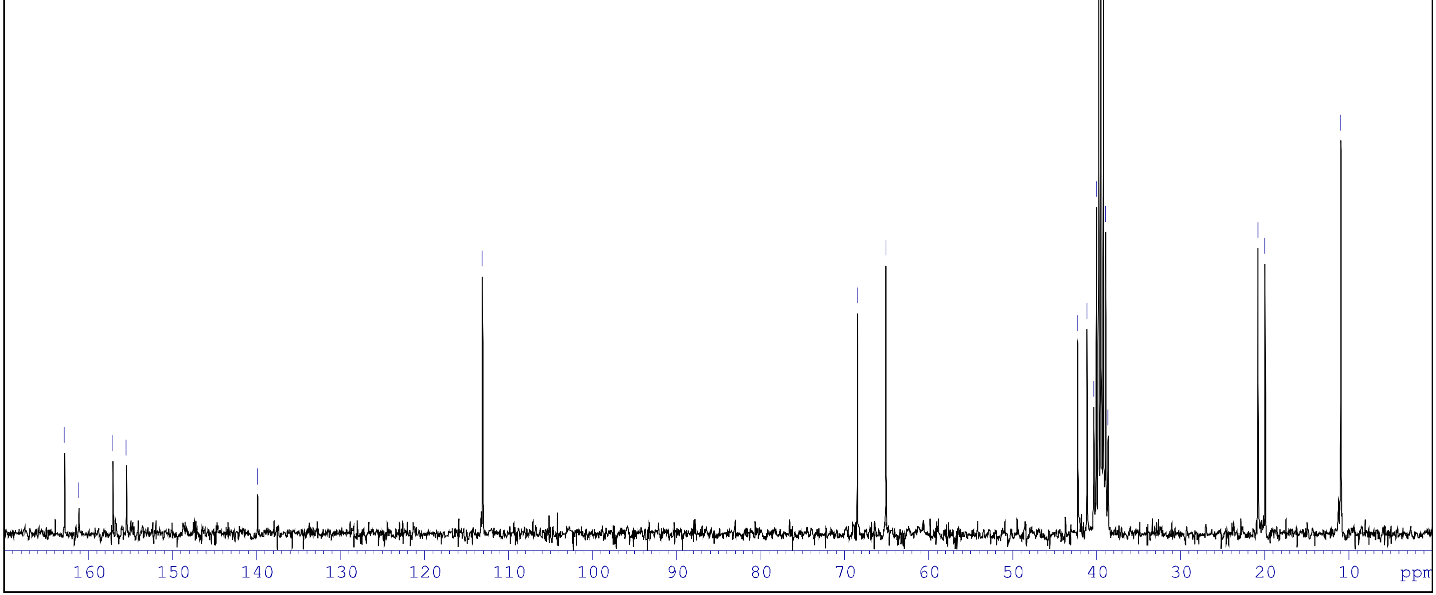

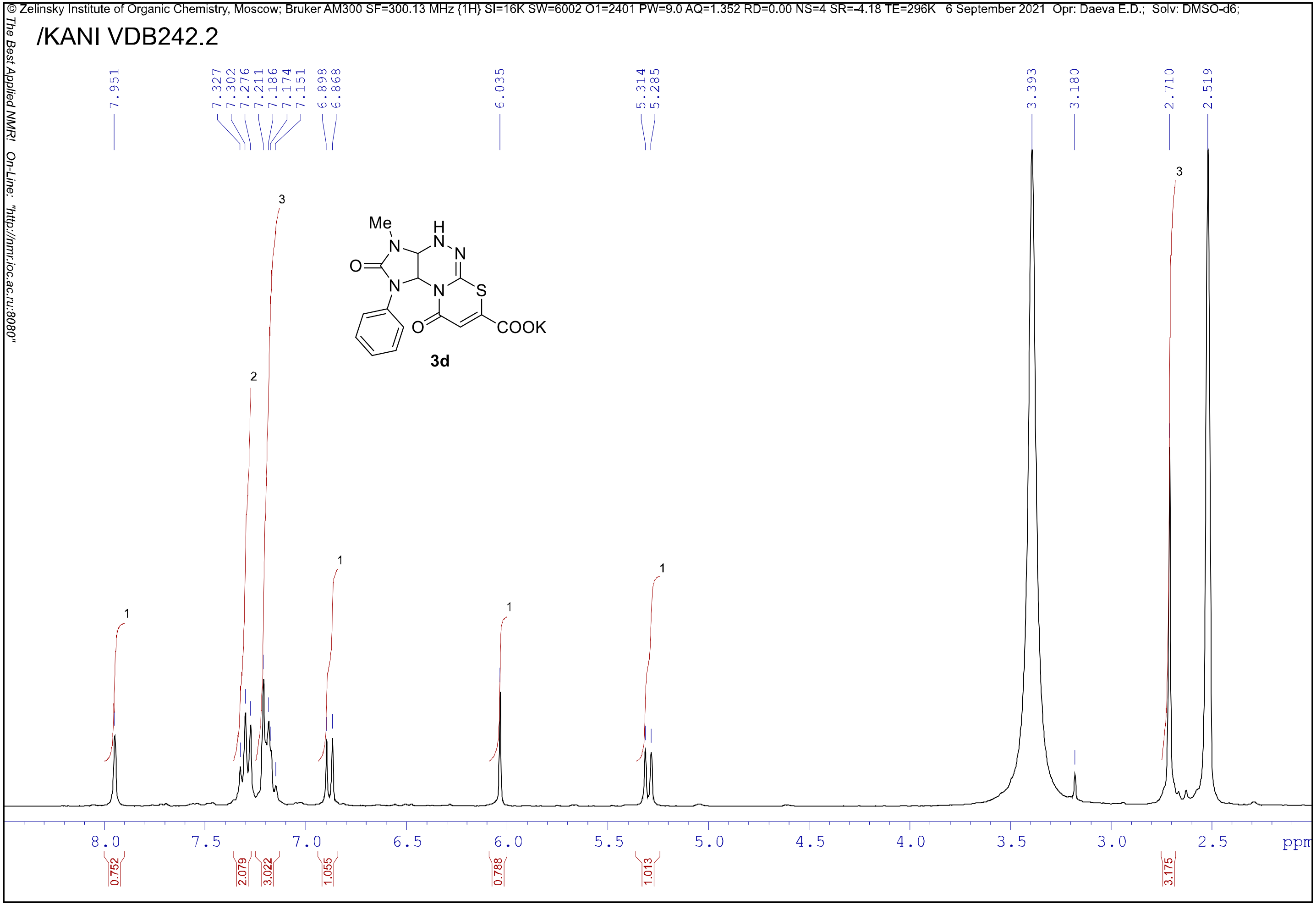
© Zelinsky Institute of Organic Chemistry, Moscow; Bruker AM300 SF=300.13 MHz {1H} SI=16K SW=6002 O1=2401 PW=9.0 AQ=1.352 RD=0.00 NS=4 SR=-4.18 TE=296K 6 September 2021 Opr: Daeva E.D.; Solv: DMSO-d6;
The Best Applied NMR! On-Line: "http://nmr.ioc.ac.ru:8080"
/KANI VDB242.2
7.951
7.327
7.302
7.276
7.211
7.186
7.174
7.151
6.898
6.868
6.035
5.314
5.285
3.393
3.180
2.710
2.519
Me
H
N
N
N
O
N
N
S
O
COOK
3d
8.0
7.5
7.0
6.5
6.0
5.5
5.0
4.5
4.0
3.5
3.0
2.5
ppm
0.752
2.079
3.022
1.055
0.788
1.013
3.175
1
2
3
1
1
1
3

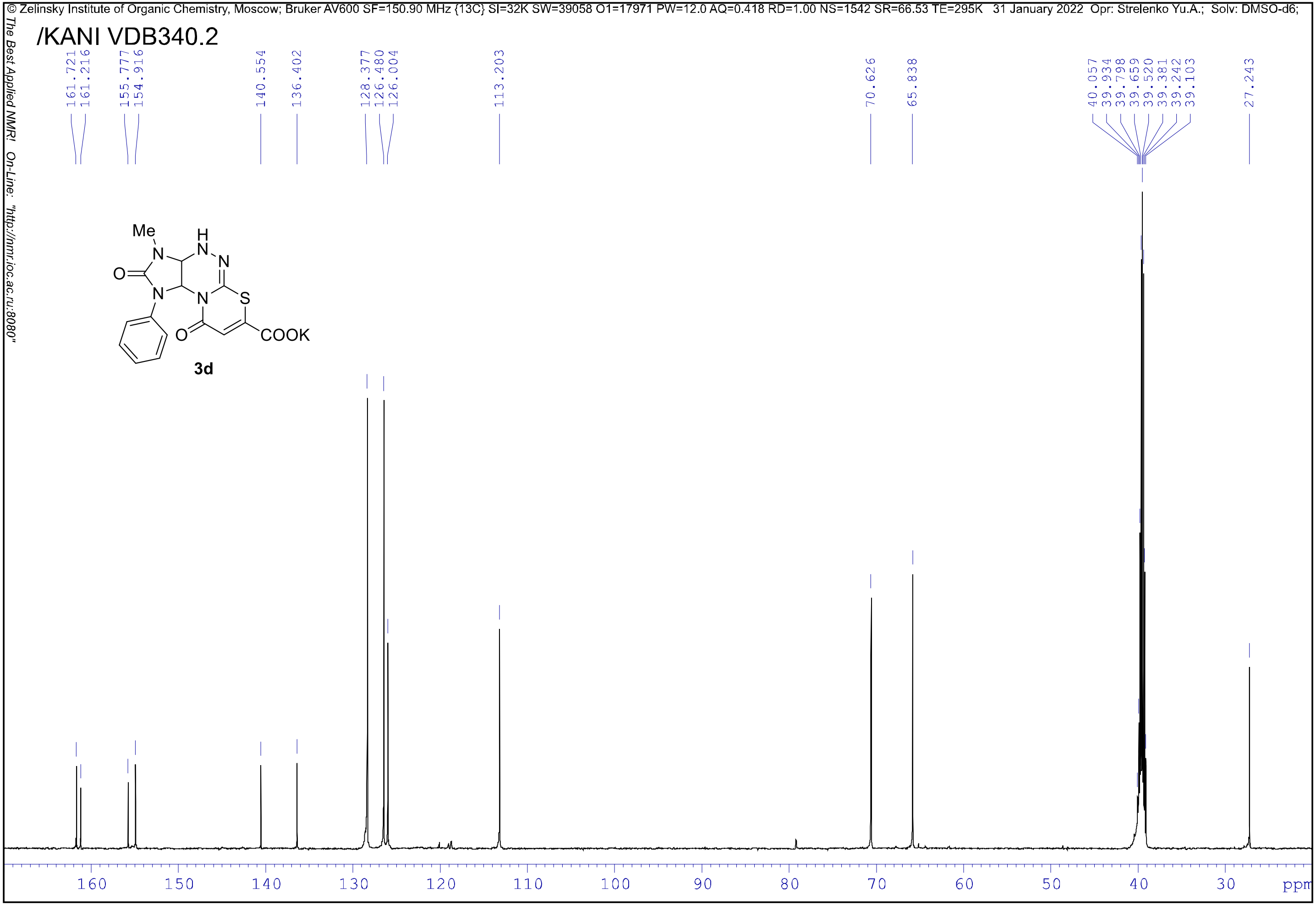
© Zelinsky Institute of Organic Chemistry, Moscow; Bruker AV600 SF=150.90 MHz {13C} SI=32K SW=39058 O1=17971 PW=12.0 AQ=0.418 RD=1.00 NS=1542 SR=66.53 TE=295K 31 January 2022 Opr: Strelenko Yu.A.; Solv: DMSO-d6;
The Best Applied NMR! On-Line: "http://nmr.ioc.ac.ru:8080"
/KANI VDB340.2
161.721
161.216
155.777
154.916
140.554
136.402
128.377
126.480
126.004
113.203
70.626
65.838
40.057
39.934
39.798
39.659
39.520
39.381
39.242
39.103
27.243
Me
H
N
O
S
COOK
3d
160
150
140
130
120
110
100
90
80
70
60
50
40
30
ppm

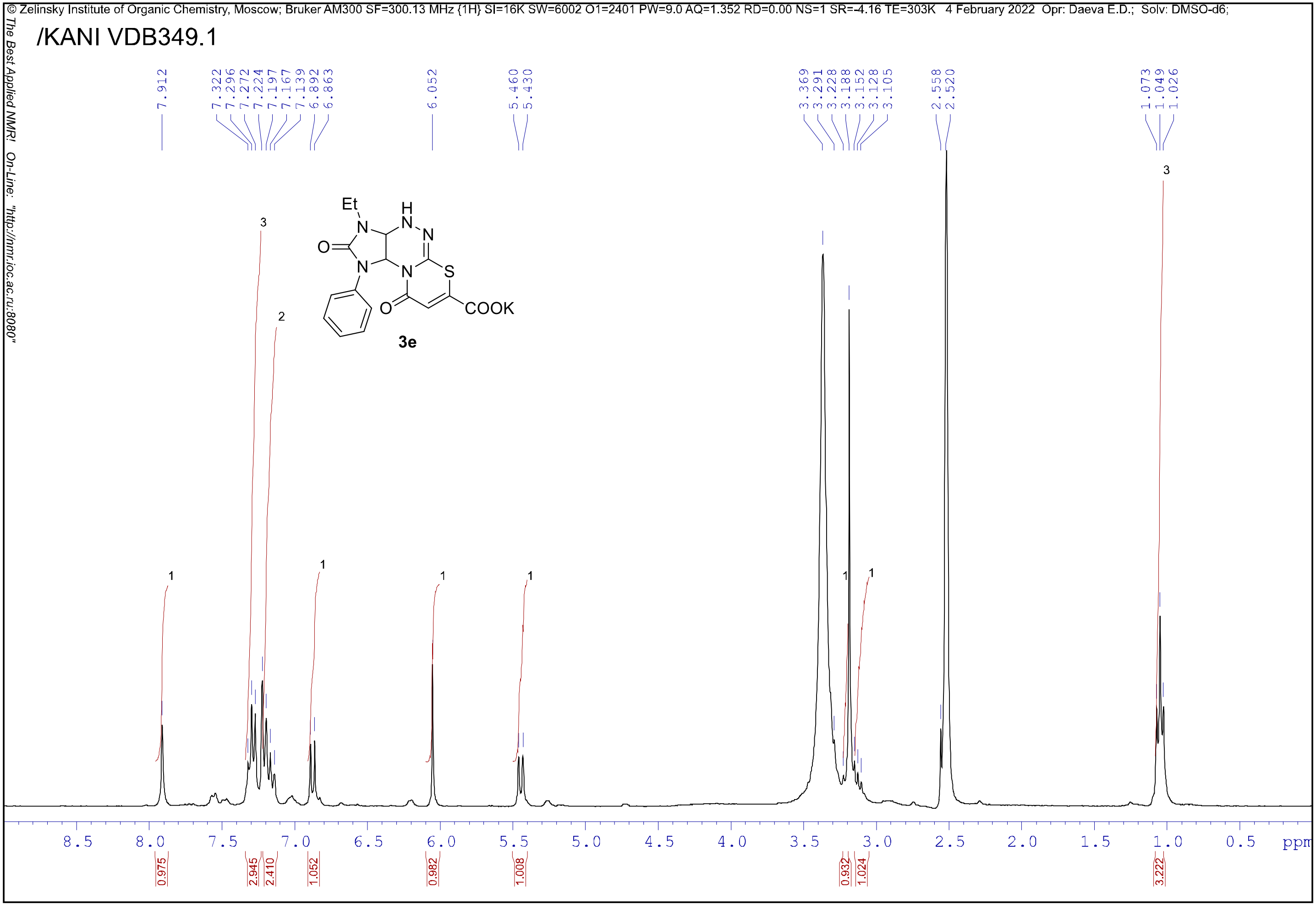
© Zelinsky Institute of Organic Chemistry, Moscow; Bruker AM300 SF=300.13 MHz {1H} SI=16K SW=6002 O1=2401 PW=9.0 AQ=1.352 RD=0.00 NS=1 SR=-4.16 TE=303K 4 February 2022 Opr: Daeva E.D.; Solv: DMSO-d6;
The Best Applied NMR! On-Line: "http://nmr.ioc.ac.ru:8080"
/KANI VDB349.1
7.912
7.322
7.296
7.272
7.224
7.197
7.167
7.139
6.892
6.863
6.052
5.460
5.430
3.369
3.291
3.228
3.188
3.152
3.128
3.105
2.558
2.520
1.073
1.049
1.026
Et
H
N
O
S
COOK
3e
ppm
0.975
2.945
2.410
1.052
0.982
1.008
0.932
1.024
3.222

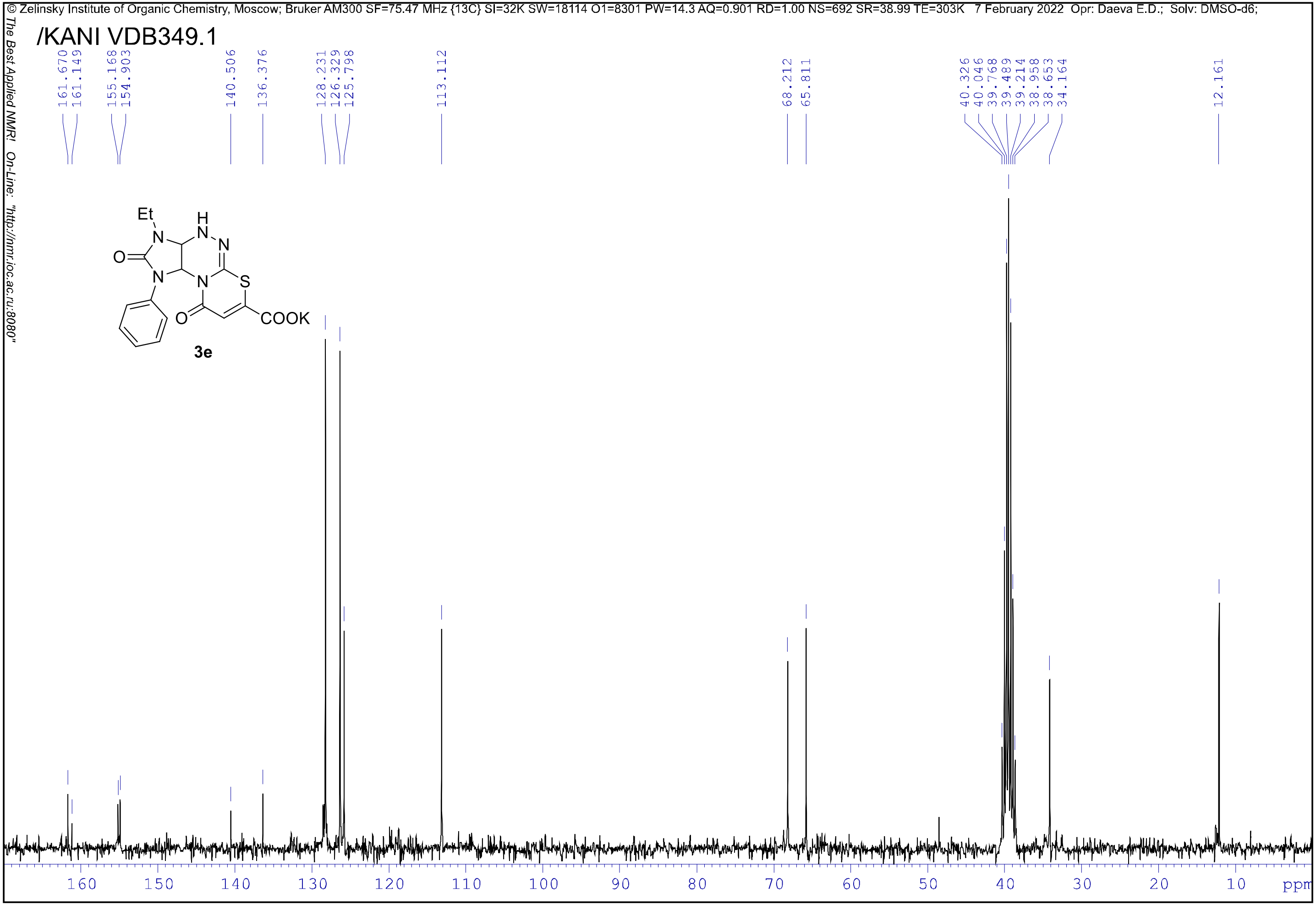
© Zelinsky Institute of Organic Chemistry, Moscow; Bruker AM300 SF=75.47 MHz {13C} SI=32K SW=18114 O1=8301 PW=14.3 AQ=0.901 RD=1.00 NS=692 SR=38.99 TE=303K 7 February 2022 Opr: Daeva E.D.; Solv: DMSO-d6;
/KANI VDB349.1
The Best Applied NMR! On-Line: "http://nmr.ioc.ac.ru:8080"
161.670
161.149
155.168
154.903
140.506
136.376
128.231
126.329
125.798
113.112
68.212
65.811
40.326
40.046
39.768
39.489
39.214
38.958
38.653
34.164
12.161
Et
H
N
N
N
O
N
N
S
O
COOK
3e
160
150
140
130
120
110
100
90
80
70
60
50
40
30
20
10
ppm

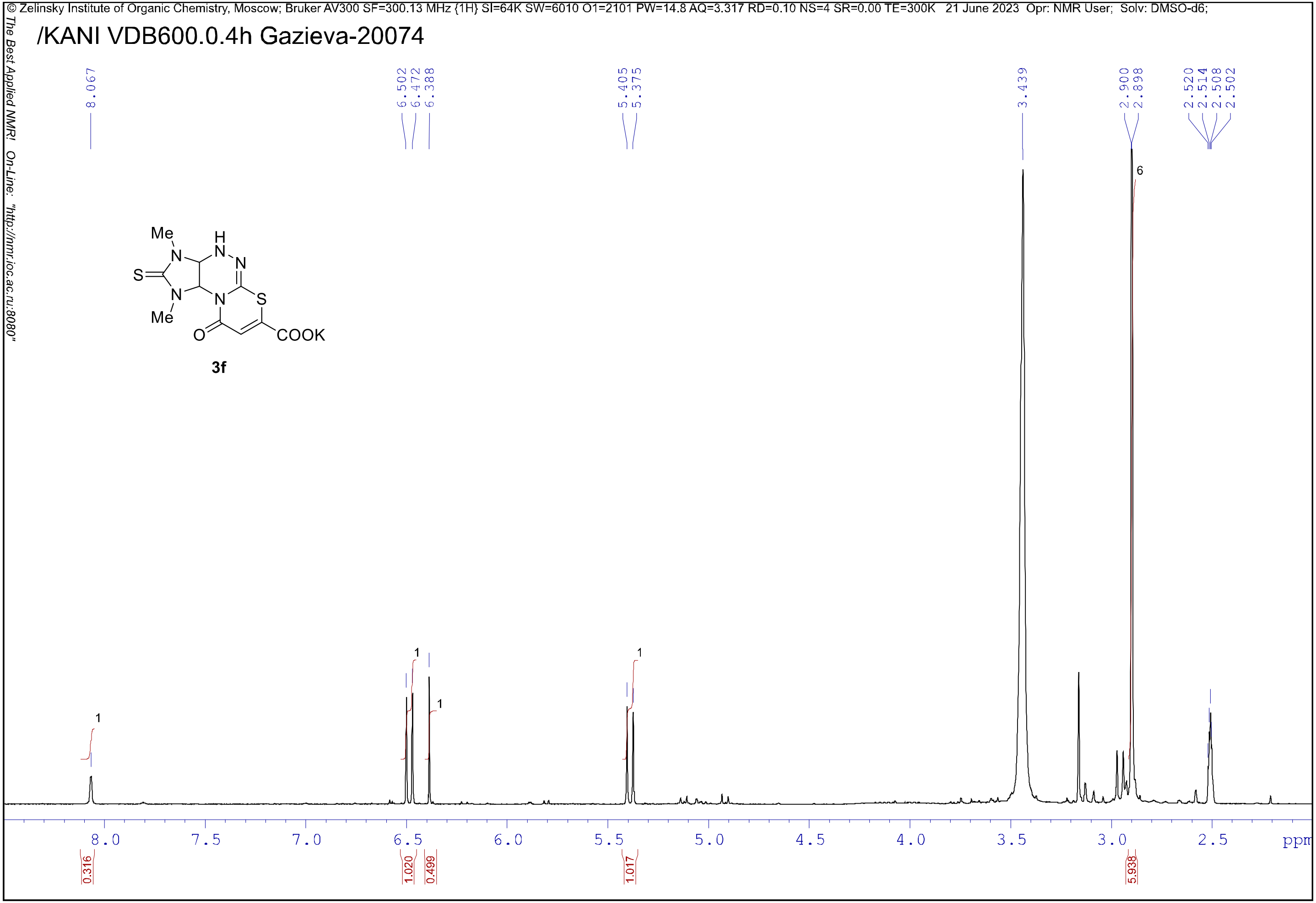
© Zelinsky Institute of Organic Chemistry, Moscow; Bruker AV300 SF=300.13 MHz {1H} SI=64K SW=6010 O1=2101 PW=14.8 AQ=3.317 RD=0.10 NS=4 SR=0.00 TE=300K 21 June 2023 Opr: NMR User; Solv: DMSO-d6;
The Best Applied NMR! On-Line: "http://nmr.ioc.ac.ru:8080"
/KANI VDB600.0.4h Gazieva-20074
8.067
6.502
6.472
6.388
5.405
5.375
3.439
2.900
2.898
2.520
2.514
2.508
2.502
3f
ppm
0.316
1.020
0.499
1.017
5.938

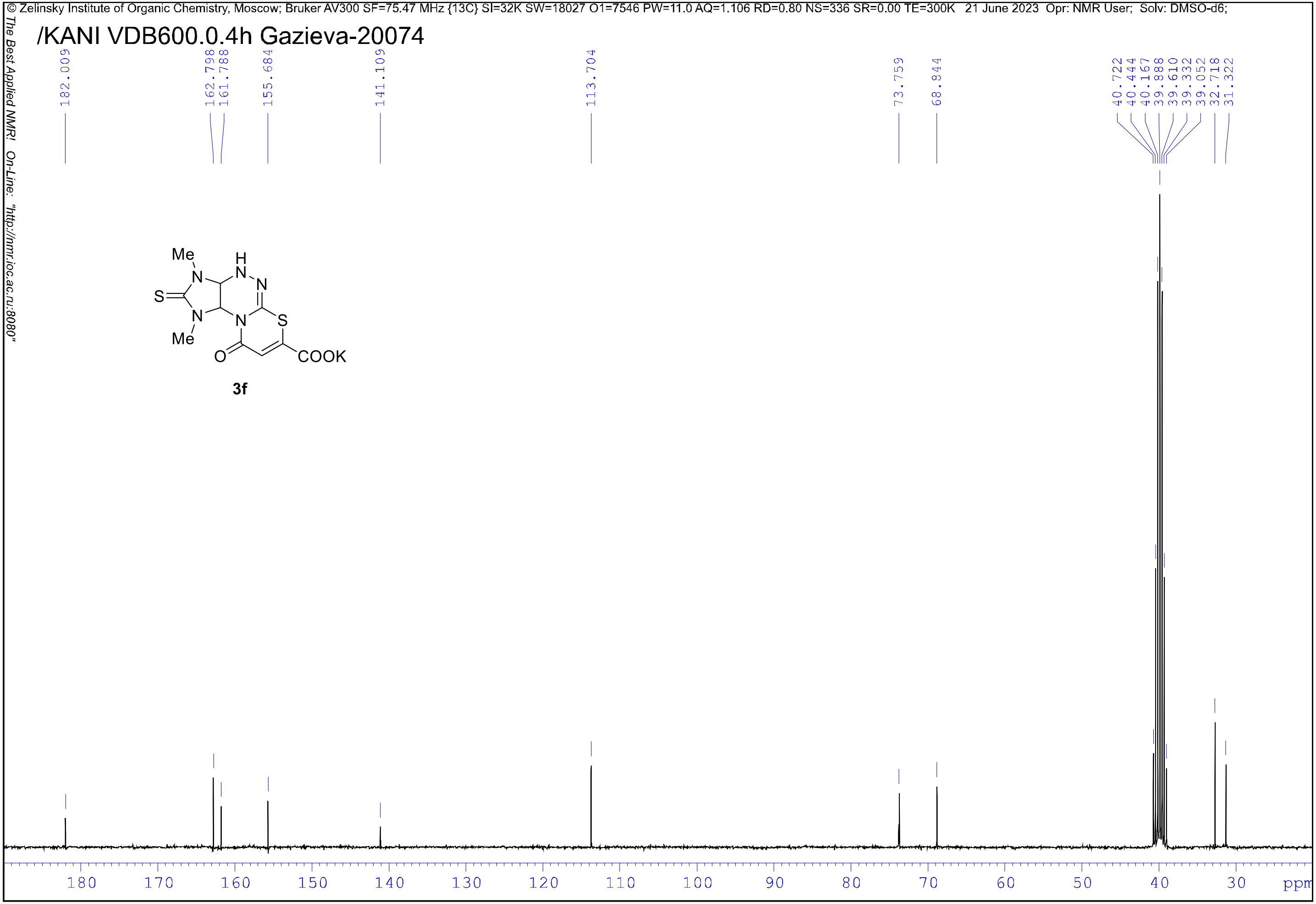
© Zelinsky Institute of Organic Chemistry, Moscow; Bruker AV300 SF=75.47 MHz {13C} SI=32K SW=18027 O1=7546 PW=11.0 AQ=1.106 RD=0.80 NS=336 SR=0.00 TE=300K 21 June 2023 Opr: NMR User; Solv: DMSO-d6;
The Best Applied NMR! On-Line: "http://nmr.ioc.ac.ru:8080"
/KANI VDB600.0.4h Gazieva-20074
182.009
162.798
161.788
155.684
141.109
113.704
73.759
68.844
40.722
40.444
40.167
39.888
39.610
39.332
39.052
32.718
31.322
Me
H
N
N
S
N
N
S
Me
O
COOK
3f
180
170
160
150
140
130
120
110
100
90
80
70
60
50
40
30
ppm

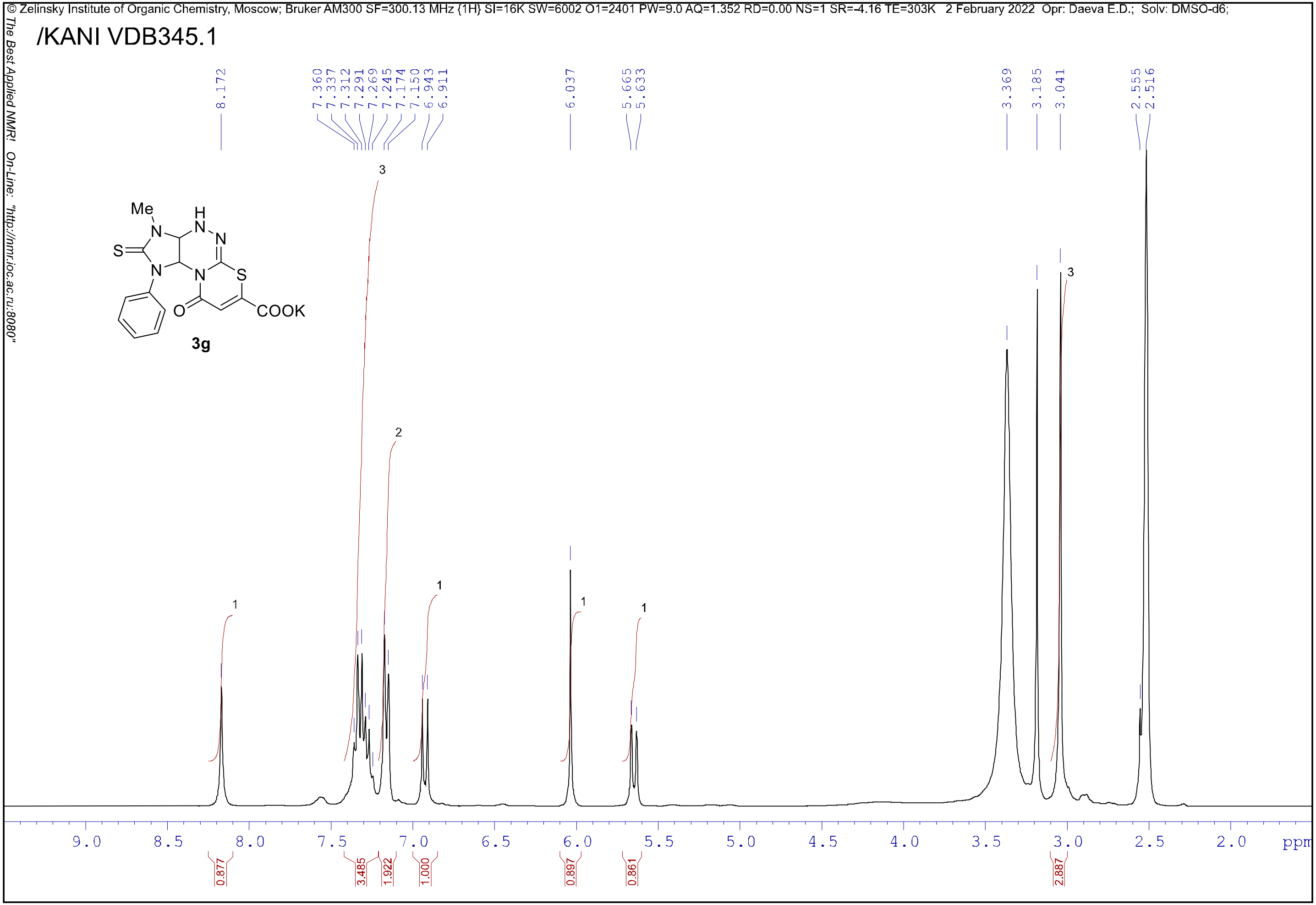
© Zelinsky Institute of Organic Chemistry, Moscow; Bruker AM300 SF=300.13 MHz {1H} SI=16K SW=6002 O1=2401 PW=9.0 AQ=1.352 RD=0.00 NS=1 SR=-4.16 TE=303K 2 February 2022 Opr: Daeva E.D.; Solv: DMSO-d6;
The Best Applied NMR! On-Line: "http://nmr.ioc.ac.ru:8080"
/KANI VDB345.1
8.172
7.360
7.337
7.312
7.291
7.269
7.245
7.174
7.150
6.943
6.911
6.037
5.665
5.633
3.369
3.185
3.041
2.555
2.516
Me
H
N
S
O
COOK
3g
9.0
8.5
8.0
7.5
7.0
6.5
6.0
5.5
5.0
4.5
4.0
3.5
3.0
2.5
2.0
ppm
0.877
3.485
1.922
1.000
0.897
0.861
2.887

© Zelinsky Institute of Organic Chemistry, Moscow; Bruker AM300 SF=75.47 MHz {13C} SI=32K SW=18114 O1=8301 PW=14.3 AQ=0.901 RD=1.00 NS=815 SR=39.15 TE=303K 2 February 2022 Opr: Daeva E.D.; Solv: DMSO-d6;

The Best Applied NMR! On-Line: "http://nmr.ioc.ac.ru:8080"

/KANI VDB345.1

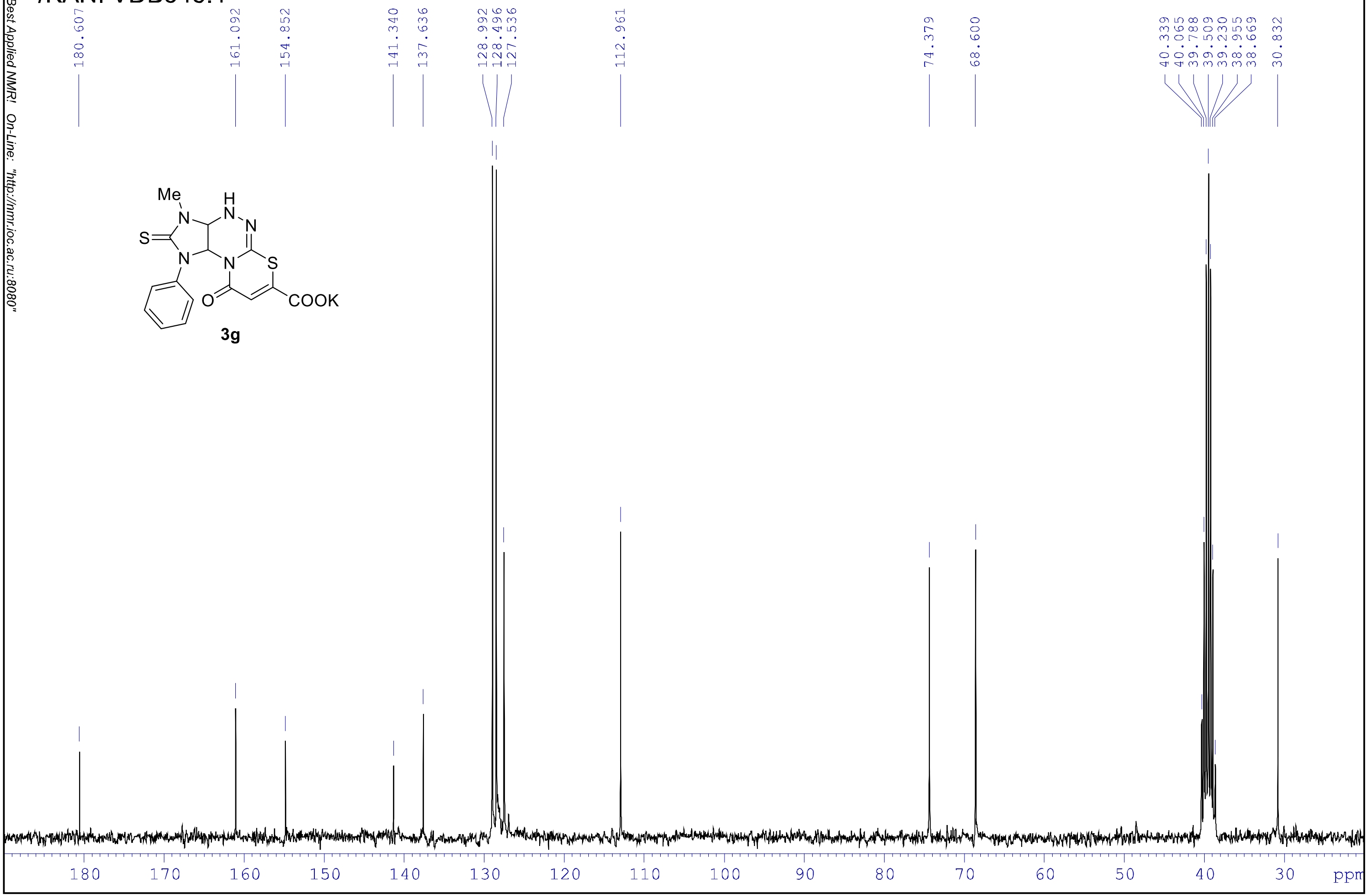

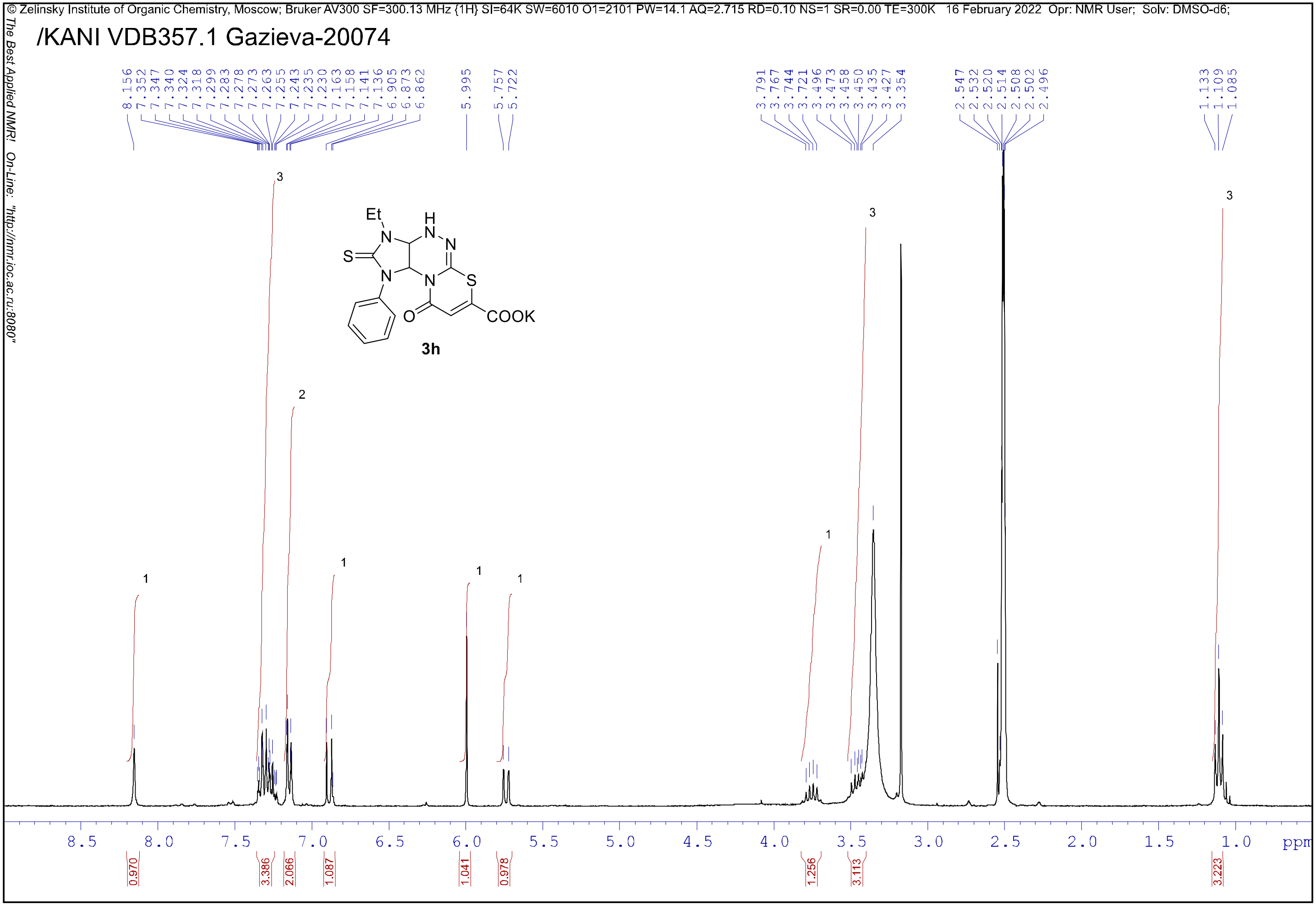

© Zelinsky Institute of Organic Chemistry, Moscow; Bruker AV300 SF=300.13 MHz {1H} SI=64K SW=6010 O1=2101 PW=14.1 AQ=2.715 RD=0.10 NS=1 SR=0.00 TE=300K 16 February 2022 Opr: NMR User; Solv: DMSO-d6;
The Best Applied NMR! On-Line: "http://nmr.ioc.ac.ru:8080"
/KANI VDB357.1 Gazieva-20074
8.156 7.352 7.347 7.340 7.324 7.318 7.299 7.283 7.278 7.273 7.263 7.255 7.243 7.235 7.230 7.163 7.158 7.141 7.136 6.905 6.873 6.862
5.995 5.757 5.722
3.791 3.767 3.744 3.721 3.496 3.473 3.458 3.450 3.435 3.427 3.354
2.547 2.532 2.520 2.514 2.508 2.502 2.496
1.133 1.109 1.085
3h
ppm
0.970 3.386 2.066 1.087 1.041 0.978 1.256 3.113 3.223

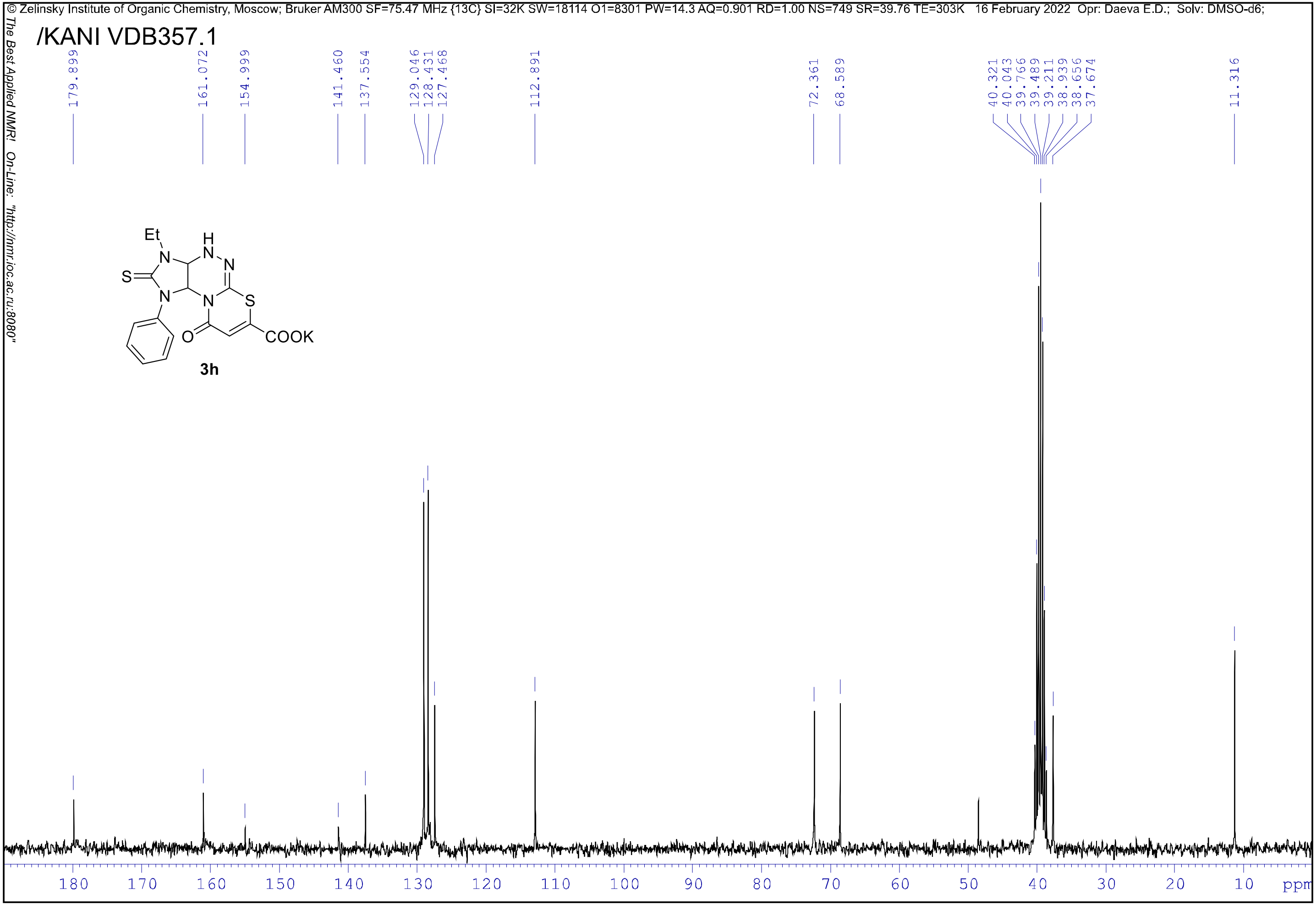
© Zelinsky Institute of Organic Chemistry, Moscow; Bruker AM300 SF=75.47 MHz {13C} SI=32K SW=18114 O1=8301 PW=14.3 AQ=0.901 RD=1.00 NS=749 SR=39.76 TE=303K 16 February 2022 Opr: Daeva E.D.; Solv: DMSO-d6;
The Best Applied NMR! On-Line: "http://nmr.ioc.ac.ru:8080"
/KANI VDB357.1
179.899
161.072
154.999
141.460
137.554
129.046
128.431
127.468
112.891
72.361
68.589
40.321
40.043
39.766
39.489
39.211
38.939
38.656
37.674
11.316
Et
H
N
S
O
COOK
3h
180
170
160
150
140
130
120
110
100
90
80
70
60
50
40
30
20
10
ppm

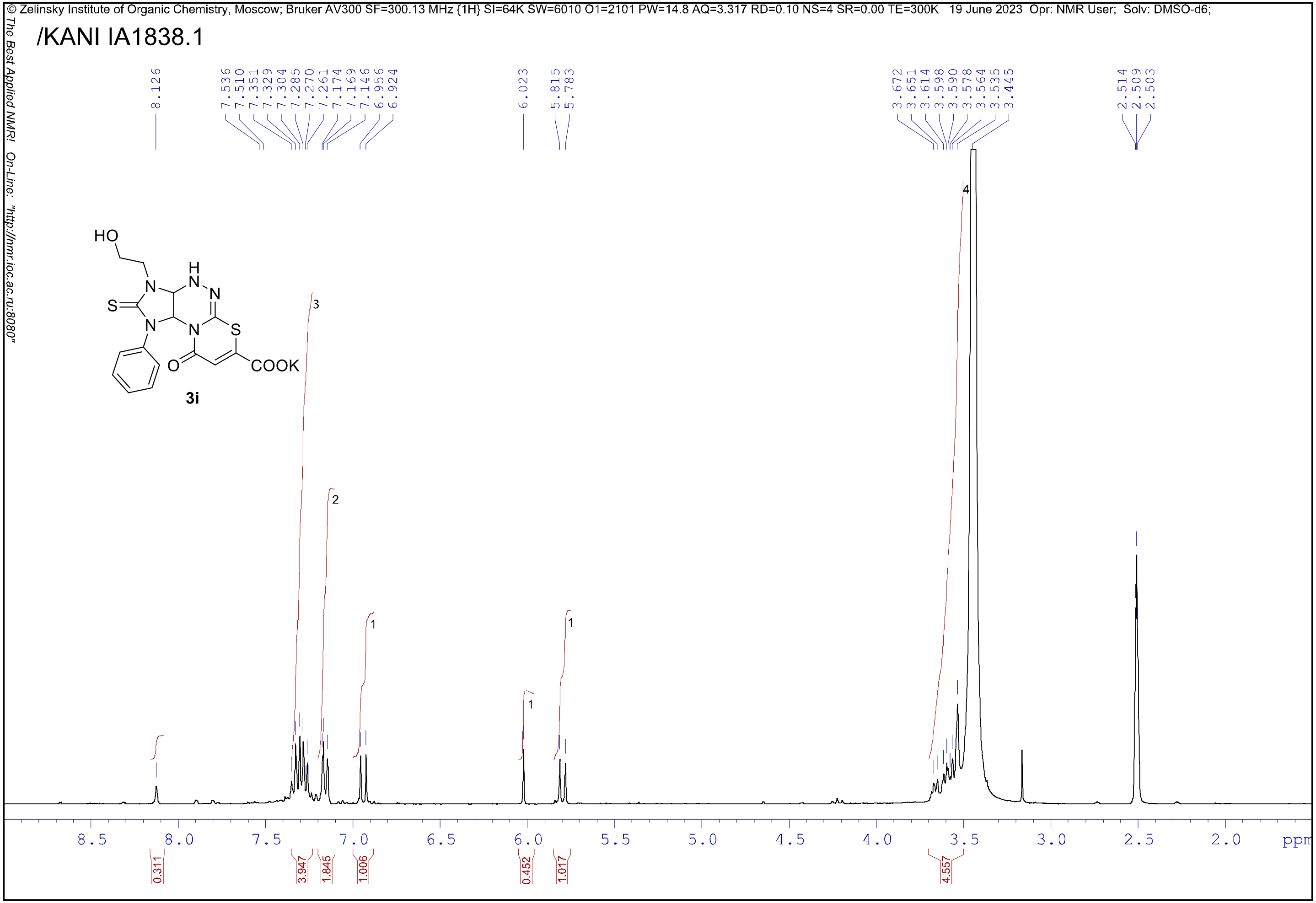
© Zelinsky Institute of Organic Chemistry, Moscow; Bruker AV300 SF=300.13 MHz {1H} SI=64K SW=6010 O1=2101 PW=14.8 AQ=3.317 RD=0.10 NS=4 SR=0.00 TE=300K 19 June 2023 Opr: NMR User; Solv: DMSO-d6;
/KANI IA1838.1
The Best Applied NMR! On-Line: "http://nmr.ioc.ac.ru:8080"
8.126
7.536 7.510 7.351 7.329 7.304 7.285 7.270 7.261 7.174 7.169 7.146 6.956 6.924
6.023
5.815 5.783
3.672 3.651 3.614 3.598 3.590 3.578 3.564 3.535 3.445
2.514 2.509 2.503
HO
H
N
N
N
S
N
N
S
O
COOK
3i
8.5 8.0 7.5 7.0 6.5 6.0 5.5 5.0 4.5 4.0 3.5 3.0 2.5 2.0 ppm
0.311
3.947
1.845
1.006
0.452
1.017
4.557

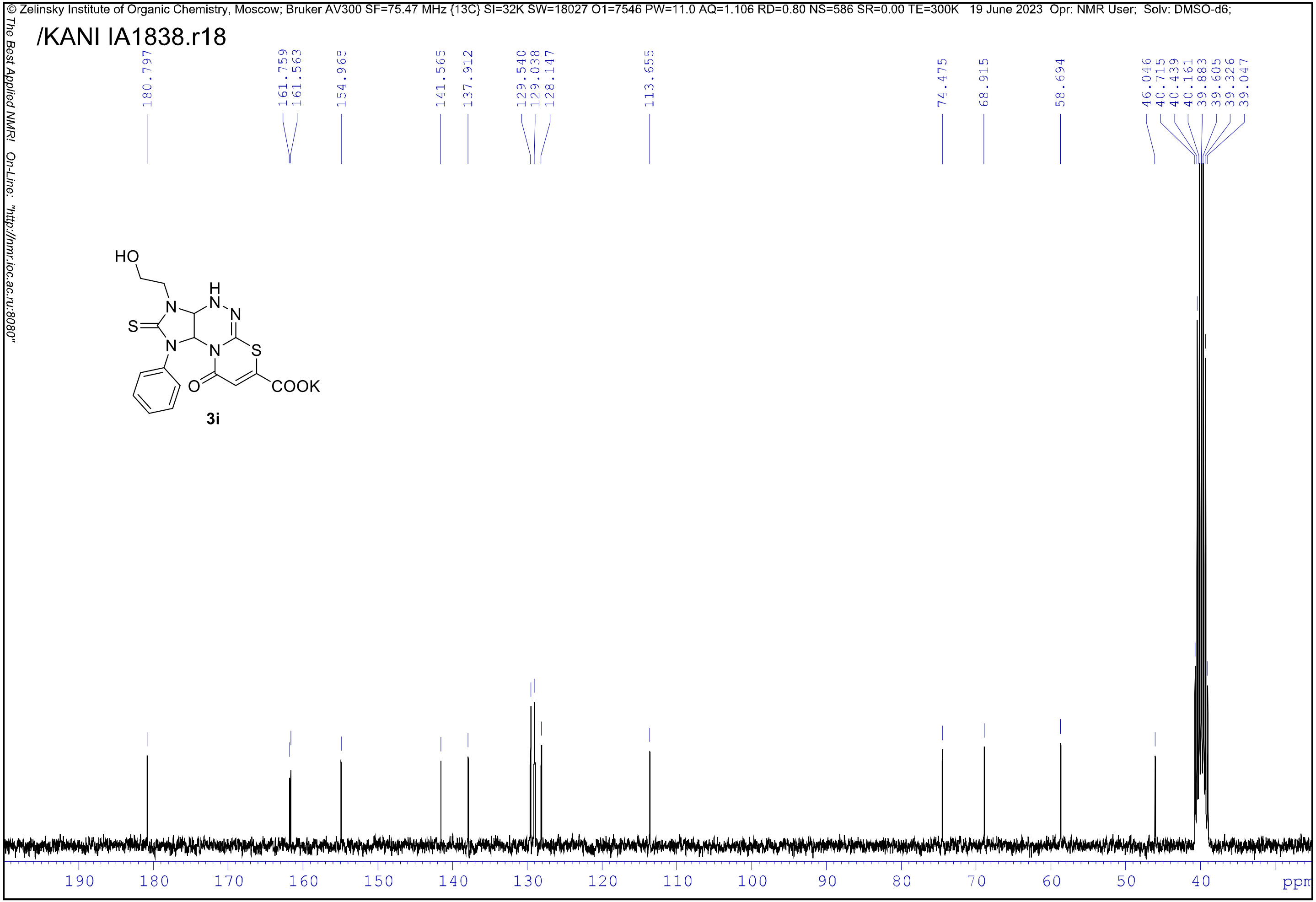
© Zelinsky Institute of Organic Chemistry, Moscow; Bruker AV300 SF=75.47 MHz {13C} SI=32K SW=18027 O1=7546 PW=11.0 AQ=1.106 RD=0.80 NS=586 SR=0.00 TE=300K 19 June 2023 Opr: NMR User; Solv: DMSO-d6;
The Best Applied NMR! On-Line: "http://nmr.ioc.ac.ru:8080"
/KANI IA1838.r18
180.797
161.759
161.563
154.96
141.565
137.912
129.540
129.038
128.147
113.655
74.475
68.915
58.694
46.046
40.715
40.439
40.161
39.883
39.605
39.326
39.047
HO
H
N
N
N
S
N
N
S
O
COOK
3i
190
180
170
160
150
140
130
120
110
100
90
80
70
60
50
40
ppm

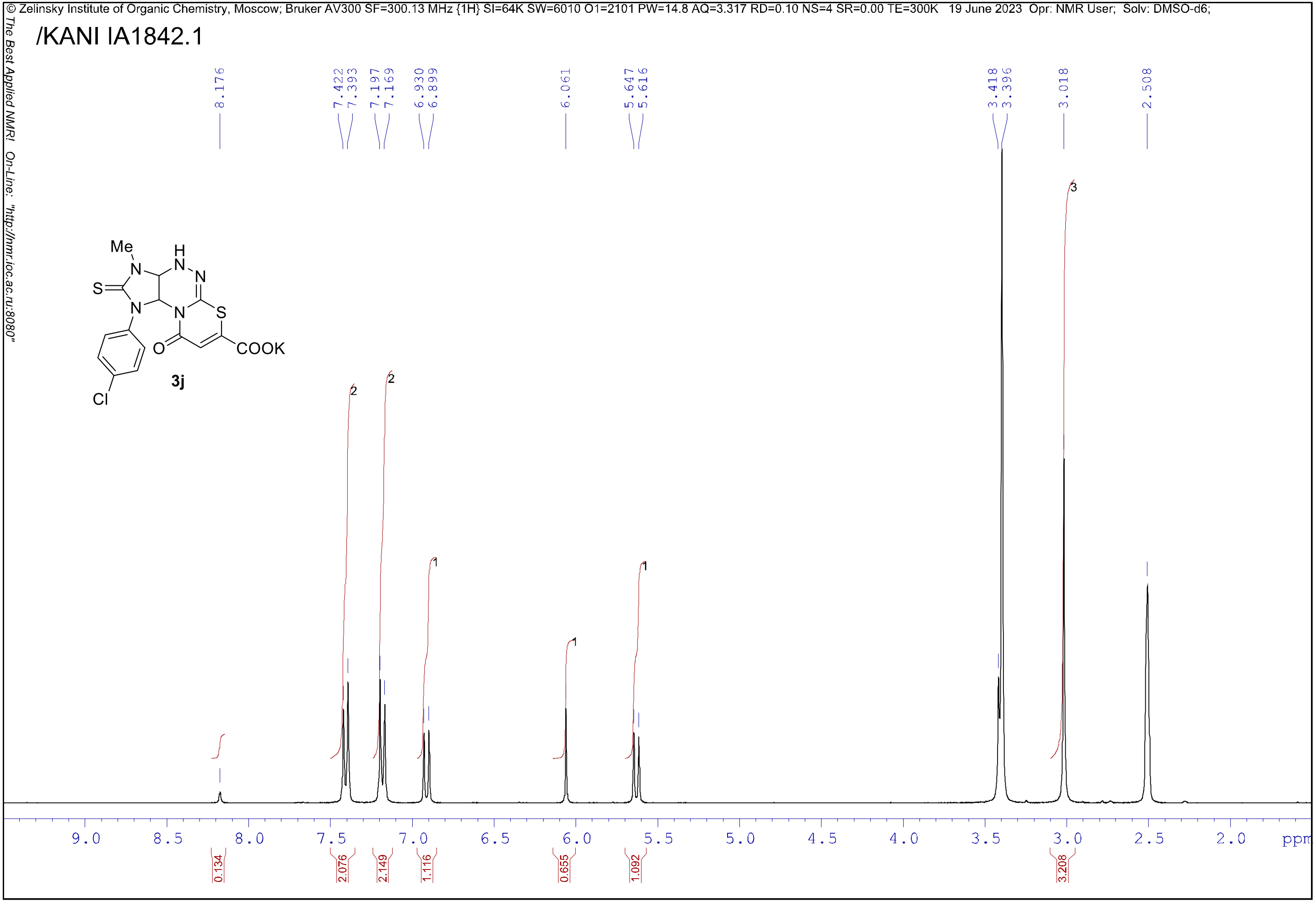
© Zelinsky Institute of Organic Chemistry, Moscow; Bruker AV300 SF=300.13 MHz {1H} SI=64K SW=6010 O1=2101 PW=14.8 AQ=3.317 RD=0.10 NS=4 SR=0.00 TE=300K 19 June 2023 Opr: NMR User; Solv: DMSO-d6;
The Best Applied NMR! On-Line: "http://nmr.ioc.ac.ru:8080"
/KANI IA1842.1
8.176
7.422
7.393
7.197
7.169
6.930
6.899
6.061
5.647
5.616
3.418
3.396
3.018
2.508
Me
H
N
N
N
S
N
N
S
O
COOK
Cl
3j
9.0
8.5
8.0
7.5
7.0
6.5
6.0
5.5
5.0
4.5
4.0
3.5
3.0
2.5
2.0
ppm
0.134
2.076
2.149
1.116
0.655
1.092
3.208

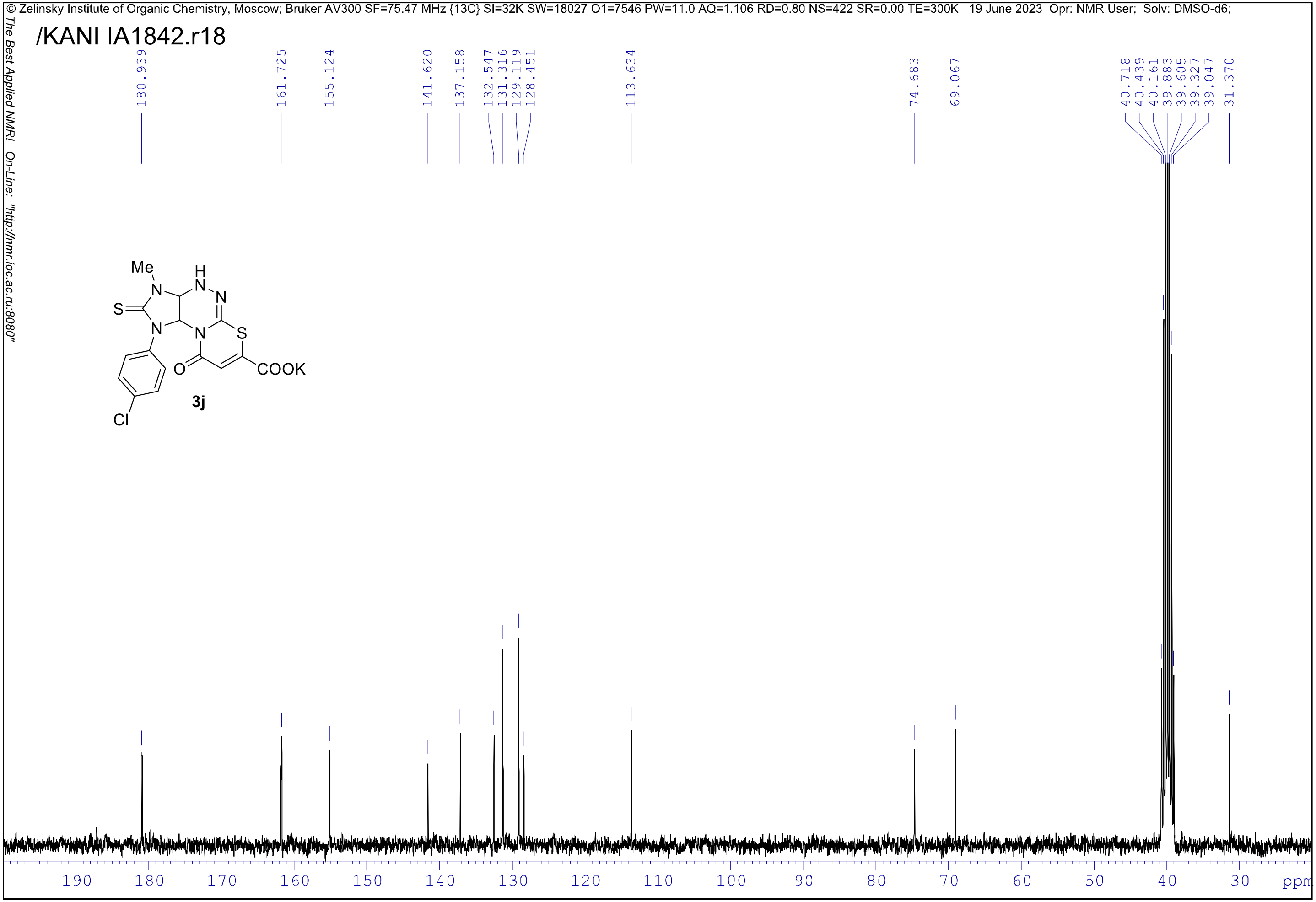
© Zelinsky Institute of Organic Chemistry, Moscow; Bruker AV300 SF=75.47 MHz {13C} SI=32K SW=18027 O1=7546 PW=11.0 AQ=1.106 RD=0.80 NS=422 SR=0.00 TE=300K 19 June 2023 Opr: NMR User; Solv: DMSO-d6;
The Best Applied NMR! On-Line: "http://nmr.ioc.ac.ru:8080"
/KANI IA1842.r18
180.939
161.725
155.124
141.620
137.158
132.547
131.316
129.119
128.451
113.634
74.683
69.067
40.718
40.439
40.161
39.883
39.605
39.327
39.047
31.370
Me
H
N
S
O
COOK
Cl
3j
190 180 170 160 150 140 130 120 110 100 90 80 70 60 50 40 30 ppm

© Zelinsky Institute of Organic Chemistry, Moscow; Bruker AV300 SF=300.13 MHz {1H} SI=64K SW=6010 O1=2101 PW=14.8 AQ=3.317 RD=0.10 NS=4 SR=0.00 TE=300K 16 June 2023 Opr: NMR User; Solv: DMSO-d6;

The Best Applied NMR! On-Line: "http://nmr.ioc.ac.ru:8080"

/KANI VDB593.1.1 Gazieva-20074

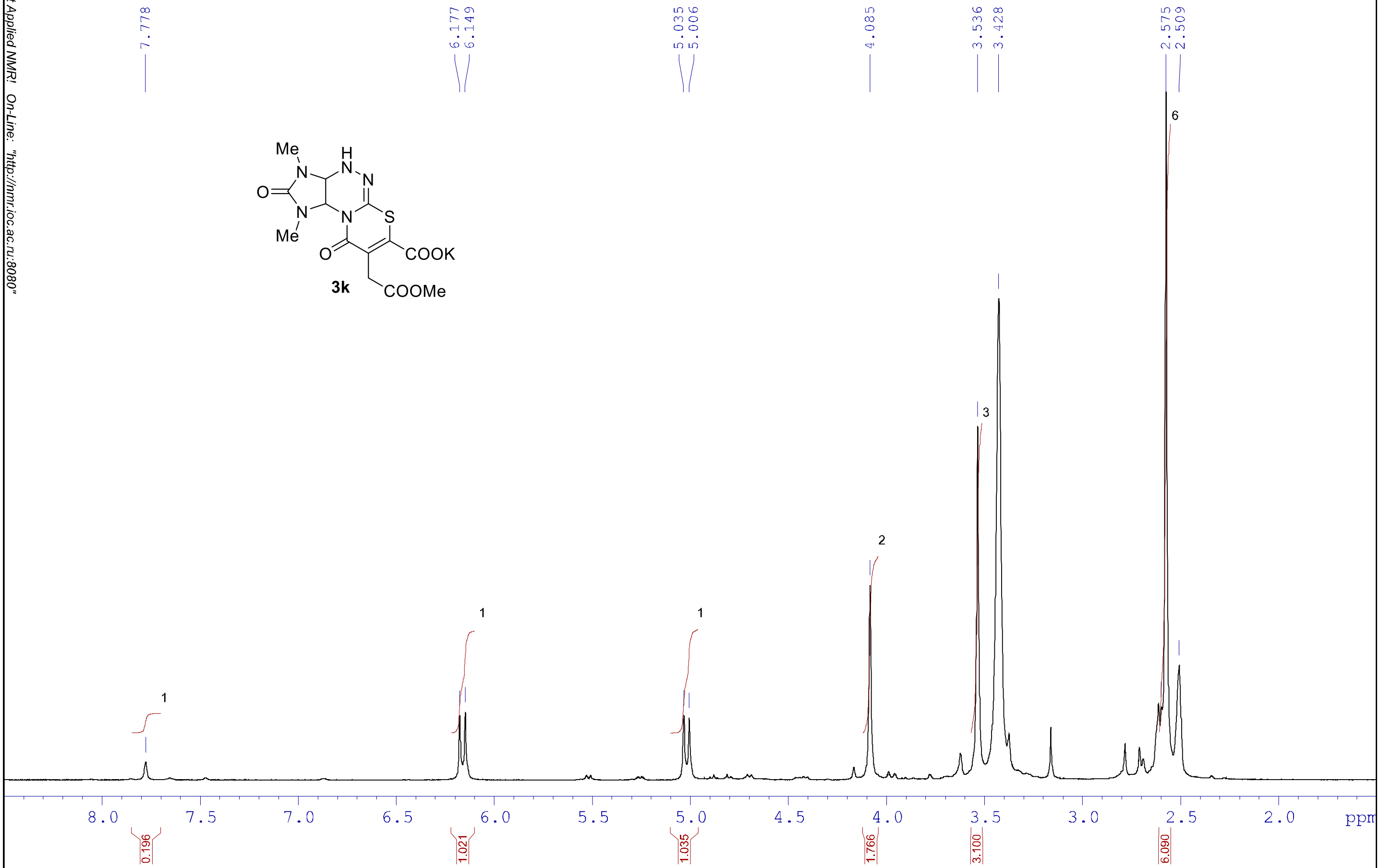

3k

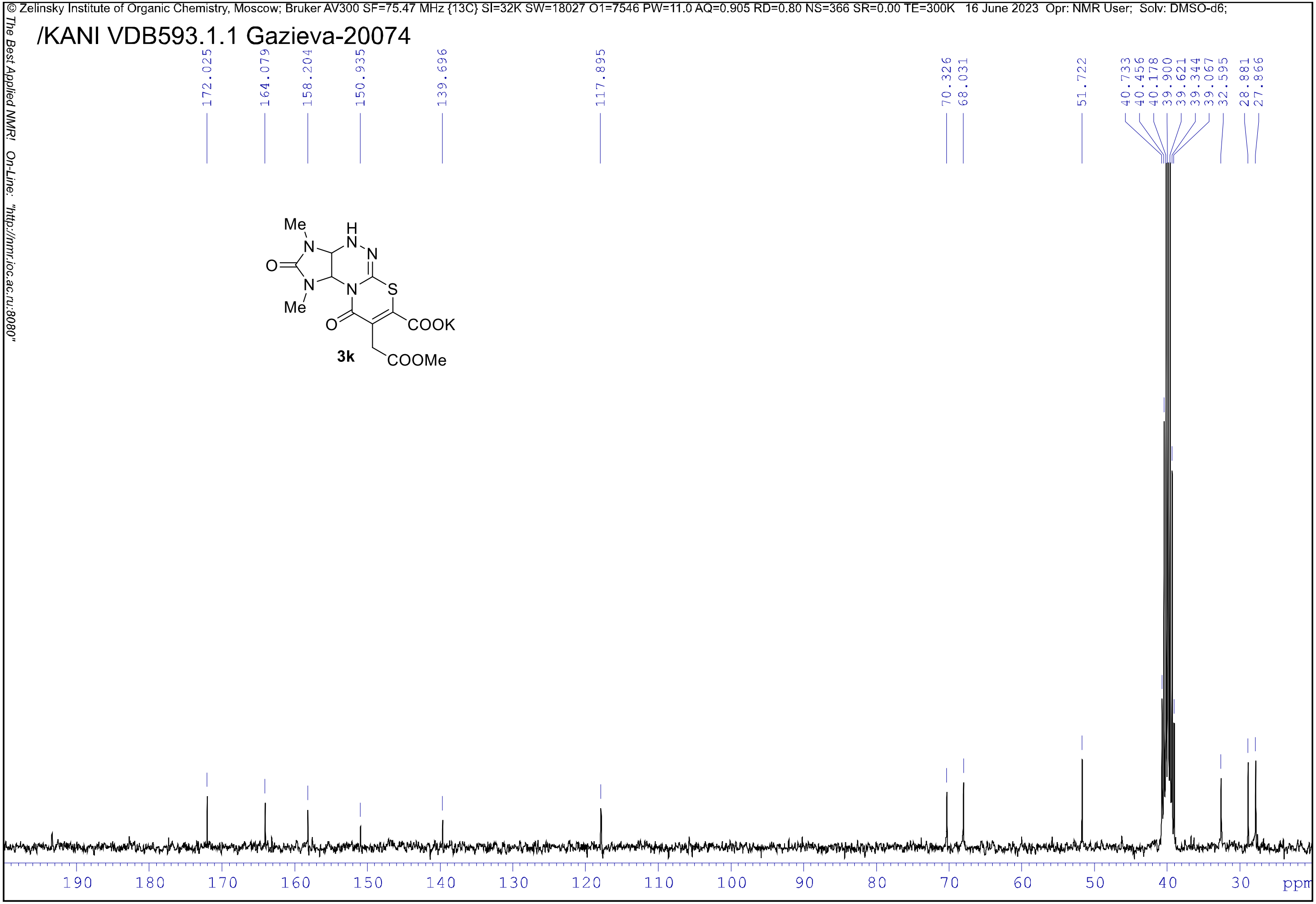
© Zelinsky Institute of Organic Chemistry, Moscow; Bruker AV300 SF=75.47 MHz {13C} SI=32K SW=18027 O1=7546 PW=11.0 AQ=0.905 RD=0.80 NS=366 SR=0.00 TE=300K 16 June 2023 Opr: NMR User; Solv: DMSO-d6;
The Best Applied NMR! On-Line: "http://nmr.ioc.ac.ru:8080"
/KANI VDB593.1.1 Gazieva-20074
172.025
164.079
158.204
150.935
139.696
117.895
70.326
68.031
51.722
40.733
40.456
40.178
39.900
39.621
39.344
39.067
32.595
28.881
27.866
Me
H
N
N
N
O
N
N
S
Me
O
COOK
3k
COOMe
190
180
170
160
150
140
130
120
110
100
90
80
70
60
50
40
30
ppm

© Zelinsky Institute of Organic Chemistry, Moscow; Bruker AV300 SF=300.13 MHz {1H-13C}HMBC SI=1024x1024 SW=1548 O1=1213 PW=14.8 AQ=0.618 RD=0.65 NS=2x300 SR=0.00 TE=300K 16 June 2023 Opr: NMR User; Solv: DMSO-d6;

The Best Applied NMR! On-Line: "http://nmr.ioc.ac.ru:8080"

/KANI VDB593.1.1 Gazieva-20074
HMBC

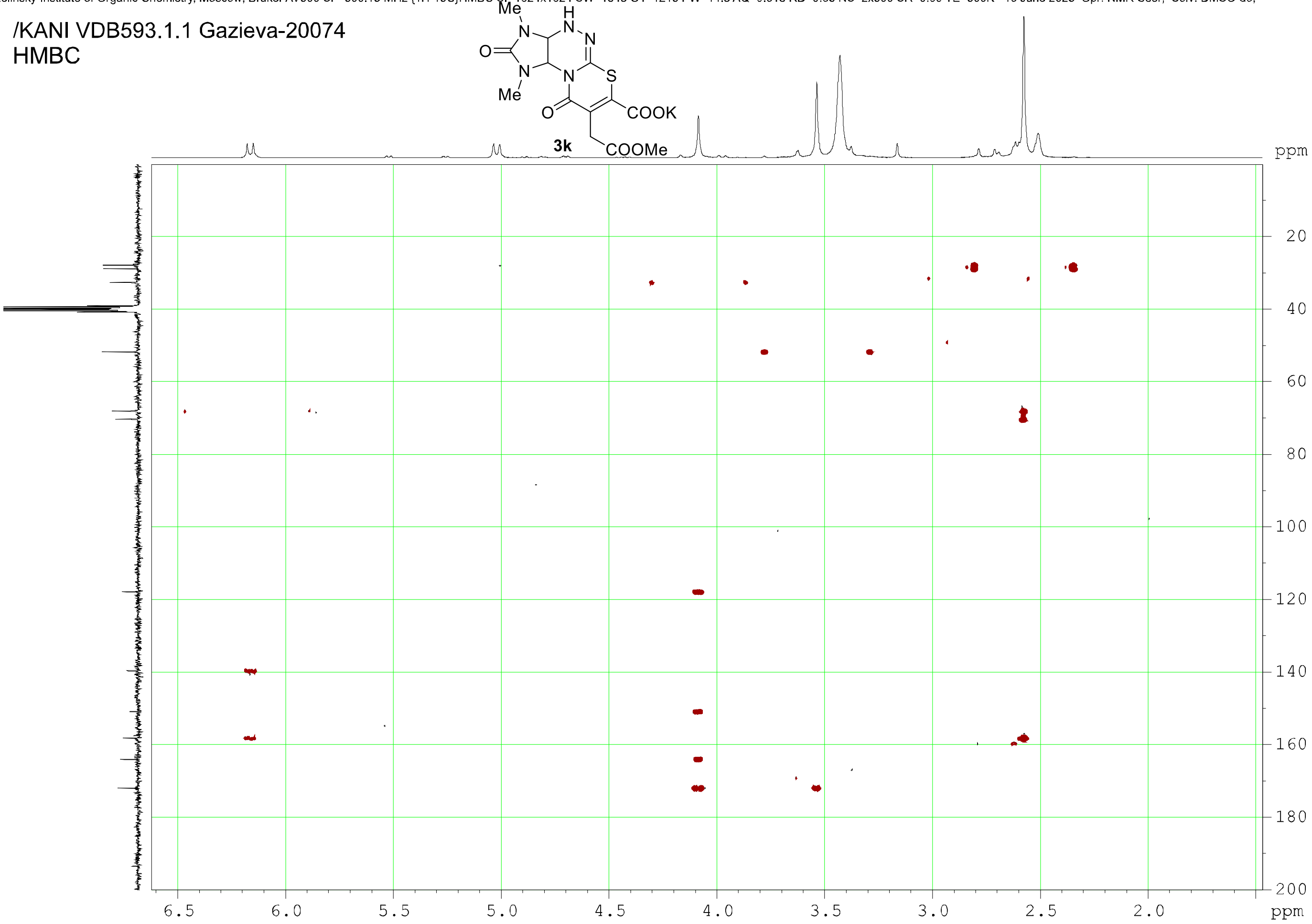

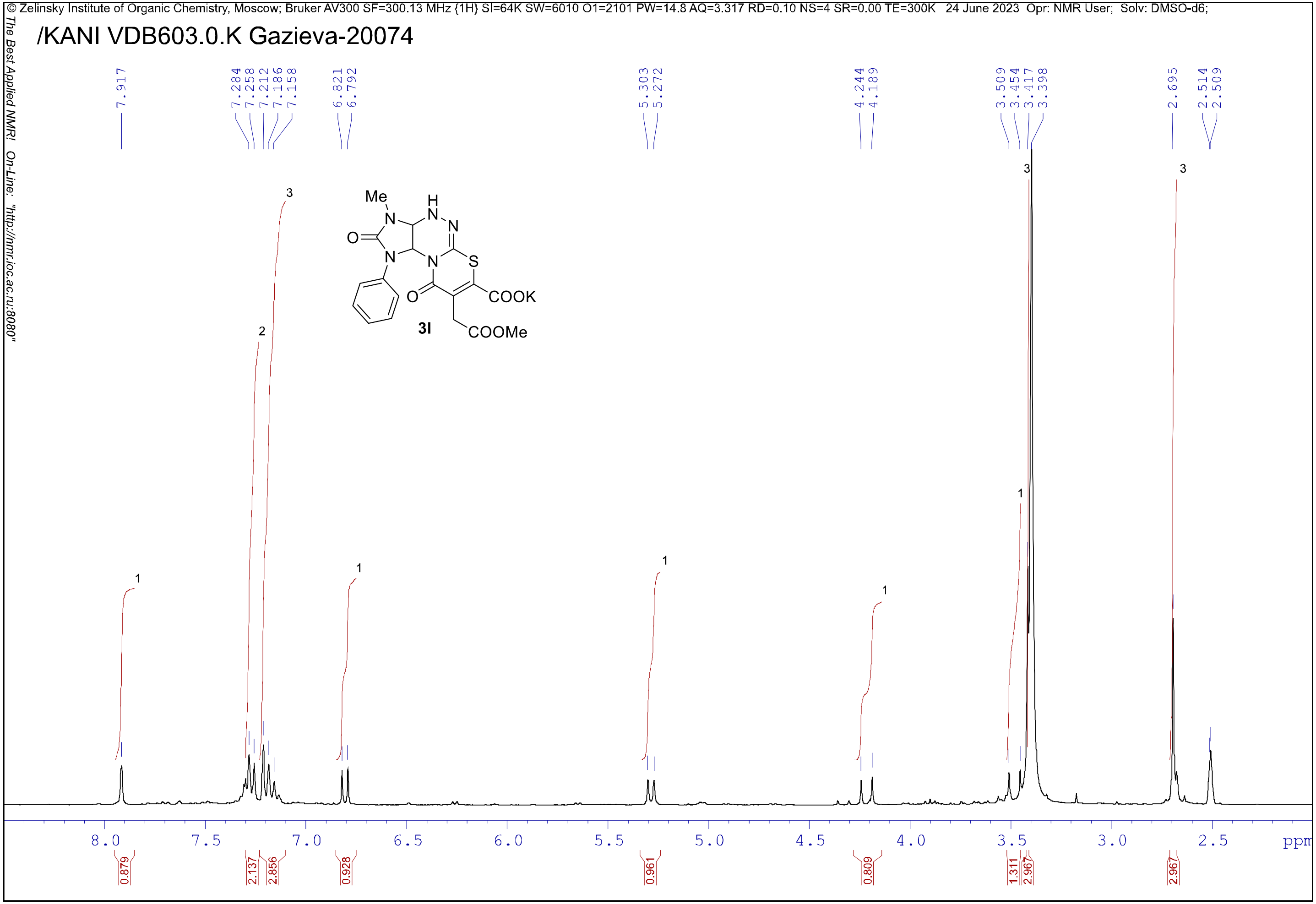
© Zelinsky Institute of Organic Chemistry, Moscow; Bruker AV300 SF=300.13 MHz {1H} SI=64K SW=6010 O1=2101 PW=14.8 AQ=3.317 RD=0.10 NS=4 SR=0.00 TE=300K 24 June 2023 Opr: NMR User; Solv: DMSO-d6;
The Best Applied NMR! On-Line: "http://nmr.ioc.ac.ru:8080"
/KANI VDB603.0.K Gazieva-20074
7.917
7.284 7.258 7.212 7.186 7.158
6.821 6.792
5.303 5.272
4.244 4.189
3.509 3.454 3.417 3.398
2.695 2.514 2.509
Me
H
N
O
S
COOK
COOMe
3l
8.0 7.5 7.0 6.5 6.0 5.5 5.0 4.5 4.0 3.5 3.0 2.5 ppm
0.879 2.137 2.856 0.928 0.961 0.809 1.311 2.967 2.967

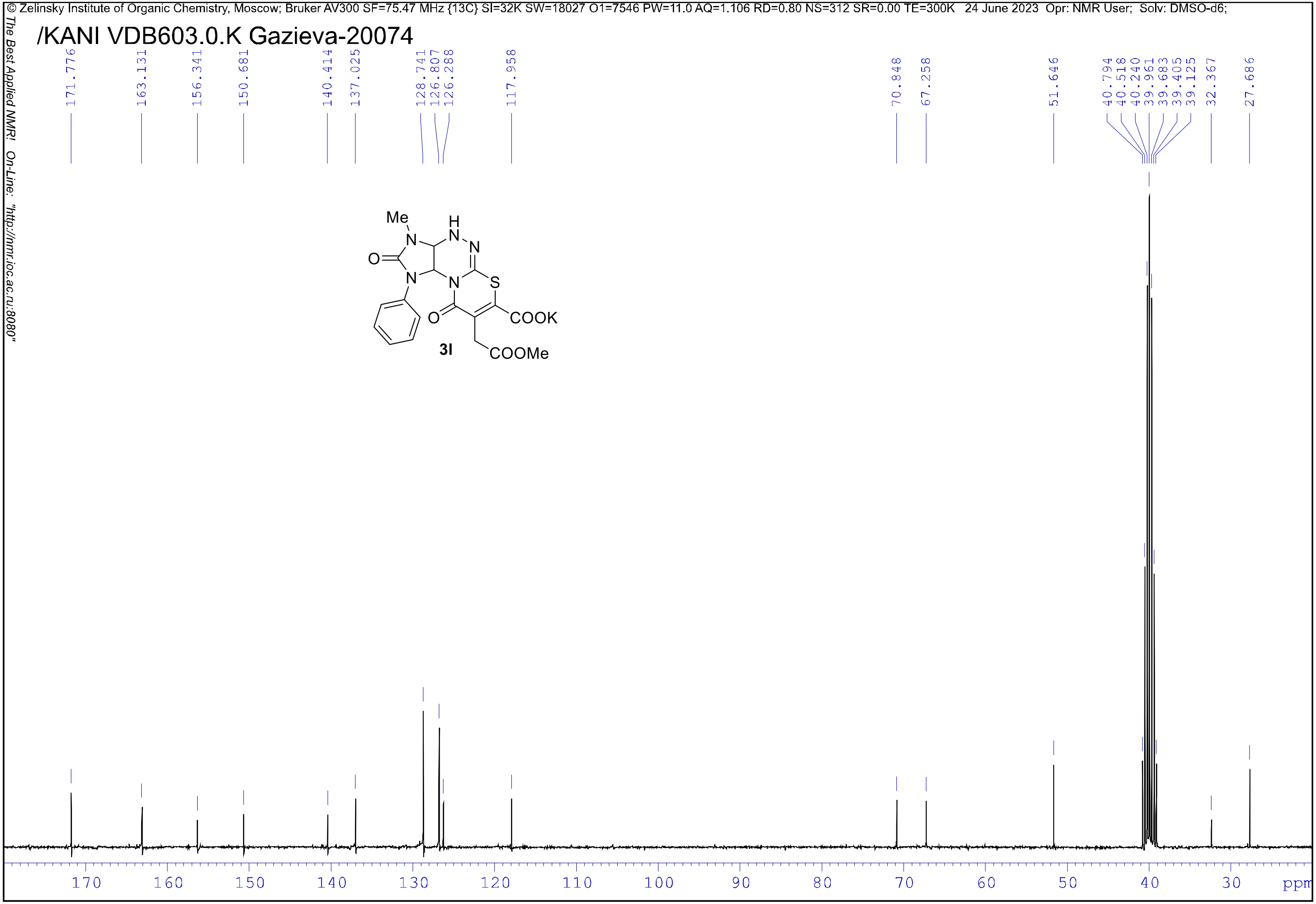
© Zelinsky Institute of Organic Chemistry, Moscow; Bruker AV300 SF=75.47 MHz {13C} SI=32K SW=18027 O1=7546 PW=11.0 AQ=1.106 RD=0.80 NS=312 SR=0.00 TE=300K 24 June 2023 Opr: NMR User; Solv: DMSO-d6;
The Best Applied NMR! On-Line: "http://nmr.ioc.ac.ru:8080"
/KANI VDB603.0.K Gazieva-20074
171.776
163.131
156.341
150.681
140.414
137.025
128.741
126.807
126.288
117.958
70.848
67.258
51.646
40.794
40.518
40.240
39.961
39.683
39.405
39.125
32.367
27.686
Me
H
N
O
S
COOK
COOMe
3l
170
160
150
140
130
120
110
100
90
80
70
60
50
40
30
ppm

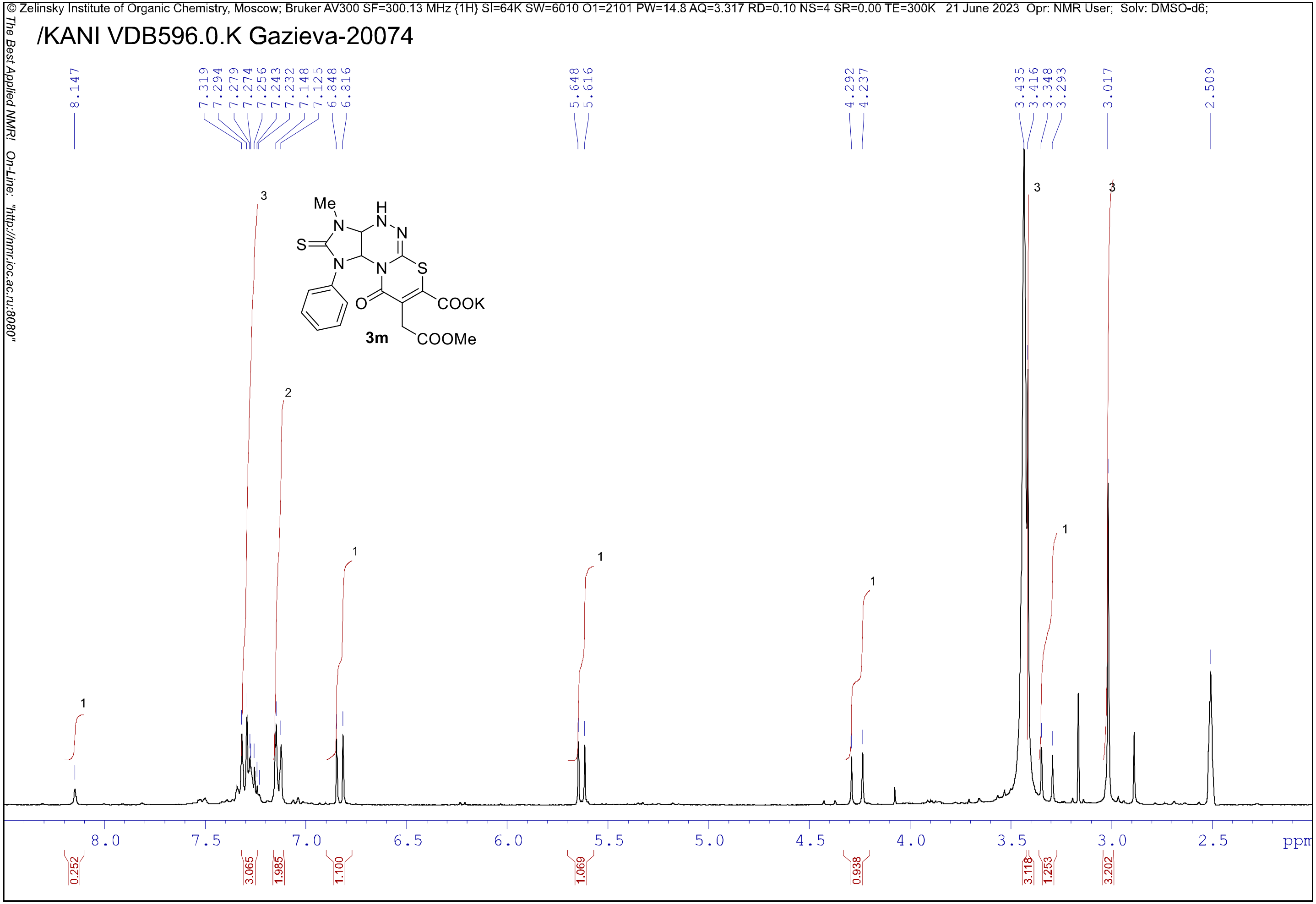
© Zelinsky Institute of Organic Chemistry, Moscow; Bruker AV300 SF=300.13 MHz {1H} SI=64K SW=6010 O1=2101 PW=14.8 AQ=3.317 RD=0.10 NS=4 SR=0.00 TE=300K 21 June 2023 Opr: NMR User; Solv: DMSO-d6;
/KANI VDB596.0.K Gazieva-20074
The Best Applied NMR! On-Line: "http://nmr.ioc.ac.ru:8080"
8.147
7.319 7.294 7.279 7.274 7.256 7.243 7.232 7.148 7.125 6.848 6.816
5.648 5.616
4.292 4.237
3.435 3.416 3.348 3.293
3.017
2.509
Me
H
N
S
O
COOK
COOMe
3m
8.0 7.5 7.0 6.5 6.0 5.5 5.0 4.5 4.0 3.5 3.0 2.5 ppm
0.252 3.065 1.985 1.100 1.069 0.938 3.118 1.253 3.202

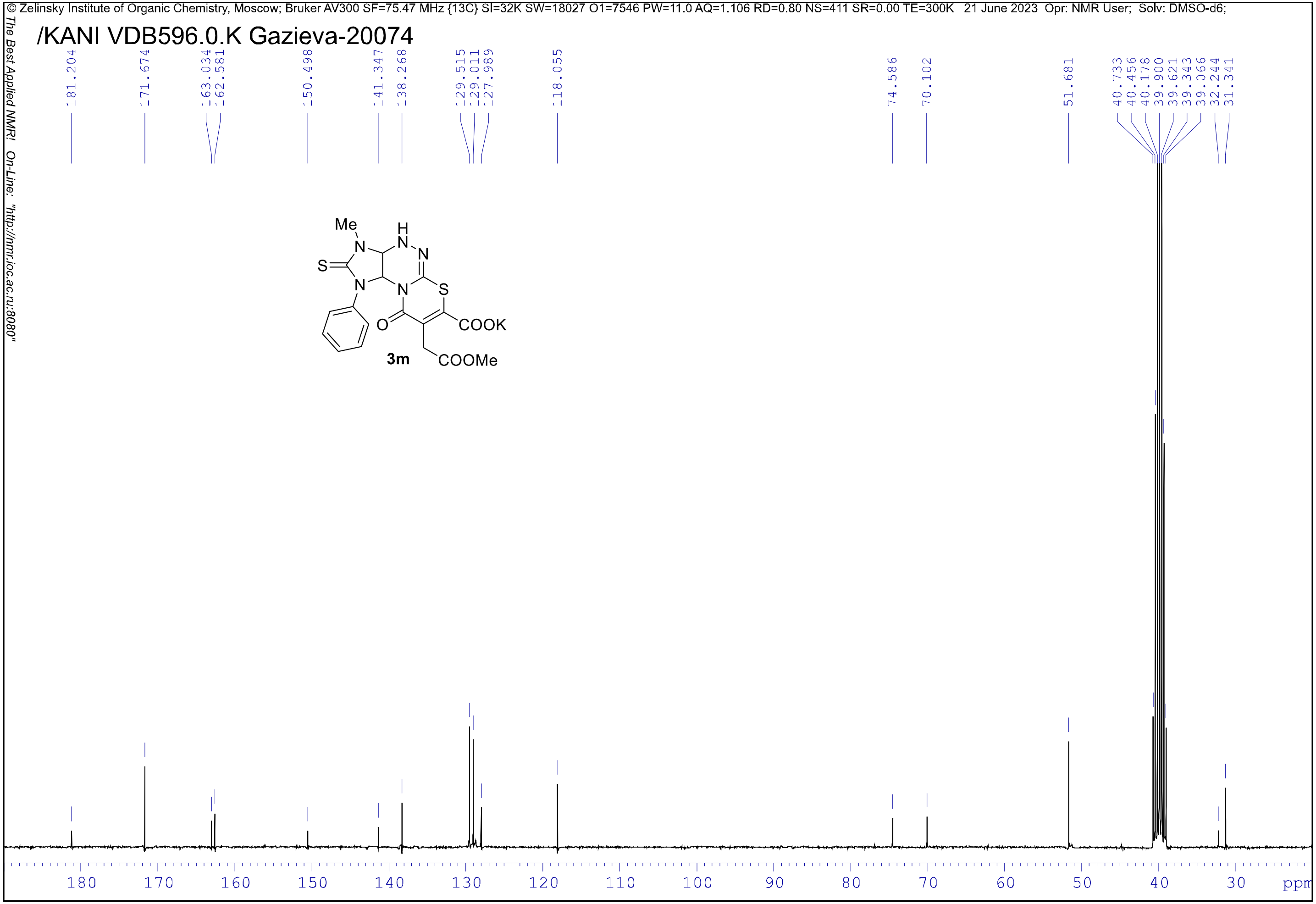
© Zelinsky Institute of Organic Chemistry, Moscow; Bruker AV300 SF=75.47 MHz {13C} SI=32K SW=18027 O1=7546 PW=11.0 AQ=1.106 RD=0.80 NS=411 SR=0.00 TE=300K 21 June 2023 Opr: NMR User; Solv: DMSO-d6;
The Best Applied NMR! On-Line: "http://nmr.ioc.ac.ru:8080"
/KANI VDB596.0.K Gazieva-20074
181.204
171.674
163.034
162.581
150.498
141.347
138.268
129.515
129.011
127.989
118.055
74.586
70.102
51.681
40.733
40.456
40.178
39.900
39.621
39.343
39.066
32.244
31.341
Me
H
N
S
O
COOK
COOMe
3m
180
170
160
150
140
130
120
110
100
90
80
70
60
50
40
30
ppm

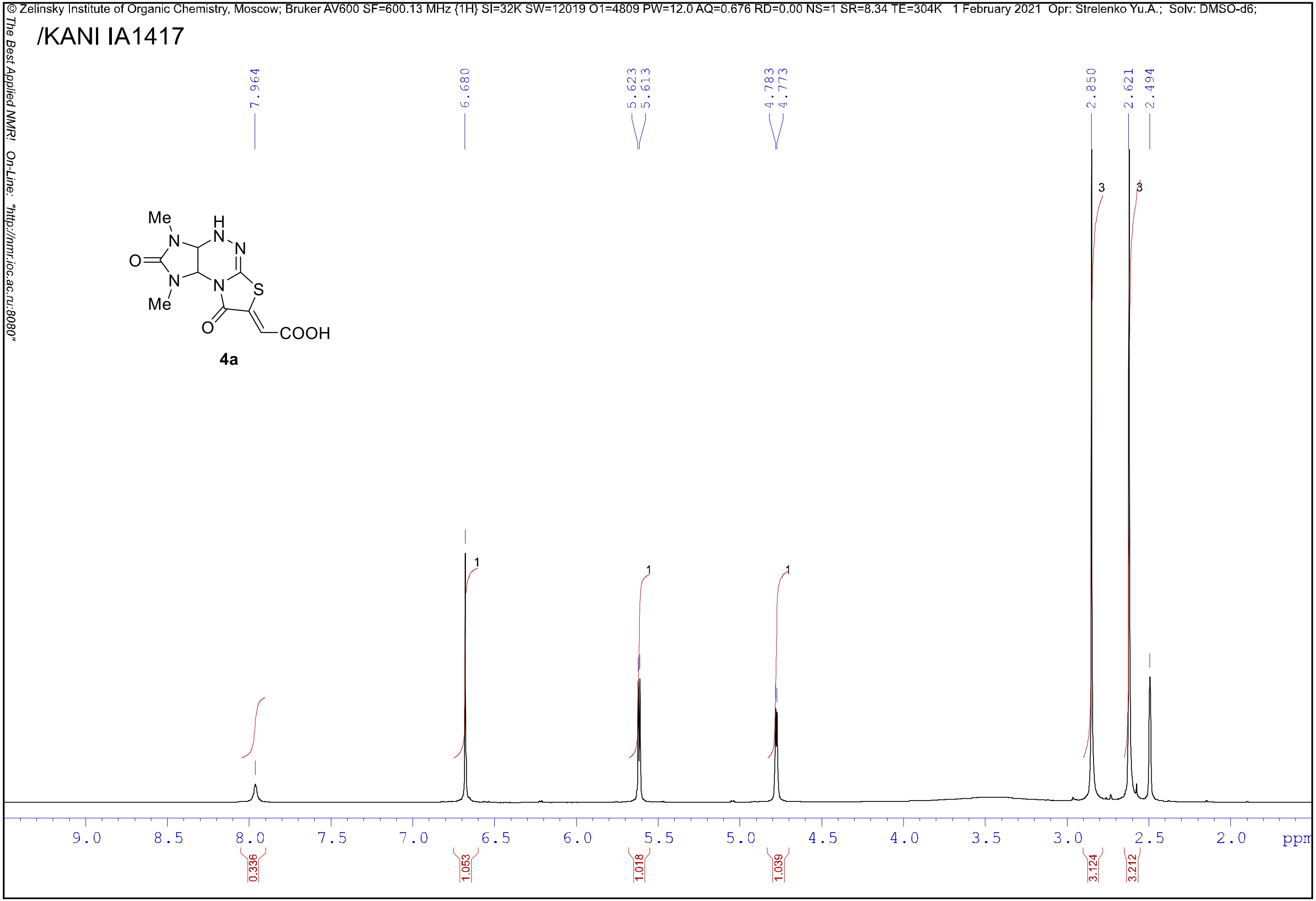
© Zelinsky Institute of Organic Chemistry, Moscow; Bruker AV600 SF=600.13 MHz {1H} SI=32K SW=12019 O1=4809 PW=12.0 AQ=0.676 RD=0.00 NS=1 SR=8.34 TE=304K 1 February 2021 Opr: Strelenko Yu.A.; Solv: DMSO-d6;
The Best Applied NMR! On-Line: "http://nmr.ioc.ac.ru:8080"
/KANI IA1417
7.964
6.680
5.623
5.613
4.783
4.773
2.850
2.621
2.494
Me
H
N
N
N
O
N
N
S
Me
O
COOH
4a
1
1
1
3
3
9.0
8.5
8.0
7.5
7.0
6.5
6.0
5.5
5.0
4.5
4.0
3.5
3.0
2.5
2.0
ppm
0.336
1.053
1.018
1.039
3.124
3.212

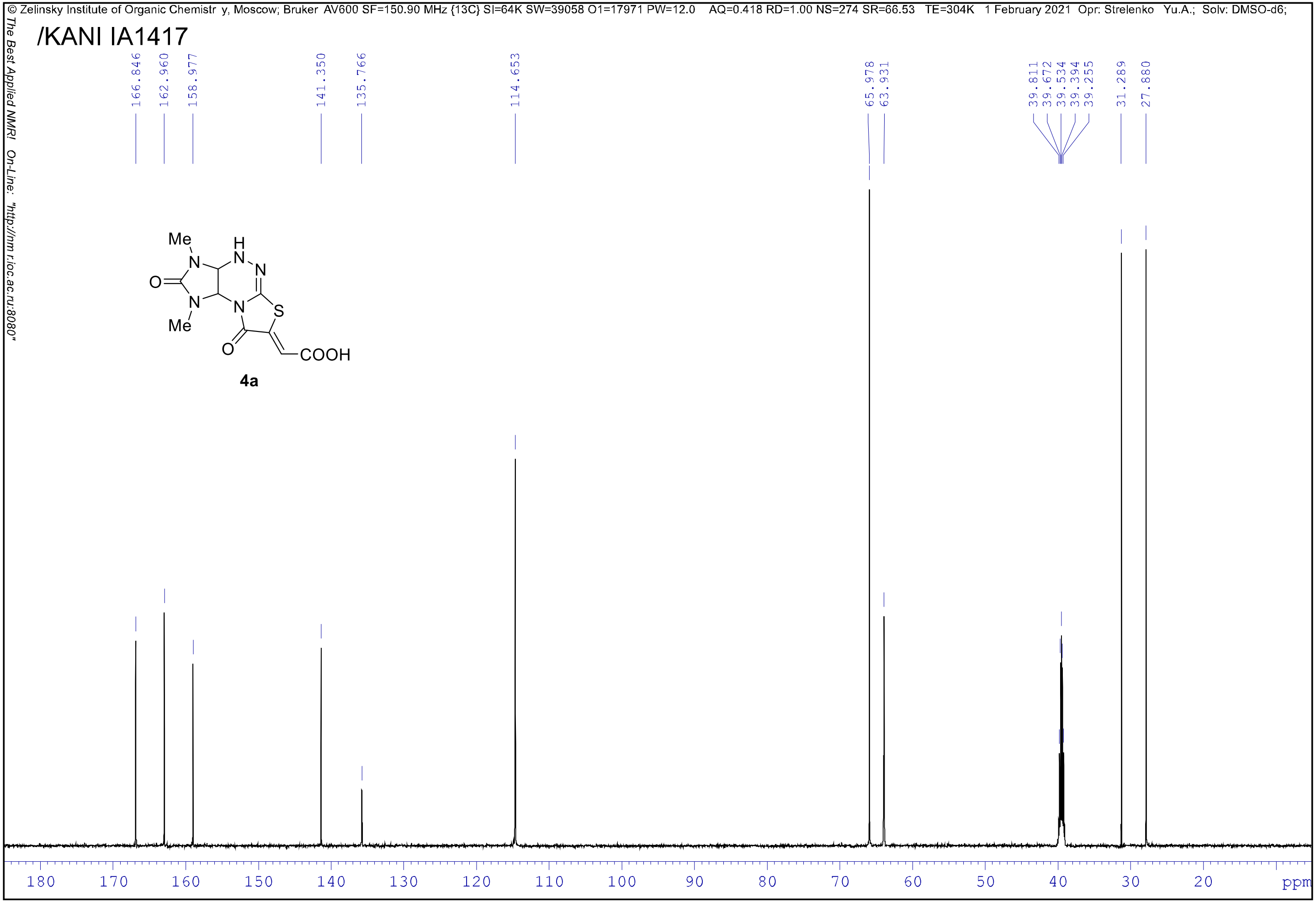
© Zelinsky Institute of Organic Chemistr y, Moscow; Bruker AV600 SF=150.90 MHz {13C} SI=64K SW=39058 O1=17971 PW=12.0 AQ=0.418 RD=1.00 NS=274 SR=66.53 TE=304K 1 February 2021 Opr: Strelenko Yu.A.; Solv: DMSO-d6;
The Best Applied NMR! On-Line: "http://nm r.ioc.ac.ru:8080"
/KANI IA1417
166.846
162.960
158.977
141.350
135.766
114.653
65.978
63.931
39.811
39.672
39.534
39.394
39.255
31.289
27.880
Me
H
N
N
N
O
N
N
S
Me
O
COOH
4a
180
170
160
150
140
130
120
110
100
90
80
70
60
50
40
30
20
ppm

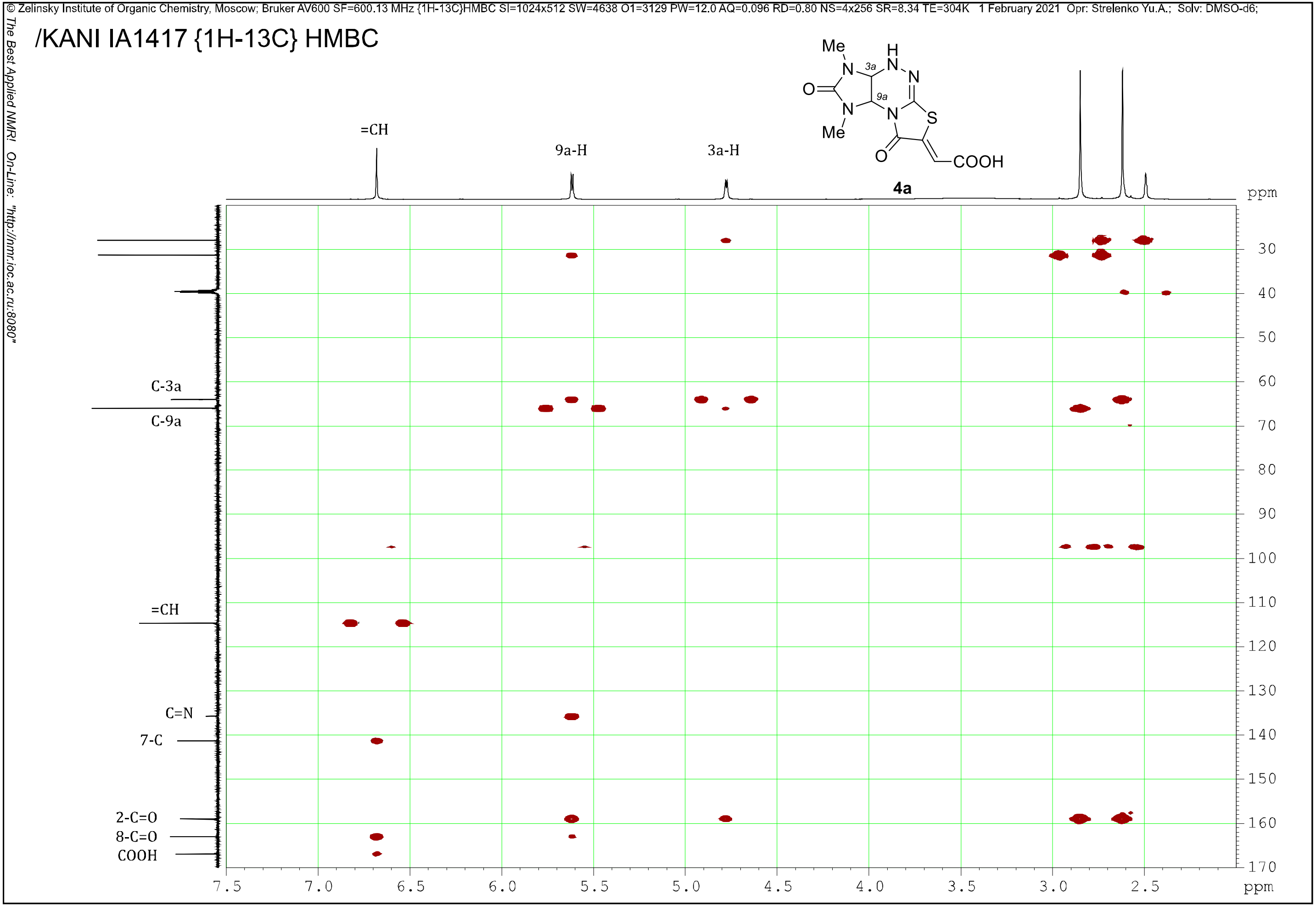

© Zelinsky Institute of Organic Chemistry, Moscow; Bruker AV600 SF=600.13 MHz {1H-13C}HMBC SI=1024x512 SW=4638 O1=3129 PW=12.0 AQ=0.096 RD=0.80 NS=4x256 SR=8.34 TE=304K 1 February 2021 Opr: Strelenko Yu.A.; Solv: DMSO-d6;
The Best Applied NMR! On-Line: "http://nmr.ioc.ac.ru:8080"
/KANI IA1417 {1H-13C} HMBC
4a
COOH
=CH
9a-H
3a-H
C-3a
C-9a
=CH
C=N
7-C
2-C=O
8-C=O
COOH
ppm

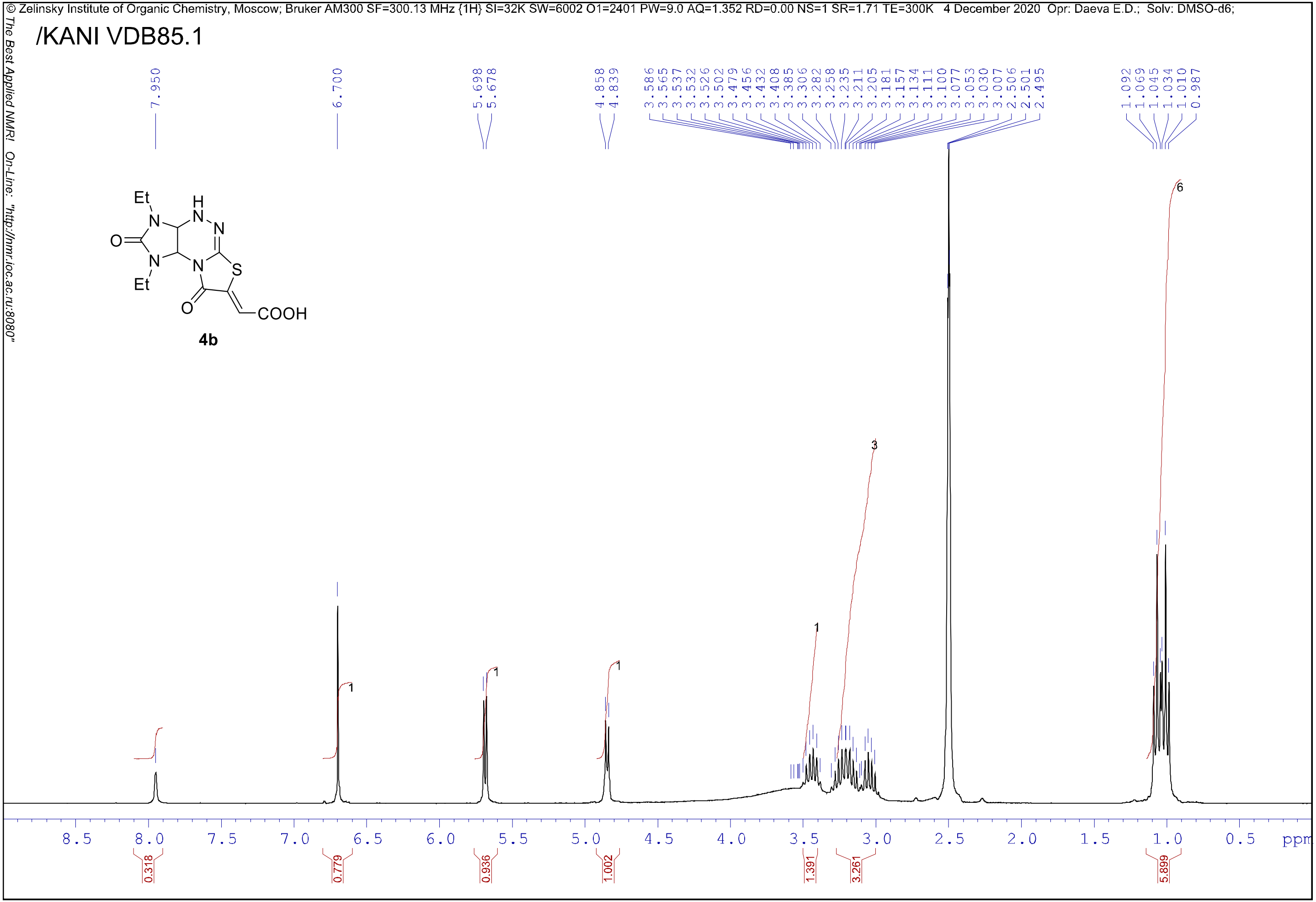
© Zelinsky Institute of Organic Chemistry, Moscow; Bruker AM300 SF=300.13 MHz {1H} SI=32K SW=6002 O1=2401 PW=9.0 AQ=1.352 RD=0.00 NS=1 SR=1.71 TE=300K 4 December 2020 Opr: Daeva E.D.; Solv: DMSO-d6;
/KANI VDB85.1
The Best Applied NMR! On-Line: "http://nmr.ioc.ac.ru:8080"
7.950
6.700
5.698
5.678
4.858
4.839
3.586
3.565
3.537
3.532
3.526
3.502
3.479
3.456
3.432
3.408
3.385
3.306
3.282
3.258
3.235
3.211
3.205
3.181
3.157
3.134
3.111
3.100
3.077
3.053
3.030
3.007
2.506
2.501
2.495
1.092
1.069
1.045
1.034
1.010
0.987
Et
H
N
N
N
O
N
N
S
Et
O
COOH
4b
ppm
0.318
0.779
0.936
1.002
1.391
3.261
5.899

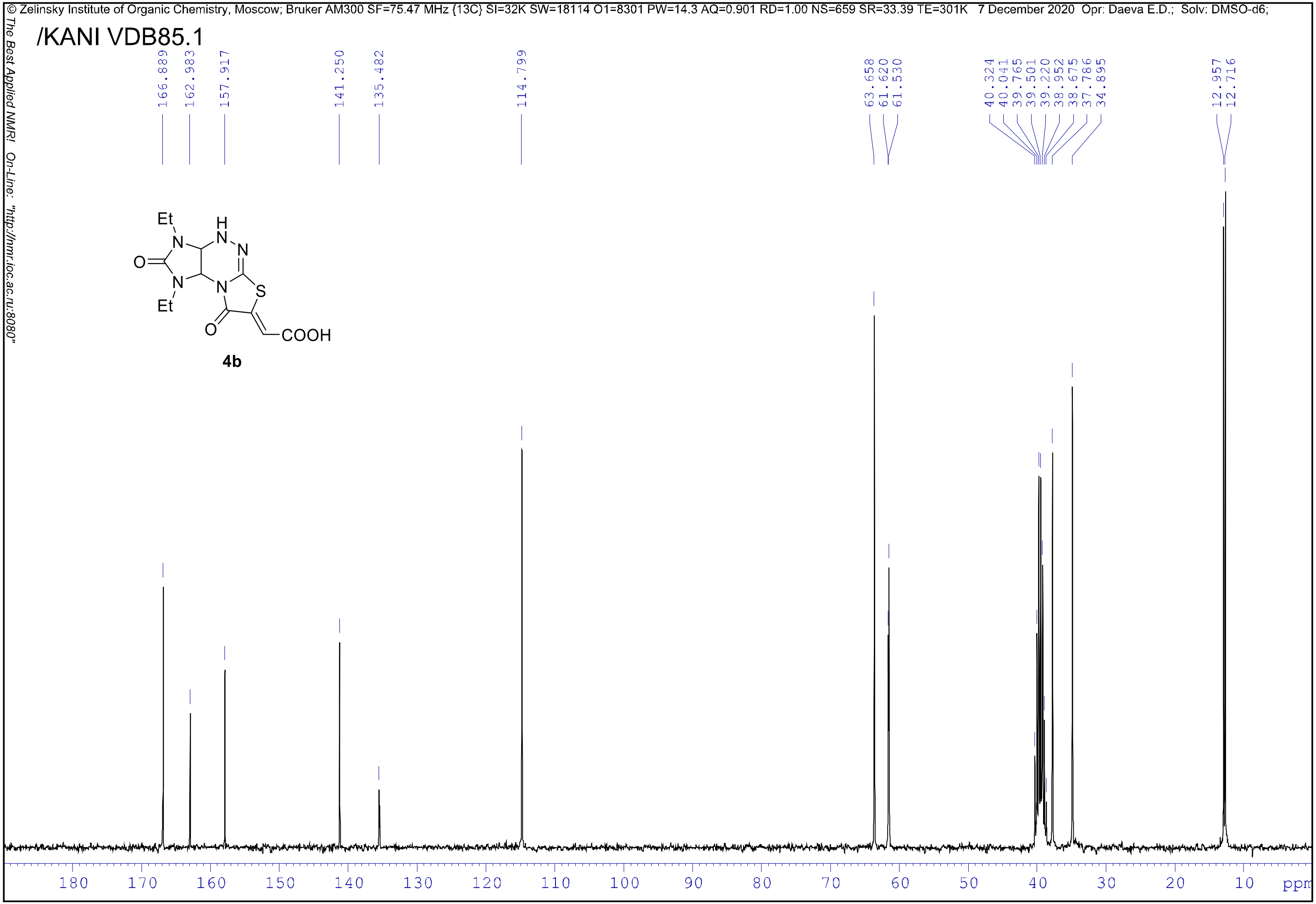
© Zelinsky Institute of Organic Chemistry, Moscow; Bruker AM300 SF=75.47 MHz {13C} SI=32K SW=18114 O1=8301 PW=14.3 AQ=0.901 RD=1.00 NS=659 SR=33.39 TE=301K 7 December 2020 Opr: Daeva E.D.; Solv: DMSO-d6;
/KANI VDB85.1
The Best Applied NMR! On-Line: "http://nmr.ioc.ac.ru:8080"
166.889
162.983
157.917
141.250
135.482
114.799
63.658
61.620
61.530
40.324
40.041
39.765
39.501
39.220
38.952
38.675
37.786
34.895
12.957
12.716
Et
H
N
N
O
N
N
S
Et
O
COOH
4b
180 170 160 150 140 130 120 110 100 90 80 70 60 50 40 30 20 10 ppm

© Zelinsky Institute of Organic Chemistry, Moscow; Bruker AM300 SF=300.13 MHz {1H} SI=32K SW=6002 O1=2401 PW=9.0 AQ=1.352 RD=0.00 NS=1 SR=1.69 TE=300K 24 January 2020 Opr: Daeva E.D.; Solv: DMSO-d6;

The Best Applied NMR! On-Line: "http://nmr.ioc.ac.ru:8080"

/KANI IA1190.1

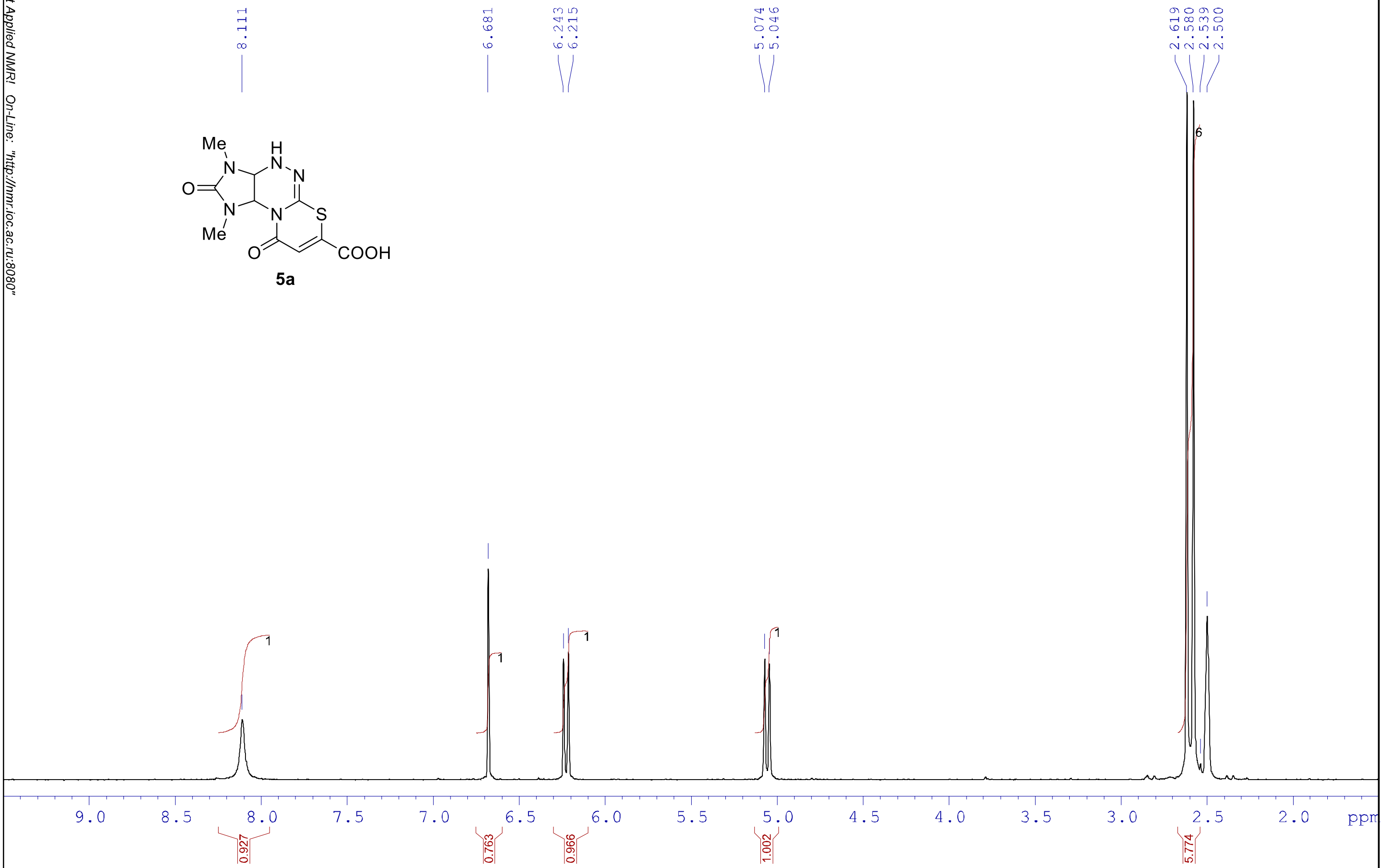

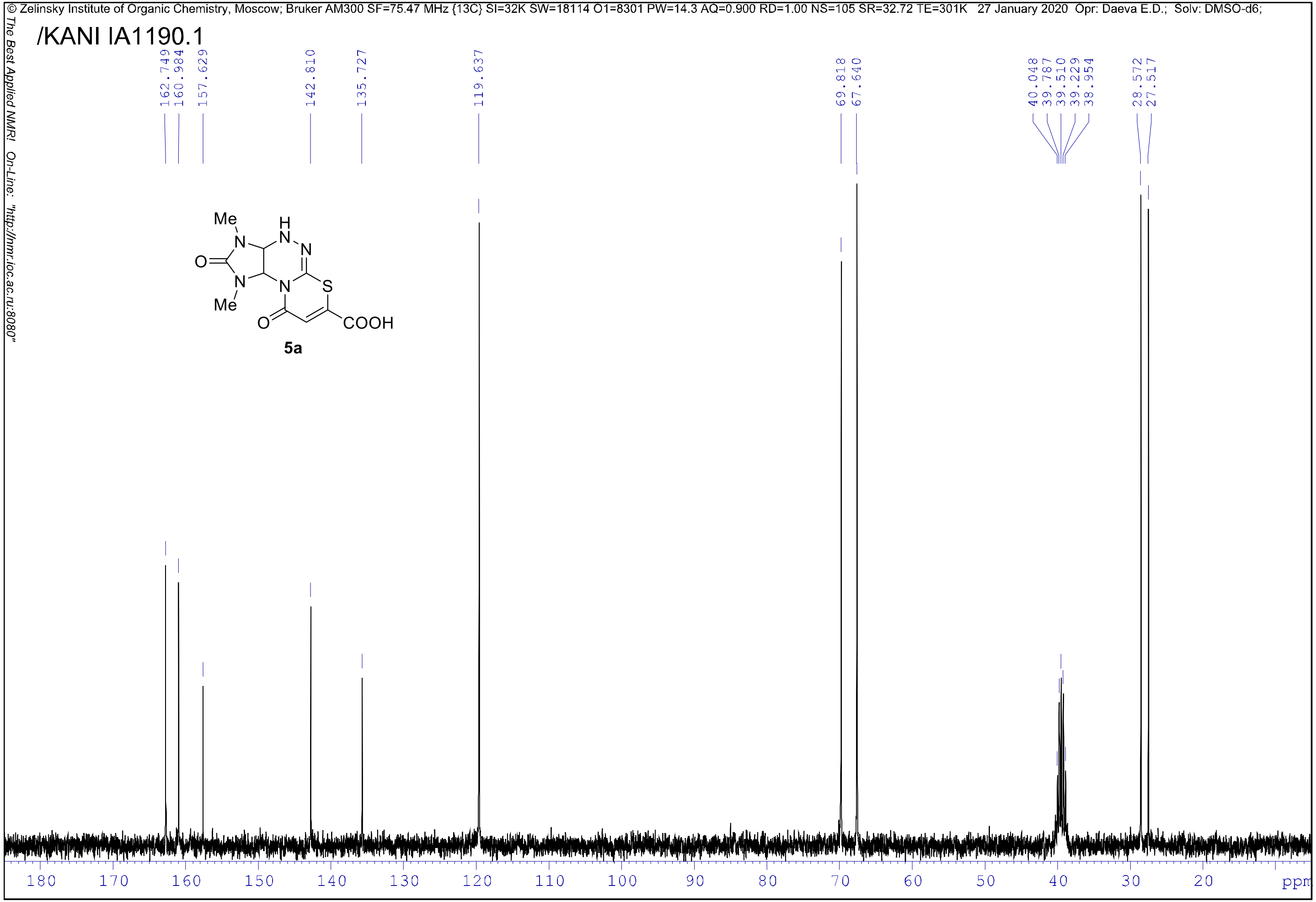

© Zelinsky Institute of Organic Chemistry, Moscow; Bruker AM300 SF=75.47 MHz {13C} SI=32K SW=18114 O1=8301 PW=14.3 AQ=0.900 RD=1.00 NS=105 SR=32.72 TE=301K 27 January 2020 Opr: Daeva E.D.; Solv: DMSO-d6;
The Best Applied NMR! On-Line: "http://nmr.ioc.ac.ru:8080"
/KANI IA1190.1
162.749
160.984
157.629
142.810
135.727
119.637
69.818
67.640
40.048
39.787
39.510
39.229
38.954
28.572
27.517
Me
H
N
N
O
N
N
N
S
Me
O
COOH
5a
180
170
160
150
140
130
120
110
100
90
80
70
60
50
40
30
20
ppm

© Zelinsky Institute of Organic Chemistry Moscow; Bruker AV600 SF=600.13 MHz {1H-13C}HMBC SI=1024x512 SW=4496 O1=3193 PW=12.0 AQ=0.099 RD=0.80 NS=2x256 SR=8.34 TE=306K 1 February 2021 Opr: Strelenko Yu.A.; Solv: DMSO-d6;

/KANI IA1190 {1H-13C} HMBC

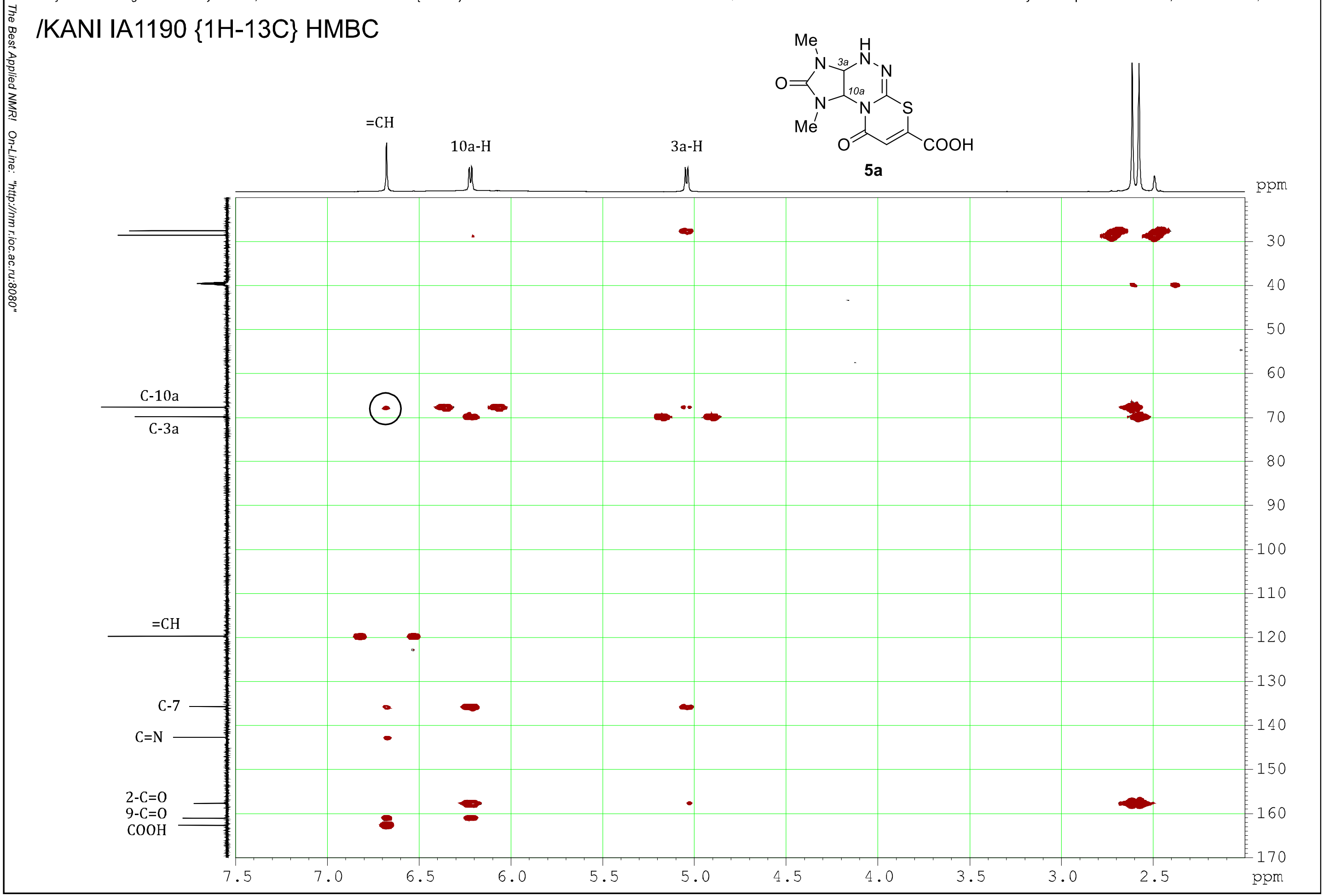

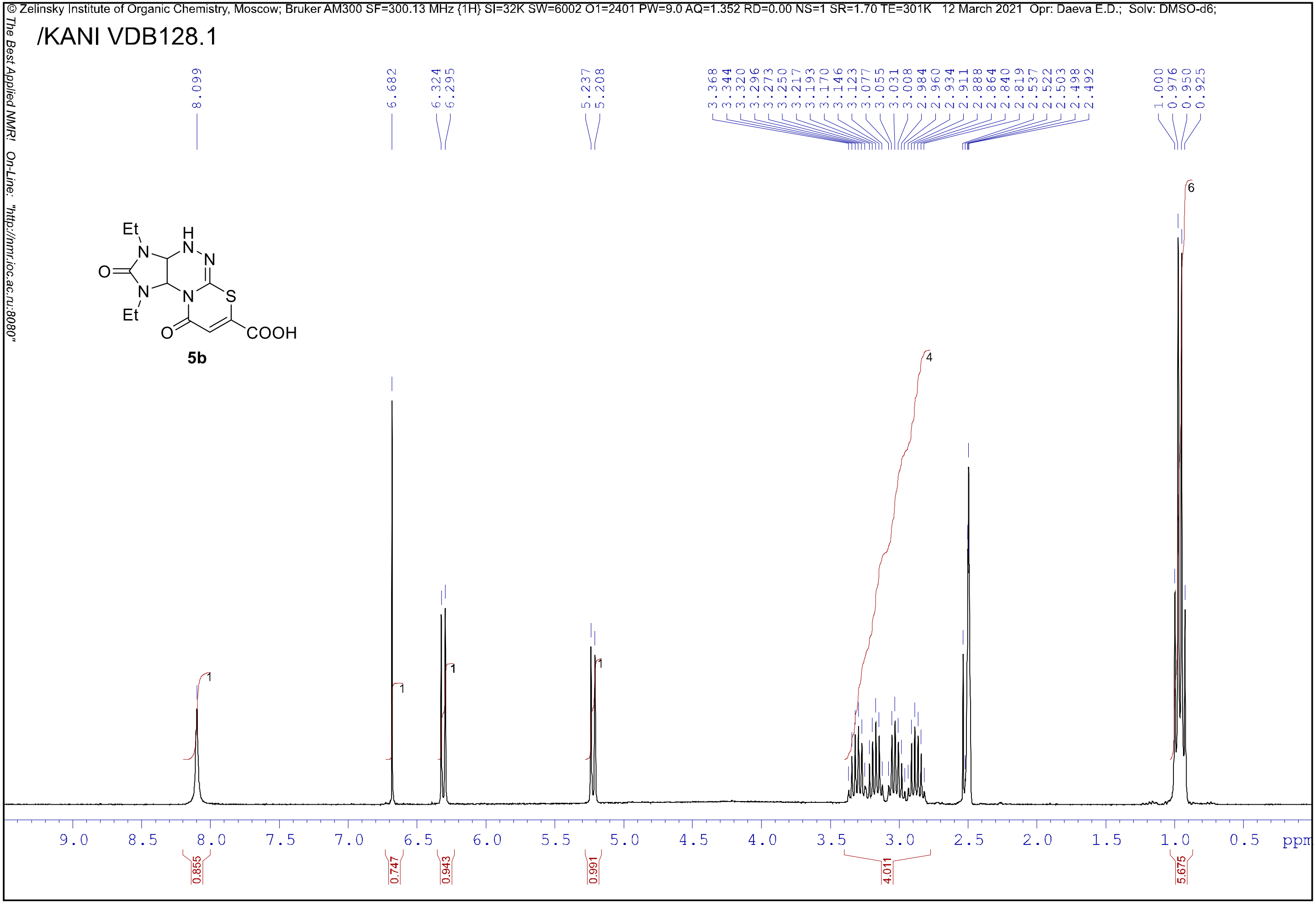
© Zelinsky Institute of Organic Chemistry, Moscow; Bruker AM300 SF=300.13 MHz {1H} SI=32K SW=6002 O1=2401 PW=9.0 AQ=1.352 RD=0.00 NS=1 SR=1.70 TE=301K 12 March 2021 Opr: Daeva E.D.; Solv: DMSO-d6;
The Best Applied NMR! On-Line: "http://nmr.ioc.ac.ru:8080"
/KANI VDB128.1
8.099
6.682
6.324
6.295
5.237
5.208
3.368
3.344
3.320
3.296
3.273
3.250
3.217
3.193
3.170
3.146
3.123
3.077
3.055
3.031
3.008
2.984
2.960
2.934
2.911
2.888
2.864
2.840
2.819
2.537
2.522
2.503
2.498
2.492
1.000
0.976
0.950
0.925
Et
N
H
N
N
O
N
Et
N
S
O
COOH
5b
ppm
0.855
0.747
0.943
0.991
4.011
5.675

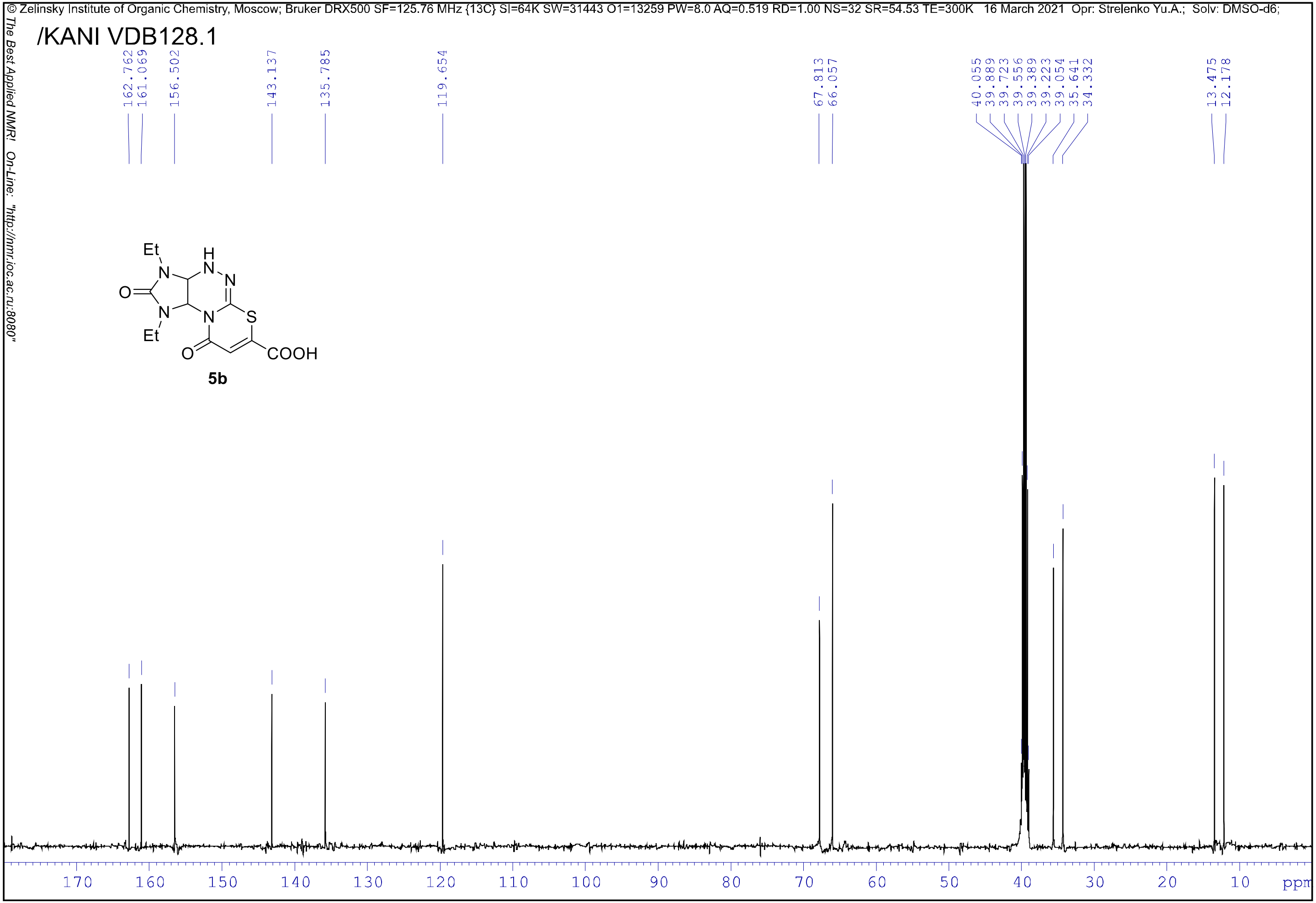
© Zelinsky Institute of Organic Chemistry, Moscow; Bruker DRX500 SF=125.76 MHz {13C} SI=64K SW=31443 O1=13259 PW=8.0 AQ=0.519 RD=1.00 NS=32 SR=54.53 TE=300K 16 March 2021 Opr: Strelenko Yu.A.; Solv: DMSO-d6;
/KANI VDB128.1
The Best Applied NMR! On-Line: "http://nmr.ioc.ac.ru:8080"
162.762
161.069
156.502
143.137
135.785
119.654
67.813
66.057
40.055
39.889
39.723
39.556
39.389
39.223
39.054
35.641
34.332
13.475
12.178
Et
H
N
N
N
O
N
N
S
Et
O
COOH
5b
170
160
150
140
130
120
110
100
90
80
70
60
50
40
30
20
10
ppm

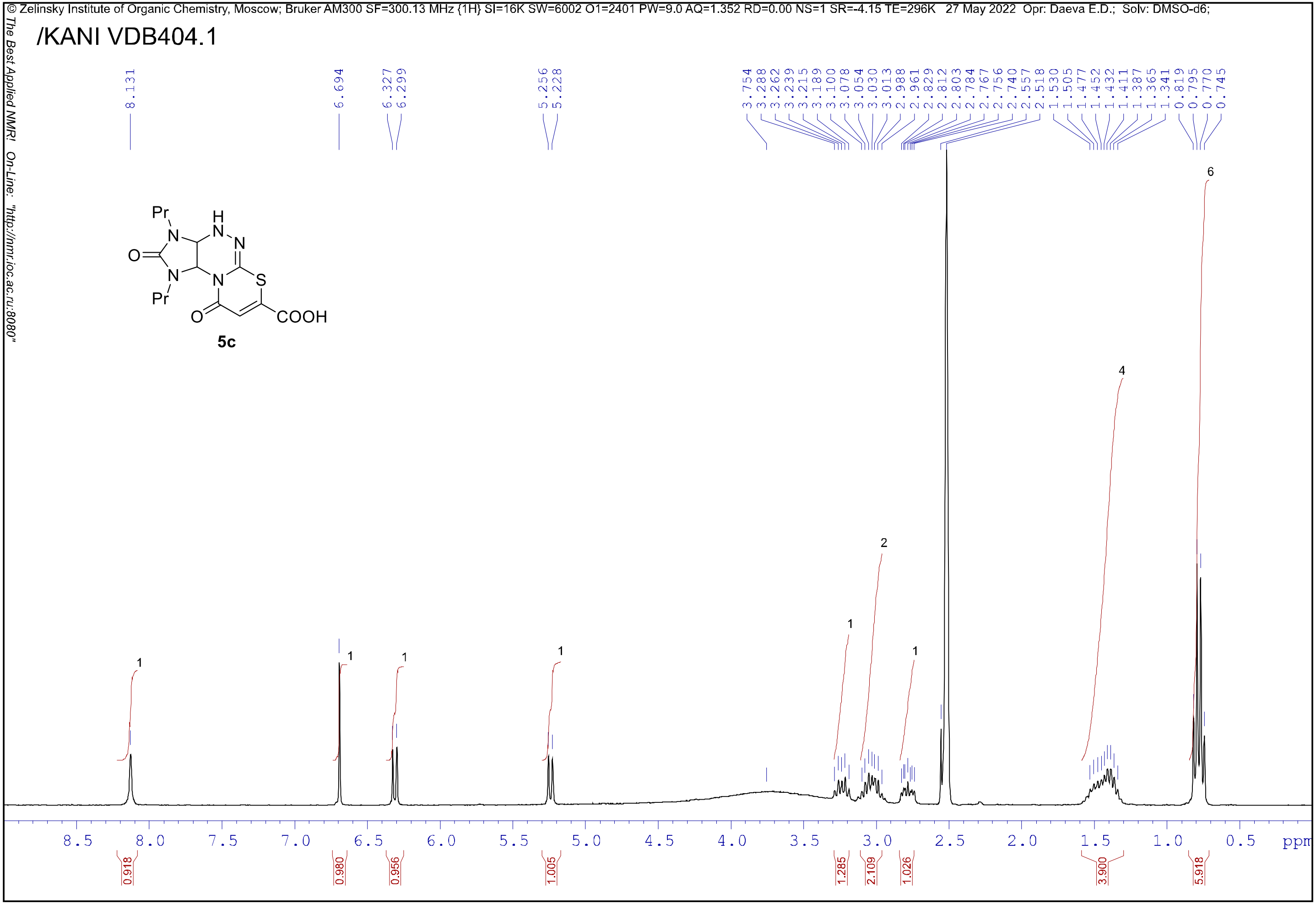

© Zelinsky Institute of Organic Chemistry, Moscow; Bruker AM300 SF=300.13 MHz {1H} SI=16K SW=6002 O1=2401 PW=9.0 AQ=1.352 RD=0.00 NS=1 SR=-4.15 TE=296K 27 May 2022 Opr: Daeva E.D.; Solv: DMSO-d6;
/KANI VDB404.1
The Best Applied NMR! On-Line: "http://nmr.ioc.ac.ru:8080"
8.131
6.694
6.327
6.299
5.256
5.228
3.754
3.288
3.262
3.239
3.215
3.189
3.100
3.078
3.054
3.030
3.013
2.988
2.961
2.829
2.812
2.803
2.784
2.767
2.756
2.740
2.557
2.518
1.530
1.505
1.477
1.452
1.432
1.411
1.387
1.365
1.341
0.819
0.795
0.770
0.745
Pr
H
N
O
S
COOH
5c
ppm
0.918
0.980
0.956
1.005
1.285
2.109
1.026
3.900
5.918

© Zelinsky Institute of Organic Chemistry, Moscow; Bruker AM300 SF=75.47 MHz {13C} SI=32K SW=18114 O1=8301 PW=14.3 AQ=0.901 RD=1.00 NS=414 SR=36.08 TE=297K 11 May 2022 Opr: Daeva E.D.; Solv: DMSO-d6;

The Best Applied NMR! On-Line: "http://nmr.ioc.ac.ru:8080"

/KANI VDB389.1

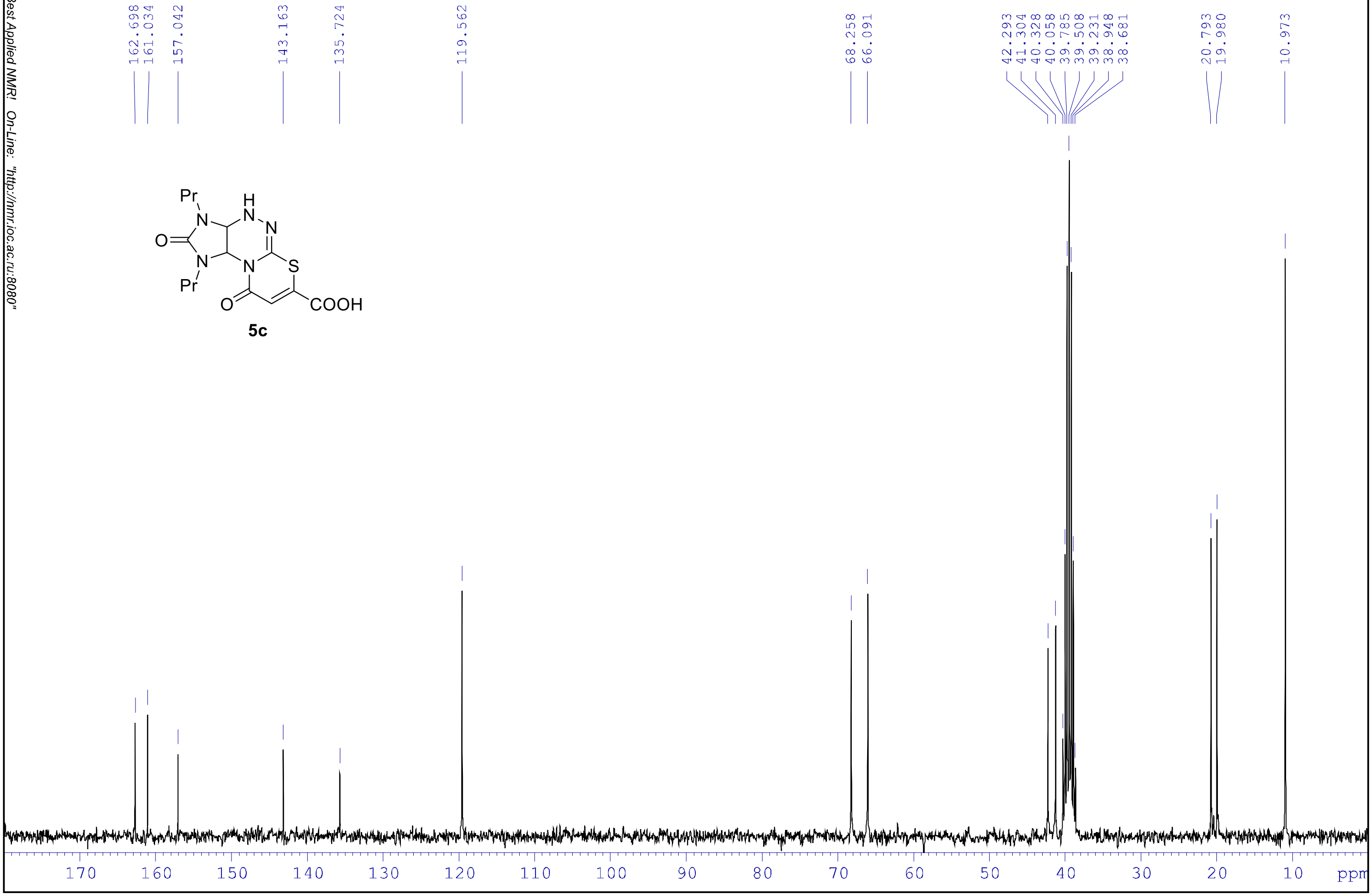

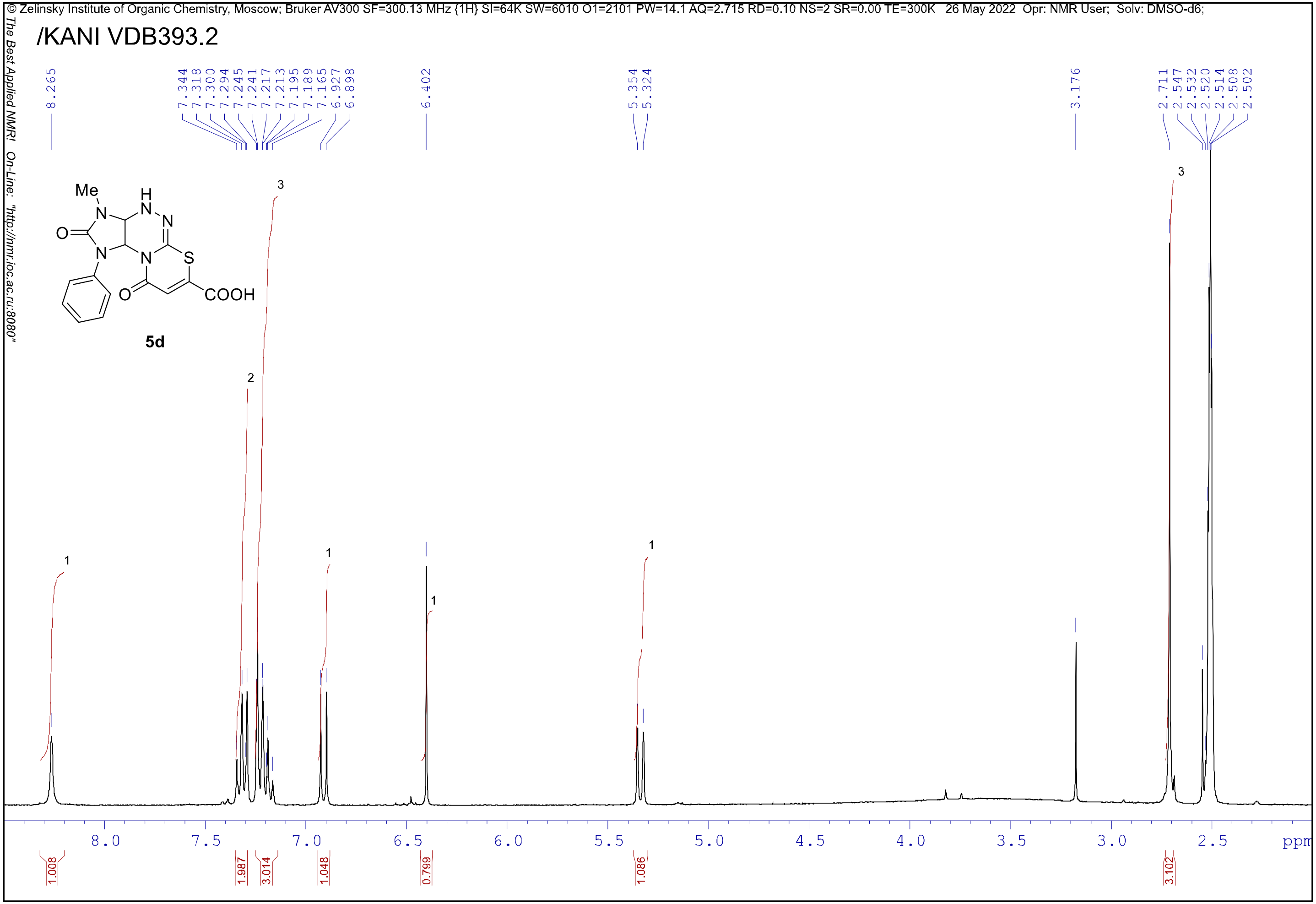
© Zelinsky Institute of Organic Chemistry, Moscow; Bruker AV300 SF=300.13 MHz {1H} SI=64K SW=6010 O1=2101 PW=14.1 AQ=2.715 RD=0.10 NS=2 SR=0.00 TE=300K 26 May 2022 Opr: NMR User; Solv: DMSO-d6;
The Best Applied NMR! On-Line: "http://nmr.ioc.ac.ru:8080"
/KANI VDB393.2
8.265
7.344 7.318 7.300 7.294 7.245 7.241 7.217 7.213 7.195 7.189 7.165 6.927 6.898
6.402
5.354 5.324
3.176
2.711 2.547 2.532 2.520 2.514 2.508 2.502
Me
H
N
O
S
COOH
5d
1
2
3
8.0 7.5 7.0 6.5 6.0 5.5 5.0 4.5 4.0 3.5 3.0 2.5 ppm
1.008 1.987 3.014 1.048 0.799 1.086 3.102

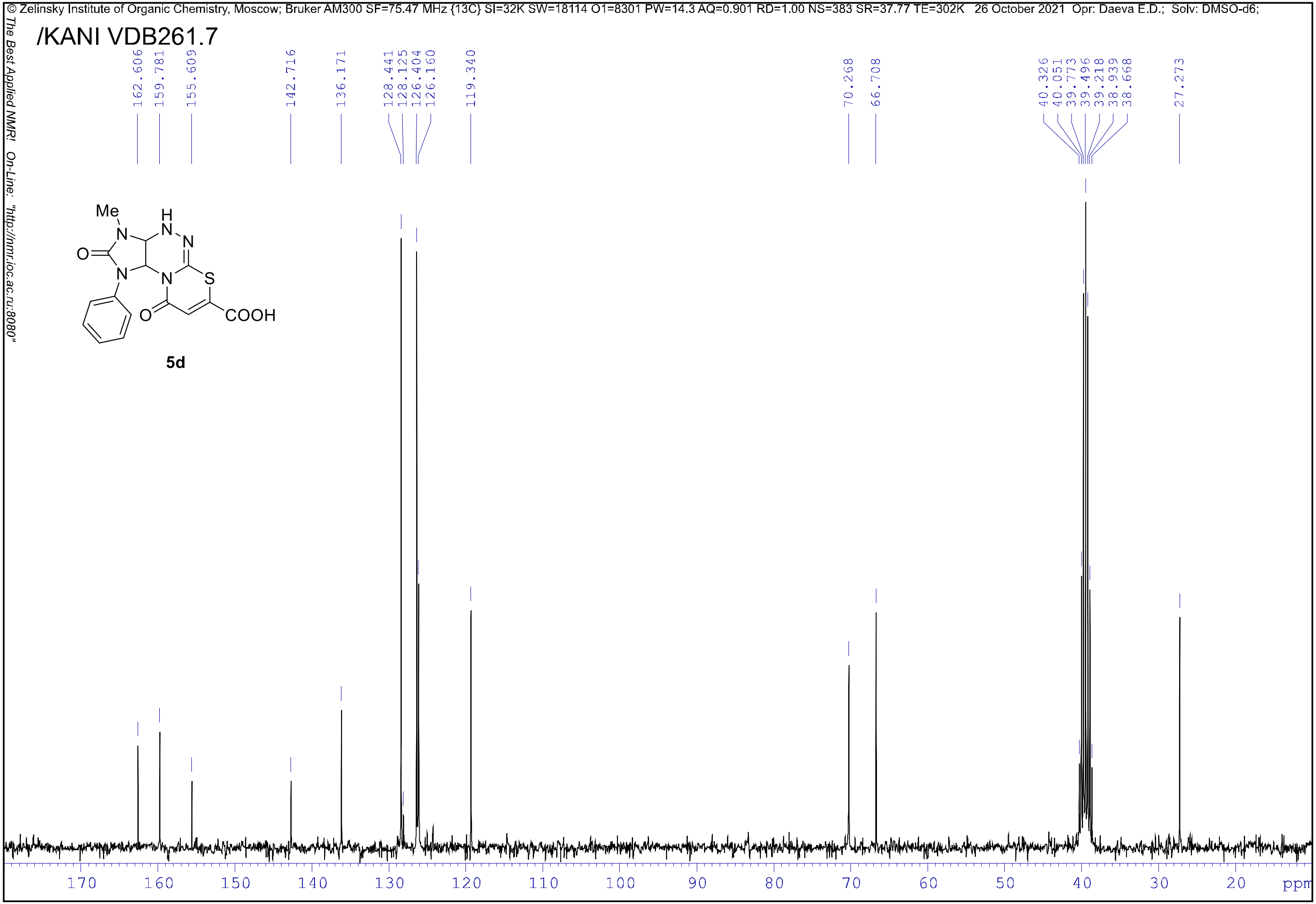
© Zelinsky Institute of Organic Chemistry, Moscow; Bruker AM300 SF=75.47 MHz {13C} SI=32K SW=18114 O1=8301 PW=14.3 AQ=0.901 RD=1.00 NS=383 SR=37.77 TE=302K 26 October 2021 Opr: Daeva E.D.; Solv: DMSO-d6;
The Best Applied NMR! On-Line: "http://nmr.ioc.ac.ru:8080"
/KANI VDB261.7
162.606
159.781
155.609
142.716
136.171
128.441
128.125
126.404
126.160
119.340
70.268
66.708
40.326
40.051
39.773
39.496
39.218
38.939
38.668
27.273
Me
H
N
O
S
COOH
5d
170 160 150 140 130 120 110 100 90 80 70 60 50 40 30 20 ppm

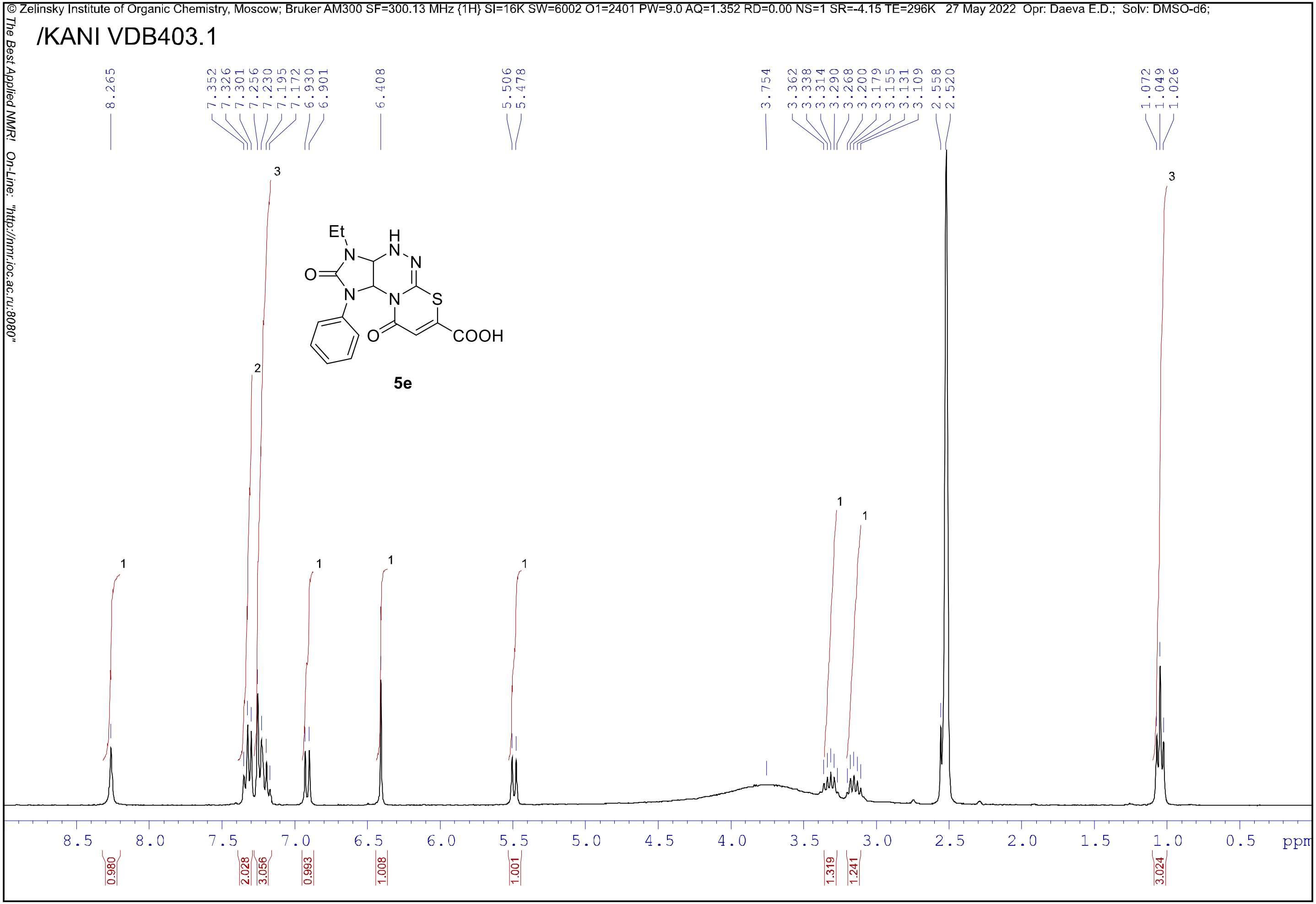
© Zelinsky Institute of Organic Chemistry, Moscow; Bruker AM300 SF=300.13 MHz {1H} SI=16K SW=6002 O1=2401 PW=9.0 AQ=1.352 RD=0.00 NS=1 SR=-4.15 TE=296K 27 May 2022 Opr: Daeva E.D.; Solv: DMSO-d6;
The Best Applied NMR! On-Line: "http://nmr.ioc.ac.ru:8080"
/KANI VDB403.1
8.265
7.352 7.326 7.301 7.256 7.230 7.195 7.172 6.930 6.901
6.408
5.506 5.478
3.754
3.362 3.338 3.314 3.290 3.268 3.200 3.179 3.155 3.131 3.109 2.558 2.520
1.072 1.049 1.026
Et
H
N
O
S
COOH
5e
8.5 8.0 7.5 7.0 6.5 6.0 5.5 5.0 4.5 4.0 3.5 3.0 2.5 2.0 1.5 1.0 0.5 ppm
0.980 2.028 3.056 0.993 1.008 1.001 1.319 1.241 3.024

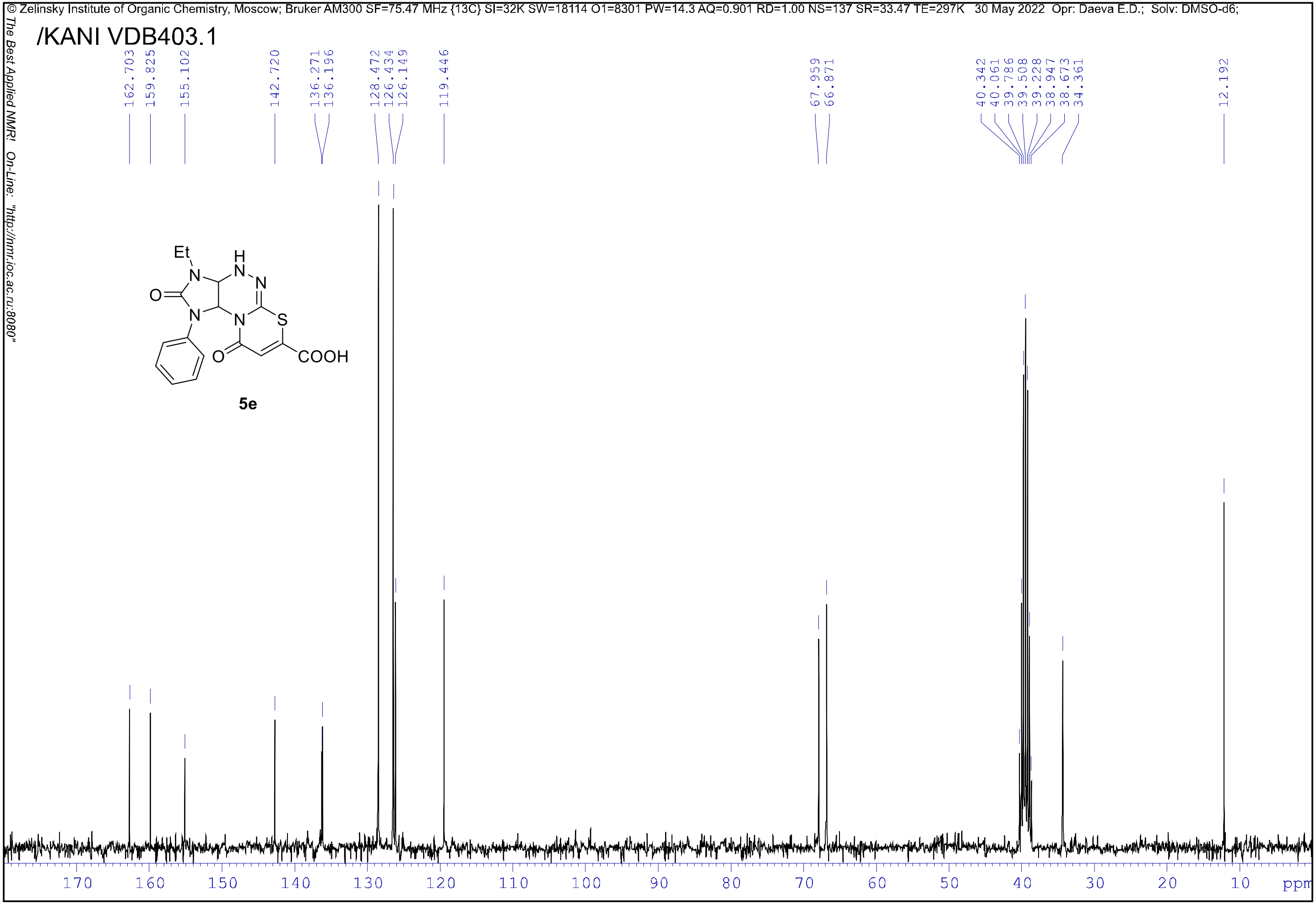

© Zelinsky Institute of Organic Chemistry, Moscow; Bruker AM300 SF=75.47 MHz {13C} SI=32K SW=18114 O1=8301 PW=14.3 AQ=0.901 RD=1.00 NS=137 SR=33.47 TE=297K 30 May 2022 Opr: Daeva E.D.; Solv: DMSO-d6;
The Best Applied NMR! On-Line: "http://nmr.ioc.ac.ru:8080"
/KANI VDB403.1
162.703
159.825
155.102
142.720
136.271
136.196
128.472
126.434
126.149
119.446
67.959
66.871
40.342
40.061
39.786
39.508
39.228
38.947
38.673
34.361
12.192
Et
H
N
N
N
O
N
N
S
O
COOH
5e
170
160
150
140
130
120
110
100
90
80
70
60
50
40
30
20
10
ppm

© Zelinsky Institute of Organic Chemistry, Moscow; Bruker AV300 SF=300.13 MHz {1H} SI=64K SW=6010 O1=2101 PW=14.8 AQ=3.317 RD=0.10 NS=4 SR=0.00 TE=300K 16 June 2023 Opr: NMR User; Solv: DMSO-d6;
The Best Applied NMR! On-Line: "http://nmr.ioc.ac.ru:8080"

/KANI VDB589.1.1 Gazieva-20074

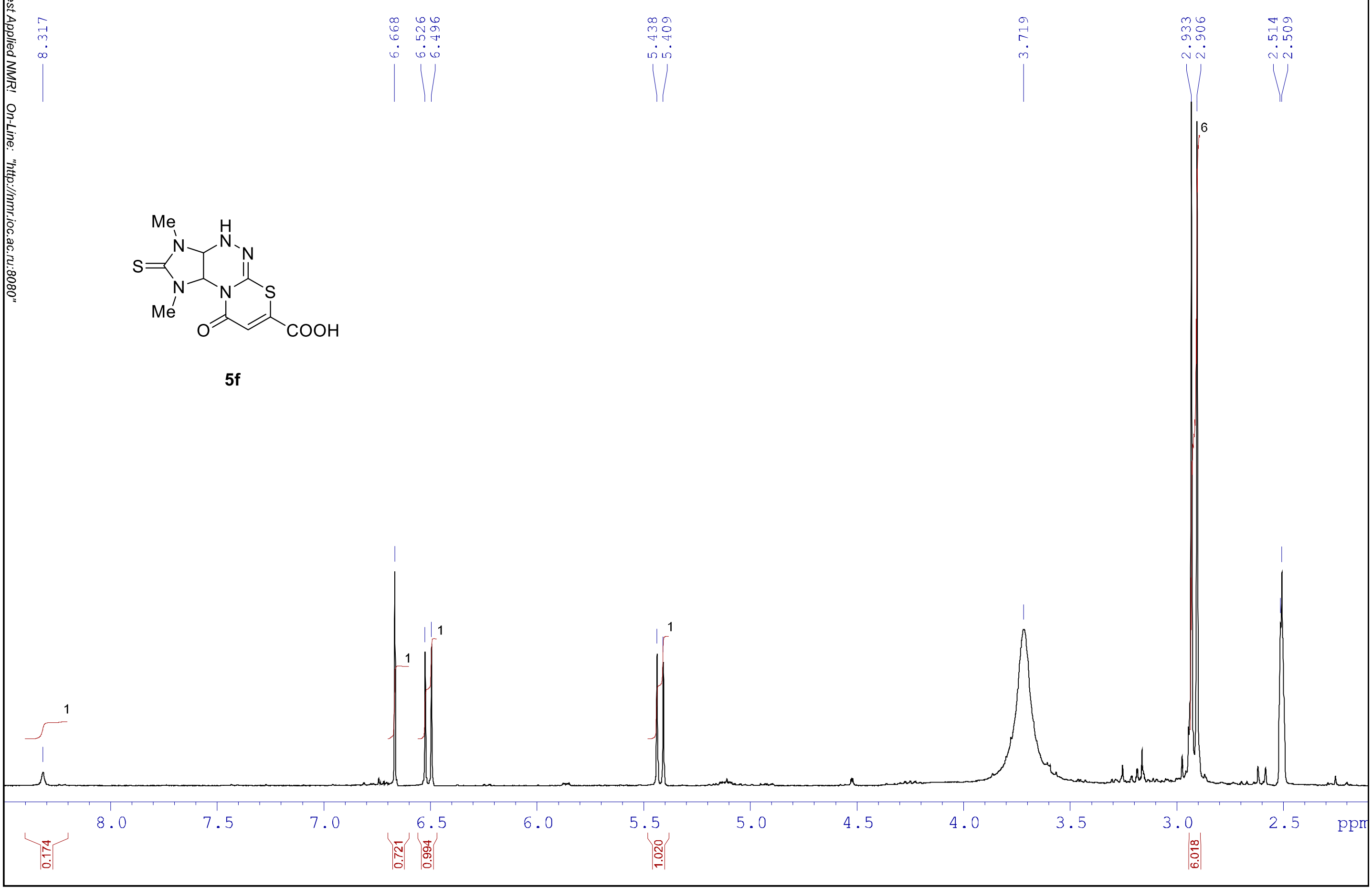

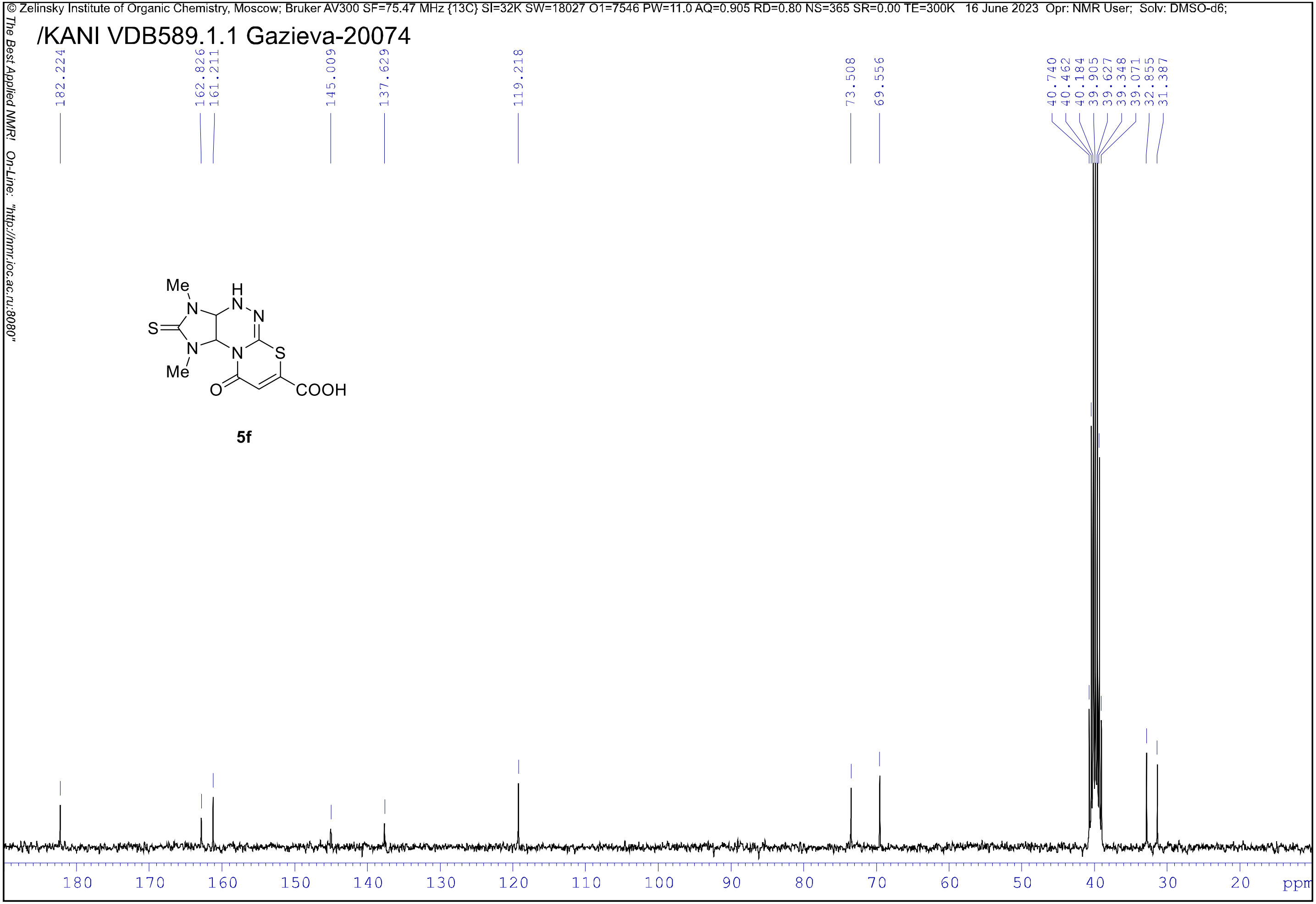
© Zelinsky Institute of Organic Chemistry, Moscow; Bruker AV300 SF=75.47 MHz {13C} SI=32K SW=18027 O1=7546 PW=11.0 AQ=0.905 RD=0.80 NS=365 SR=0.00 TE=300K 16 June 2023 Opr: NMR User; Solv: DMSO-d6;
The Best Applied NMR! On-Line: "http://nmr.ioc.ac.ru:8080"
/KANI VDB589.1.1 Gazieva-20074
182.224
162.826
161.211
145.009
137.629
119.218
73.508
69.556
40.740
40.462
40.184
39.905
39.627
39.348
39.071
32.855
31.387
Me
H
N
N
N
S
N
N
S
Me
O
COOH
5f
180 170 160 150 140 130 120 110 100 90 80 70 60 50 40 30 20 ppm

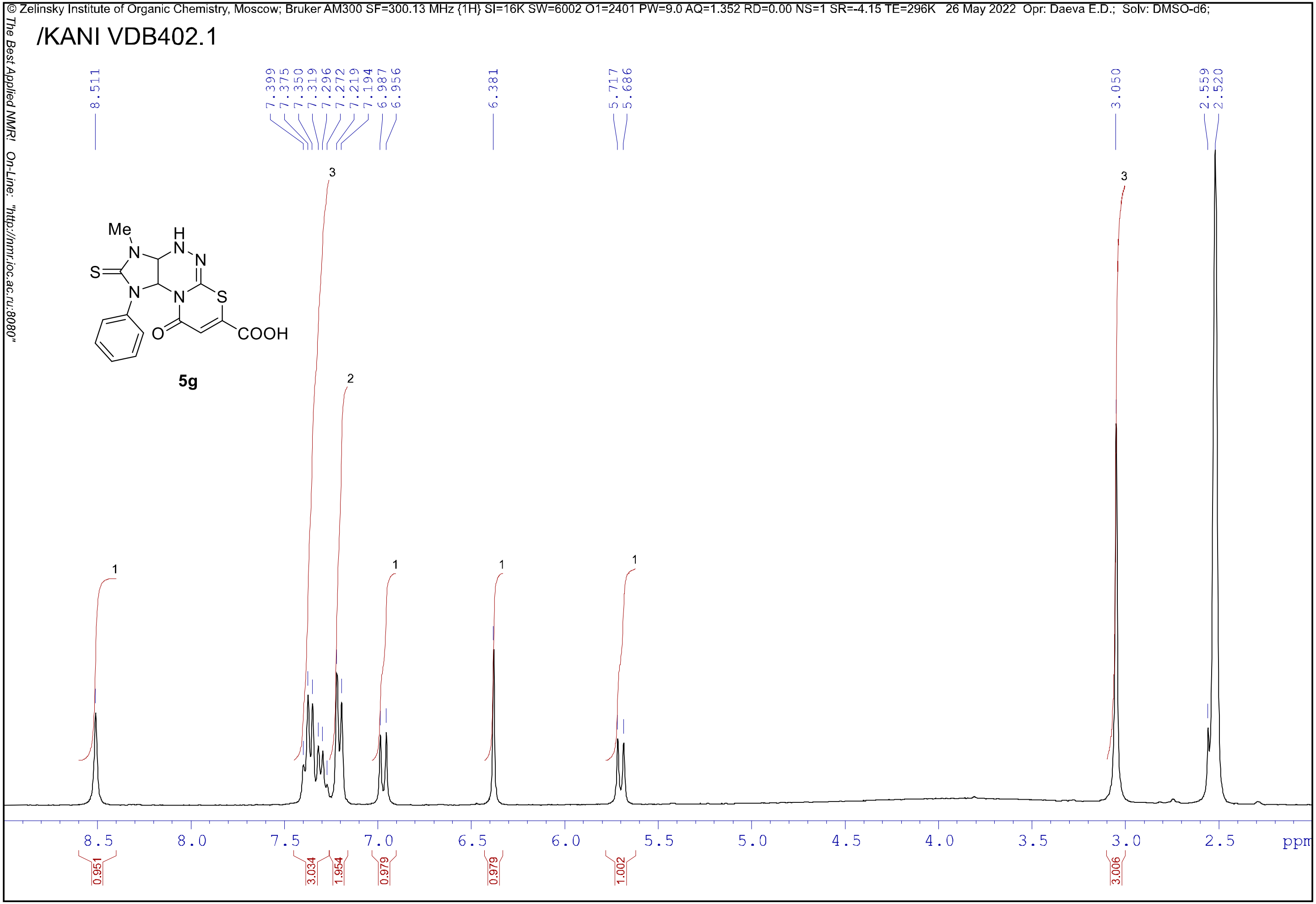

© Zelinsky Institute of Organic Chemistry, Moscow; Bruker AM300 SF=300.13 MHz {1H} SI=16K SW=6002 O1=2401 PW=9.0 AQ=1.352 RD=0.00 NS=1 SR=-4.15 TE=296K 26 May 2022 Opr: Daeva E.D.; Solv: DMSO-d6;
The Best Applied NMR! On-Line: "http://nmr.ioc.ac.ru:8080"
/KANI VDB402.1
8.511
7.399 7.375 7.350 7.319 7.296 7.272 7.219 7.194 6.987 6.956
6.381
5.717 5.686
3.050
2.559 2.520
Me
H
N
S
COOH
O
5g
0.951 3.034 1.954 0.979 0.979 1.002 3.006
8.5 8.0 7.5 7.0 6.5 6.0 5.5 5.0 4.5 4.0 3.5 3.0 2.5 ppm

© Zelinsky Institute of Organic Chemistry, Moscow; Bruker AM300 SF=75.47 MHz {13C} SI=32K SW=18114 O1=8301 PW=14.3 AQ=0.901 RD=1.00 NS=378 SR=34.38 TE=296K 27 May 2022 Opr: Daeva E.D.; Solv: DMSO-d6;

The Best Applied NMR! On-Line: "http://nmr.ioc.ac.ru:8080"

/KANI VDB402.1

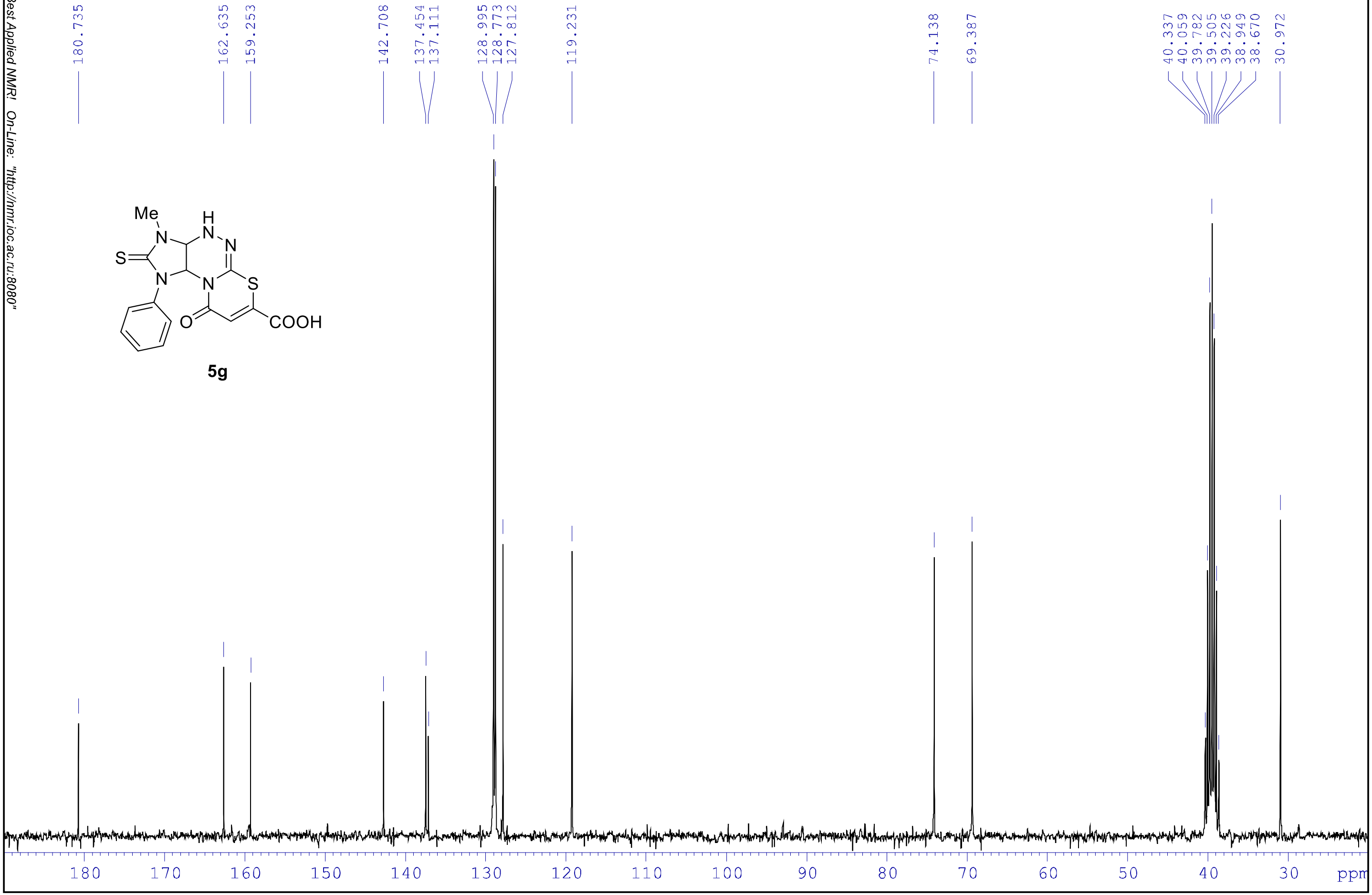

5g

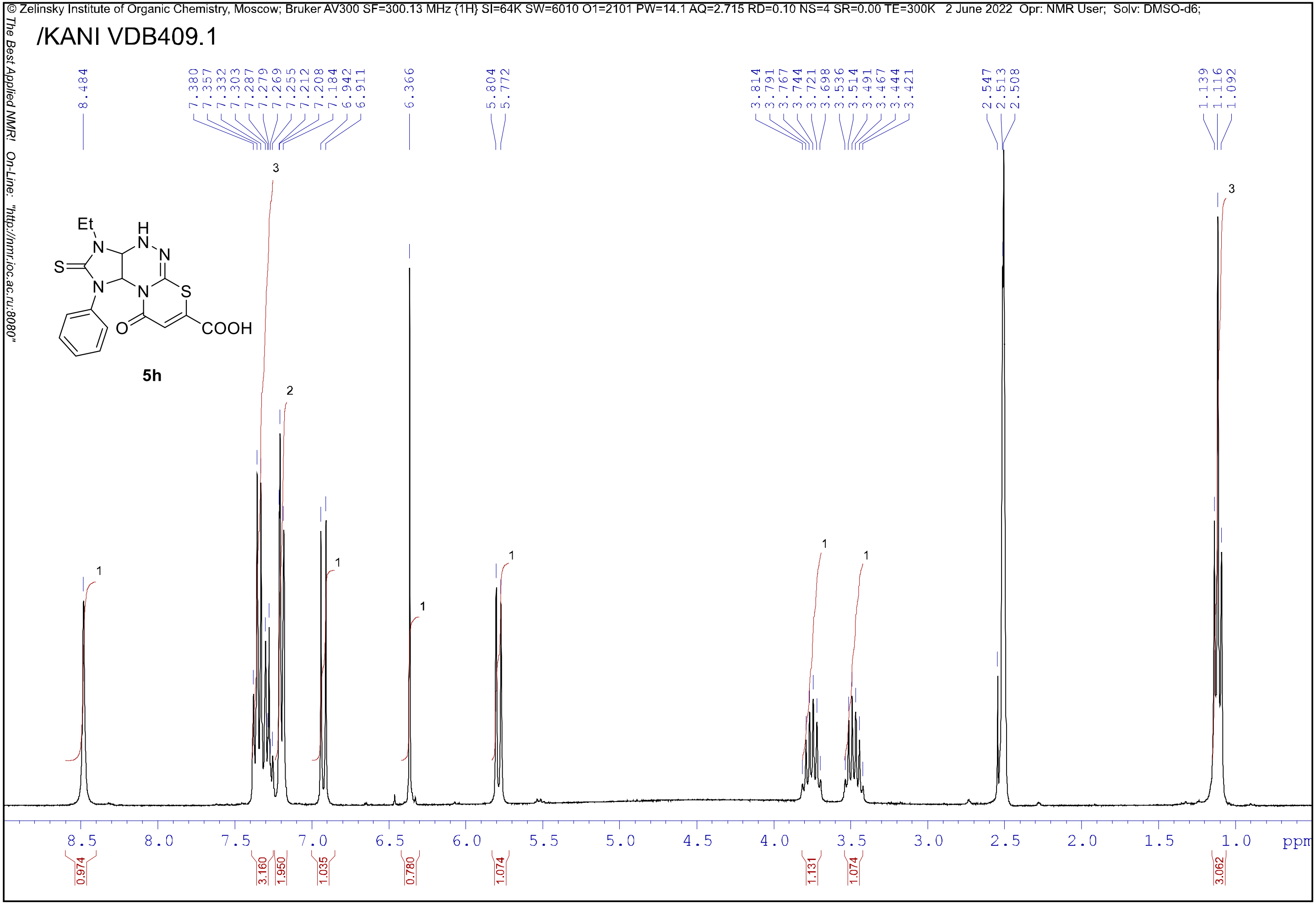
© Zelinsky Institute of Organic Chemistry, Moscow; Bruker AV300 SF=300.13 MHz {1H} SI=64K SW=6010 O1=2101 PW=14.1 AQ=2.715 RD=0.10 NS=4 SR=0.00 TE=300K 2 June 2022 Opr: NMR User; Solv: DMSO-d6;
The Best Applied NMR! On-Line: "http://nmr.ioc.ac.ru:8080"
/KANI VDB409.1
8.484
7.380 7.357 7.332 7.303 7.287 7.279 7.269 7.255 7.212 7.208 7.184 6.942 6.911
6.366
5.804 5.772
3.814 3.791 3.767 3.744 3.721 3.698 3.536 3.514 3.491 3.467 3.444 3.421
2.547 2.513 2.508
1.139 1.116 1.092
Et
H
N
S
O
COOH
5h
0.974 3.160 1.950 1.035 0.780 1.074 1.131 1.074 3.062
ppm

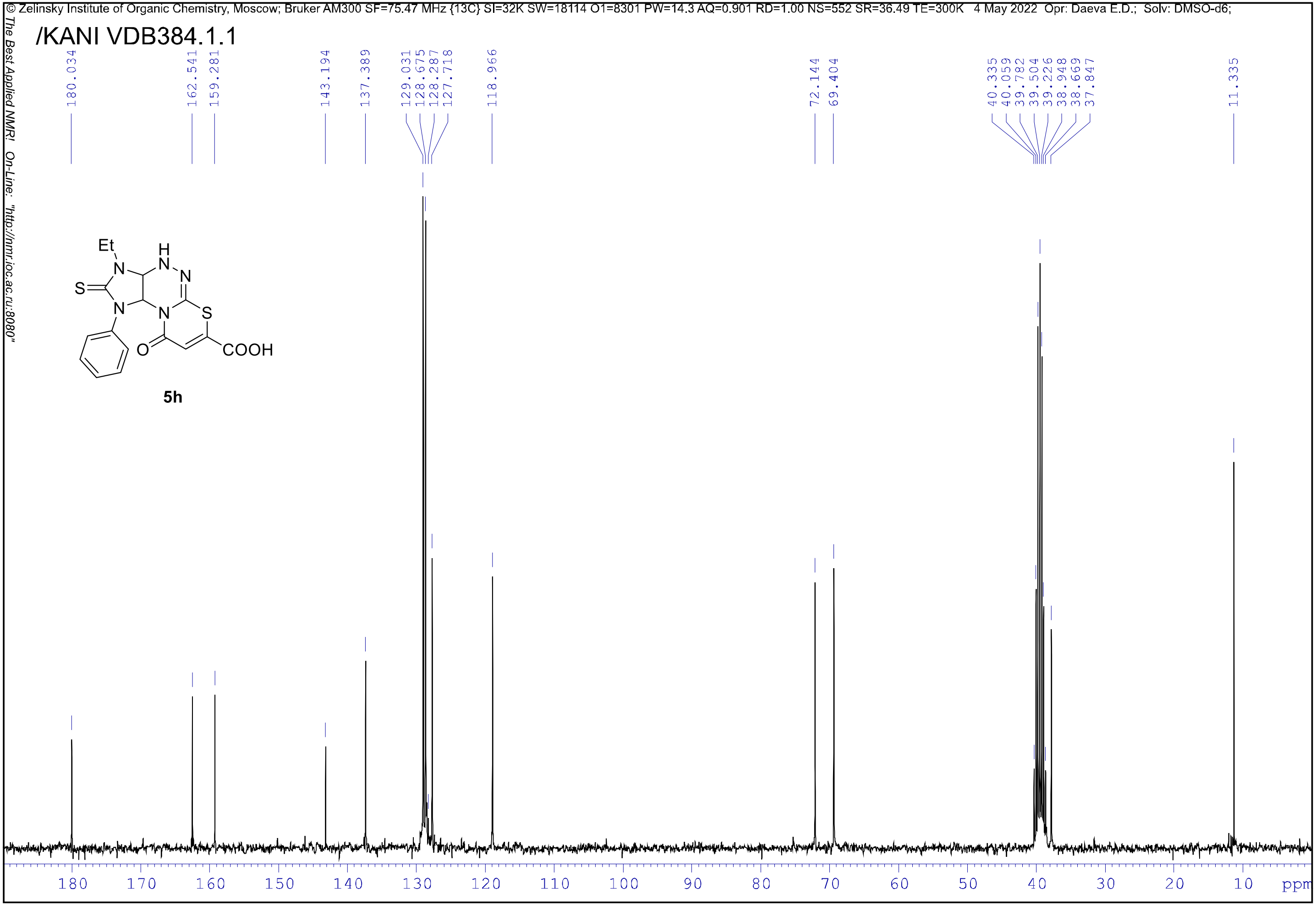
© Zelinsky Institute of Organic Chemistry, Moscow; Bruker AM300 SF=75.47 MHz {13C} SI=32K SW=18114 O1=8301 PW=14.3 AQ=0.901 RD=1.00 NS=552 SR=36.49 TE=300K 4 May 2022 Opr: Daeva E.D.; Solv: DMSO-d6;
The Best Applied NMR! On-Line: "http://nmr.ioc.ac.ru:8080"
/KANI VDB384.1.1
180.034
162.541
159.281
143.194
137.389
129.031
128.675
128.287
127.718
118.966
72.144
69.404
40.335
40.059
39.782
39.504
39.226
38.948
38.669
37.847
11.335
Et
H
N
N
N
S
N
N
S
O
COOH
5h
180
170
160
150
140
130
120
110
100
90
80
70
60
50
40
30
20
10
ppm

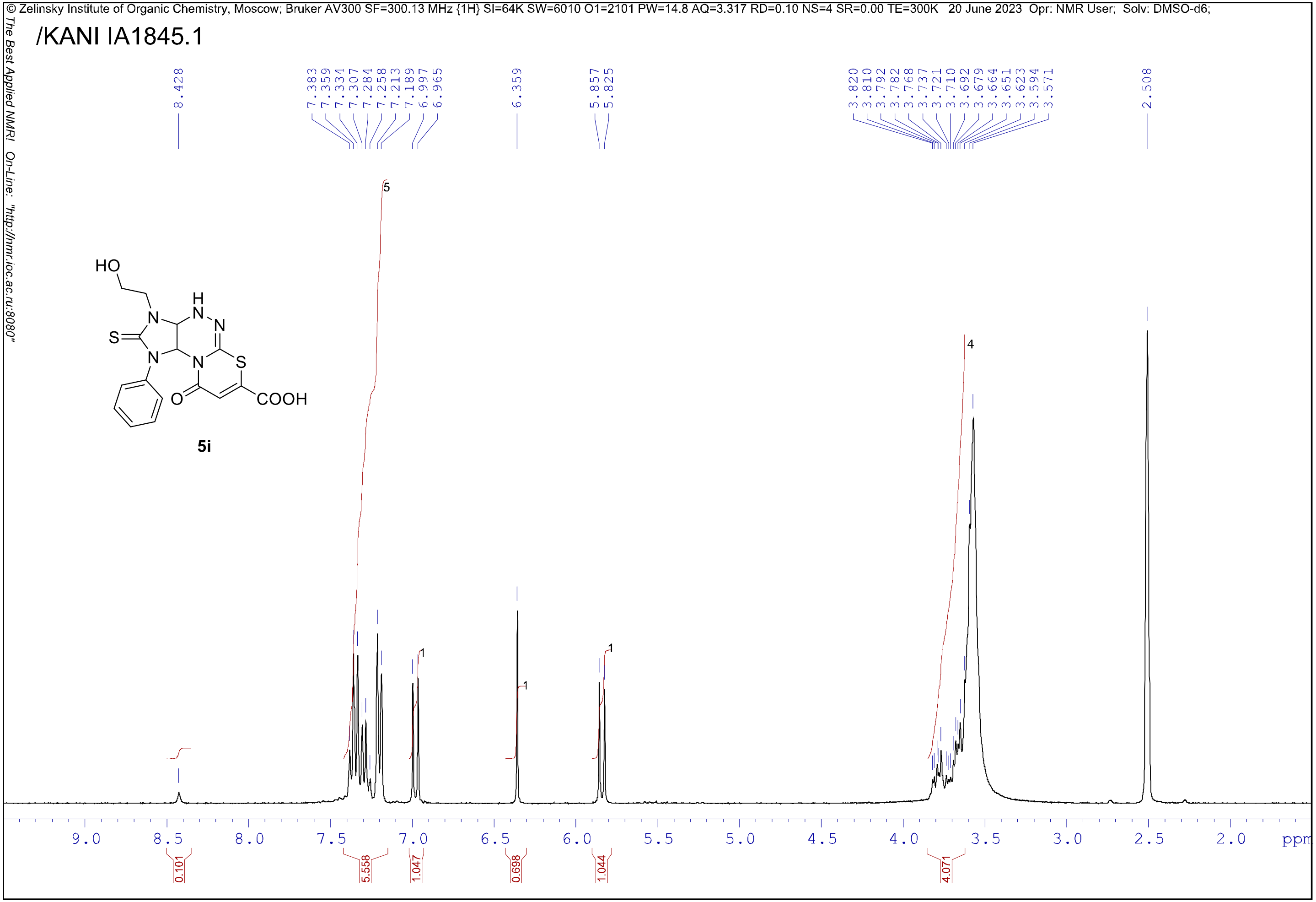
© Zelinsky Institute of Organic Chemistry, Moscow; Bruker AV300 SF=300.13 MHz {1H} SI=64K SW=6010 O1=2101 PW=14.8 AQ=3.317 RD=0.10 NS=4 SR=0.00 TE=300K 20 June 2023 Opr: NMR User; Solv: DMSO-d6;
The Best Applied NMR! On-Line: "http://nmr.ioc.ac.ru:8080"
/KANI IA1845.1
HO
H
N
S
COOH
O
5i
8.428
7.383 7.359 7.334 7.307 7.284 7.258 7.213 7.189 6.997 6.965
6.359
5.857 5.825
3.820 3.810 3.792 3.782 3.768 3.737 3.721 3.710 3.692 3.679 3.664 3.651 3.623 3.594 3.571
2.508
9.0 8.5 8.0 7.5 7.0 6.5 6.0 5.5 5.0 4.5 4.0 3.5 3.0 2.5 2.0 ppm
0.101 5.558 1.047 0.698 1.044 4.071

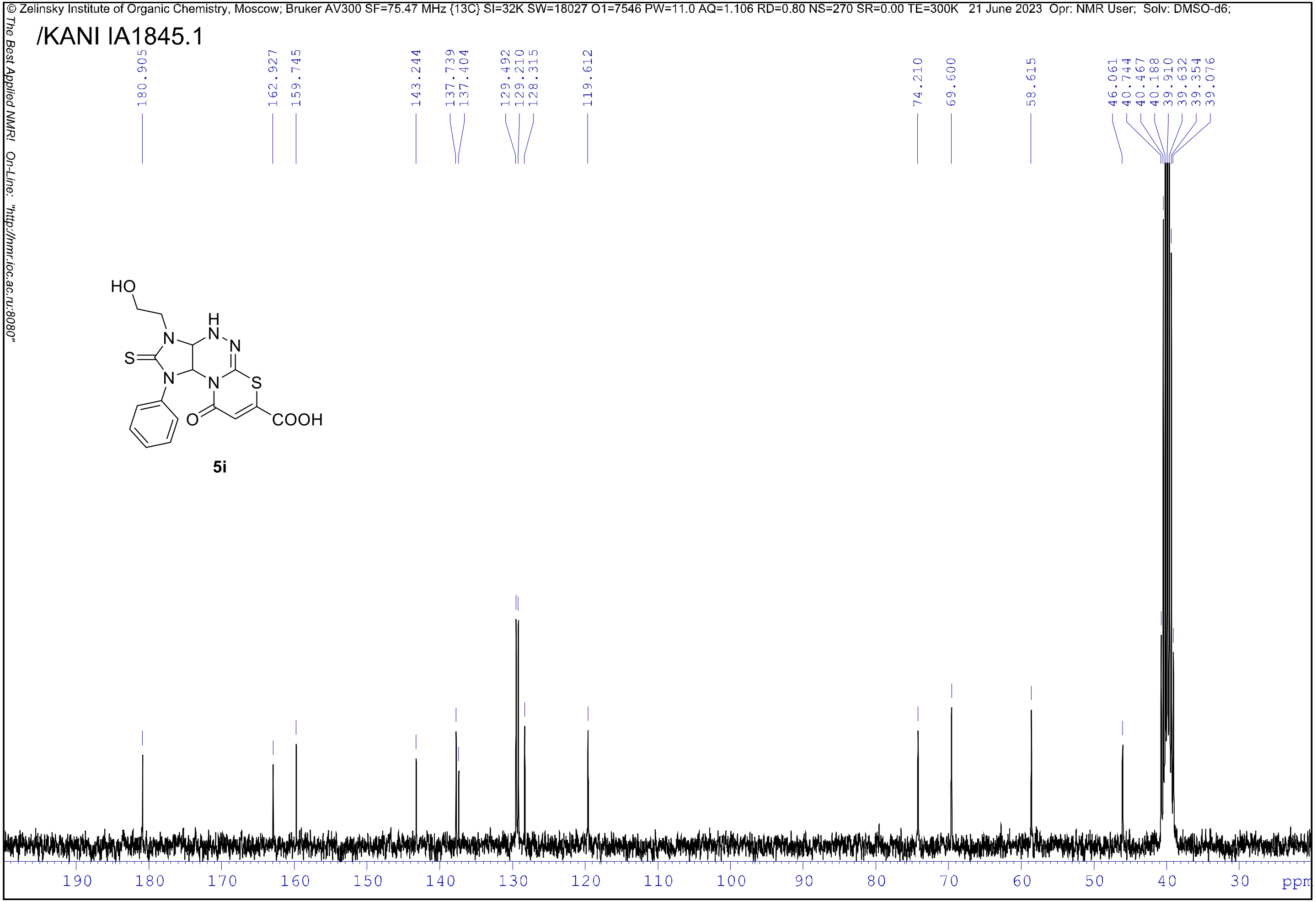
© Zelinsky Institute of Organic Chemistry, Moscow; Bruker AV300 SF=75.47 MHz {13C} SI=32K SW=18027 O1=7546 PW=11.0 AQ=1.106 RD=0.80 NS=270 SR=0.00 TE=300K 21 June 2023 Opr: NMR User; Solv: DMSO-d6;
The Best Applied NMR! On-Line: "http://nmr.ioc.ac.ru:8080"
/KANI IA1845.1
180.905
162.927
159.745
143.244
137.739
137.404
129.492
129.210
128.315
119.612
74.210
69.600
58.615
46.061
40.744
40.467
40.188
39.910
39.632
39.354
39.076
HO
H
N
N
N
S
N
N
S
O
COOH
5i
190
180
170
160
150
140
130
120
110
100
90
80
70
60
50
40
30
ppm

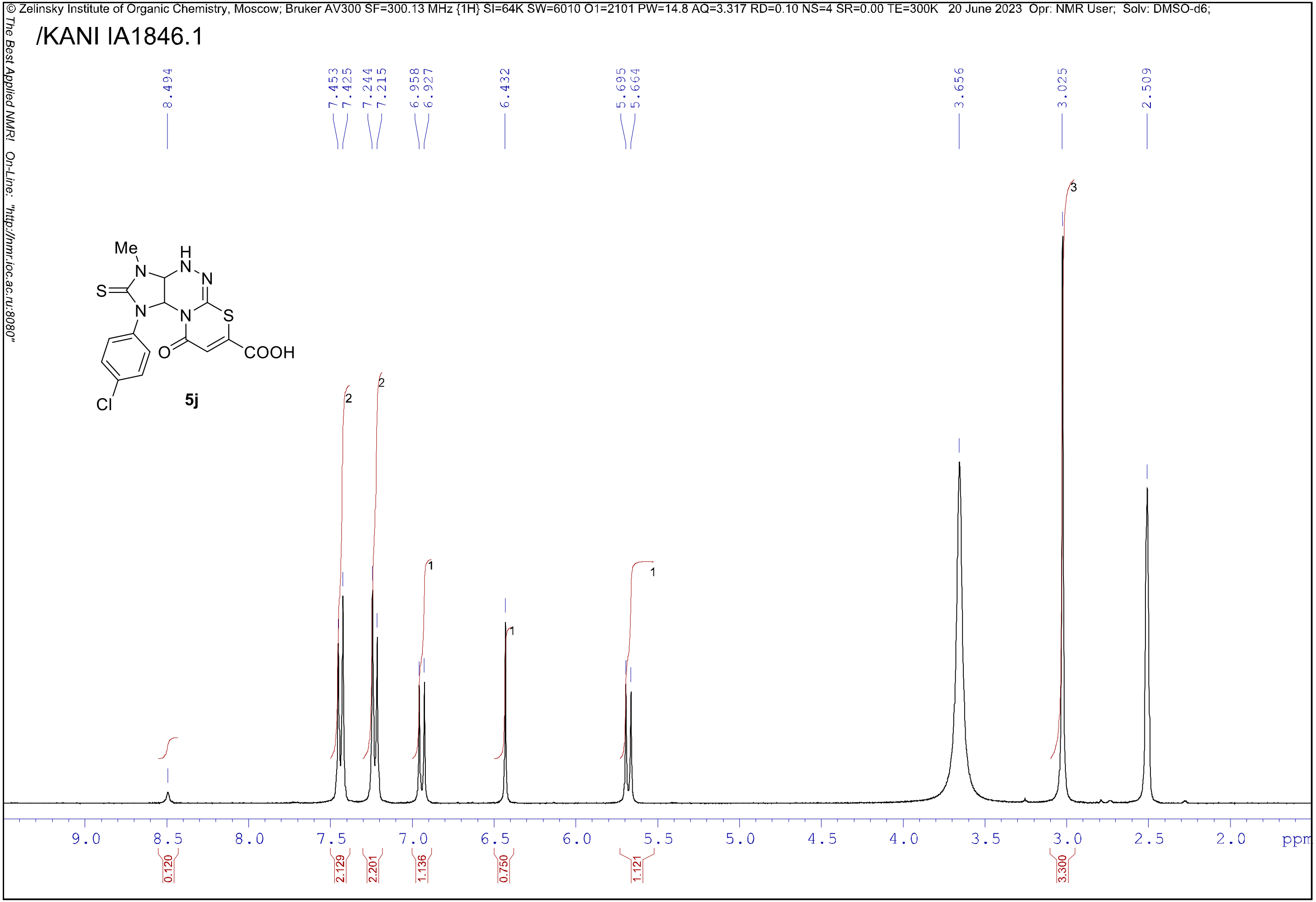
© Zelinsky Institute of Organic Chemistry, Moscow; Bruker AV300 SF=300.13 MHz {1H} SI=64K SW=6010 O1=2101 PW=14.8 AQ=3.317 RD=0.10 NS=4 SR=0.00 TE=300K 20 June 2023 Opr: NMR User; Solv: DMSO-d6;
The Best Applied NMR! On-Line: "http://nmr.ioc.ac.ru:8080"
/KANI IA1846.1
8.494
7.453
7.425
7.244
7.215
6.958
6.927
6.432
5.695
5.664
3.656
3.025
2.509
Me
N
H
N
N
S
N
N
S
O
COOH
Cl
5j
9.0
8.5
8.0
7.5
7.0
6.5
6.0
5.5
5.0
4.5
4.0
3.5
3.0
2.5
2.0
ppm
0.120
2.129
2.201
1.136
0.750
1.121
3.300

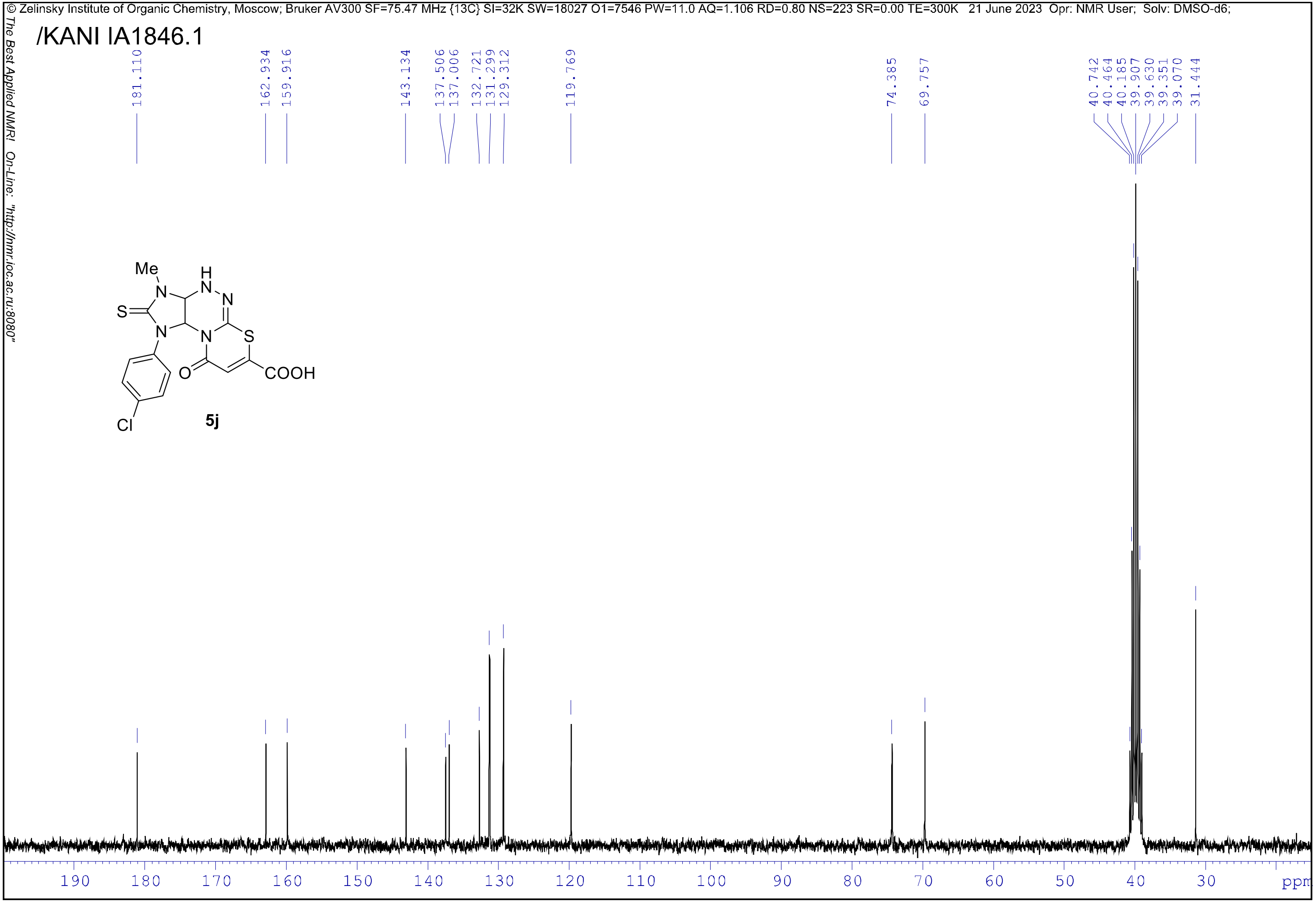
© Zelinsky Institute of Organic Chemistry, Moscow; Bruker AV300 SF=75.47 MHz {13C} SI=32K SW=18027 O1=7546 PW=11.0 AQ=1.106 RD=0.80 NS=223 SR=0.00 TE=300K 21 June 2023 Opr: NMR User; Solv: DMSO-d6;
The Best Applied NMR! On-Line: "http://nmr.ioc.ac.ru:8080"
/KANI IA1846.1
181.110
162.934
159.916
143.134
137.506
137.006
132.721
131.299
129.312
119.769
74.385
69.757
40.742
40.464
40.185
39.907
39.630
39.351
39.070
31.444
Me
H
N
S
O
COOH
Cl
5j
190 180 170 160 150 140 130 120 110 100 90 80 70 60 50 40 30 ppm

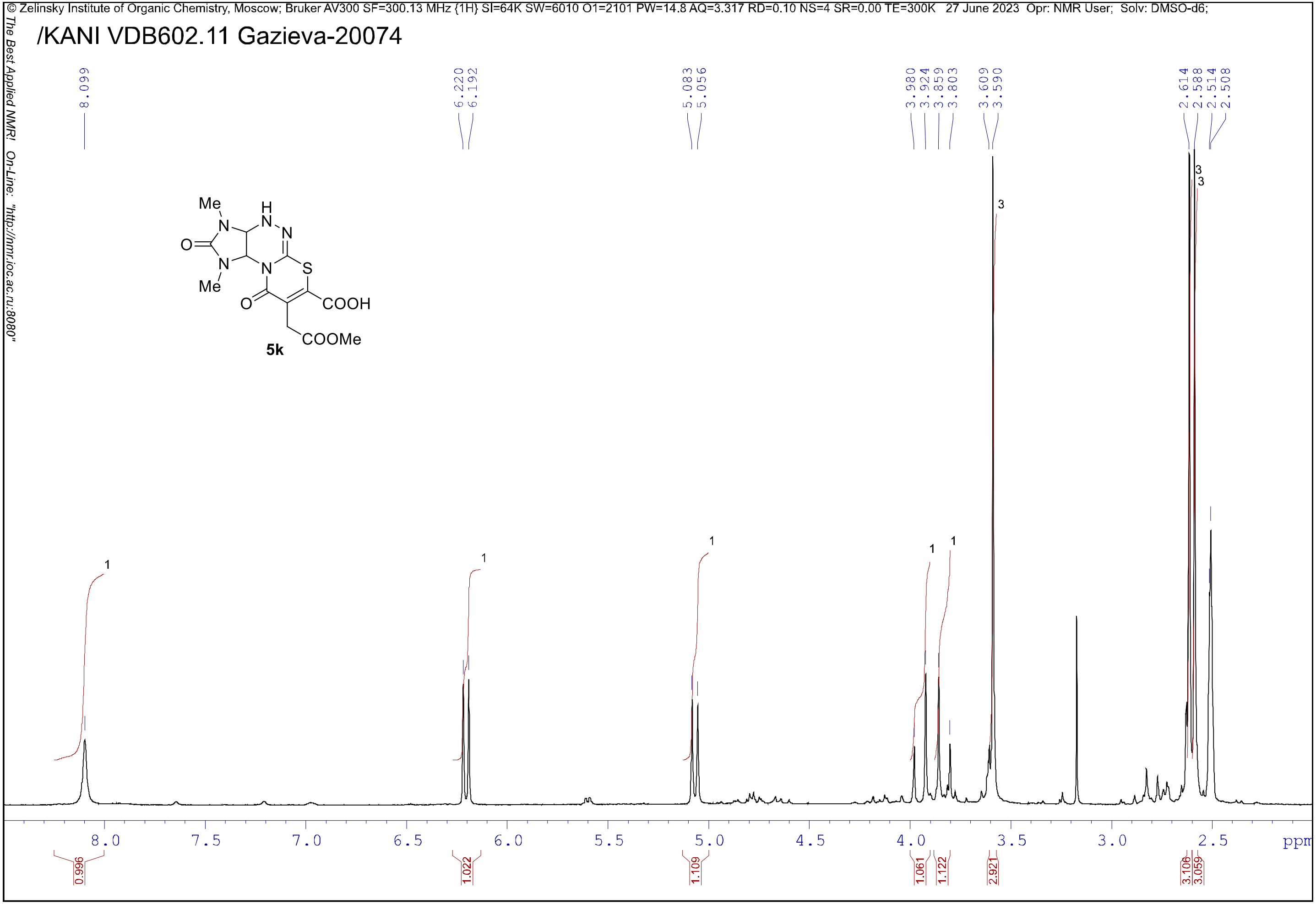
© Zelinsky Institute of Organic Chemistry, Moscow; Bruker AV300 SF=300.13 MHz {1H} SI=64K SW=6010 O1=2101 PW=14.8 AQ=3.317 RD=0.10 NS=4 SR=0.00 TE=300K 27 June 2023 Opr: NMR User; Solv: DMSO-d6;
The Best Applied NMR! On-Line: "http://nmr.ioc.ac.ru:8080"
/KANI VDB602.11 Gazieva-20074
8.099
6.220
6.192
5.083
5.056
3.980
3.924
3.859
3.803
3.609
3.590
2.614
2.588
2.514
2.508
Me
H
N
N
N
O
N
N
S
Me
O
COOH
COOMe
5k
8.0
7.5
7.0
6.5
6.0
5.5
5.0
4.5
4.0
3.5
3.0
2.5
ppm
0.996
1.022
1.109
1.061
1.122
2.921
3.106
3.059

© Zelinsky Institute of Organic Chemistry, Moscow; Bruker AV300 SF=75.47 MHz {13C} SI=32K SW=18027 O1=7546 PW=11.0 AQ=1.106 RD=0.80 NS=501 SR=0.00 TE=300K 26 June 2023 Opr: NMR User; Solv: DMSO-d6;

The Best Applied NMR! On-Line: "http://nmr.ioc.ac.ru:8080"

/KANI VDB602.1 Gazieva-20074

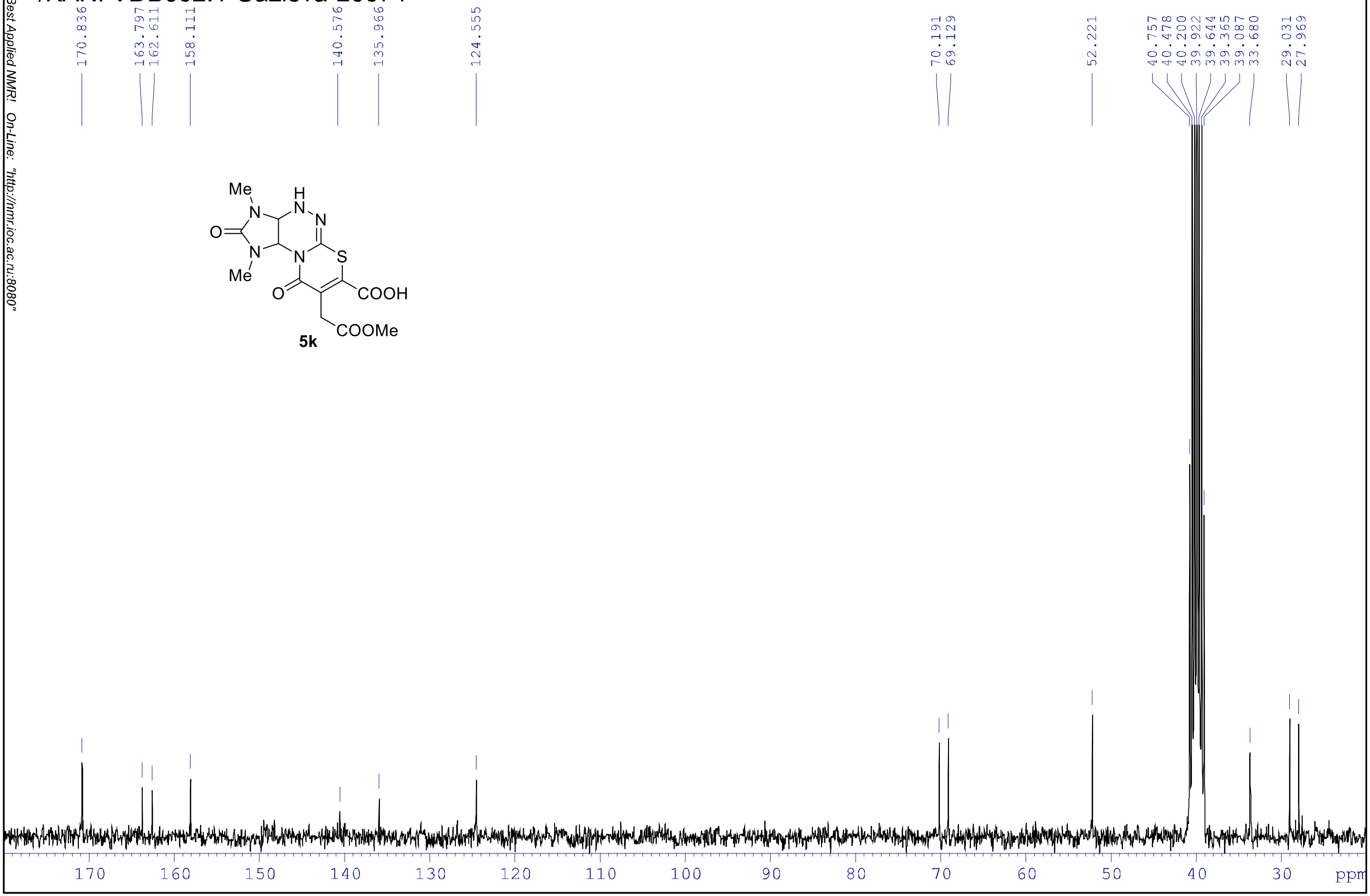

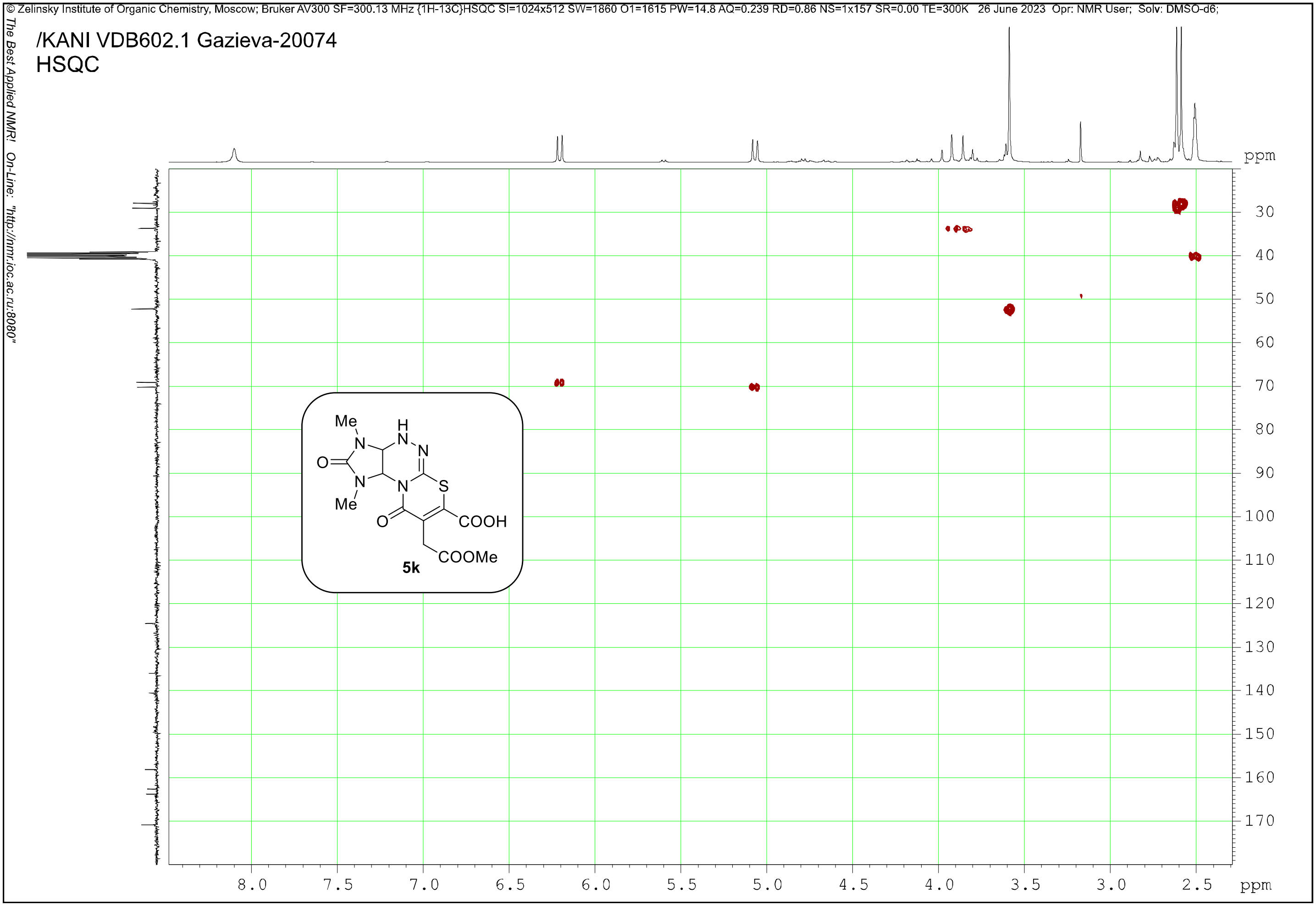
© Zelinsky Institute of Organic Chemistry, Moscow; Bruker AV300 SF=300.13 MHz {1H-13C}HSQC SI=1024x512 SW=1860 O1=1615 PW=14.8 AQ=0.239 RD=0.86 NS=1x157 SR=0.00 TE=300K 26 June 2023 Opr: NMR User; Solv: DMSO-d6;
The Best Applied NMR! On-Line: "http://nmr.ioc.ac.ru:8080"
/KANI VDB602.1 Gazieva-20074
HSQC
ppm
30
40
50
60
70
80
90
100
110
120
130
140
150
160
170
8.0
7.5
7.0
6.5
6.0
5.5
5.0
4.5
4.0
3.5
3.0
2.5
ppm
Me
H
N
N
O
N
N
S
Me
O
COOH
COOMe
5k

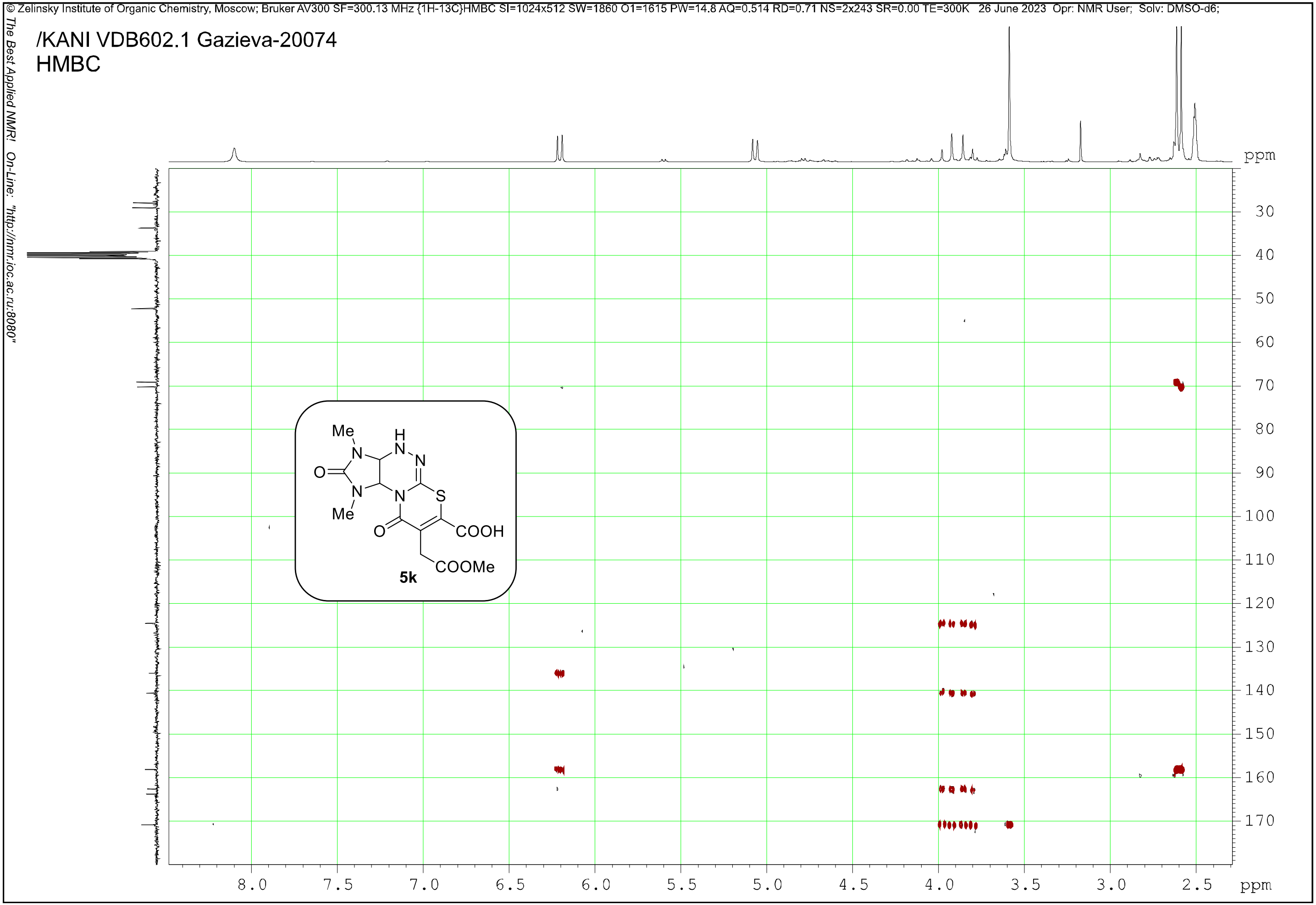
© Zelinsky Institute of Organic Chemistry, Moscow; Bruker AV300 SF=300.13 MHz {1H-13C}HMBC SI=1024x512 SW=1860 O1=1615 PW=14.8 AQ=0.514 RD=0.71 NS=2x243 SR=0.00 TE=300K 26 June 2023 Opr: NMR User; Solv: DMSO-d6;
The Best Applied NMR! On-Line: "http://nmr.ioc.ac.ru:8080"
/KANI VDB602.1 Gazieva-20074
HMBC
ppm
30
40
50
60
70
80
90
100
110
120
130
140
150
160
170
8.0
7.5
7.0
6.5
6.0
5.5
5.0
4.5
4.0
3.5
3.0
2.5
ppm
Me
H
N
N
N
O
N
N
S
Me
O
COOH
COOMe
5k

© Zelinsky Institute of Organic Chemistry, Moscow; Bruker AV300 SF=300.13 MHz {1H} SI=64K SW=6010 O1=2101 PW=14.8 AQ=3.317 RD=0.10 NS=6 SR=0.00 TE=300K 22 June 2023 Opr: NMR User; Solv: DMSO-d6;

The Best Applied NMR! On-Line: "http://nmr.ioc.ac.ru:8080"

/KANI VDB596.3 Gazieva-20074

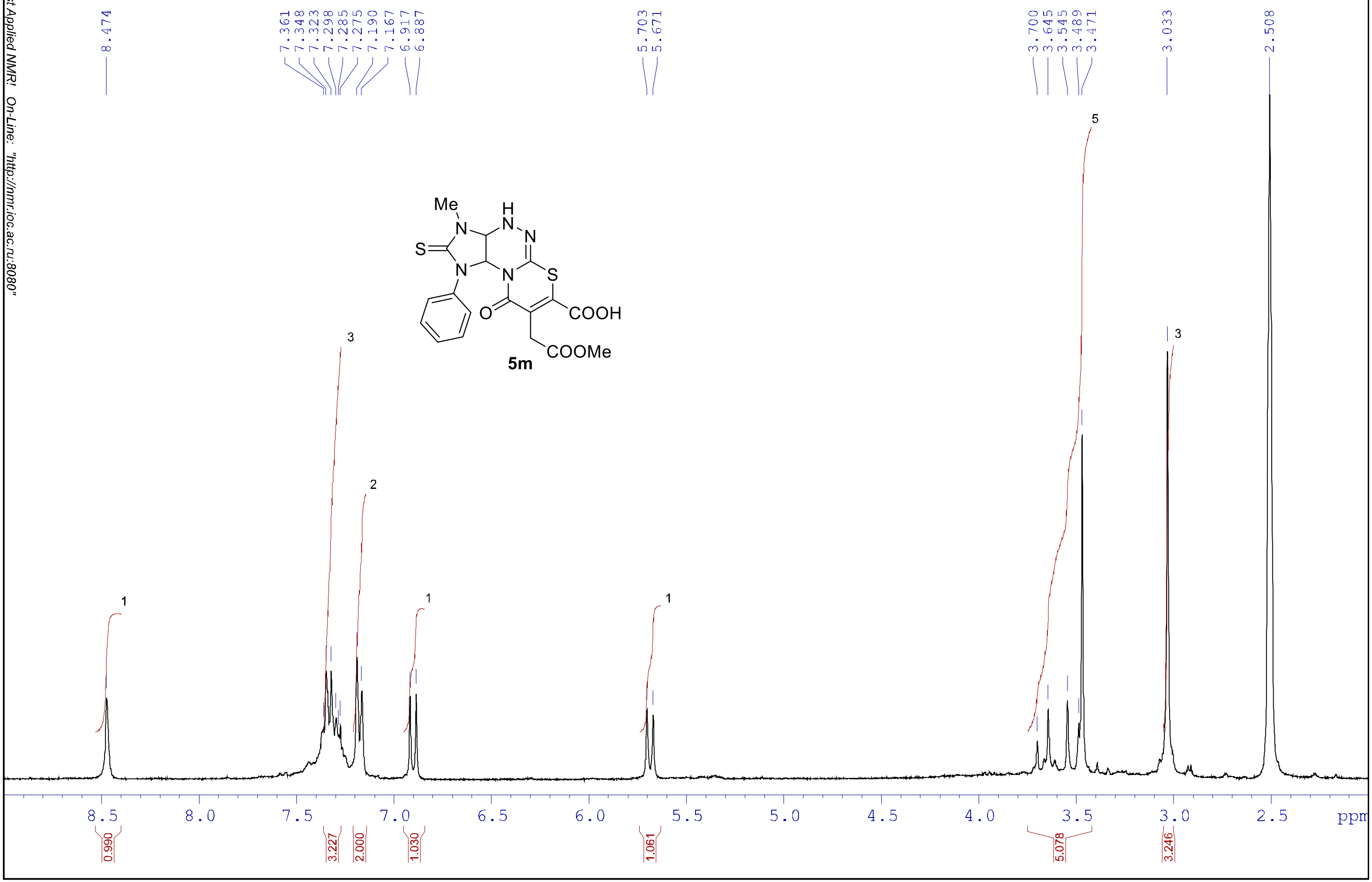

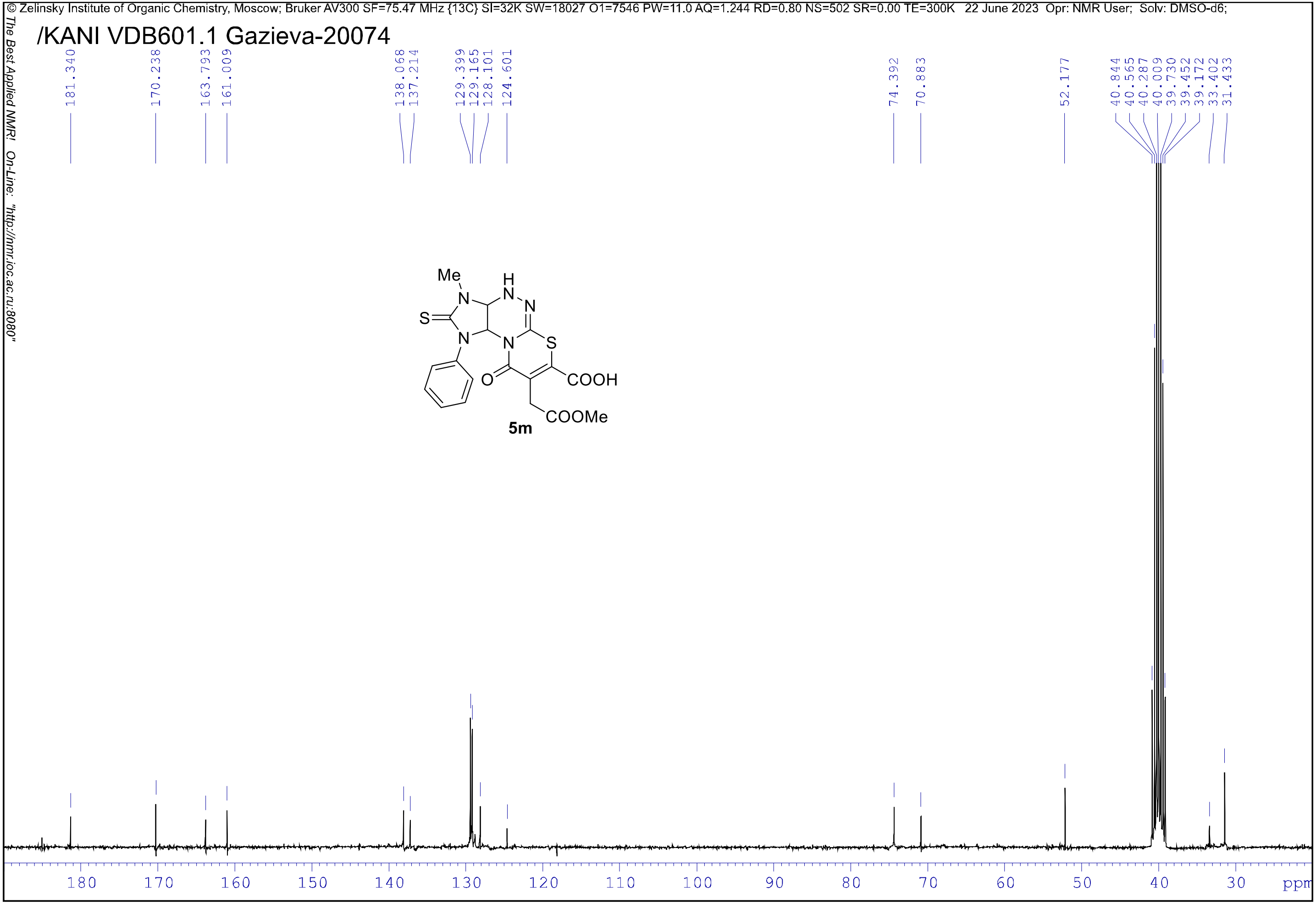
© Zelinsky Institute of Organic Chemistry, Moscow; Bruker AV300 SF=75.47 MHz {13C} SI=32K SW=18027 O1=7546 PW=11.0 AQ=1.244 RD=0.80 NS=502 SR=0.00 TE=300K 22 June 2023 Opr: NMR User; Solv: DMSO-d6;
The Best Applied NMR! On-Line: "http://nmr.ioc.ac.ru:8080"
/KANI VDB601.1 Gazieva-20074
181.340
170.238
163.793
161.009
138.068
137.214
129.399
129.165
128.101
124.601
74.392
70.883
52.177
40.844
40.565
40.287
40.009
39.730
39.452
39.172
33.402
31.433
Me
H
N
N
N
S
N
N
S
O
COOH
COOMe
5m
180
170
160
150
140
130
120
110
100
90
80
70
60
50
40
30
ppm

© Zelinsky Institute of Organic Chemistry, Moscow; Bruker AM300 SF=300.13 MHz {1H} SI=32K SW=6002 O1=2401 PW=9.0 AQ=1.352 RD=0.00 NS=1 SR=1.70 TE=301K 23 December 2020 Opr: Daeva E.D.; Solv: DMSO-d6;

The Best Applied NMR! On-Line: "http://nmr.ioc.ac.ru:8080"

/KANI Kao86.4

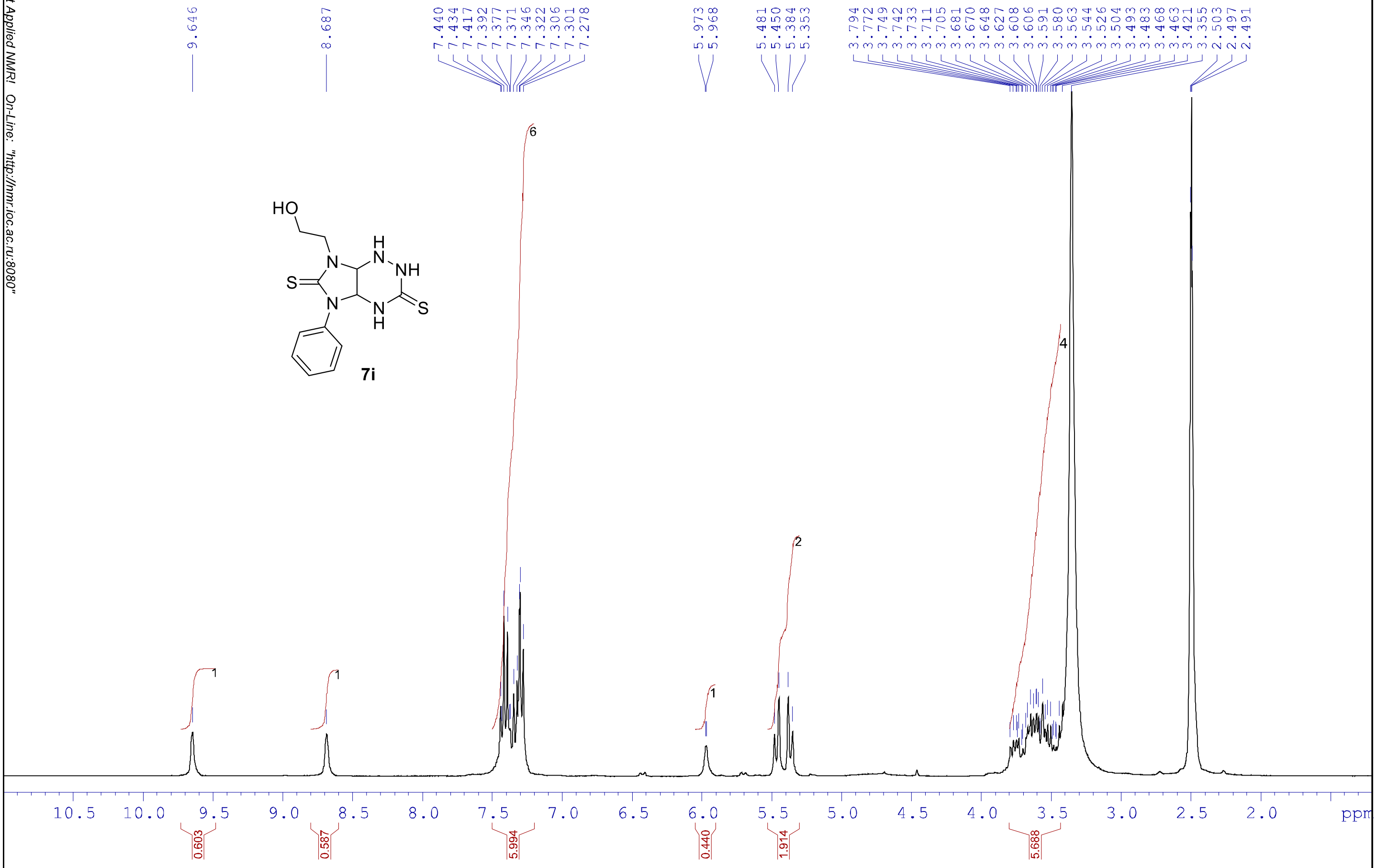

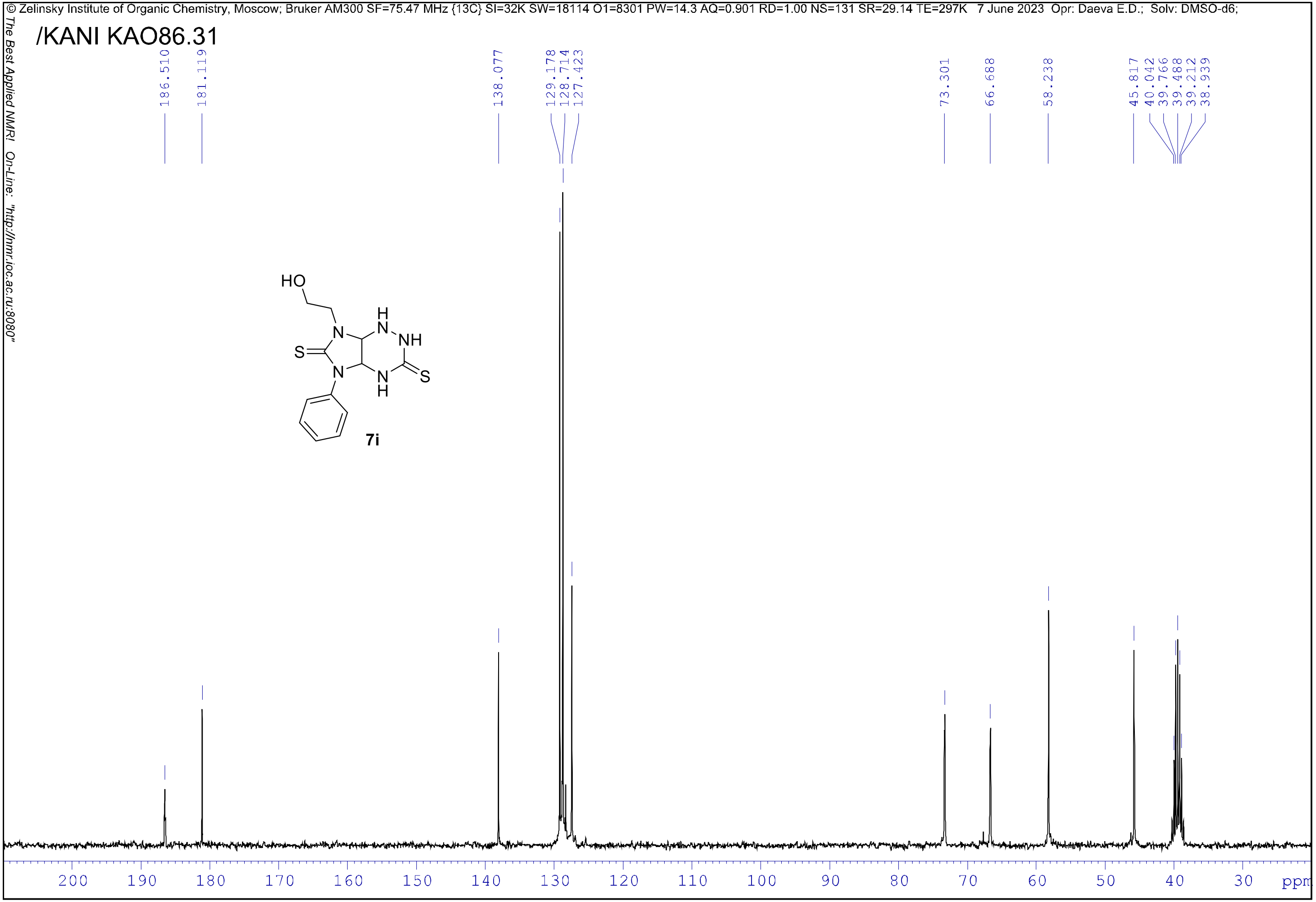

© Zelinsky Institute of Organic Chemistry, Moscow; Bruker AM300 SF=75.47 MHz {13C} SI=32K SW=18114 O1=8301 PW=14.3 AQ=0.901 RD=1.00 NS=131 SR=29.14 TE=297K 7 June 2023 Opr: Daeva E.D.; Solv: DMSO-d6;
The Best Applied NMR! On-Line: "http://nmr.ioc.ac.ru:8080"
/KANI KAO86.31
186.510
181.119
138.077
129.178
128.714
127.423
73.301
66.688
58.238
45.817
40.042
39.766
39.488
39.212
38.939
HO
N
H
N
NH
S
N
N
H
S
7i
200
190
180
170
160
150
140
130
120
110
100
90
80
70
60
50
40
30
ppm

© Zelinsky Institute of Organic Chemistry, Moscow; Bruker AM300 SF=300.13 MHz {1H} SI=32K SW=6002 O1=2401 PW=9.0 AQ=1.352 RD=0.00 NS=1 SR=-4.18 TE=298K 14 June 2023 Opr: Daeva E.D.; Solv: DMSO-d6;

The Best Applied NMR! On-Line: "http://nmr.ioc.ac.ru:8080"

/KANI IA1831.2

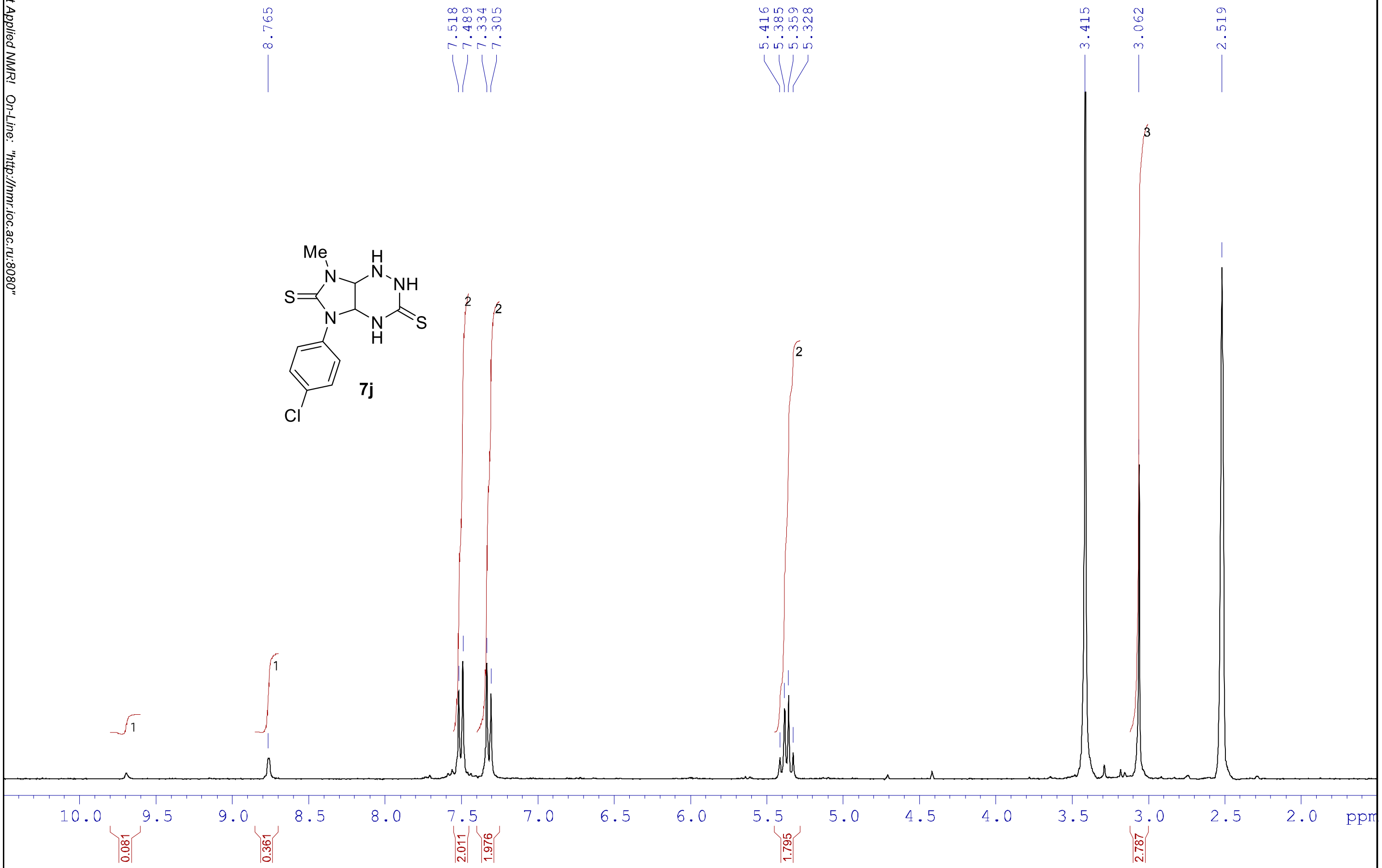

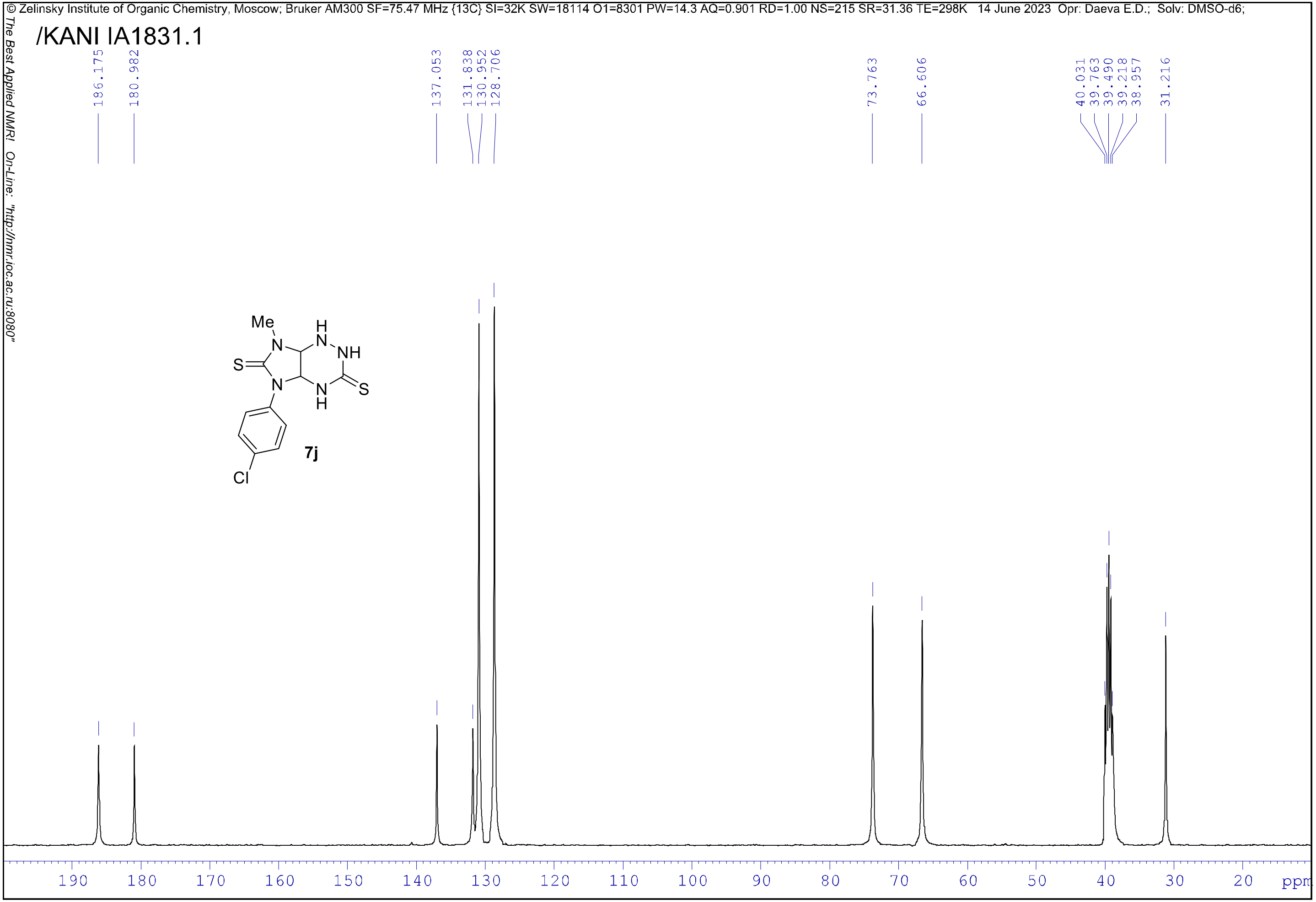
© Zelinsky Institute of Organic Chemistry, Moscow; Bruker AM300 SF=75.47 MHz {13C} SI=32K SW=18114 O1=8301 PW=14.3 AQ=0.901 RD=1.00 NS=215 SR=31.36 TE=298K 14 June 2023 Opr: Daeva E.D.; Solv: DMSO-d6;
The Best Applied NMR! On-Line: "http://nmr.ioc.ac.ru:8080"
/KANI IA1831.1
186.175
180.982
137.053
131.838
130.952
128.706
73.763
66.606
40.031
39.763
39.490
39.218
38.957
31.216
Me
N
H
N
NH
S
N
N
H
S
Cl
7j
190
180
170
160
150
140
130
120
110
100
90
80
70
60
50
40
30
20
ppm

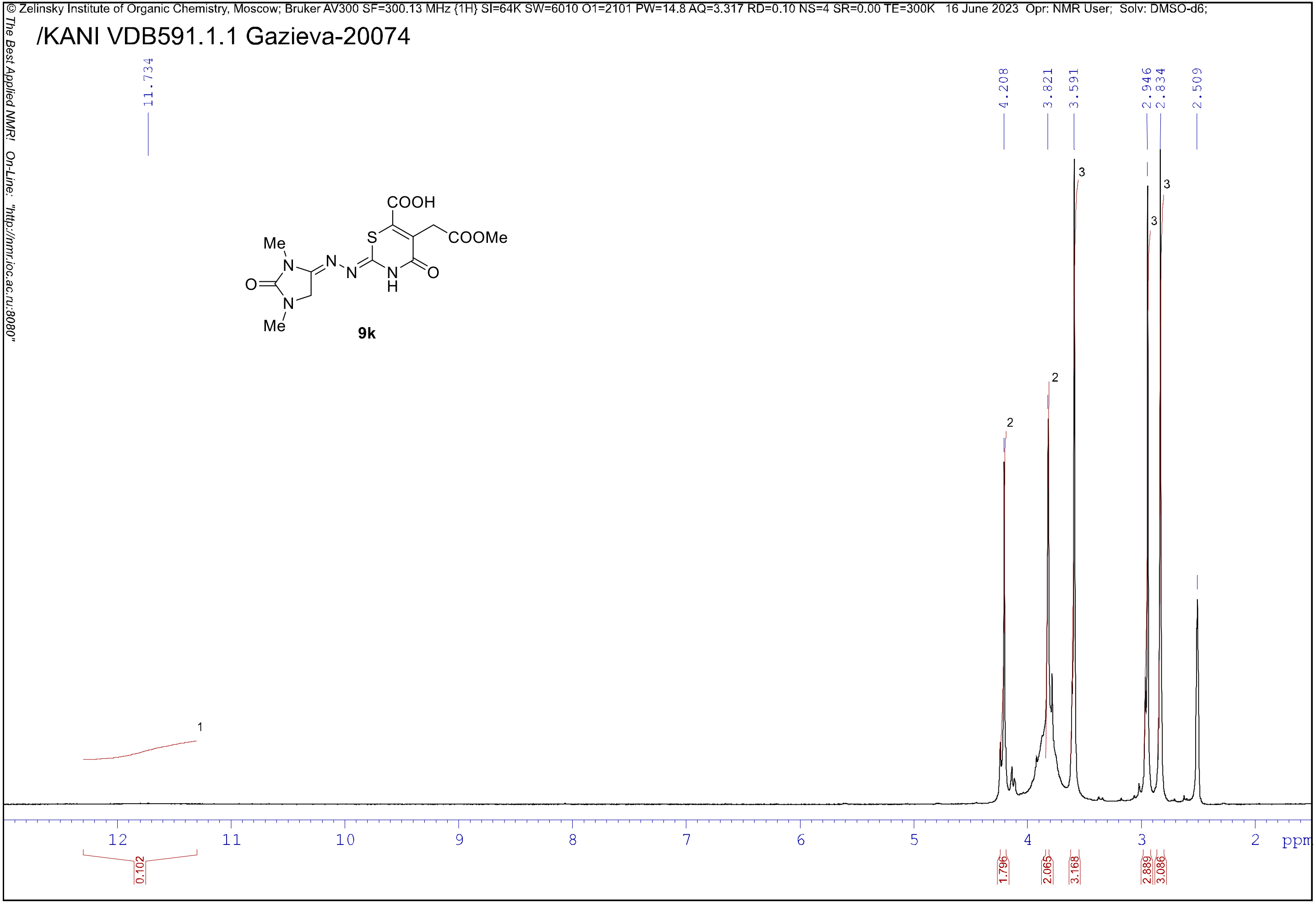
© Zelinsky Institute of Organic Chemistry, Moscow; Bruker AV300 SF=300.13 MHz {1H} SI=64K SW=6010 O1=2101 PW=14.8 AQ=3.317 RD=0.10 NS=4 SR=0.00 TE=300K 16 June 2023 Opr: NMR User; Solv: DMSO-d6;
The Best Applied NMR! On-Line: "http://nmr.ioc.ac.ru:8080"
/KANI VDB591.1.1 Gazieva-20074
11.734
4.208
3.821
3.591
2.946
2.834
2.509
COOH
COOMe
Me
N
S
N
N
N
H
O
O
N
Me
9k
12
11
10
9
8
7
6
5
4
3
2
ppm
0.102
1.796
2.065
3.168
2.889
3.086

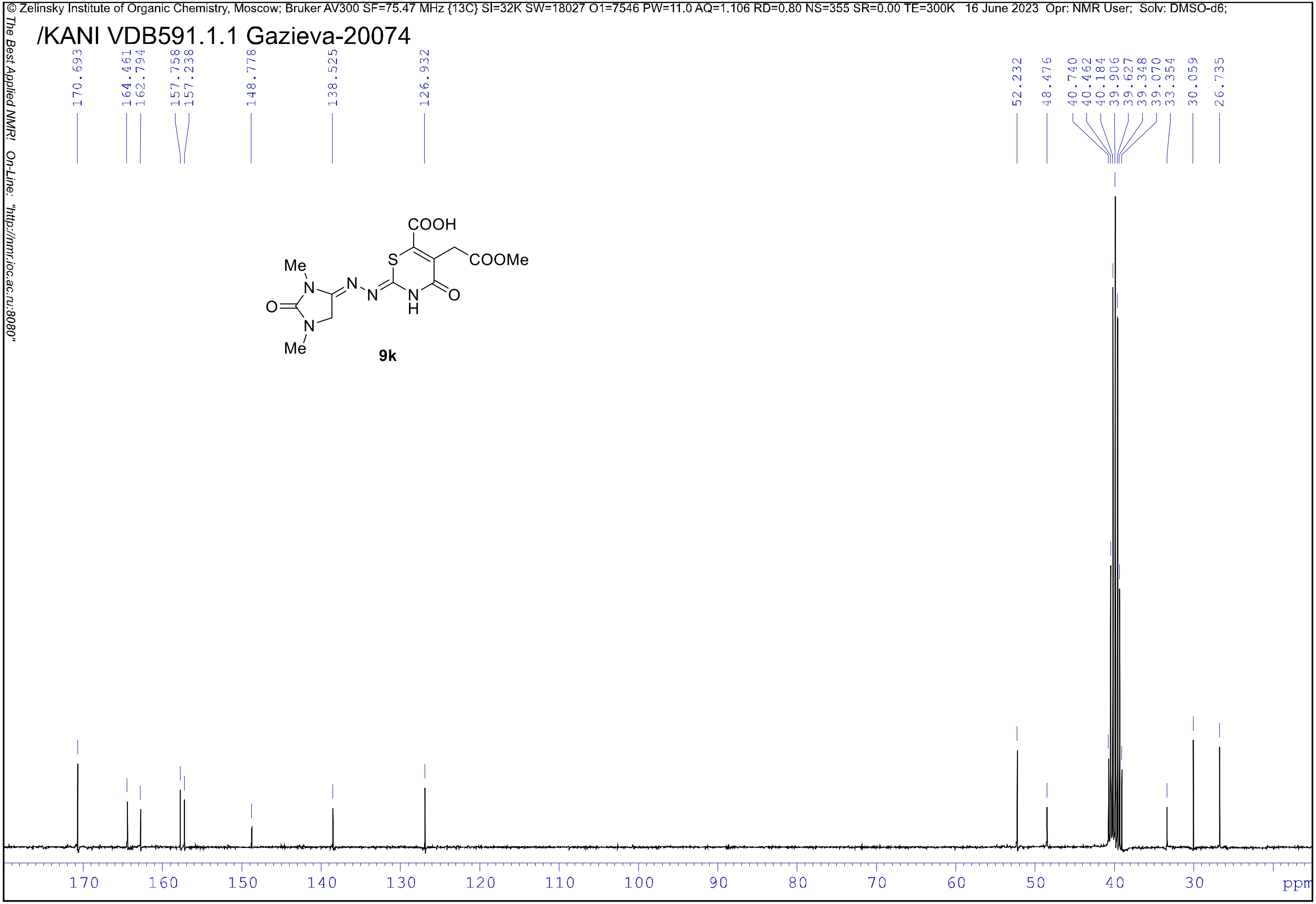

© Zelinsky Institute of Organic Chemistry, Moscow; Bruker AV300 SF=75.47 MHz {13C} SI=32K SW=18027 O1=7546 PW=11.0 AQ=1.106 RD=0.80 NS=355 SR=0.00 TE=300K 16 June 2023 Opr: NMR User; Solv: DMSO-d6;
The Best Applied NMR! On-Line: "http://nmr.ioc.ac.ru:8080"
/KANI VDB591.1.1 Gazieva-20074
170.693
164.461
162.794
157.758
157.238
148.778
138.525
126.932
52.232
48.476
40.740
40.462
40.184
39.906
39.627
39.348
39.070
33.354
30.059
26.735
COOH
COOMe
S
Me
N
N
N
O
N
H
O
N
Me
9k
170
160
150
140
130
120
110
100
90
80
70
60
50
40
30
ppm

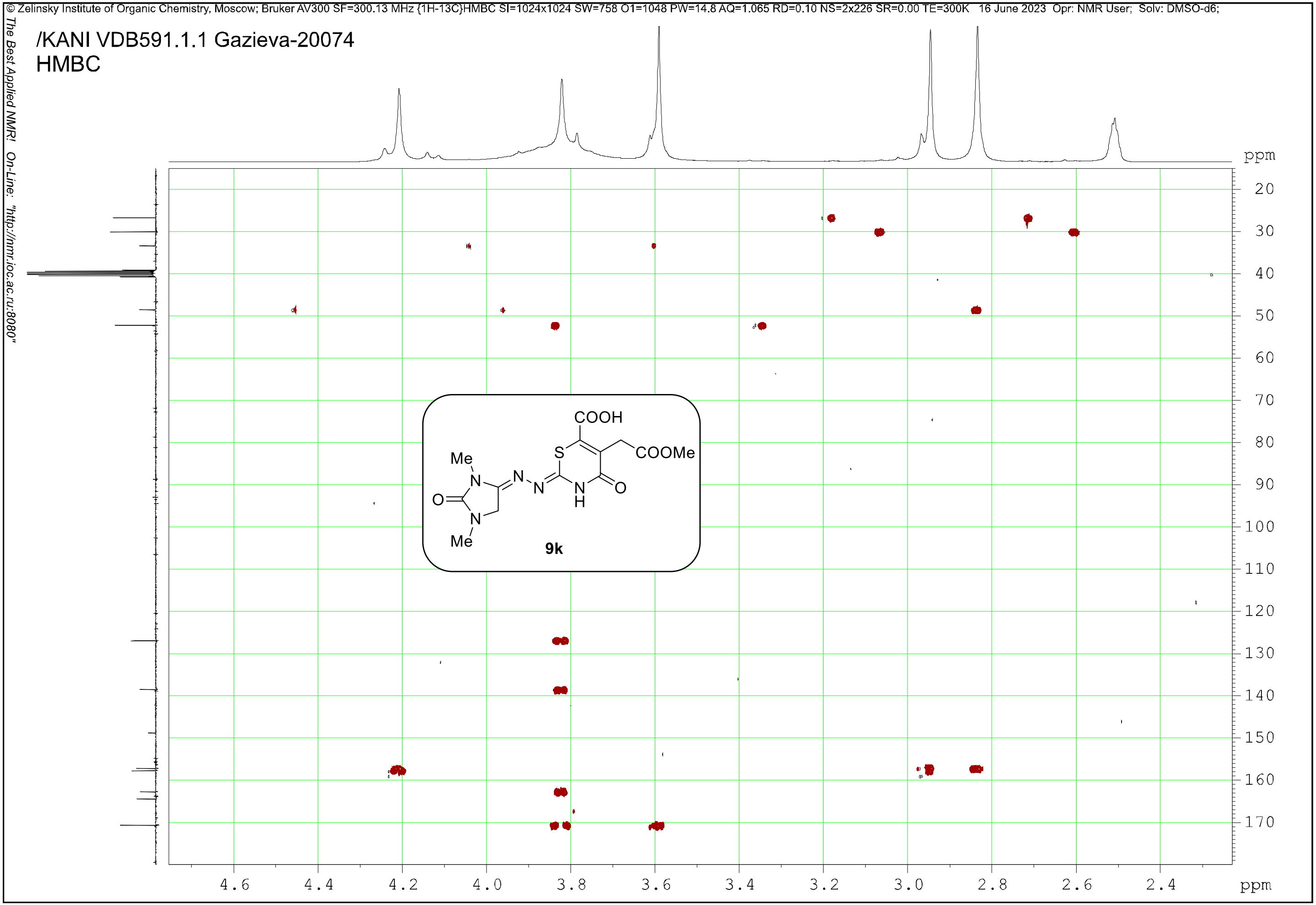
© Zelinsky Institute of Organic Chemistry, Moscow; Bruker AV300 SF=300.13 MHz {1H-13C}HMBC SI=1024x1024 SW=758 O1=1048 PW=14.8 AQ=1.065 RD=0.10 NS=2x226 SR=0.00 TE=300K 16 June 2023 Opr: NMR User; Solv: DMSO-d6;
The Best Applied NMR! On-Line: "http://nmr.ioc.ac.ru:8080"
/KANI VDB591.1.1 Gazieva-20074
HMBC
COOH
COOMe
Me
9k
ppm

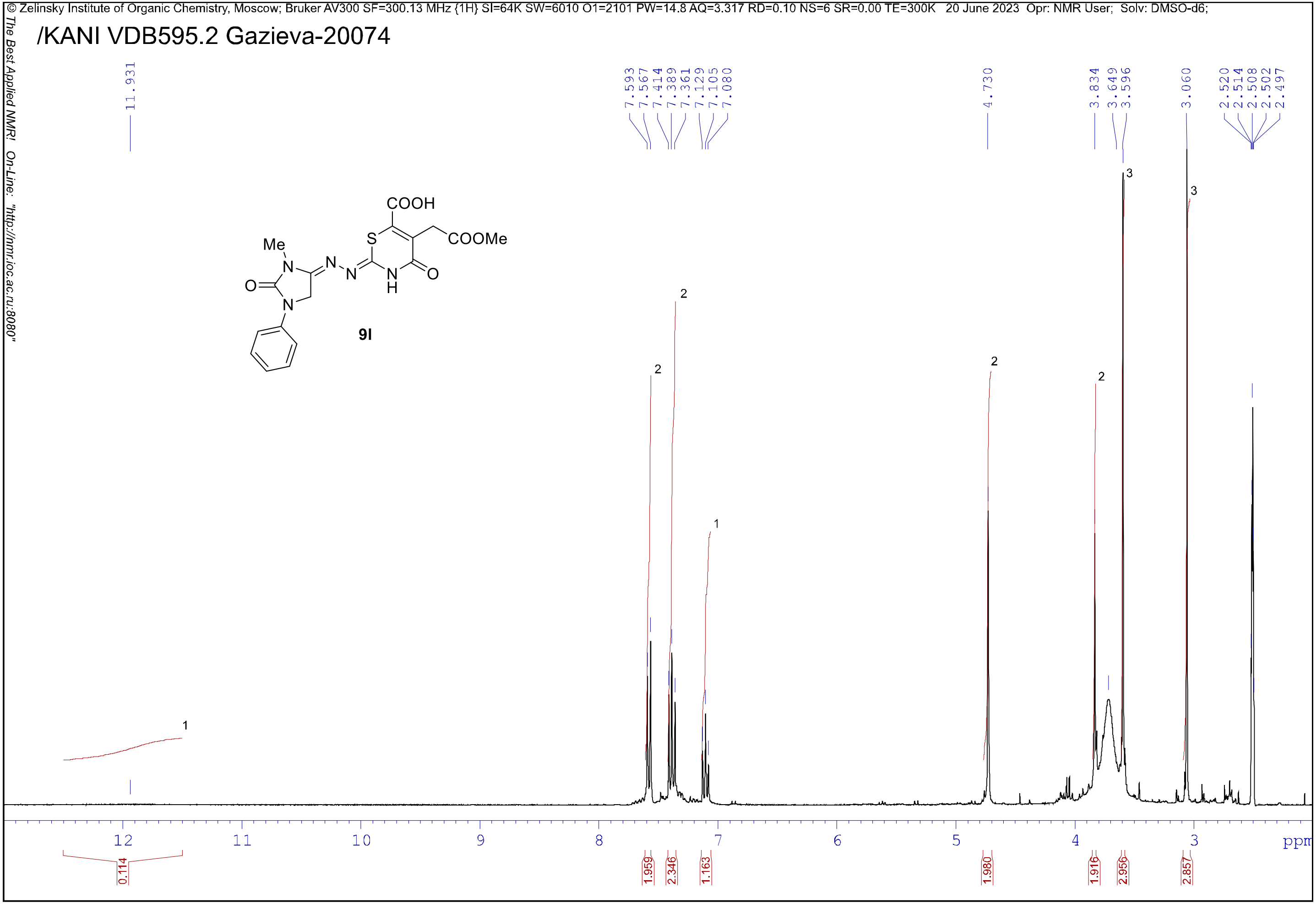
© Zelinsky Institute of Organic Chemistry, Moscow; Bruker AV300 SF=300.13 MHz {1H} SI=64K SW=6010 O1=2101 PW=14.8 AQ=3.317 RD=0.10 NS=6 SR=0.00 TE=300K 20 June 2023 Opr: NMR User; Solv: DMSO-d6;
/KANI VDB595.2 Gazieva-20074
The Best Applied NMR! On-Line: "http://nmr.ioc.ac.ru:8080"
11.931
7.593
7.567
7.414
7.389
7.361
7.129
7.105
7.080
4.730
3.834
3.649
3.596
3.060
2.520
2.514
2.508
2.502
2.497
COOH
COOMe
Me
N
S
N
N
N
H
O
O
N
9l
12
11
10
9
8
7
6
5
4
3
ppm
0.114
1.959
2.346
1.163
1.980
1.916
2.956
2.857

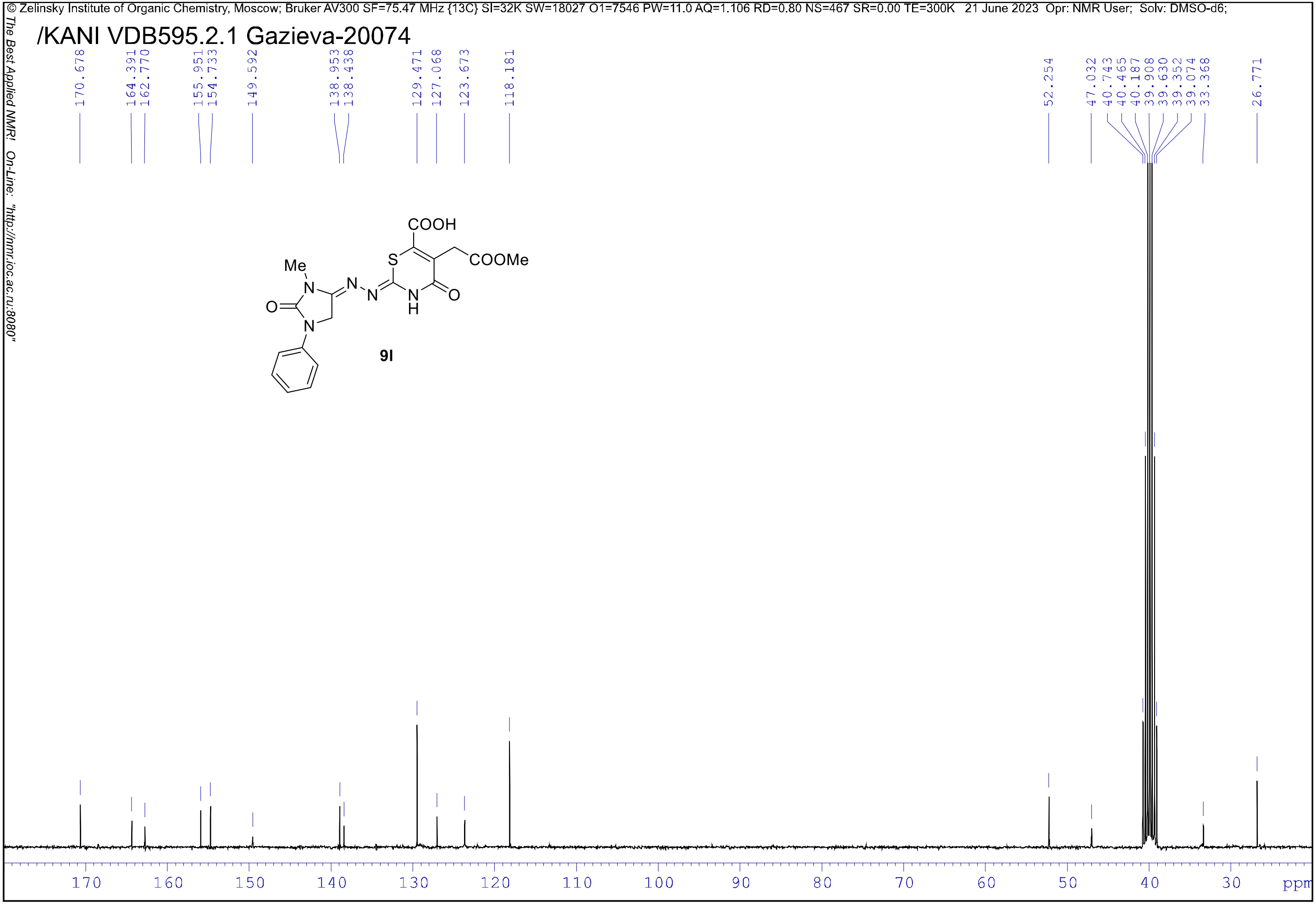

© Zelinsky Institute of Organic Chemistry, Moscow; Bruker AV300 SF=75.47 MHz {13C} SI=32K SW=18027 O1=7546 PW=11.0 AQ=1.106 RD=0.80 NS=467 SR=0.00 TE=300K 21 June 2023 Opr: NMR User; Solv: DMSO-d6;
The Best Applied NMR! On-Line: "http://nmr.ioc.ac.ru:8080"
/KANI VDB595.2.1 Gazieva-20074
170.678
164.391
162.770
155.951
154.733
149.592
138.953
138.438
129.471
127.068
123.673
118.181
52.254
47.032
40.743
40.465
40.187
39.908
39.630
39.352
39.074
33.368
26.771
COOH
S
COOMe
Me
N
N
N
O
N
H
O
N
9l
170
160
150
140
130
120
110
100
90
80
70
60
50
40
30
ppm

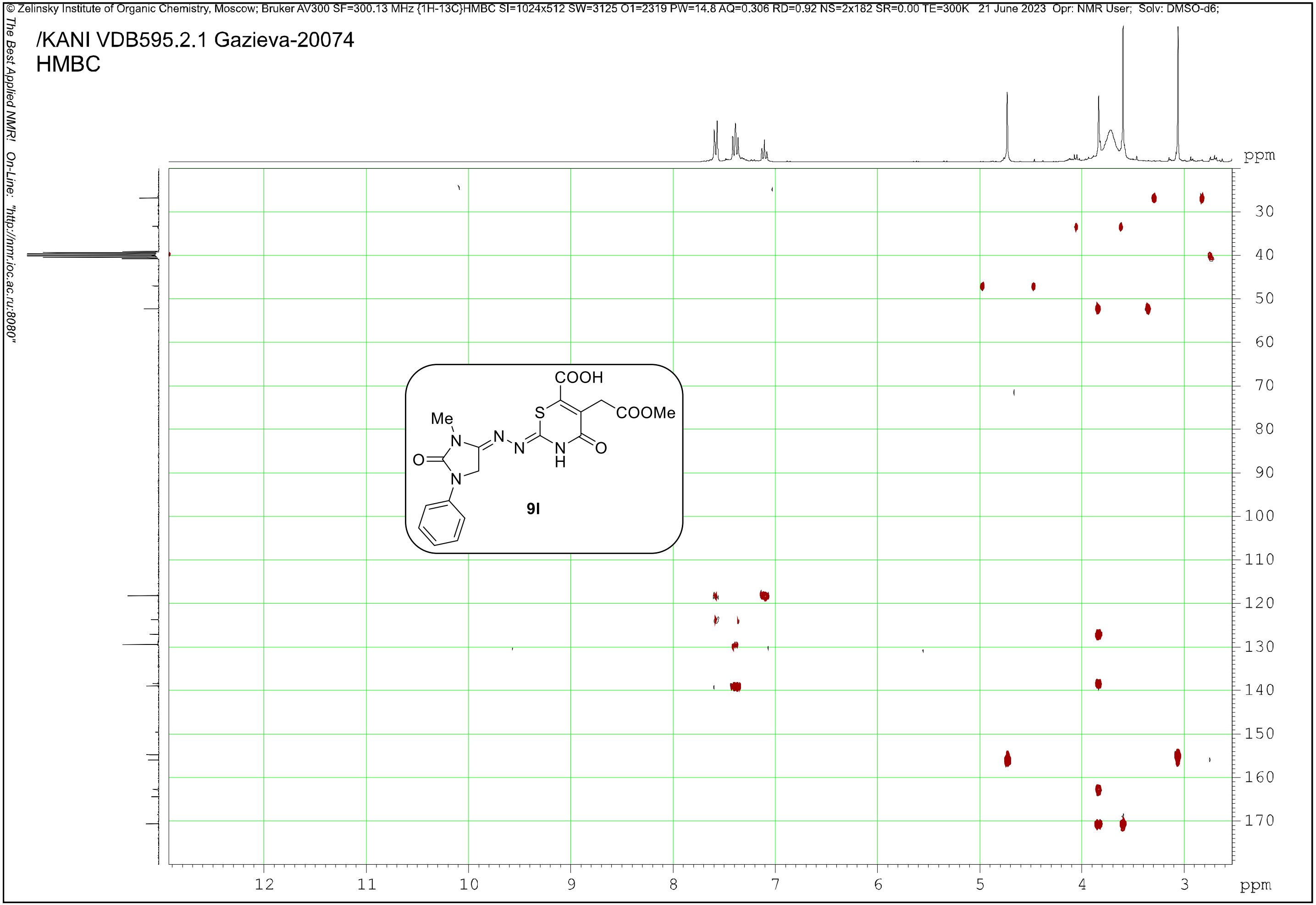
© Zelinsky Institute of Organic Chemistry, Moscow; Bruker AV300 SF=300.13 MHz {1H-13C}HMBC SI=1024x512 SW=3125 O1=2319 PW=14.8 AQ=0.306 RD=0.92 NS=2x182 SR=0.00 TE=300K 21 June 2023 Opr: NMR User; Solv: DMSO-d6;
The Best Applied NMR! On-Line: "http://nmr.ioc.ac.ru:8080"
/KANI VDB595.2.1 Gazieva-20074
HMBC
ppm
30
40
50
60
70
80
90
100
110
120
130
140
150
160
170
12
11
10
9
8
7
6
5
4
3
ppm
COOH
S
COOMe
Me
N
N
N
O
N
H
O
N
9l

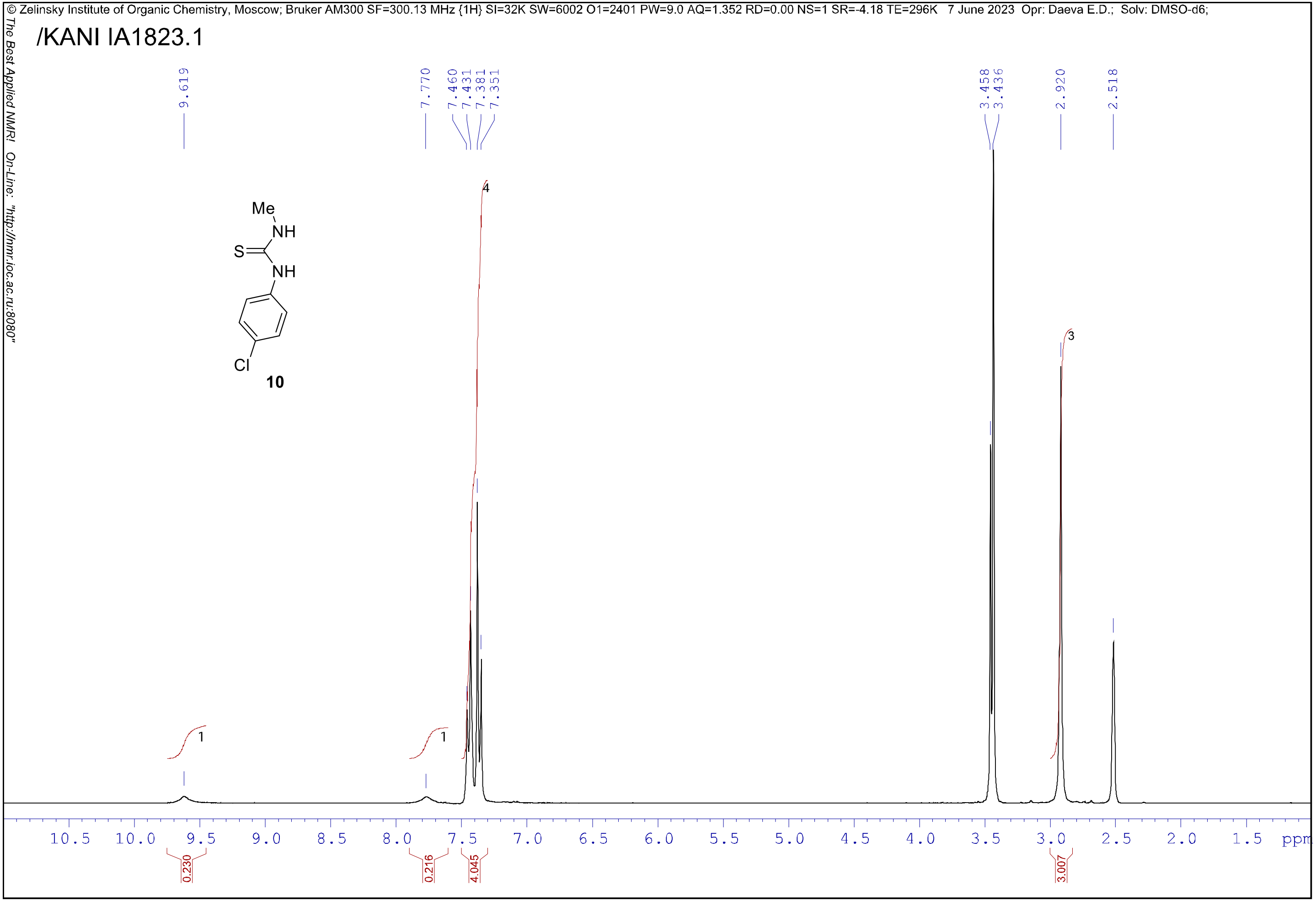
© Zelinsky Institute of Organic Chemistry, Moscow; Bruker AM300 SF=300.13 MHz {1H} SI=32K SW=6002 O1=2401 PW=9.0 AQ=1.352 RD=0.00 NS=1 SR=-4.18 TE=296K 7 June 2023 Opr: Daeva E.D.; Solv: DMSO-d6;
/KANI IA1823.1
The Best Applied NMR! On-Line: "http://nmr.ioc.ac.ru:8080"
9.619
7.770
7.460
7.431
7.381
7.351
3.458
3.436
2.920
2.518
Me
NH
S
NH
Cl
10
1
1
4
3
10.5
10.0
9.5
9.0
8.5
8.0
7.5
7.0
6.5
6.0
5.5
5.0
4.5
4.0
3.5
3.0
2.5
2.0
1.5
ppm
0.230
0.216
4.045
3.007

© Zelinsky Institute of Organic Chemistry, Moscow; Bruker AM300 SF=75.47 MHz {13C} SI=32K SW=18114 O1=8301 PW=14.3 AQ=0.901 RD=1.00 NS=294 SR=28.53 TE=297K 8 June 2023 Opr: Daeva E.D.; Solv: DMSO-d6;

The Best Applied NMR! On-Line: "http://nmr.ioc.ac.ru:8080"

/KANI IA1823.1

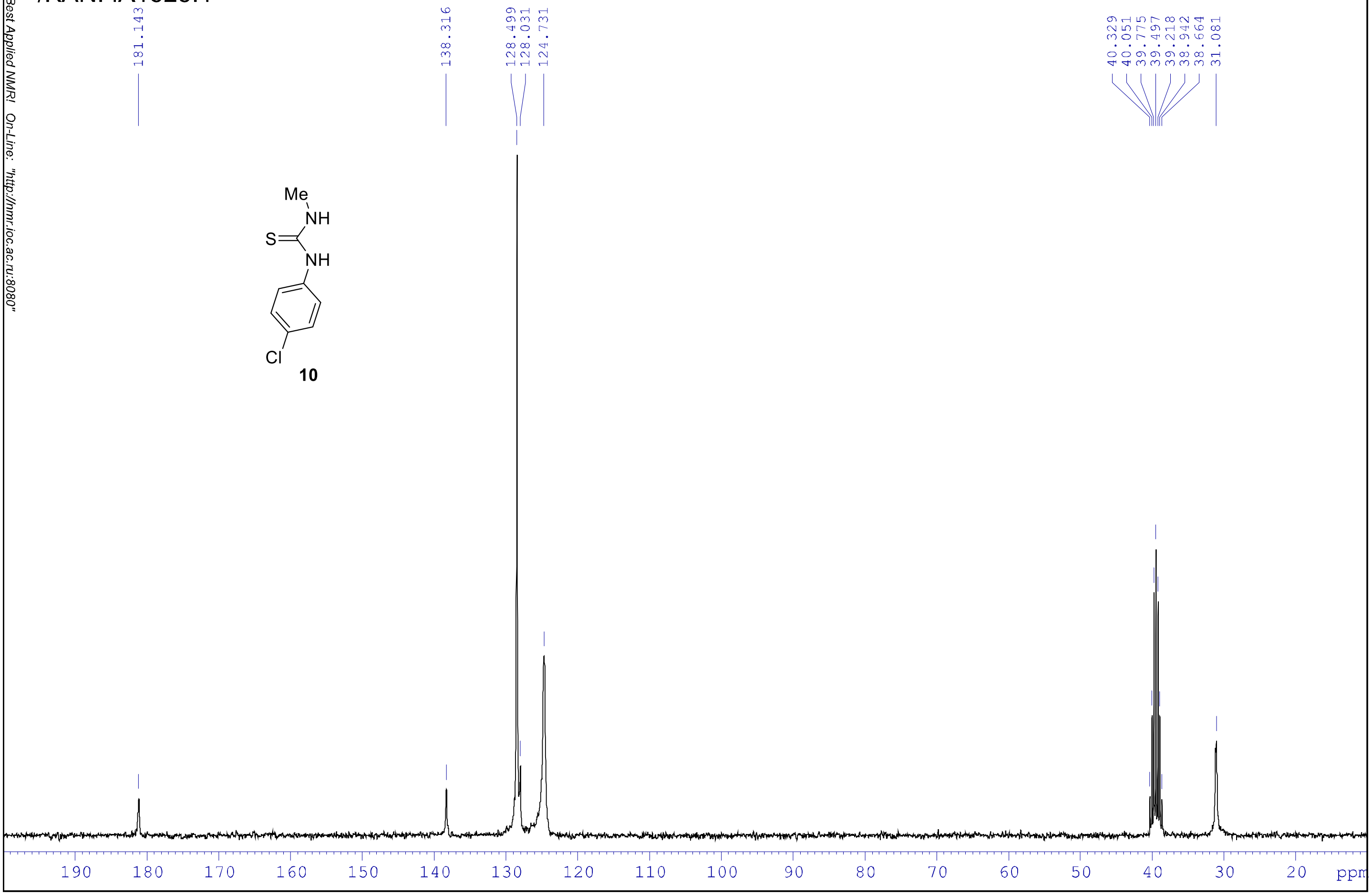

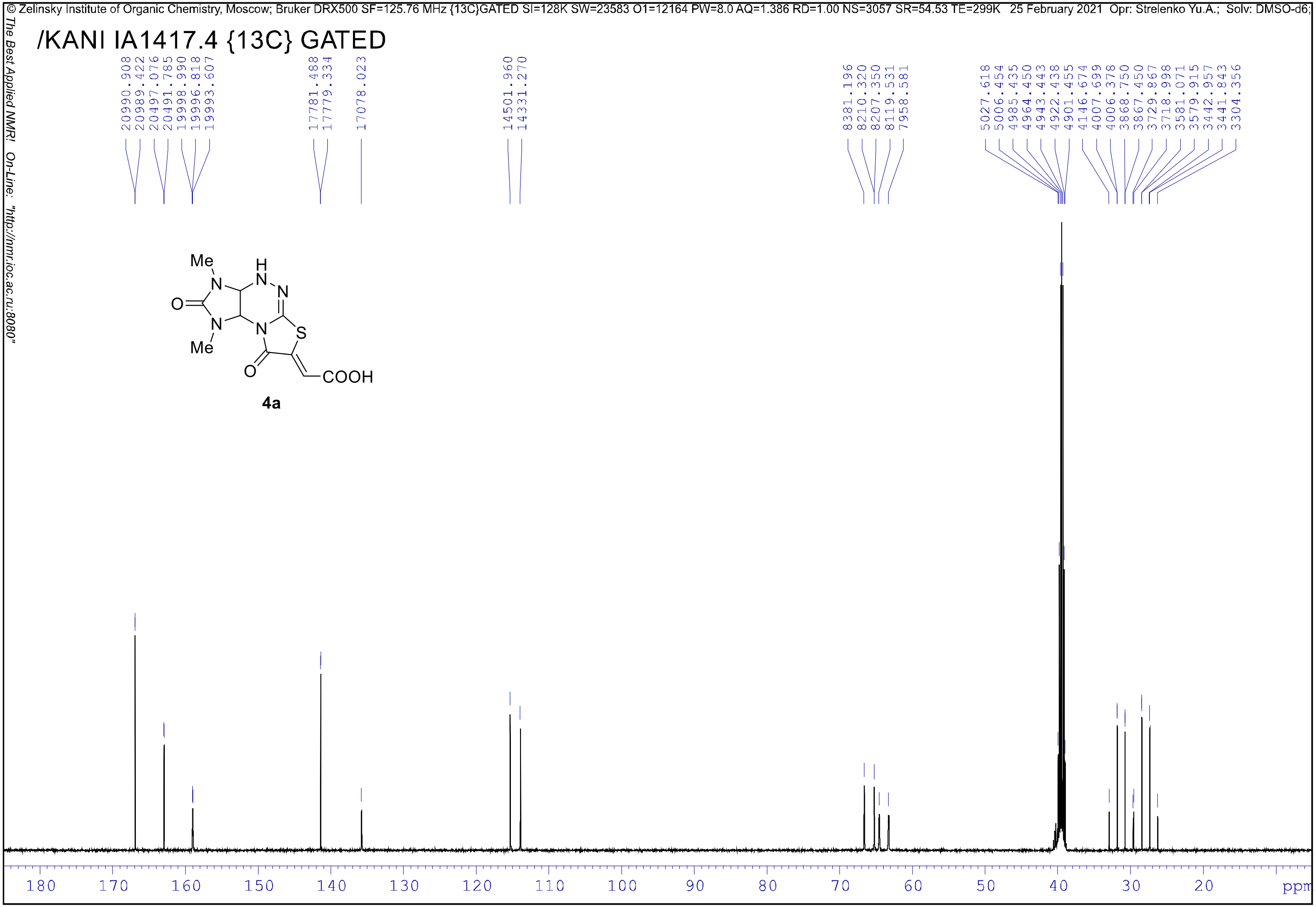
© Zelinsky Institute of Organic Chemistry, Moscow; Bruker DRX500 SF=125.76 MHz {13C}GATED SI=128K SW=23583 O1=12164 PW=8.0 AQ=1.386 RD=1.00 NS=3057 SR=54.53 TE=299K 25 February 2021 Opr: Strelenko Yu.A.; Solv: DMSO-d6;
The Best Applied NMR! On-Line: "http://nmr.ioc.ac.ru:8080"
/KANI IA1417.4 {13C} GATED
20990.908
20989.422
20497.076
20491.785
19998.990
19996.818
19993.607
17781.488
17779.334
17078.023
14501.960
14331.270
8381.196
8210.320
8207.350
8119.531
7958.581
5027.618
5006.454
4985.435
4964.450
4943.443
4922.438
4901.455
4146.674
4007.699
4006.378
3868.750
3867.450
3729.867
3718.998
3581.071
3579.915
3442.957
3441.843
3304.356
Me
H
N
N
N
O
N
N
Me
S
O
COOH
4a
180
170
160
150
140
130
120
110
100
90
80
70
60
50
40
30
20
ppm

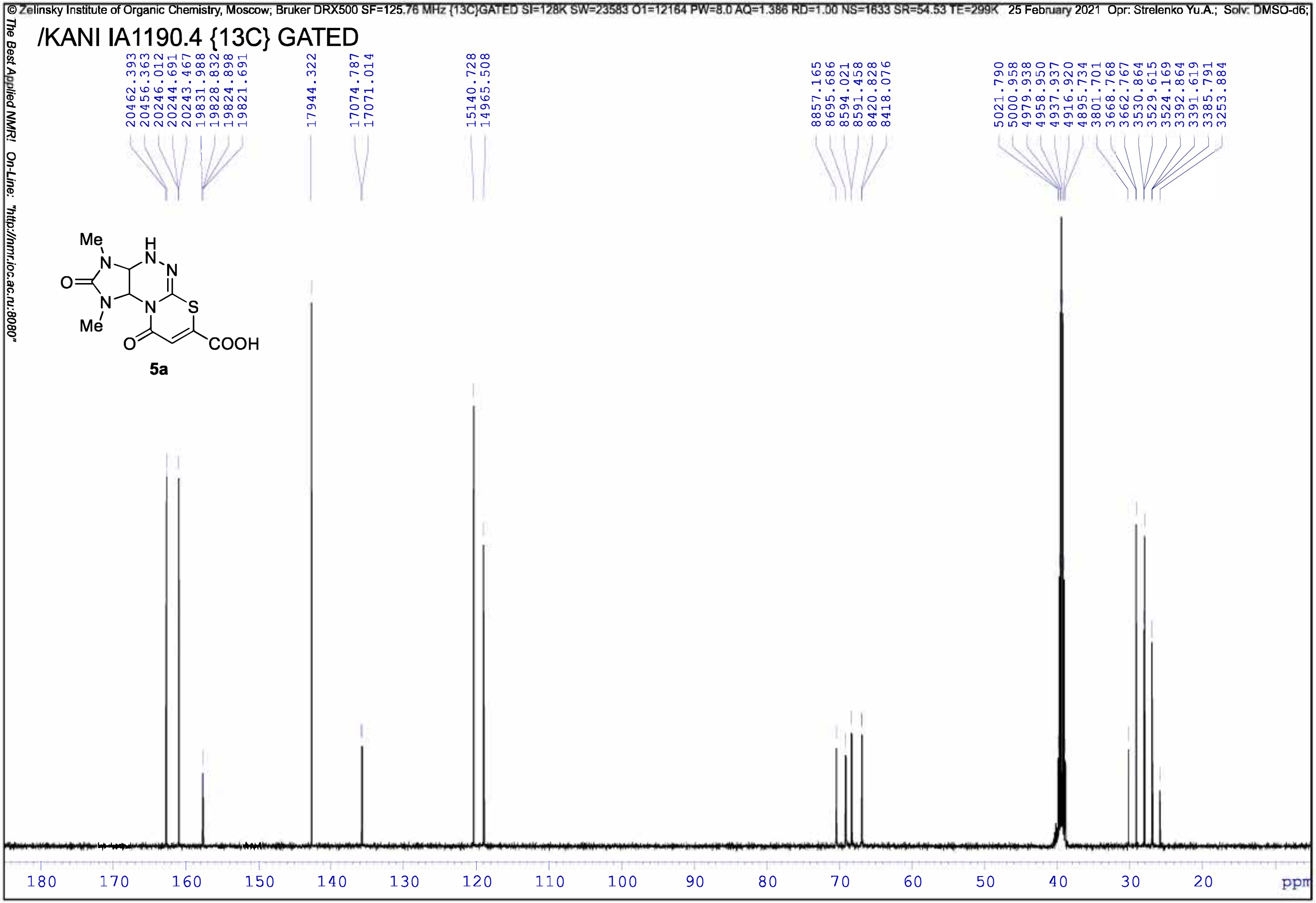
© Zelinsky Institute of Organic Chemistry, Moscow; Bruker DRX500 SF=125.76 MHz {13C}GATED SI=128K SW=23583 O1=12164 PW=8.0 AQ=1.386 RD=1.00 NS=1633 SR=54.53 TE=299K 25 February 2021 Opr: Strelenko Yu.A.; Solv: DMSO-d6;
/KANI IA1190.4 {13C} GATED
The Best Applied NMR! On-Line: "http://nmr.ioc.ac.ru:8080"
20462.393
20456.363
20246.012
20244.691
20243.467
19831.988
19828.832
19824.898
19821.691
17944.322
17074.787
17071.014
15140.728
14965.508
8857.165
8695.686
8594.021
8591.458
8420.828
8418.076
5021.790
5000.958
4979.938
4958.950
4937.937
4916.920
4895.734
3801.701
3668.768
3662.767
3530.864
3529.615
3524.169
3392.864
3391.619
3385.791
3253.884
Me
H
N
N
O
N
N
S
Me
O
COOH
5a
180 170 160 150 140 130 120 110 100 90 80 70 60 50 40 30 20 ppm
